# Sila-spirocyclization involving unstrained C(sp^3^)−Si bond cleavage

**DOI:** 10.1038/s41467-022-34466-4

**Published:** 2022-11-05

**Authors:** Yufeng Shi, Xiaonan Shi, Jinyu Zhang, Ying Qin, Bo Li, Dongbing Zhao

**Affiliations:** 1 State Key Laboratory and Institute of Elemento-Organic Chemistry, Haihe Laboratory of Sustainable Chemical Transformations, College of Chemistry, Nankai University, Tianjin, 300071 China; 2 Division of Chemistry and Chemical Engineering, California Institute of Technology, Pasadena, CA 91106 USA

**Keywords:** Synthetic chemistry methodology, Chemical synthesis, Homogeneous catalysis

## Abstract

C − Si Bond cleavage is one of the key elemental steps for a wide variety of silicon-based transformations. However, the cleavage of unstrained Si−C(sp^3^) bonds catalyzed by transition metal are still in their infancy. They generally involve the insertion of a M − C(sp^2^) species into the C − Si bond and consequent intramolecular C(sp^2^)‒Si coupling to exclusively produce siloles. Here we report the Pd-catalyzed sila-spirocyclization, in which the Si−C(sp^3^) bond is activated by the insertion of a M − C(sp^3^) species and followed by the formation of a new C(sp^3^)‒Si bond, allowing the construction of diverse spirosilacycles. This reactivity mode, which is strongly supported by DFT calculations may open an avenue for the Si−C(sp^3^) bond cleavage and silacycle synthesis.

## Abstract (web summary)

Methods that enable the cleavage of Si−C(sp^3^) bonds catalytically remain rare. Here the authors report the synthesis of spirosilacycles via palladium-catalysed spiro-sila-cyclization of trialkylsilane derivatives; the reaction proceeds via cleavage of a C(sp^3^)−Si bond and insertion of an alkylpalladium complex.

## Introduction

**Fig. 1:**
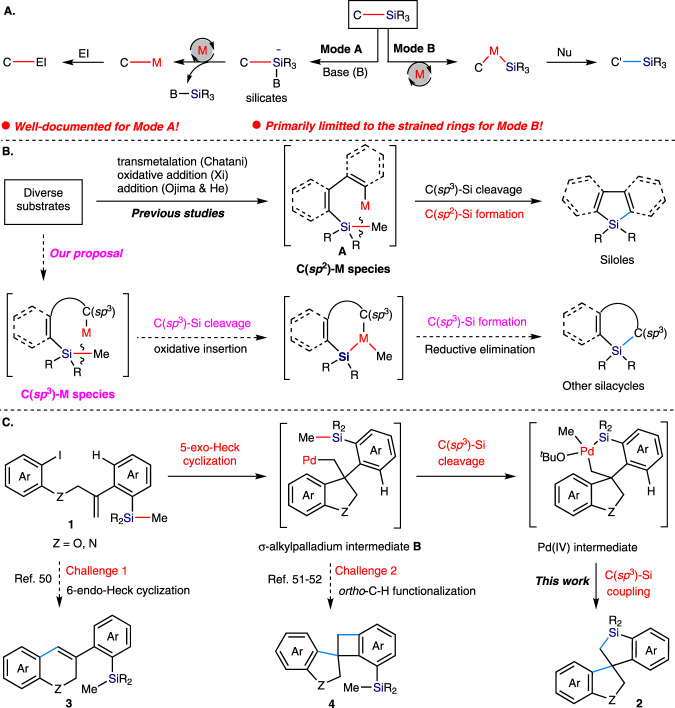
Current state and our study for unstrained Si−C(sp^3^) bond activiation by transition metal insertion. **A** Two general activation modes for C–Si bond cleavage in transition metal catalysis. **B** Literatures and our proposal on unstrained C(sp^3^)-Si activation by transition metal insertion. **C** Our work and possible challenges on Pd-catalyzed sila-spirocyclization involving C(sp^3^)‒Si bond activation via the insertion of a Pd−C(sp^3^) species.

Developing protocols that afford catalytic C–Si bond cleavage is a longstanding goal in transition metal catalysis^1–5^, because it has a substantial impact on the retrosynthetic analysis and subsequent synthesis of organosilicon compounds used in many disciplines^6–11^. Classic mode of C–Si bond activation in transition metal catalysis involved the generation of discrete hypervalent silicon species^12–19^, thus promoting the subsequent transmetalation to form C–M intermediate (Fig. 1A, Mode A), and the silicon moiety is finally removed as a byproduct. In recent years, the C–Si bond activation by oxidative insertion of transition metal (Fig. 1A, Mode B) is attracting growing attention^20,21^. Cleavage of the C–Si bond is followed by the formation of a new Si–C bond in this reaction mode, which provides the chance to functionalize the C–Si bond and create new organosilicon compounds such as diverse silacycles. However, such an elementary step is very challenging. The current scope of C–Si bond that can be activated via Mode B is primarily restricted to the silacycles with a small ring size^22–35^, in which strain release provides thermodynamic driving forces. The catalytic cleavage of unstrained Si−C(sp^3^) bonds by transition metal insertion is still exceedingly rare. Those examples generally involved the insertion of a M−C(sp^2^) species **A** into the Si−C(sp^3^) bond along with the formation of a new Si–C(sp^2^) bond in an intramolecular fashion and exclusively produced silole derivatives (Fig. 1B)^36–43^, thus limiting its synthetic versatility. We wonder if a M−C(sp^3^) species could also insert into the Si−C(sp^3^) bond, followed by the production of a new Si–C(sp^3^) bond, thus providing an entry to diverse silacycles but not siloles.

Inspired by the tremendous progress in utilization of C(sp^3^)−Pd species for domino processes, which generated by intramolecular carbopalladation of alkenes^44–48^, we therefore designed the alkene-tethered aryl iodide **1** bearing a trialkyl silyl group to verify the possibility of C(sp^3^)−Si bond activation via the insertion of a metal−C(sp^3^) species. We envisioned that the substrate **1** would undergo a intramolecular 5-exo selective carbopalladation to deliver σ-alkylpalladium intermediate **B** with lack of β-H, which might possess similar reactivity to that of M−C(sp^2^) species **A** and thus be competent for insertion into the C(sp^3^)−Si bond, followed by reductive elimination to produce new C(sp^3^)−Si bond (Fig. 1C). If successful, the reaction would constitute an alternative to construction of structurally unique spirosilacycles **2**. Spirocyclic scaffolds have been found in a wide range of bioactive natural products and are widely incorporated in various approved drugs and catalysts. Even, a plenty of methods have been disclosed for rapid access to spirocycles, the catalytic methods for preparation of spirosilacycles are still rare^49^ and highly desired because of the popularity of C/Si switch in drug discovery. However, the following challenges may need to be addressed to realize our proposed sila-spirocyclization reaction: (1) the competitive 6-endo-selective Heck cyclization^50^; (2) ortho-C−H functionalization of σ-alkylpalladium intermediate **B** to deliver the spiro-fused benzocyclobutenes **4** (Fig. 1C)^51,52^.

In this work, we minimize the formation of side products by deliberately tuning the reaction condition and finally realize the Pd-catalyzed sila-spirocyclization reaction involving a sequential carbopalladation, C(sp^3^)‒Si bond cleavage/coupling. This work provides a method that allows facile access to diverse spirosilacycles in good yields. Notably, Si–C(sp^3^) bond cleavage via the insertion of an M−C(sp^3^) species observed in our study may open opportunities in the synthetic quest for organosilicon compounds.

## Results and discussion

### Reaction conditions optimization

To test the hypothesis, we decided to employ **1aa** as a model substrate to begin our study using Xi’s Pd-catalyzed procedure^39^. The reaction did indeed result in the formation of trace amounts of desired spirosilacycle **2aa**. To significantly improve the yield, we performed an exhaustive screening of a range of metal precatalysts, bases, additives, ligands and solvents (see Supplementary Table 1). Finally, a 90% isolated yield of **2aa** was obtained with **[Pd]−1** (5 mol%) as the (pre)catalyst, LiO^*t*^Bu (3.0 eq) as the base, together with AgOAc (2.0 eq) and copper(I) thiophene-2-carboxylate (CuTc; 0.2 eq) as additives in cyclohexane (0.4 M) at 125 °C for 12 h (Table 1, entry 1). A series of control experiments were then performed to investigate the effect of each components. Unsurprisingly, in the absence of the palladium, no desired product was formed (Entry 2). The LiO^*t*^Bu is also indispensable to trigger this reaction (Entry 3). We reasoned that the I/O^*t*^Bu exchange in reaction intermediate may have happened to decrease the reaction activation energy. The presence of CuTc might promote the C(sp^3^)-Si bond cleavage^18^, thus facilitating 5-exo cyclization pathway (Entry 4). Next, we proved that acetate ion is crucial to improve the turnover of the catalyst, therefore securing the high yield (Entries 5–8). It would shut down the reaction entirely if replacement of the **[Pd]−1** by [Pd(allyl)Cl]_2_ (Entry 9). Pd(P^*t*^Bu_3_)_2_ is capable of initiating the reaction in 66% yield (Entry 10). These results indicate that the reaction may be initiated by Pd(0) species. By employment of toluene as the solvent, the reaction offered a 70% yield of **2aa** along with a considerable amount of 6-endo-cyclization byproduct **3aa** (Entry 11). Finally, a lower catalyst loading or lower temperature stunted the formation of **2aa** (Entry 12 and 13). Notably, the structure of the desired product **2aa** was clearly confirmed by X-ray analysis of the single crystal. We have also successfully scaled up the reaction and carried out the reaction under an air atmosphere by using **1aa** as the substrate. The reaction also proceed smoothly without a significant loss in yield (75% yield obtained at a 2.5 mmol scale; 80% yield obtained under an air atmosphere).

**Table 1:** Condition optimization^*a*^


Entry	Variations from the “standard” conditions	Yield of 2aa [%]^b^	Yield of 3aa [%]^b^
1	None	93 (90)	trace
2	No Pd	N.R.	N.R.
3	No LiO^*t*^Bu	N.R.	N.R.
4	No CuTc	67	28
5	No AgOAc	20	10
6	AgOAc (1.5 eq)	75	12
7	KOAc instead of AgOAc	85(86)	trace
8	Ag_2_CO_3_ instead of AgOAc	20	trace
9	[Pd(allyl)Cl]_2_ instead of **[Pd]-1**	N.R.	N.R.
10	Pd(P^*t*^Bu_3_)_2_ instead of **[Pd]-1**	70(66)	19
11	Toluene instead of cyclohexane	70	12
12	2.5 mol% **[Pd]-1**	45	<10
13	100 °C instead of 125 °C	60	22

^*a*^Reactions were carried out by using **[Pd]-1** (5 mol%), TcCu (20 mol%), AgOAc (2 eq), LiO^*t*^Bu (3 eq) and **1aa** (0.2 mmol) in cyclohexane (0.4 M) at 125 °C for 12 h. *N.R.* No Reaction. ^*b*^Yield of **2aa** and **3aa**, as determined by GC analysis of the mixture; the values in parentheses indicate the isolated yield.

### Substrate scope

**Fig. 2:**
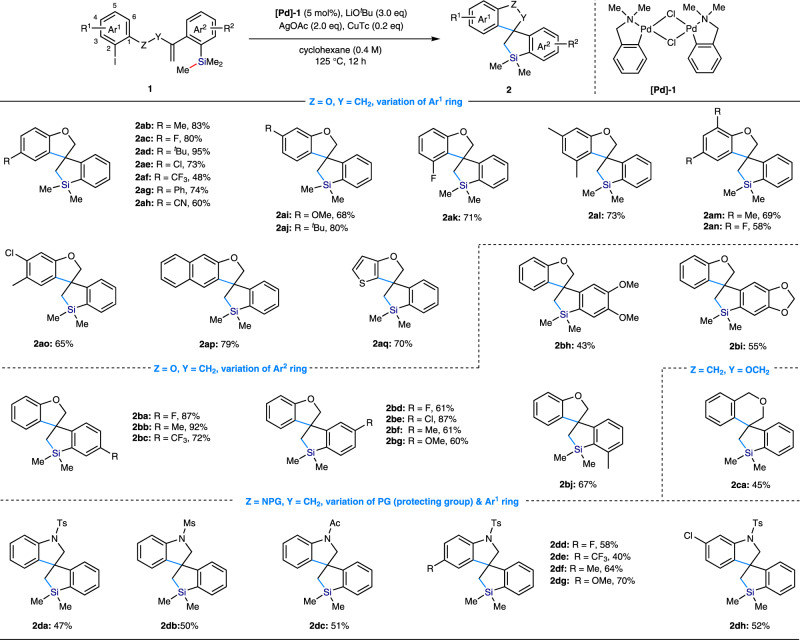
Substrate scope for the Pd-catalyzed sila-spirocyclization of 1 involving Si–C(sp3) bond cleavage/coupling. Reactions were carried out by using **[Pd]-1** (0.01 mmol), LiO^*t*^Bu (0.6 mmol), AgOAc (0.4 mmol), CuTc (0.04–0.2 mmol), and **1** (0.2 mmol) in Cyclohexane (0.4 M) or Toluene (0.4 M) for 4–12 h. Isolated yields are shown.

Under the optimized conditions, we next explored the substrate scope as summarized in Fig. 2. The scope of substituents R^1^ at the different positions of the Ar^1^ ring was first evaluated. A wide range of electron-withdrawing, electron neutral and electron-rich substituents at the C4-position were well-tolerated, thus giving moderate to good yields of the desired spirosilacycles (**2ab**−**ah**, 48−95% yield). The C5-substituted substrates also react well under the optimized conditions without significant loss of the yields (**2ai** & **2aj**, 68–80% yield). Installation of the different substituents at the C3-position, which would significantly increase the steric hindrance of the aryl iodide, did not affect the reactivity (**2ak** & **2al**, 71% & 73% yield, respectively). Sterically encumbered substrates bearing −F or −Me group at the C6-position also cyclized smoothly to produce the desired products **2am** & **2an** in 69 and 58% yields, respectively. The densely substituted aryl iodides are also effective substrates, which give rise to the products **2ak**−**ao** in 58–73% yields. The Ar^1^ ring could also be a napthalene and heteroarene in this spirosilacyclization reaction as evidenced by **2ap** & **2aq**. We then attempted to vary the R^2^-substituent on Ar^2^ ring bearing the TMS group of the substrate. We first proved that introducing a wide range of electron-withdrawing, electron neutral and electron-rich substituents into the meta- or para-position with respect to the TMS group did not significantly affect the cyclization efficiency, offering a broad range of substituted spirosilacycles (**2ba–bg**) in a 60−92% yield. The reactions with the substrates bearing two additional substituents on the Ar^2^ ring also proceeded well to give the corresponding products (**2bh**−**bi**). Installation of a methyl group at the adjacent position of TMS group did not impede the generation of the desired product **2bj**. Following the success of the preparation of 5,5-spirocycle, we further proved that this spirosilacyclization protocol is also capable of producing 5,6-spirocycle skeleton by employment of the substrate derived from (2-iodophenyl)methanol (**2ca**, 45% yield). It is worth mentioning that the spirocyclization reaction was completely hampered and 6-endo-cyclization was triggered if installation of an ortho-substituent with respect to the alkenyl group on the Ar^2^ ring. It might attribute to the increase of the steric hinerance around the alkene moiety. There is no impact on the reaction outcome if changing the tether atom from oxygen to nitrogen in substrate, delivering the corresponding spirosilacycles in moderate yields. A variety of protecting groups on the nitrogen, such as Ts, Ms, and Ac, were compatible with this transformation (**2da**−**dc**). The procedure was successfully expanded to the substrates bearing halogens and the other electron neutral, electron-donating as well as electron-withdrawing substituents at the 4- and 5-positions of Ar^1^ ring, furnished the corresponding spirosilacycles in good yields (**2dd**−**dh**, 40−70% yield).

**Fig. 3:**
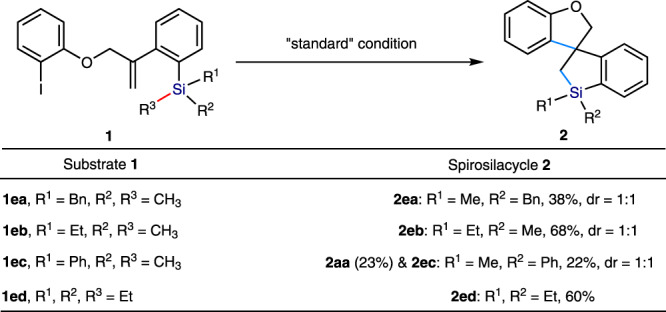
Investigation into the reactivity of the substituents on the silicon center. Reactions were carried out by using **[Pd]−1** (0.01 mmol), LiO^*t*^Bu (0.6 mmol), AgOAc (0.4 mmol), CuTc (0.04–0.2 mmol), and **1** (0.2 mmol) in Cyclohexane (0.4 M) or Toluene (0.4 M) for 4–12 h. Isolated yields are shown. The diastereomeric ratio (dr) of product **2** was determined by ^1^H NMR. Both of isomers were isolated by column.

Then, the effect of different substituents at the silicon center was studied under the optimized conditions (Fig. 3). In SiMe_2_Bn and SiMe_2_Et, the less sterically-hindered Si−Me bond was preferentially cleaved to give the corresponding spirosilacycles **2ea** (38% yield) and **2eb** (68% yield) with 1:1 dr, respectively. The low yield of **2ea** was attributed to the obvious increase of the steric hindrance of SiMe_2_Bn in contrast to the TMS group. The SiPhMe_2_ substituent delivered the mixture of **2aa** (23% yield) and **2ec** (22% yield), which originated from the competitive cleavage between C(sp^2^)−Si bond and C(sp^3^)−Si bond under the reaction. Moreover, we demonstrate that besides Si−Me bond, the other C(sp^3^)−Si bond could also be cleaved under the reaction as evidenced by the reaction of SiEt_3_ affording **2ed** in a 60% yield. The decrease of the yield again suggests a steric effect.

### Mechanistic studies

**Fig. 4:**
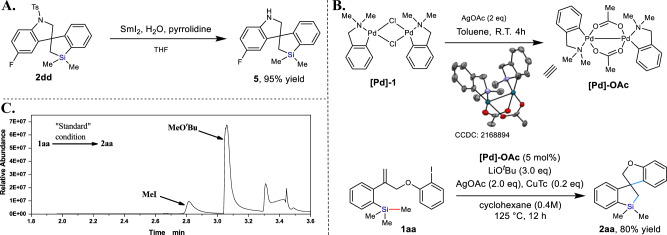
Removal of the protecting group and mechanistic investigation. **A** Removal of the protecting group (-Ts) in spirosilacycle **2dd**. **B** Synthesis, characterization, and application of **[Pd]-OAc** complex. **C** GC analysis of the gas composition of the model reaction.

Having established a structurally diverse library of spirosilacycles **2**, we first then proved that the protecting group (Ts) of **2dd** can be easily removed by treatment with SmI_2_ to yield the free amine **5** in 95% yield (Fig. 4A), therefore further increasing the synthetic utility of this cyclization strategy. Then, several control experiments were conducted to understand the reaction mechanism. Treatment of the **[Pd]−1** precatalyst with AgOAc (2.0 eq) in toluene at room temperature leads to the new Pd^II^-complex **[Pd]-OAc** (Fig. 4B), which has been clearly confirmed by single crystal X-ray crystallography. Furthermore, employment of 5 mol% **[Pd]-OAc** as the catalyst in the presence of LiO^*t*^Bu, the spirosilacyclization reaction of **1aa** works efficiently to deliver the desired product **2aa** in 80% yield (Fig. 4B). These results indicate that **[Pd]-OAc** complex may formed as the active catalyst during the reaction. In addition, we carried out the reaction of **1aa** in the presence of stoichiometric amount of **[Pd]-OAc**. A large amount of *N*,*N*-dimethyl-1-phenylmethanamine were detected by GC-MS during the reaction (See Supplementary Fig. 1). On the other hand, we attempted to trace the cleaved Si‒Me group by GC analysis of the gas composition of the model reaction under the standard condition. We found that the reaction generated considerable amount of MeO^*t*^Bu and MeI (Fig. 4C). Thus, we believe that I^-^/tBuO^-^ exchange happened under the catalytic cycle.

**Fig. 5:**
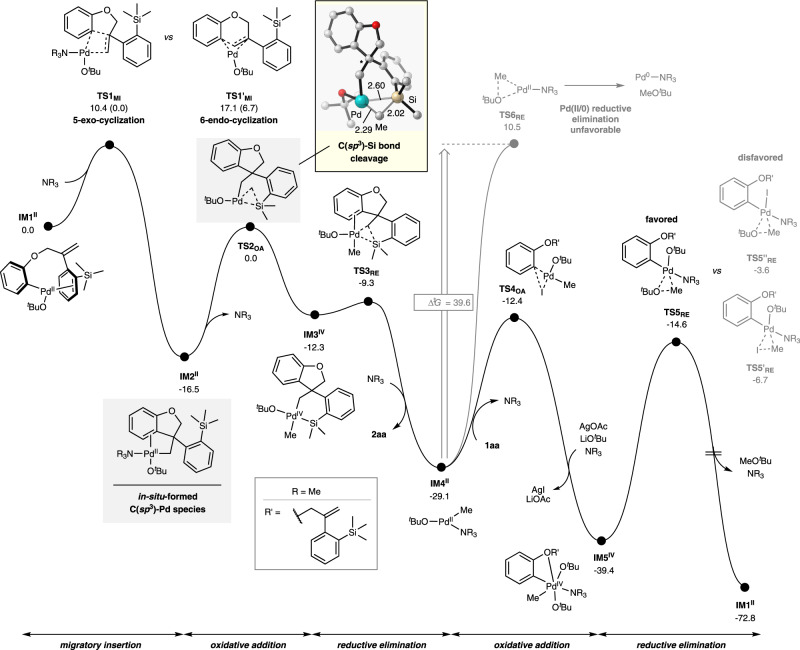
Free energy profile for proposed catalytic mechanism of this reaction. Free energies are shown in kcal/mol, distances in Å. All calculations were performed at the SMD-PBE0-D3/def2-TZVP//M06-L/def2-SV(P) level of theory in the Gaussian 16 software package.

Finally, quantum mechanical studies were carried out to gain further mechanistic insights into the elementary steps of the catalytic transformation, especially regarding the catalytic cycle of Pd catalyst, the mode of C(sp^3^)‒Si bond cleavage and the origin of the high selectivity of 5-exo-cyclization to form the desired C(sp^3^)-Pd species. The main results for the reaction of **1aa** to **2aa** are presented in Fig. 5. We postulate that a catalytic amount of Pd^0^ is formed in situ at the beginning of the reaction and undergoes oxidative addition with the aryl−I moiety of **1aa**, giving rise to a Pd^II^–aryl intermediate, which is in accordance with our experimental result (Table 1, entry 10), XPS analysis (See Supplementary Fig. 2) and has been well-documented in literature^53,54^. Based on evaluations of possible ligation modes, we found that the Pd^II^-aryl species **IM1**^**II**^ is most stabilized with the coordination of ^*t*^BuO^-^ (See Supplementary Figs. 3 and 4). The 5-exo-cyclization of **IM1**^**II**^ smoothly occurs via intramolecular migratory insertion (**TS1**_**MI**_), furnishing C(sp^3^)-Pd^II^ species **IM2**^**II**^ with a low barrier of only 10.4 kcal/mol. In contrast, the alternative 6-endo-cyclization (**TS1’**_**MI**_) is calculated to have a significantly larger barrier of 17.1 kcal/mol because of the high strain of the bridged ring structure in **TS1’**_**MI**_, resulting in a strong preference for the observed 5-exo pathway.

Then we focus on obtaining deep insight into the Si–C(sp^3^) bond cleavage and subsequent spirocyclization process. Despite numerous attempts with various initial geometries, we could not find a putative σ-methathesis transition state. The oxidative addition transition state **TS2**_**OA**_ was obtained instead in all convergend optimization jobs. It pointed out that a Pd(II)/Pd(IV) oxidative addition process of one of the three Si–C(sp^3^) bonds in **IM2**^**II**^ via the transition state **TS2**_**OA**_ for realizing the the Si–C(sp^3^) cleavage is favorable, giving rise to Pd^IV^-silametallacycle intermediate **IM3**^**IV**^ ^55–58^. Then, reductive elimination from **IM3**^**IV**^ along with the formation of new Si–C(sp^3^) bond occurs via the transition state **TS3**_**RE**_ to afford the desired spirosilacycle **2aa** and a Pd(II) intermediate **IM4**^**II**^. The hitherto largest barrier for this pathway is observed from **IM2**^**II**^ to **TS2**_**OA**_, which only demands 16.5 kcal/mol and is viewed as a facile reaction. We identify the consecutive oxidative addition/reductive-elimination process of the in-situ-generated C(sp^3^)-Pd^II^ species as a key mechanistic underpinning for the successful transformation.

The formed Pd(II) species **IM4**^**II**^ can either undergo further reductive elimination to regenerate the Pd(0) catalysts and complete the catalytic cycle or oxidative addition of the aryl−I moiety of **1aa** to form a Pd(IV)–aryl intermediate. Further DFT calculations were carried out to compare these two potential pathways. Reductive elimination from Pd(II) species **IM4**^**II**^ to Pd(0) species via the transition state **TS6**_**RE**_ is calculated to have a prohibitively high barrier of 39.6 kcal/mol. In contrast, oxidative addition of the active Pd(II) species **IM4**^**II**^ with aryl–I via the transition state **TS4**_**OA**_ to form the Pd(IV) intermediate **IM5**^**IV**^ is facile with a barrier of 16.7 kcal/mol and an immediate ligand exchange substituting I^–^ with tBuO^–^ in the presence of AgOAc and LiO^*t*^Bu. Then, a subsequent reductive elimination from **IM5**^**IV**^ takes place via the transition state **TS5**_**RE**_ with a feasible energetic barrier of 24.8 kcal/mol to yield the MeO^*t*^Bu as well as regenerate the active Pd(II) species **IM1**^**II**^ with the completion of the catalytic cycle. The reductive elimination favors Me–O^*t*^Bu generation over Ar–Me bond formation by a 1.2 kcal/mol kinetic difference, which explains the absence of Ar–Me byproduct (See Supplementary Fig. 5). Further analysis also shows that the I^–^-ligated **TS5’**_**RE**_ and **TS5’’R**_**E**_ are both higher in energy than the ^*t*^BuO^–^-coordinated **TS5**_**RE**_, supporting the stabilizing effect of ^*t*^BuO^–^. Overall, operation of the catalytic system on a Pd(II)/Pd(IV) manifold is more compatible with our computations and well accords with the experimental evidences.

In summary, we report the Pd-catalyzed spirosilacyclization, which proceeds via a Heck reaction/ Si−C(sp^3^) cleavage/ Si−C(sp^3^) bond formation sequence, allowing the construction of diverse spirosilacycles. From the mechanistic viewpoint, our study shows that Si–C(sp^3^) bond cleavage can be realized via the insertion of a M−C(sp^3^) species. This reactivity mode may open opportunities for the development of other reaction processes. DFT calculations reveals that 1) the reaction mechanism likely involves a Pd(II)/Pd(IV) catalytic cycle; 2) the Si−C(sp^3^) activation step proceeds via the intermediacy of a Pd(IV) species, which is generated by oxidative addition of σ-alkylpalladium(II) species to Si−C(sp^3^) bond.

## Methods

### General procedure

In a nitrogen-filled glovebox, an oven-dried 15 mL screw capped sealed tube was charged with a magnetic stir bar, **1** (0.20 mmol), **[Pd]-1** (5 mmol%), AgOAc (2 equiv), LiO^*t*^Bu (3 equiv), additive and cyclohexane (0.5 mL) or PhMe (0.5 mL). The tube was sealed, then removed from the glovebox, and the formed mixture was stirred at 125 °C under N_2_ for 12 h. After being cooled to room temperature, Saturated aqueous NH_4_Cl (5 mL) was added and the mixture was extracted with EA (3 × 5 mL). The combined organic phases were washed with water and brine, dried (MgSO_4_) and evaporated. The crude product was purified by preparative RP-HPLC on reversed phase column (C18(ODS)) (eluent: CH_3_CN) to afford the corresponding product.

## Supplementary information

Supplementary Information

Description of Additional Supplementary Files

Supplementary Data 1

## Data Availability

The authors declare that the data supporting the findings of this study are available within the article and its Supplementary Information Files as well as from the corresponding authors on request. Cartesian coordinates of the calculated structures are available from the Supplementary Data 1. The X-ray crystallographic coordinates for structures reported in this study have been deposited at the Cambridge Crystallographic Data Center (CCDC), under deposition numbers CCDC 2168893 (**2aa**) and CCDC 2168894 (**[Pd]-OAc**). These data can be obtained free of charge from The Cambridge Crystallographic Data Center via www.ccdc.cam.ac.uk/data_request/cif.
