# Author Correction: Sila-spirocyclization involving unstrained C(sp^3^)−Si bond cleavage

**DOI:** 10.1038/s41467-023-40944-0

**Published:** 2023-08-29

**Authors:** Yufeng Shi, Xiaonan Shi, Jinyu Zhang, Ying Qin, Bo Li, Dongbing Zhao

**Affiliations:** 1 State Key Laboratory and Institute of Elemento-Organic Chemistry, Haihe Laboratory of Sustainable Chemical Transformations, College of Chemistry, Nankai University, Tianjin, 300071 China; 2 Division of Chemistry and Chemical Engineering, California Institute of Technology, Pasadena, CA 91106 USA

**Keywords:** Synthetic chemistry methodology, Chemical synthesis, Homogeneous catalysis

Correction to: *Nature Communications* 10.1038/s41467-022-34466-4, published online 05 November 2022

In the original version of this article the ^1^H NMR spectra of compounds **1aa, 1ab, 1ac,1ad, 1ae, 1af, 1ag, 1ah, 1ai, 1aj, 1ak, 1al, 1am, 1an, 1ao, 1ap, 1aq, 1ba, 1bb, 1bc, 1bd, 1be, 1bf, 1bg, 1bh, 1bi, 1bj, 1ca, 1da, 1db, 1dc, 1dd, 1de, 1df, 1dg, 1dh, 1ea, 1eb, 1ec, 1ed, 2aa, 2ab, 2ac, 2ad, 2ae, 2af, 2ag, 2ah, 2ai, 2aj, 2ak, 2al, 2am, 2an, 2ao, 2ap, 2aq, 2ba, 2bb, 2bc, 2bd, 2be, 2bf, 2bg, 2bh, 2bi, 2bj, 2ca, 2da, 2db, 2dc, 2dd, 2de, 2df, 2dg, 2dh, 2ea** first and second diastereomer**, 2eb, 2ec, 2ed** and **5** in the supplementary information file were not calibrated using solvent peak or TMS as a standard. The spectra and the peak lists have been corrected in the PDF version of the supplementary information file. The missing calibrations of the ^1^H NMR spectra did not affect the results of this study.

### Supplementary information

Updated Supplementary Information

