# Supplementary material for: Sila-spirocyclization involving unstrained C(sp3)−Si bond cleavage
Source: Nat Commun. 2022 Nov 5;13:6697. doi: 10.1038/s41467-022-34466-4 (PMC9637223; doi:10.1038/s41467-022-34466-4)
Supplement: Supplementary file 1 — Supplementary Information [file 41467_2022_34466_MOESM1_ESM.pdf]

Supplementary Information

Sila-spirocyclization Involving Unstrained C(sp^3)−Si Bond Cleavage

Yufeng Shi,[1] Xiaonan Shi,[1] Jinyu Zhang,[1] Ying Qin,[1] Bo Li,[2]* and Dongbing Zhao[1]*

[1]State Key Laboratory and Institute of Elemento-Organic Chemistry, College of Chemistry, Nankai University, Tianjin 300071, People's Republic of China

[2]Division of Chemistry and Chemical Engineering, California Institute of Technology, Pasadena, CA 91106 (USA)

* Correspondence: bli3@caltech.edu (B. L.) & dongbing.chem@nankai.edu.cn (D. Z.)

Table of Content

1. Supplementary Notes 2

2. Supplementary Methods 3

    Synthesis of Starting Materials 3

    Characterization of Products 1 7

Pd-Catalyzed Spirocyclization Reaction of 1..27

    Condition Screening...27

    General procedure..28

    Characterization of Products 2 28

Removal of the Protecting Group (-Ts) in Spirosilacycle 2dd 55

    General Procedure..55

    Characterization of Product 5...56

3. Supplementary Discussion..56

Experimental Mechanistic Study 56

Computational Studies...59

    Quantum Mechanical Studies 59

    Energies and Coordinates of Calculated Structures 60

4. Supplementary Figures 62

    NMR Spectra 62

5. Supplementary References...163

1. Supplementary Notes

Unless otherwise noted, all reactions were set up on a Schlenk vacuum line or in a glovebox using oven-dried glassware and were stirred with Teflon-coated magnetic stirring bars under a N_2 atmosphere with dry solvents. Et_2O and toluene were distilled with Na before using. Other dry solvents or commercially available chemicals were obtained from Adamas-beta, Alfa Aesar, *J&K*, Sigma-Aldrich, Energy Chemical, Bide Pharmatech, Sinocompound, TCI, Sinocompound and used as received unless otherwise stated. Analytical thin layer chromatography (TLC) was performed on SANPONT SGF254 glass plates. TLC plates were visualized by exposure to short wave ultraviolet light (254 nm, 365 nm) and/or iodine. Column chromatography was performed using GENERAL-REAGENT silica gel (200-300 mesh).

Nuclear magnetic resonance (NMR) spectra were recorded on Bruker AV 400 spectrometer at 400 MHz for ^{1}H NMR, 100 MHz for ^{13}C NMR, 376 MHz for ^{19}F NMR using $CDCl_3$ as solvent. ^{1}H and ^{13}C NMR are reported (ppm) relative to the $CDCl_3$ peak (δ_H = 7.26 ppm, δ_C = 77.16 ppm). All coupling constants (*J* values) were reported in Hertz (Hz). Multiplicities are reported as follows: singlet (s), doublet (d), triplet (t), quartet (q), doublet of doublets (dd), doublet of triplets (dt), triplet of doublets (td) and multiplet (m).

GC-MS analysis were performed on a GC-MS with an EI mode (Thermo Scientific Trace 300/GC-System and ISQ/QD). High-resolution mass spectra (HRMS) were performed on a Q Exactive GC-Orbitrap MS (EI). The residues of the catalytic reactions were purified on C18(ODS) column (5μm, 21.2x250 mm) with CH_3CN by preparative RP-HPLC with an Bonna-Agela CHEETAH HP series.

2. Supplementary Methods

Synthesis of Starting Materials

Procedure A:

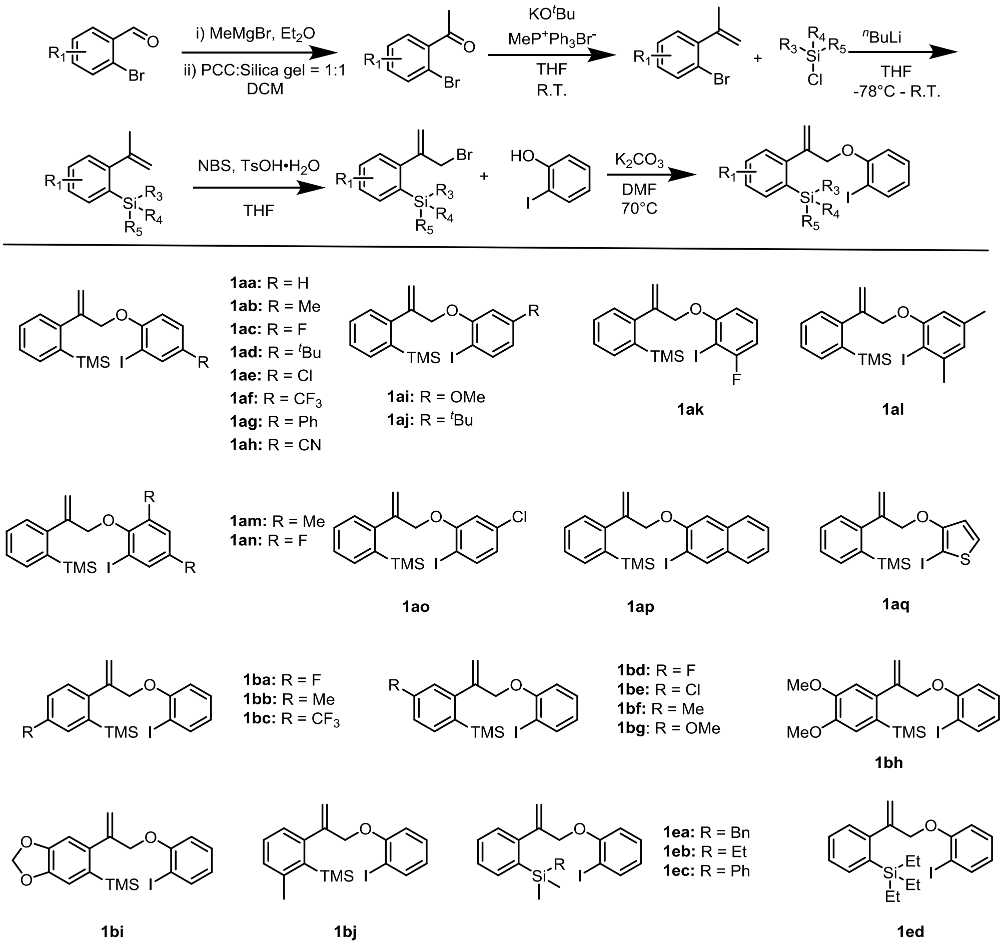

Step I: 1-(2-Bromophenyl) ethan-1-one derivatives were prepared following a previously reported procedure[1]. To a solution of 2-bromobenzaldehyde derivatives (commercially available chemicals; 20 mmol) in THF (20 mL) was added MeMgBr (commercially available chemicals; 24 mmol) dropwise at 0 °C under N_2, and stirred at the same temperature. Upon completion, the reaction was quenched with saturated NH_4Cl solution and extracted with EtOAc. The combined organic layers were washed with brine, dried over anhydrous Na_2SO_4, filtered, and concentrated to deliver the corresponding secondary benzylic alcohols, which were directly used for the next

without further purification. To a solution of 1-(2-bromophenyl) ethan-1-ol derivatives (15 mmol) in DCM was added a mixture of PCC (45.0 mmol) and silica gel [PCC/silica gel = 1/1 (w/w)]. The resulted reaction mixture was stirred at room temperature. Upon completion, the reaction mixture was then filtered through a pad of silica gel with EtOAc as eluent. The resulting solution was concentrated, and the residue was purified by silica gel chromatography column to yield the desired 1-(2-bromophenyl) ethan-1-one derivatives.

Step II: Following a modified literature procedure[2], to the mixture of methyl triphenylphosphonium bromide (40 mmol, 2 equiv.) and ^{t}BuOK (50.0 mmol, 2.5 equiv.) was added anhydrous THF (50.0 mL) and stirred at rt. for 20 min. under argon, then the solution of ketone (20 mmol, 1.0 equiv.) in THF (50.0 mL) was added slowly, the resulting solution was allowed to stir at rt. overnight. To the mixture was added sat. NH_4Cl, then THF was removed and the mixture was extracted with EtOAc (3 x 30 mL), the combined EtOAc solution was washed with brine, dried over anhydrous Na_2SO_4. After removing the solvent, the residue was purified by flash chromatography on silica gel to yield 1-bromo-2-(prop-1-en-2-yl) benzene derivatives.

Step III: Following a modified literature procedure[3], a solution of ^{n}BuLi in hexane (11 mmol, 1.1 equiv., 2.5 M in hexane) was added dropwise to a solution of 1-bromo-2-(prop-1-en-2-yl) benzene derivatives (10 mmol, 1.0 equiv.) in anhydrous THF (20 mL) at -78 °C. After the reaction mixture was stirred at this temperature for 0.5 hour, chlorosilane (12 mmol, 1.2 equiv.) was added via a syringe. Then, the mixture was warmed to room temperature. The solvent was removed in vacuo, and evaporated under reduced pressure to give trimethyl(2-(prop-1-en-2-yl) phenyl) silane derivatives, which was used for the next step without purification.

Step IV: Following a modified literature procedure[2], in an oven dried flask trimethyl (2-(prop-1-en-2-yl) phenyl) silane derivatives (8 mmol, 1.0 equiv.) was dissolved in anhydrous THF (3.0 mL/mmol). To the resulting solution *N*-Bromosuccinimide (NBS, 8.4mmol, 1.05 equiv.) and TsOH (0.8 mmol, 0.1 equiv.) was added and the solution was

refluxed at 80 °C for 4 h. Reaction mixture was cooled to rt and the reaction mixture was taken in petroleum ether (15 mL/mmol), washed with H_2O (15 mL × 3). Organic phase was dried over anh. Na_2SO_4, concentrated under reduced pressure to afford the corresponding compounds.

Step V: Following a modified literature procedure [4], to a solution of 2-iodophenol (5 mmol, 1.0 equiv.) and K_2CO_3 (7.5 mmol, 1.5 equiv.) in DMF (10 mL) was added allyl halide (6 mmol, 1.2 equiv). The reaction mixture was allowed to warm at 70 °C and stirred overnight. The reaction was quenched with water (10 mL), and extracted with ethyl acetate (3 ×10 mL), wash with brine, dried over anhydrous Na_2SO_4 and concentrated under reduced pressure. The residue was purified by flash chromatography on silica gel to give the products **1**.

Procedure B:

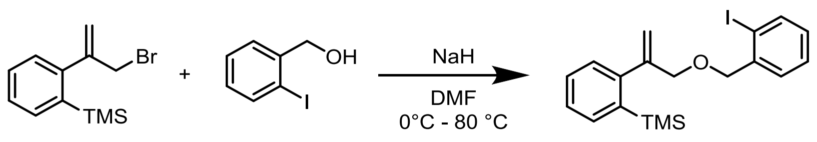

(2-(3-Bromoprop-1-en-2-yl) phenyl) trimethylsilane was prepared following **Procedure A**.

Following a modified literature procedure [5], to a suspension of NaH (60% in oil, 6 mmol, 1.2 equiv.) in DMF (10 mL) were added (2-iodophenyl) methanol (5 mmol, 1.0 equiv.) in DMF (5 mL) at 0 °C under Ar atmosphere. When the evolution of H_2 has ceased, (2-(3-bromoprop-1-en-2-yl) phenyl) trimethylsilane (5.5 mmol, 1.0 equiv.) was added. The reaction mixture was heated to 80 °C overnight. The reaction mixture was quenched with water and extracted with Et_2O. The organic phase was dried over $MgSO_4$ and concentrated in vacuo. The residue was purified by column chromatography (PE: EA = 40:1) to afford compound **1ca.**

Procedure C:

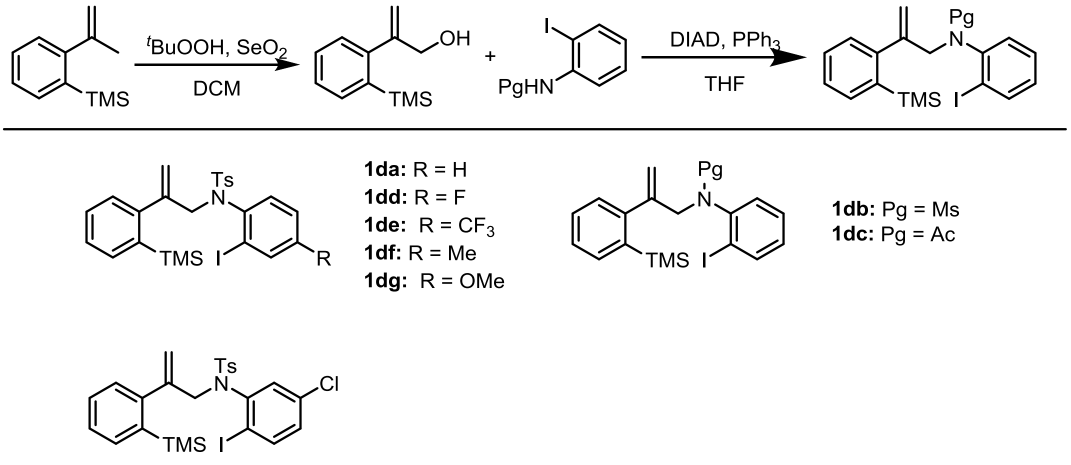

1dh

Trimethyl(2-(prop-1-en-2-yl) phenyl) silane was prepared following **Procedure A.**

Step I: Following a modified literature procedure [6], to a stirred suspension of SeO_2 (15 mmol, 1.5 equiv.) in CH_2Cl_2 (10 mL) at R.T. was added a solution of tert-butyl hydroperoxide (70% in water, 15 mmol, 1.5 equiv.). After 10 min a solution of trimethyl(2-(prop-1-en-2-yl) phenyl) silane (10.0 mmol, 1.0 equiv.) in CH_2Cl_2 (5 mL) was added and the mixture stirred at ambient temperature for 4 h. Saturated aqueous $NaHCO_3$ (10 mL) was added and the mixture was extracted with CH_2Cl_2 (3 × 10 mL). The combined organic phases were washed with water and brine, dried ($MgSO_4$) and evaporated. Flash chromatography (PE: EA = 5:1) afforded 2-(2-(trimethylsilyl) phenyl) prop-2-en-1-ol as a clear colorless oil (yield = 50%).

Step II: Following a modified literature procedure [7], to a flame-dried flask were added PPh_3 (15 mmol, 1.5 equiv.), aryl iodide (10.05 mmol, 1.05 equiv.), allylic alcohol (10 mmol, 1.0 equiv.) and anhydrous THF (40 mL) sequentially under Ar atmosphere at room temperature. A solution of DIAD (15 mmol, 1.5 equiv.) in 10 mL of anhydrous THF was added dropwise via addition funnel at 0 °C. The resulting mixture was allowed to warm to room temperature naturally with stirring. After the reaction was complete as monitored by TLC, the reaction mixture was concentrated on a rotary evaporator and the residue was purified by chromatography on silica gel to afford the desired product

Characterization of Products 1

(2-(3-(2-Iodophenoxy)prop-1-en-2-yl)phenyl)trimethylsilane (1aa)

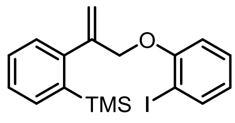

1aa

Following the **procedure A**, **1aa** was obtained as a colorless oil (R_f = 0.3, petroleum ether) in 50% yield (2 g, 5 mmol). **^{1}H NMR (400 MHz, $CDCl_3$)** δ 7.80 (m, 1H), 7.58 (m, 1H), 7.39 – 7.28 (m, 2H), 7.28 – 7.24 (m, 2H), 6.79 (m, 1H), 6.72 (m, 1H), 5.88 (q, *J* = 1.8 Hz, 1H), 5.24 (d, *J* = 1.8 Hz, 1H), 4.67 (d, *J* = 1.8 Hz, 2H), 0.30 (s, 9H). **^{13}C NMR (101 MHz, $CDCl_3$)** δ 156.13, 145.02, 144.66, 138.65, 137.73, 134.17, 128.52, 127.73, 125.98, 121.82, 115.19, 111.24, 85.67, 70.92, 52.55, -0.00. **MS (EI)** calcd. for $C_{18}H_{21}IOSi$ $[M]^+$: 408.04. Found: 408.15.

(2-(3-(2-Iodo-4-methylphenoxy)prop-1-en-2-yl)phenyl)trimethylsilane (1ab)

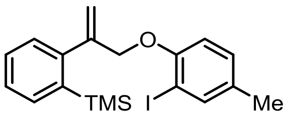

1ab

Following the **procedure A**, **1ab** was obtained as a colorless oil (R_f = 0.3, petroleum ether) in 57% yield (2.4 g, 5.7 mmol). **^{1}H NMR (400 MHz, $CDCl_3$)** δ 7.63 (m, 1H), 7.61 – 7.56 (m, 1H), 7.41 – 7.28 (m, 2H), 7.27 – 7.23 (m, 1H), 7.07 (m, 1H), 6.68 (m, 1H), 5.86 (d, *J* = 1.8 Hz, 1H), 5.22 (q, *J* = 1.8 Hz, 1H), 4.63 (t, *J* = 1.8 Hz, 2H), 2.26 (s, 3H), 0.30 (s, 9H). **^{13}C NMR (101 MHz, $CDCl_3$)** δ 154.18, 145.16, 144.84, 139.03, 137.82, 134.22, 131.42, 129.00, 127.77, 127.70, 125.99, 115.08, 111.05, 85.49, 71.09, 19.16, -0.00. **MS (EI)** calcd. for $C_{19}H_{23}IOSi$ $[M]^+$: 422.06. Found: 422.15.

(2-(3-(4-Fluoro-2-iodophenoxy)prop-1-en-2-yl)phenyl)trimethylsilane (1ac)

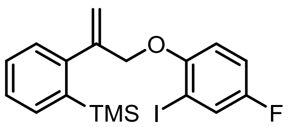

1ac

Following the **procedure A**, **1ac** was obtained as a white solid (R_f = 0.25, petroleum ether) in 42% yield (1.8 g, 4.2 mmol). **^{1}H NMR (400 MHz, $CDCl_3$)** δ 7.64 (m, 1H), 7.56 (m, 1H), 7.40 – 7.36 (m, 2H), 7.30 – 7.28 (m, 1H), 7.09 – 6.98 (m, 1H), 6.76 (m, 1H), 5.89 (p, *J* = 1.5 Hz, 1H), 5.28 (t, *J* = 1.5 Hz, 1H), 4.67 (t, *J* = 1.5 Hz, 2H), 0.35 (s, 9H).**^{13}C NMR (101 MHz, $CDCl_3$)** δ 156.00 (d, *J* = 243.7 Hz), 152.92 (d, *J* = 2.4 Hz), 144.92, 144.63, 137.76, 134.23, 127.71, 126.04, 125.48, 125.23, 115.27, 114.68 (d, *J* = 22.8 Hz), 111.39 (d, *J* = 8.1 Hz), 85.08 (d, *J* = 8.7 Hz), 71.73, -0.00. **^{19}F NMR (376 MHz, $CDCl_3$)** δ -121.76. **MS (EI)** calcd. for $C_{18}H_{20}FIOSi$ $[M]^+$: 426.03. Found: 426.13.

(2-(3-(4-(tert-Butyl)-2-iodophenoxy)prop-1-en-2-yl)phenyl)trimethylsilane (1ad)

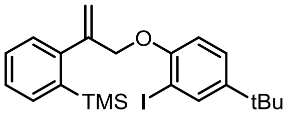

1ad

Following the **procedure A**, **1ad** was obtained as a colorless oil (R_f = 0.3, petroleum ether) in 44% yield (2.0 g, 4.4 mmol). **^{1}H NMR (400 MHz, $CDCl_3$)** δ 7.79 (m, 1H), 7.59 (m, 1H), 7.42 – 7.22 (m, 4H), 6.72 (m, 1H), 5.86 (s, 1H), 5.22 (s, 1H), 4.65 (m, 2H), 1.28 (s, 9H), 0.30 (s, 9H). **^{13}C NMR (101 MHz, $CDCl_3$)** δ 154.01, 145.17, 144.90, 144.84, 137.80, 135.77, 134.19, 127.75, 127.67, 125.96, 125.37, 115.08, 110.71, 85.57, 71.03, 33.18, 30.55, 0.00. **MS (EI)** calcd. for $C_{22}H_{29}IOSi$ $[M]^+$: 464.10. Found: 464.25.

(2-(3-(4-Chloro-2-iodophenoxy)prop-1-en-2-yl)phenyl)trimethylsilane (1ae)

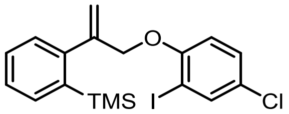

1ae

Following the **procedure A**, **1ae** was obtained as a colorless oil (R_f = 0.3, petroleum ether) in 40% yield (1.77 g, 4.0 mmol). **^{1}H NMR (400 MHz, $CDCl_3$)** δ 7.77 (m, 1H), 7.59 (m, 1H), 7.40 – 7.28 (m, 2H), 7.27 – 7.22 (m, 2H), 6.70 (m, 1H), 5.83 (q, *J* = 1.8 Hz, 1H), 5.24 (d, *J* = 1.8 Hz, 1H), 4.64 (d, *J* = 1.8 Hz, 2H), 0.29 (s, 9H). **^{13}C NMR (101 MHz, $CDCl_3$)** δ 155.19, 144.86, 144.45, 137.88, 137.83, 134.29, 134.29, 128.33, 127.76, 126.12, 125.80, 115.43, 111.72, 85.84, 71.43, 0.00. **MS (EI)** calcd. for $C_{18}H_{20}ClIOSi$ $[M]^+$: 442.00. Found: 441.95

(2-(3-(2-Iodo-4-(trifluoromethyl)phenoxy)prop-1-en-2-yl)phenyl)trimethylsilane (1af)

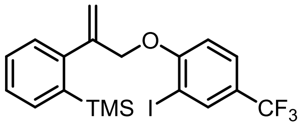

1af

Following the **procedure A**, **1af** was obtained as a yellow liquid (R_f = 0.3, petroleum ether) in 46% yield (2.2 g, 4.6 mmol). **^{1}H NMR (400 MHz, $CDCl_3$)** δ 8.05 (m, 1H), 7.58 (m, 2H), 7.35 (m, 2H), 7.25 – 7.16 (m, 1H), 6.83 (m, 1H), 5.85 (q, *J* = 1.8 Hz, 1H), 5.27 (d, *J* = 1.8 Hz, 1H), 4.72 (t, *J* = 1.8 Hz, 2H), 0.30 (s, 9H). **^{13}C NMR (101 MHz, $CDCl_3$)** δ 158.73, 144.66, 144.13, 137.85, 135.86 (q, *J* = 3.7 Hz), 134.35, 127.81, 127.78, 126.22, 126.09 (q, *J* = 3.7 Hz), 123.76 (q, *J* = 30.4 Hz), 122.42 (q, *J* = 269.3 Hz), 115.66, 110.54, 85.31, 71.30, -0.00.**^{19}F NMR (376 MHz, $CDCl_3$)** δ -61.62. **MS (EI)** calcd. for $C_{19}H_{20}F_3IOSi$ $[M]^+$: 476.03. Found: 476.35.

(2-(3-((3-Iodo-[1,1'-biphenyl]-4-yl)oxy)prop-1-en-2-yl)phenyl)trimethylsilane (1ag)

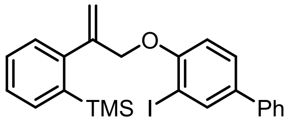

1ag

Following the **procedure B**, **1ag** was obtained as a colorless oil (R_f = 0.3, petroleum ether) in 42% yield (2.0 g, 4.2 mmol). **^{1}H NMR (400 MHz, $CDCl_3$)** δ 8.06 (m, 1H), 7.65 – 7.59 (m, 1H), 7.53 (m, 3H), 7.43 (s, 2H), 7.40 – 7.27 (m, 4H), 6.87 (m, 1H), 5.91 (q, *J* = 1.8 Hz, 1H), 5.27 (q, *J* = 1.8 Hz, 1H), 4.73 (t, *J* = 1.8 Hz, 2H), 0.33 (s, 9H). **^{13}C NMR (101 MHz, $CDCl_3$)** δ 155.60, 144.96, 144.59, 138.34, 137.74, 137.15, 135.05, 134.19, 127.91, 127.72, 127.69, 127.12, 126.27, 126.01, 125.82, 115.23, 111.20, 86.09, 71.07, 0.00. **MS (EI)** calcd. for $C_{24}H_{25}IOSi$ $[M]^+$: 484.07. Found: 483.87.

3-Iodo-4-((2-(2-(trimethylsilyl)phenyl)allyl)oxy)benzonitrile (1ah)

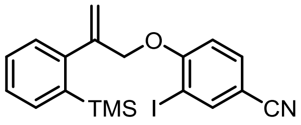

1ah

Following the **procedure A**, **1ah** was obtained as a colorless oil (R_f = 0.3, petroleum ether) in 52% yield (2.3 g, 5.2 mmol). **^{1}H NMR (400 MHz, $CDCl_3$)** δ 8.08 (m, 1H), 7.61 (m, 2H), 7.35 (m, 2H), 7.24 (m, 1H), 6.82 (m, 1H), 5.82 (q, *J* = 1.7 Hz, 1H), 5.28 (q, *J* = 1.7 Hz, 1H), 4.73 (d, *J* = 1.7 Hz, 2H), 0.30 (s, 9H). **^{13}C NMR (101 MHz, $CDCl_3$)** δ 159.65, 144.41, 143.78, 142.07, 137.84, 134.39, 133.14, 127.84, 127.76, 126.29, 116.68, 115.94, 110.94, 105.33, 85.64, 71.43, 0.00. **MS (EI)** calcd. for $C_{19}H_{20}INOSi$ $[M]^+$: 433.04. Found: 433.03.

1-(2-(1-(4-Ethylphenyl)vinyl)phenyl)-1-methylsiletane (1ai)

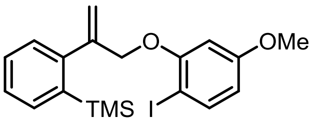

1ai

Following the **procedure A**, **1ai** was obtained as a colorless oil (R_f = 0.3, petroleum ether) in 42% yield (1.8 g, 4.2 mmol). **^{1}H NMR (400 MHz, $CDCl_3$)** δ 7.65 (m, 1H), 7.62 – 7.56 (m, 1H), 7.34 (m, 2H), 7.27 (m, 1H), 6.40 (s, 1H), 6.34 (m, 1H), 5.87 (d, *J* = 1.8 Hz, 1H), 5.24 (d, *J* = 1.8 Hz, 1H), 4.64 (t, *J* = 1.8 Hz, 2H), 3.77 (s, 3H), 0.30 (s, 9H). **^{13}C NMR (101 MHz, $CDCl_3$)** δ 160.44, 156.99, 145.07, 144.60, 138.39, 137.86, 134.25, 127.82, 127.73, 126.05, 115.31, 106.70, 99.35, 74.41, 70.96, 54.68, 0.00. **MS (EI)** calcd. for $C_{19}H_{23}IO_2Si$ $[M]^+$: 438.05. Found: 438.10.

(2-(3-(5-(tert-Butyl)-2-iodophenoxy)prop-1-en-2-yl)phenyl)trimethylsilane (1aj)

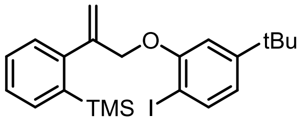

1aj

Following the **procedure A**, **1aj** was obtained as a colorless oil (R_f = 0.3, petroleum ether) in 56% yield (2.6 g, 5.6 mmol). **^{1}H NMR (400 MHz, $CDCl_3$)** δ 7.70 (m, 1H), 7.61 (m, 1H), 7.42 – 7.29 (m, 2H), 7.27 (m, 1H), 6.82 (m, 1H), 6.78 (m, 1H), 5.91 (d, *J* = 1.9 Hz, 1H), 5.24 (d, *J* = 1.9 Hz, 1H), 4.69 (t, *J* = 1.9 Hz, 2H), 1.29 (s, 9H), 0.32 (s, 9H). **^{13}C NMR (101 MHz, $CDCl_3$)** δ 155.94, 152.53, 145.20, 144.82, 137.93, 137.89, 134.23, 127.82, 127.73, 126.02, 119.24, 109.09, 115.06, 81.96, 70.86, 34.03, 30.39, 0.00. **MS (EI)** calcd. for $C_{22}H_{29}IOSi$ $[M]^+$: 464.10. Found: 464.19.

(2-(3-(3-Fluoro-2-iodophenoxy)prop-1-en-2-yl)phenyl)trimethylsilane (1ak)

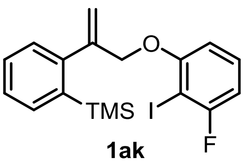

1ak

Following the **procedure A**, **1ak** was obtained as a colorless oil (R_f = 0.3, petroleum ether) in 46% yield (2.0 g, 4.6mmol). **^{1}H NMR (400 MHz, CDCl$_3$)** δ 7.60 (m, 2H), 7.34 (m, 2H), 7.19 (m, 1H), 6.73 (m, 1H), 6.59 (m, 1H), 5.59 (q, J = 1.7 Hz, 1H), 5.19 (q, J = 1.7 Hz, 1H), 4.63 (t, J = 1.7 Hz, 2H), 0.29 (s, 9H). **^{13}C NMR (101 MHz, CDCl$_3$)** δ 163.01 (d, J = 247.1 Hz), 157.17, 144.74, 144.19, 138.76 (d, J = 9.9 Hz), 137.81, 134.29, 127.77, 126.13, 115.56, 108.71 (d, J = 21.8 Hz), 100.03, 99.77, 78.46, 71.20, -0.00. **^{19}F NMR (376 MHz, CDCl$_3$)** δ -92.06. **MS (EI)** calcd. for $C_{18}H_{20}FIOSi$ $[M]^+$: 426.03. Found: 426.25.

(2-(3-(2-Iodo-3,5-dimethylphenoxy)prop-1-en-2-yl)phenyl)trimethylsilane (1al)

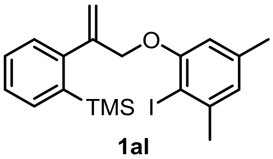

1al

Following the **procedure A**, **1al** was obtained as a colorless liquid (R_f = 0.3, petroleum ether) in 55% yield (2.4 g, 5.5 mmol). **^{1}H NMR (400 MHz, CDCl$_3$)** δ 7.60 (m, 1H), 7.39 – 7.29 (m, 2H), 7.28 – 7.24 (m, 1H), 6.74 (d, J = 1.8 Hz, 1H), 6.43 (d, J = 1.8 Hz, 1H), 5.94 (t, J = 1.8 Hz, 1H), 5.24 (q, J = 1.8 Hz, 1H), 4.65 (t, J = 1.8 Hz, 2H), 2.45 (s, 3H), 2.27 (s, 3H), 0.30 (s, 9H). **^{13}C NMR (101 MHz, CDCl$_3$)** δ 156.06, 145.23, 144.87, 142.15, 137.95, 137.83, 134.20, 127.75, 127.68, 125.96, 122.68, 115.01, 109.40, 88.58, 71.06, 27.68, 20.36, 0.00. **MS (EI)** calcd. for $C_{20}H_{25}IOSi$ $[M]^+$: 436.07. Found: 436.00.

(2-(3-(2-Iodo-4,6-dimethylphenoxy)prop-1-en-2-yl)phenyl)trimethylsilane (1am)

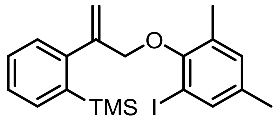

1am

Following the **procedure A**, **1am** was obtained as a pale yellow liquid (R_f = 0.6, petroleum ether) in 49% yield (2.2 g, 4.9mmol). **^{1}H NMR (400 MHz, CDCl$_3$)** δ 7.62 – 7.56 (m, 1H), 7.49 – 7.42 (m, 1H), 7.37 – 7.26 (m, 2H), 7.27 – 7.21 (m, 1H), 6.99 – 6.91 (m, 1H), 5.84 (d, *J* = 1.8 Hz, 1H), 5.22 (q, *J* = 1.8 Hz, 1H), 4.49 (t, *J* = 1.8 Hz, 2H), 2.26 (s, 3H), 2.23 (s, 3H), 0.33 (s, 9H). **^{13}C NMR (101 MHz, CDCl$_3$)** δ 153.37, 145.41, 145.24, 137.36, 136.29, 134.55, 133.84, 131.17, 130.69, 127.49, 127.24, 125.50, 114.50, 90.57, 73.73, 19.06, 15.84, 0.00. **MS (EI)** calcd. for $C_{20}H_{25}IOSi$ $[M]^+$: 436.07 Found: 436.09.

(2-(3-(2,4-Difluoro-6-iodophenoxy)prop-1-en-2-yl)phenyl)trimethylsilane (1an)

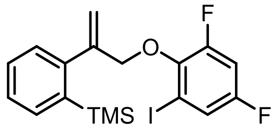

1an

Following the **procedure A**, **1an** was obtained as a colorless oil (R_f = 0.4, petroleum ether) in 57% yield (2.5 g, 5.7 mmol). **^{1}H NMR (400 MHz, CDCl$_3$)** δ 7.60 – 7.55 (m, 1H), 7.34 – 7.27 (m, 3H), 7.23 (m, 1H), 6.88 (m, 1H), 5.84 (q, *J* = 1.8 Hz, 1H), 5.23 (q, *J* = 1.8 Hz, 1H), 4.68 (q, *J* = 1.8 Hz, 2H), 0.32 (s, 9H). **^{13}C NMR (101 MHz, CDCl$_3$)** δ 158.41 (dd, *J* = 249.3, 11.7 Hz), 153.16 (dd, *J* = 254.3, 12.7 Hz), 145.11, 144.90, 141.57 (dd, *J* = 12.8, 4.2 Hz), 137.65, 134.07, 127.65, 127.49, 125.80, 120.20 (dd, *J* = 24.6, 3.8 Hz), 115.11, 104.70 (dd, *J* = 26.7, 23.4 Hz), 90.26 (dd, *J* = 10.2, 2.6 Hz), 75.29, 0.00. **^{19}F NMR (376 MHz, CDCl$_3$)** δ -115.36, -122.12. **MS (EI)** calcd. for $C_{18}H_{19}F_2IOSi$ $[M]^+$: 444.02. Found: 444.09.

(2-(3-(5-Chloro-2-iodo-4-methylphenoxy)prop-1-en-2-yl)phenyl)trimethylsilane (1ao)

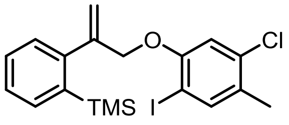

1ao

Following the **procedure A**, **1ao** was obtained as a yellow liquid (R_f = 0.3, petroleum ether) in 50% yield (2.3 g, 5.0 mmol). **^{1}H NMR (400 MHz, CDCl$_3$)** δ 7.65 (m, 1H), 7.62 – 7.52 (m, 1H), 7.34 (m, 2H), 7.26 – 7.22 (m, 1H), 6.78 (s, 1H), 5.83 (d, *J* = 1.8 Hz, 1H), 5.24 (d, *J* = 1.8 Hz, 1H), 4.62 (t, *J* = 1.8 Hz, 2H), 2.27 (s, 3H), 0.30 (s, 9H). **^{13}C NMR (101 MHz, CDCl$_3$)** δ 154.94, 144.84, 144.43, 139.74, 137.80, 134.26, 133.97, 129.40, 127.74, 126.08, 115.31, 112.20, 107.85, 82.85, 71.33, 17.93, 0.00. **MS (EI)** calcd. for $C_{19}H_{22}ClIOSi$ [M]$^+$: 456.02. Found: 456.13

(2-(3-((3-Iodonaphthalen-2-yl)oxy)prop-1-en-2-yl)phenyl)trimethylsilane (1ap)

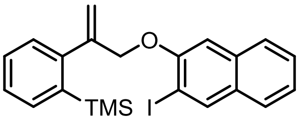

1ap

Following the **procedure A**, **1ao** was obtained as a colorless oil (R_f = 0.3, petroleum ether) in 38% yield (1.7 g, 3.8 mmol). **^{1}H NMR (400 MHz, CDCl$_3$)** δ 8.38 (s, 1H), 7.73 – 7.63 (m, 3H), 7.49 – 7.43 (m, 1H), 7.43 – 7.33 (m, 4H), 7.08 (s, 1H), 6.01 (q, *J* = 1.8 Hz, 1H), 5.32 (q, *J* = 1.8 Hz, 1H), 4.82 (t, *J* = 1.8 Hz, 2H), 0.36 (s, 9H). **^{13}C NMR (101 MHz, CDCl$_3$)** δ 153.08, 145.08, 144.59, 138.40, 137.84, 134.24, 133.25, 129.53, 127.81, 127.73, 126.06, 126.03, 125.76, 125.71, 123.50, 115.21, 105.73, 87.59, 70.97, 0.00. **MS (EI)** calcd. for $C_{22}H_{23}IOSiL$ [M]$^+$: 458.06. Found: 458.03.

(2-(3-((2-Iodothiophen-3-yl)oxy)prop-1-en-2-yl)phenyl)trimethylsilane (1aq)

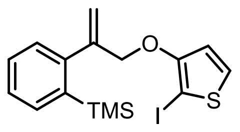

1aq

Following the **procedure A**, **1aq** was obtained as a colorless oil (R_f = 0.5, petroleum ether) in 39% yield (1.6 g, 3.9 mmol). **^{1}H NMR (400 MHz, CDCl$_3$)** δ 7.65 – 7.56 (m, 1H), 7.43 (m, 1H), 7.39 – 7.28 (m, 2H), 7.24 (m, 1H), 6.68 (m, 1H), 5.74 (q, *J* = 1.8 Hz, 1H), 5.22 (q, *J* = 1.8 Hz, 1H), 4.71 (t, *J* = 1.8 Hz, 2H), 0.33 (s, 9H). **^{13}C NMR (101 MHz, CDCl$_3$)** δ 157.72, 145.13, 144.93, 137.66, 134.15, 129.05, 127.63, 127.61, 125.91, 115.77, 115.00, 73.71, 54.11, 0.00. **MS (EI)** calcd. for $C_{16}H_{19}IOSSi$ $[M]^+$: 414.00. Found: 414.15.

(5-Fluoro-2-(3-(2-iodophenoxy)prop-1-en-2-yl)phenyl)trimethylsilane (1ba)

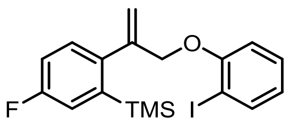

1ba

Following the **procedure A**, **1ba** was obtained as a blue liquid (R_f= 0.3, petroleum ether) in 24% yield (1.0 g, 2.4 mmol). **^{1}H NMR (400 MHz, CDCl$_3$)** δ 7.80 (m, 1H), 7.33 – 7.27 (m, 1H), 7.23 (m, 2H), 7.03 (m, 1H), 6.79 (m, 1H), 6.73 (m, 1H), 5.87 (d, *J* = 1.8 Hz, 1H), 5.23 (q, *J* = 1.8 Hz, 1H), 4.63 (t, *J* = 1.8 Hz, 2H), 0.30 (s, 9H). **^{13}C NMR (101 MHz, CDCl$_3$)** δ 161.18 (d, *J* = 248.5 Hz), 156.37, 144.12, 141.26 (d, *J* = 3.4 Hz), 141.01 (d, *J* = 3.3 Hz), 138.98, 129.93 (d, *J* = 7.5 Hz), 128.80, 122.18, 120.64 (d, *J* = 18.6 Hz), 116.34, 114.68 (d, *J* = 20.9 Hz), 111.53, 85.89, 71.29, -0.00. **^{19}F NMR (376 MHz, CDCl$_3$)** δ -115.62. **MS (EI)** calcd. for $C_{18}H_{20}FIOSi$ $[M]^+$: 426.03. Found: 425.88.

(2-(3-(2-Iodophenoxy)prop-1-en-2-yl)-5-methylphenyl)trimethylsilane (1bb)

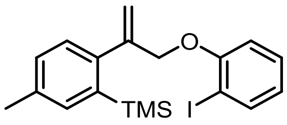

1bb

Following the **procedure A**, **1bb** was obtained as a pale yellow solid (R_f = 0.4, petroleum ether) in 48% yield (2.0 g, 4.8 mmol). **^{1}H NMR (400 MHz, $CDCl_3$)** δ 7.82 (m, 1H), 7.42 (s, 1H), 7.30 (m, 1H), 7.22 – 7.15 (m, 2H), 6.81 (m, 1H), 6.74 (m, 1H), 5.89 (t, J = 1.7 Hz, 1H), 5.24 (q, J = 1.7 Hz, 1H), 4.68 (m, 2H), 2.39 (s, 3H), 0.32 (s, 9H). **^{13}C NMR (101 MHz, $CDCl_3$)** δ 156.20, 144.55, 142.16, 138.68, 137.61, 135.40, 134.88, 128.54, 128.44, 127.76, 121.80, 115.11, 111.26, 85.66, 71.00, 20.44, 0.00. **MS (EI)** calcd. for $C_{19}H_{23}IOSi$ $[M]^+$: 422.06. Found: 422.22.

(2-(3-(2-Iodophenoxy)prop-1-en-2-yl)-5-(trifluoromethyl)phenyl)trimethylsilane (1bc)

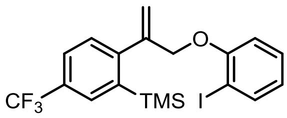

1bc

Following the **procedure A**, **1bc** was obtained as a colorless oil (R_f = 0.3, petroleum ether) in 33% yield (1.6 g, 3.3 mmol). **^{1}H NMR (400 MHz, $CDCl_3$)** δ 7.83 – 7.78 (m, 2H), 7.59 (m, 1H), 7.40 (m, 1H), 7.29 (m, 1H), 6.79 (m, 1H), 6.77 – 6.71 (m, 1H), 5.92 (q, J = 1.8 Hz, 1H), 5.27 (q, J = 1.8 Hz, 1H), 4.79 – 4.50 (q, J = 1.8 Hz, 2H), 0.33 (s, 9H). **^{13}C NMR (101 MHz, $CDCl_3$)** δ 160.61, 160.52, 141.57, 137.61, 129.97 (q, J = 31.8Hz), 129.22 (q, J = 3.6 Hz), 128.09 (q, J = 3.5 Hz), 126.63, 124.05, 122.55 (q, J = 271 Hz), 122.29, 110.68, 87.45, 78.00, 58.43, 29.24, -0.77.**^{19}F NMR (376 MHz, $CDCl_3$)** δ -62.49. **MS (EI)** calcd. for $C_{19}H_{20}F_3IOSi$ $[M]^+$: 476.03. Found: 476.22.

(4-Fluoro-2-(3-(2-iodophenoxy)prop-1-en-2-yl)phenyl)trimethylsilane (1bd)

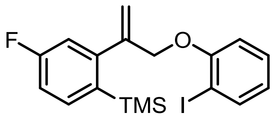

1bd

Following the **procedure A**, **1bd** was obtained as a colorless oil (R_f = 0.5, petroleum ether) in 42% yield (1.8 g, 4.2 mmol). **^{1}H NMR (400 MHz, $CDCl_3$)** δ 7.81 (m, 1H), 7.61 – 7.51 (m, 1H), 7.38 – 7.27 (m, 1H), 7.08 – 6.98 (m, 2H), 6.81 (m, 1H), 6.74 (m, 1H), 5.89 (q, *J* = 1.7 Hz, 1H), 5.28 (q, *J* = 1.7 Hz, 1H), 4.66 (t, *J* = 1.7 Hz, 2H), 0.30 (s, 9H). **^{13}C NMR (101 MHz, $CDCl_3$)** δ 163.57 (d, *J* = 249.3 Hz), 156.06, 147.38 (d, *J* = 7.0 Hz), 143.67, 138.73, 136.10, 133.28, 133.25, 128.57, 121.97, 115.02, (d, *J* = 19.9 Hz), 113.00 (d, *J* = 19.0 Hz), 111.21, 85.61, 70.75, 0.02. **^{19}F NMR (376 MHz, $CDCl_3$)** δ -112.96. **MS (EI)** calcd. for $C_{18}H_{20}FIOSi$ $[M]^+$: 426.03. Found: 426.34.

(4-Chloro-2-(3-(2-iodophenoxy)prop-1-en-2-yl)phenyl)trimethylsilane (1be)

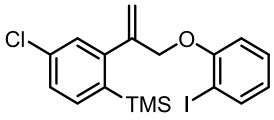

1be

Following the **procedure A**, **1be** was obtained as a colorless liquid (R_f = 0.3, petroleum ether) in 43% yield (1.9 g, 4.3 mmol). **^{1}H NMR (400 MHz, $CDCl_3$)** δ 7.81 (m, 1H), 7.51 (m, 1H), 7.35 – 7.26 (m, 3H), 6.81 (m, 1H), 6.75 (m, 1H), 5.96 – 5.74 (m, 1H), 5.39 – 5.14 (m, 1H), 4.70 – 4.60 (m, 2H), 0.30 (s, 9H). **^{13}C NMR (101 MHz, $CDCl_3$)** δ 156.15, 146.79, 143.72, 138.84, 136.20, 135.61, 133.94, 128.65, 128.05, 126.19, 122.07, 116.49, 111.32, 85.71, 70.91, 0.00. **MS (EI)** calcd. for $C_{18}H_{20}C_lIOSi$ $[M]^+$: 442.00. Found: 441.97.

(2-(3-(2-Iodophenoxy)prop-1-en-2-yl)-4-methylphenyl)trimethylsilane (1bf)

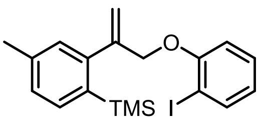

1bf

Following the **procedure A**, **1bf** was obtained as a yellow oil (R_f = 0.5, petroleum ether) in 40% yield (1.7 g, 4.0 mmol). **^{1}H NMR (400 MHz, $CDCl_3$)** δ 7.81 (m, 1H), 7.49 (m, 1H), 7.32 – 7.26 (m, 1H), 7.15 (m, 1H), 7.12 (m, 1H), 6.81 (m, 1H), 6.74 (m, 1H), 5.86 (q, J = 1.8 Hz, 1H), 5.24 (q, J = 1.8 Hz, 1H), 4.67 (t, J = 1.8 Hz, 2H), 2.37 (s, 3H), 0.29 (s, 9H). **^{13}C NMR (101 MHz, $CDCl_3$)** δ 156.23, 145.13, 144.71, 138.70, 137.59, 134.30, 134.12, 128.66, 128.54, 126.86, 121.81, 115.19, 111.26, 85.64, 71.03, 20.35, 0.00. **MS (EI)** calcd. For $C_{19}H_{23}IOSi$ $[M]^+$: 422.06. Found: 422.32

(2-(3-(2-Iodophenoxy)prop-1-en-2-yl)-4-methoxyphenyl)trimethylsilane (1bg)

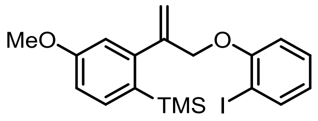

1bg

Following the **procedure A**, **1bg** was obtained as a yellow oil (R_f = 0.3, petroleum ether) in 52% yield (2.3 g, 5.2 mmol). **^{1}H NMR (400 MHz, $CDCl_3$)** δ 7.81 (m, 1H), 7.51 (d, J = 8.3 Hz, 1H), 7.28 (m, 1H), 6.88 (dd, J = 8.3, 2.4 Hz, 1H), 6.84 – 6.76 (m, 2H), 6.73 (d, J = 2.4 Hz, 1H), 5.87 (q, J = 1.8 Hz, 1H), 5.26 (q, J = 1.8 Hz, 1H), 4.67 (t, J = 1.8 Hz, 2H), 3.83 (s, 3H), 0.27 (s, 9H). **^{13}C NMR (101 MHz, $CDCl_3$)** δ 158.89, 156.13, 146.71, 144.42, 138.63, 135.61, 128.61, 128.48, 121.79, 115.25, 113.65, 111.55, 111.21, 85.58, 70.87, 54.24, -0.00. **MS (EI)** calcd. for $C_{19}H_{23}IO_2Si$ $[M]^+$: 438.05. Found: 437.95.

(2-(3-(2-iodophenoxy)prop-1-en-2-yl)-4,5-dimethoxyphenyl)trimethylsilane (1bh)

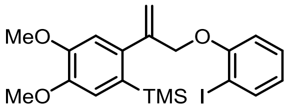

1bh

Following the **procedure A**, **1bh** was obtained as a yellow oil (R_f = 0.4, petroleum ether) in 45% yield (2.1 g, 4.5 mmol). **^{1}H NMR (400 MHz, $CDCl_3$)** δ 7.80 (m, 1H), 7.29 (m, 1H), 7.04 (s, 1H), 6.84 – 6.79 (m, 2H), 6.77 – 6.67 (m, 1H), 5.87 (t, J = 1.7 Hz, 1H), 5.27 (d, J = 1.7 Hz, 1H), 4.66 (t, J = 1.7 Hz, 2H), 3.91 (s, 3H), 3.89 (s, 3H), 0.29 (s, 9H). **^{13}C NMR (101 MHz, $CDCl_3$)** δ 156.10, 148.13, 146.64, 144.32, 138.61, 138.52, 128.73, 128.48, 121.77, 116.40, 115.87, 111.35, 111.17, 85.56, 71.10, 54.97, 54.93, -0.00. **MS (EI)** calcd. for $C_{20}H_{25}IO_3Si$ $[M]^+$: 468.06. Found: 468.00.

(6-(3-(2-Iodophenoxy)prop-1-en-2-yl)benzo[d][1,3]dioxol-5-yl)trimethylsilane (1bi)

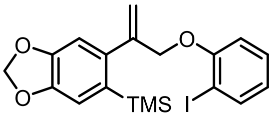

1bi

Following the **procedure A**, **1bi** was obtained as a colorless oil (R_f = 0.4, petroleum ether) in 54% yield (2.3 g, 5.5 mmol). **^{1}H NMR (400 MHz, $CDCl_3$))** δ 7.81 (m, 1H), 7.33 – 7.26 (m, 1H), 7.04 (s, 1H), 6.85 – 6.77 (m, 2H), 6.74 (m, 1H), 5.96 (s, 2H), 5.85 (t, J = 1.8 Hz, 1H), 5.24 (t, J = 1.8 Hz, 1H), 4.63 (d, J = 1.8 Hz, 2H), 0.28 (s, 9H). **^{13}C NMR (101 MHz, $CDCl_3$)** δ 156.00, 146.84, 145.68, 143.98, 139.33, 138.56, 130.49, 128.42, 121.73, 115.78, 113.01, 111.07, 108.71, 99.92, 85.49, 70.88, 0.00. **MS (EI)** calcd. for $C_{19}H_{21}IO_3Si$ $[M]^+$: 452.03. Found: 451.88.

(2-(3-(2-Iodophenoxy)prop-1-en-2-yl)-6-methylphenyl)trimethylsilane (1bj)

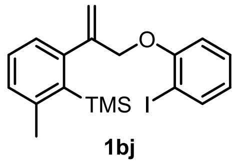

1bj

Following the **procedure A**, **1bj** was obtained as a colorless oil (R_f = 0.3, petroleum ether) in 37% yield (1.6 g, 3.7 mmol). **^{1}H NMR (400 MHz, $CDCl_3$)** δ 7.80 (m, 1H), 7.39 (s, 1H), 7.32 – 7.27 (m, 1H), 7.17 (s, 2H), 6.79 (m, 1H), 6.73 (m, 1H), 5.86 (d, *J* = 1.8 Hz, 1H), 5.22 (d, *J* = 1.8 Hz, 1H), 4.65 (t, *J* = 1.8 Hz, 2H), 2.37 (s, 3H), 0.29 (s, 9H). **^{13}C NMR (101 MHz, $CDCl_3$)** δ 156.22, 144.55, 142.18, 138.69, 137.63, 135.41, 134.89, 128.55, 128.45, 127.76, 121.81, 115.11, 111.28, 85.67, 71.02, 20.43, -0.02. **MS (EI)** calcd. for $C_{19}H_{23}IOSi$ $[M]^+$: 422.06. Found: 422.00.

(2-(3-((2-Iodobenzyl)oxy)prop-1-en-2-yl)phenyl)trimethylsilane (1ca)

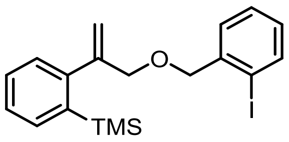

1ca

Following the **procedure B**, **1ca** was obtained as a colorless oil (R_f= 0.3, petroleum ether) in 44% yield (1.9 g, 4.4 mmol). **^{1}H NMR (400 MHz, $CDCl_3$)** δ 7.60 (m, 1H), 7.34 (m, 1H), 7.28 (m, 1H), 7.19 – 7.00 (m, 3H), 6.95 (m, 1H), 6.77 (m, 1H), 5.36 (p, *J* = 1.7 Hz, 1H), 4.90 (p, *J* = 1.7 Hz, 1H), 4.40 (d, *J* = 1.7 Hz, 2H), 4.03 (q, *J* = 1.7 Hz, 2H), 0.06 (s, 9H). **^{13}C NMR (101 MHz, $CDCl_3$)** δ 146.73, 145.85, 139.58, 138.16, 137.39, 134.02, 128.18, 127.65, 127.53, 127.49, 127.29, 125.62, 113.86, 96.55, 75.57, 73.12, 0.00. **MS (EI)** calcd. For $C_{19}H_{23}IOSi$ $[M]^+$: 422.06. Found: 422.26.

***N*-(2-iodophenyl)-4-methyl-*N*-(2-(2(trimethylsilyl) phenyl) allyl) benzene sulfonamide (1da)**

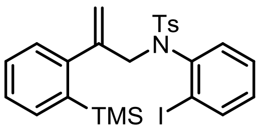

1da

Following the **procedure C**, **1da** was obtained as a yellow solid (R_f = 0.4, petroleum ether/ethyl acetate = 20:1) in 46% yield (2.4 g, 4.6 mmol). **^{1}H NMR (400 MHz, $CDCl_3$)** δ 7.84 (m, 1H), 7.58 – 7.53 (m, 2H), 7.50 – 7.45 (m, 1H), 7.28 (m, 2H), 7.23 (m, 4H), 7.08 – 6.88 (m, 2H), 5.66 (q, *J* = 1.5 Hz, 1H), 5.12 (q, *J* = 1.5 Hz, 1H), 4.68 (dt, *J* = 17.5, 1.5 Hz, 1H), 4.35 (dt, *J* = 17.5, 1.5 Hz, 1H), 2.42 (s, 3H), 0.09 (s, 9H). **^{13}C NMR (101 MHz, $CDCl_3$)** δ 146.22, 145.33, 142.99, 140.88, 140.14, 137.40, 136.06, 134.29, 132.22, 129.03, 128.81, 128.04, 127.91, 127.44, 125.93, 116.71, 99.94, 57.13, 20.93, -0.00. **MS (EI)** calcd. For $C_{25}H_{28}INO_2SSi$ $[M]^+$: 561.07. Found: 560.95.

***N*-(2-iodophenyl)-*N*-(2-(2-(trimethylsilyl)phenyl)allyl)methanesulfonamide (1db)**

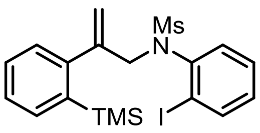

1db

Following the **procedure C**, **1db** was obtained as a yellow solid (R_f = 0.4, petroleum ether/ethyl acetate = 20:1r) in 46% yield (1.6 g, 4.6 mmol). **^{1}H NMR (400 MHz, $CDCl_3$)** δ 7.93 (m, 1H), 7.50 (m, 1H), 7.46 (m, 1H), 7.36 (m, 1H), 7.33 – 7.19 (m, 2H), 7.13 – 7.01 (m, 2H), 5.66 (q, *J* = 1.7 Hz, 1H), 5.17 (q, *J* = 1.3 Hz, 1H), 4.83 (dt, *J* = 17.7, 1.7 Hz, 1H), 4.30 (dt, *J* = 17.7, 1.3 Hz, 1H), 3.02 (s, 3H), 0.10 (s, 9H). **^{13}C NMR (101 MHz, $CDCl_3$)** δ 146.11, 145.79, 140.53, 140.19, 137.56, 134.47, 133.24, 129.43, 128.43, 128.25, 128.00, 126.12, 116.46, 98.95, 57.28, 40.83, 0.00. **MS (EI)** calcd. For $C_{19}H_{24}INO_2SSi$ $[M]^+$: 485.03. Found: 485.01.

N-(2-iodophenyl)-N-(2-(2-(trimethylsilyl)phenyl)allyl)acetamide (1dc)

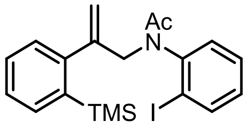

1dc

Following the **procedure C**, **1dc** was obtained as a yellow solid (R_f = 0.4, petroleum ether/ethyl acetate = 20:1 in 37% yield (1.7 g, 3.7 mmol). **^{1}H NMR (400 MHz, $CDCl_3$)** δ 7.94 (m, 1H), 7.52 (m, 1H), 7.34 (m, 1H), 7.31 – 7.23 (m, 2H), 7.14 (m, 1H), 7.10 – 7.03 (m, 2H), 5.41 (q, J = 1.7 Hz, 1H), 5.24 (dt, J = 16.9, 1.7 Hz, 1H), 5.13 (q, J = 1.4 Hz, 1H), 3.58 (dt, J = 16.9, 1.4 Hz, 1H), 1.85 (s, 3H), 0.17 (s, 9H). **^{13}C NMR (101 MHz, $CDCl_3$)** δ 169.62, 146.49, 145.04, 144.92, 139.64, 137.38, 134.18, 129.64, 129.09, 128.57, 127.87, 127.76, 125.77, 114.34, 99.32, 54.78, 22.06, -0.00. **MS (EI)** calcd. For $C_{20}H_{24}INOSi$ $[M]^+$: 449.07. Found: 449.02.

N-(4-fluoro-2-iodophenyl)-4-methyl-N-(2-(2-(trimethylsilyl)phenyl)allyl)benzene-sulfonamide (1dd)

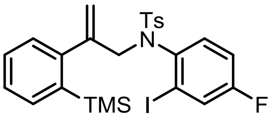

1dd

Following the **procedure C**, **1dd** was obtained as a yellow solid (R_f = 0.4, petroleum ether/ethyl acetate = 20:1) in 41% yield (2.4 g, 4.1 mmol). **^{1}H NMR (400 MHz, $CDCl_3$)** δ 7.55 (m, 3H), 7.52 – 7.45 (m, 1H), 7.26 – 7.21 (m, 4H), 7.14 (m, 1H), 6.99 (m, 2H), 5.62 (q, J = 1.5 Hz, 1H), 5.13 (d, J = 1.5 Hz, 1H), 4.68 (dt, J = 17.3, 1.5 Hz, 1H), 4.32 (dt, J = 17.3, 1.5 Hz, 1H), 2.42 (s, 3H), 0.11 (s, 9H). **^{13}C NMR (101 MHz, $CDCl_3$)** δ 160.44 (d, J = 254.1 Hz), 146.09, 145.32, 143.14, 137.40, 137.25 (d, J = 3.5 Hz), 135.93, 134.36, 133.00 (d, J = 9.0 Hz), 128.88, 128.00 (d, J = 13.7 Hz), 127.44, 126.93, 126.68, 125.99, 116.82, 114.90 (d, J = 22.0 Hz), 99.86 (d, J = 8.5 Hz), 57.29, 20.92, 0.00. **^{19}F NMR (376 MHz, $CDCl_3$)** δ -111.42. **MS (EI)** calcd. For $C_{25}H_{27}FINO_2SSi$ $[M]^+$: 579.06. Found: 578.92.

N-(2-iodo-4-(trifluoromethyl)phenyl)-4-methyl-N-(2-(2-(trimethylsilyl)phenyl)-allyl)benzenesulfonamide (1de)

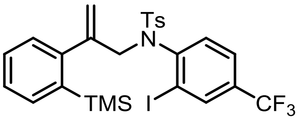

1de

Following the **procedure C**, **1de** was obtained as a yellow solid (R_f = 0.4, petroleum ether/ethyl acetate = 20:1) in 43% yield (1.8 g, 4.3 mmol). **^{1}H NMR (400 MHz, $CDCl_3$)** δ 8.07 (m, 1H), 7.57 (s, 1H), 7.55 (s, 1H), 7.54 – 7.50 (m, 1H), 7.48 (m, 1H), 7.25 (m, 5H), 6.92 (m, 1H), 5.61 (m, 1H), 5.17 – 5.10 (m, 1H), 4.67 (d, J = 17.2 Hz, 1H), 4.40 (d, J = 17.2 Hz, 1H), 2.43 (s, 3H), 0.09 (s, 9H). **^{13}C NMR (101 MHz, $CDCl_3$)** δ 145.81, 145.11, 144.50, 143.46, 137.42, 137.06 (q, J = 3.7 Hz), 135.78, 134.45, 132.31, 130.73 (q, J = 33.2 Hz), 129.06, 128.08, 127.98, 127.42, 126.12, 124.85 (q, J = 3.7 Hz), 121.89 (q, J = 273.1 Hz), 117.12, 99.90, 56.99, 20.96, 0.00. **^{19}F NMR (376 MHz, $CDCl_3$)** δ -61.58. **MS (EI)** calcd. For $C_{26}H_{27}F_3INO_2SSi$ $[M]^+$: 629.05. Found: 629.00.

N-(2-iodo-4-methylphenyl)-4-methyl-N-(2-(2-(trimethylsilyl)phenyl)allyl)benzene -sulfonamide (1df)

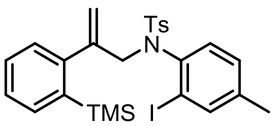

1df

Following the **procedure C**, **1df** was obtained as a yellow solid (R_f = 0.4, petroleum ether/ethyl acetate = 20:1) in 39% yield (2.2 g, 3.9 mmol). **^{1}H NMR (400 MHz, $CDCl_3$)** δ 7.67 (m, 1H), 7.56 (m, 2H), 7.51 – 7.45 (m, 1H), 7.26 – 7.22 (m, 3H), 7.22 – 7.20 (m, 1H), 7.09 (m, 2H), 6.99 – 6.94 (m, 1H), 5.65 (q, J = 1.4 Hz, 1H), 5.11 (d, J = 1.4 Hz, 1H), 4.65 (dt, J = 17.3 Hz, 1.4 Hz, 1H), 4.33 (dt, J = 17.3, 1.4 Hz, 1H), 2.41 (s, 3H), 2.30 (s, 3H), 0.11 (s, 9H). **^{13}C NMR (101 MHz, $CDCl_3$)** δ 146.32, 145.29, 142.84, 140.50, 139.35, 138.14, 137.43, 136.14, 134.26, 131.63, 128.75, 128.69,

128.01, 127.86, 127.44, 125.87, 116.64, 99.64, 57.11, 20.91, 19.82, 0.00. **MS (EI)** calcd. For $C_{26}H_{30}INO_2SSi$ $[M]^+$: 575.08. Found: 575.02

***N*-(2-iodo-4-methoxyphenyl)-4-methyl-*N*-(2-(2-(trimethylsilyl)phenyl)allyl)benze -nesulfonamide (1dg)**

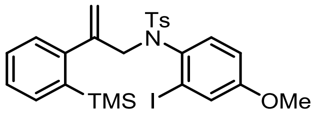

1dg

Following the **procedure C**, **1dg** was obtained as a yellow solid (R_f = 0.4, petroleum ether/ethyl acetate = 20:1) in 34% yield (2.0 g, 3.4 mmol). **^{1}H NMR (400 MHz, $CDCl_3$)** δ 7.56 (m, 1H), 7.54 (m, 1H), 7.51 – 7.44 (m, 1H), 7.34 (m, 1H), 7.23 (m, 4H), 7.10 (m, 1H), 7.01 – 6.94 (m, 1H), 6.81 (m, 1H), 5.64 (d, *J* = 1.4 Hz, 1H), 5.12 (q, *J* = 1.4 Hz, 1H), 4.66 (dt, *J* = 17.4, 1.4 Hz, 1H), 4.31 (dt, *J* = 17.4, 1.4 Hz, 1H), 3.79 (s, 3H), 2.42 (s, 3H), 0.12 (s, 9H). **^{13}C NMR (101 MHz, $CDCl_3$)** δ 158.49, 146.35, 145.39, 142.81, 137.42, 136.13, 134.26, 133.51, 132.39, 128.75, 128.02, 127.86, 127.44, 125.86, 124.80, 116.62, 113.54, 100.23, 57.31, 54.98, 20.90, 0.00. **MS (EI)** calcd. For $C_{26}H_{30}INO_3SSi$ $[M]^+$: 591.08. Found: 591.03.

***N*-(5-chloro-2-iodophenyl)-4-methyl-*N*-(2-(2-(trimethylsilyl)phenyl)allyl)benzene -sulfonamide (1dh)**

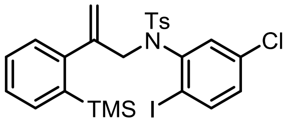

1dh

Following the **procedure C**, **1dh** was obtained as a yellow solid (R_f = 0.4, petroleum ether/ethyl acetate = 20:1) in 42% yield (2.5 g, 4.2 mmol). **^{1}H NMR (400 MHz, $CDCl_3$)** δ 7.73 (m, 1H), 7.57 (m, 2H), 7.48 (m, 1H), 7.25 (m, 4H), 7.03 (m, 1H), 6.99 (m, 1H), 6.94 – 6.90 (m, 1H), 5.60 (s, 1H), 5.14 (s, 1H), 4.64 (d, *J* = 17.1 Hz, 1H), 4.33 (d, *J* = 17.1 Hz, 1H), 2.43 (s, 3H), 0.10 (s, 9H). **^{13}C NMR (101 MHz, $CDCl_3$)** δ 145.81, 145.13, 143.30, 142.20, 140.50, 137.33, 135.76, 134.36, 133.63, 132.30,

129.25, 128.91, 128.09, 127.93, 127.43, 126.11, 117.10, 97.36, 57.10, 20.94, 0.00. **MS (EI)** calcd. For $C_{25}H_{27}ClINO_2SSi$ [M]$^+$: 595.03. Found: 594.99.

Benzyl(2-(3-(2-iodophenoxy)prop-1-en-2-yl)phenyl)dimethylsilane (1ea)

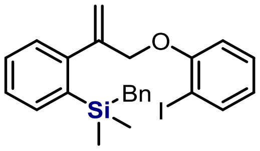

1ea

Following the **procedure A**, **1ea** was obtained as a yellow oil (R_f = 0.3, petroleum ether) in 49% yield (2.4 g, 4.9 mmol). **^{1}H NMR (400 MHz, $CDCl_3$)** δ 7.79 (m, 1H), 7.55 (m, 1H), 7.42 – 7.22 (m, 4H), 7.16 (m, 2H), 7.09 – 7.02 (m, 1H), 6.97 – 6.88 (m, 2H), 6.73 (m, 2H), 5.89 (q, *J* = 1.8 Hz, 1H), 5.20 (q, *J* = 1.8 Hz, 1H), 4.60 (t, *J* = 1.8 Hz, 2H), 2.36 (s, 2H), 0.25 (s, 6H). **^{13}C NMR (101 MHz, $CDCl_3$)** δ 158.38, 147.46, 147.01, 141.20, 140.94, 138.30, 136.85, 130.79, 130.21, 130.16, 129.80, 129.50, 128.23, 125.51, 124.15, 117.62, 113.61, 87.94, 73.24, 28.54, 0.00. **MS (EI)** calcd. for $C_{24}H_{25}IOSi$ [M]$^+$: 484.07. Found: 484.06.

Ethyl(2-(3-(2-iodophenoxy)prop-1-en-2-yl)phenyl)dimethylsilane (1eb)

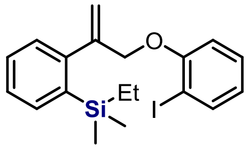

1eb

Following the **procedure A**, **1eb** was obtained as a yellow solid (R_f = 0.3, petroleum ether) in 37% yield (1.6 g, 3.7 mmol). **^{1}H NMR (400 MHz, $CDCl_3$)** δ 7.88 (m, 1H), 7.74 – 7.60 (m, 1H), 7.42 (m, 2H), 7.39 – 7.31 (m, 2H), 6.88 (m, 1H), 6.80 (m, 1H), 5.98 (d, *J* = 1.8 Hz, 1H), 5.33 (d, *J* = 1.8 Hz, 1H), 4.76 (t, *J* = 1.8 Hz, 2H), 1.11 – 0.95 (m, 3H), 0.95 – 0.76 (m, 2H), 0.39 (s, 6H). **^{13}C NMR (101 MHz, $CDCl_3$)** δ 158.56, 147.59, 147.09, 141.07, 139.09, 136.91, 130.93, 130.23, 130.04, 128.31, 124.23,

117.48, 113.67, 88.11, 73.34, 10.19, 9.25, 0.00. **MS (EI)** calcd. for $C_{19}H_{23}IOSi$ $[M]^+$: 422.06. Found: 422.04.

1-(4,5-Dimethoxy-2-(1-(4-methoxyphenyl)vinyl)phenyl)-1-methylsiletane (1ec)

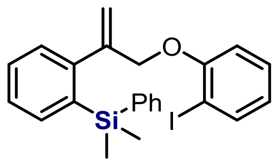

1ec

Following the **procedure A**, **1ec** was obtained as a colorless oil (R_f = 0.3, petroleum ether) in 46% yield (2.2 g, 4.6 mmol). **^{1}H NMR (400 MHz, CDCl$_3$)** δ 7.76 (m, 1H), 7.66 – 7.59 (m, 1H), 7.49 – 7.43 (m, 2H), 7.41 – 7.29 (m, 2H), 7.28 – 7.11 (m, 5H), 6.68 (m, 1H), 6.37 (m, 1H), 5.75 (d, J = 1.8 Hz, 1H), 5.09 (q, J = 1.8 Hz, 1H), 4.10 (t, J = 1.8 Hz, 2H), 0.59 (s, 6H). **^{13}C NMR (101 MHz, CDCl$_3$)** δ 157.45, 147.08, 145.92, 139.91, 139.70, 137.33, 136.34, 134.78, 133.59, 129.80, 129.59, 129.40, 128.27, 127.39, 123.01, 117.28, 112.62, 86.96, 71.77, 0.00. **MS (EI)** calcd. for $C_{23}H_{23}IOSi$ $[M]^+$: 470.06. Found: 470.04.

Triethyl(2-(3-(2-iodophenoxy)prop-1-en-2-yl)phenyl)silane (1ed)

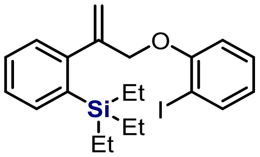

1ed

Following the **procedure A**, **1ed** was obtained as a colorless oil (R_f = 0.3, petroleum ether) in 40% yield (1.8 g, 4.0 mmol). **^{1}H NMR (400 MHz, CDCl$_3$)** δ 7.73 (m, 1H), 7.47 (m, 1H), 7.33 – 7.12 (m, 4H), 6.73 (m, 1H), 6.66 (m, 1H), 5.77 (q, J = 1.8 Hz, 1H), 5.12 (q, J = 1.8 Hz, 1H), 4.59 (t, J = 1.8 Hz, 2H), 0.91 – 0.80 (m, 9H), 0.81 – 0.71 (m, 6H). **^{13}C NMR (101 MHz, CDCl$_3$)** δ 157.13, 146.49, 145.62, 139.59, 136.12, 135.34, 129.45, 128.92, 128.42, 126.64, 122.75, 115.69, 112.24, 86.62, 71.82, 7.66, 4.42. **MS (EI)** calcd. for $C_{21}H_{27}IOSi$ $[M]^+$: 450.09. Found: 450.04.

Pd-Catalyzed Spirocyclization Reaction of 1

Condition Screening

Supplementary Table S1. Optimization of reaction conditions [a]

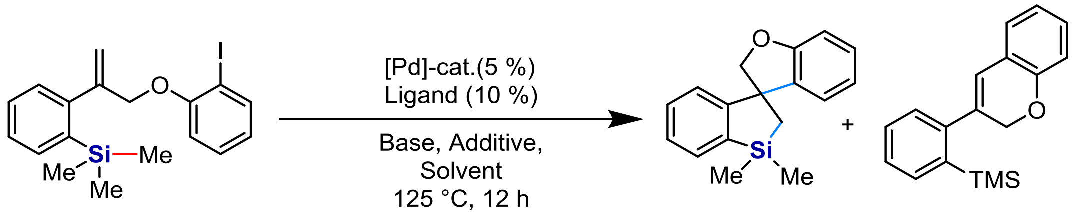

1aa **2aa** **3aa**

Entry	[Pd] Source	Ligand	Base	Additive (X eq.)	Solvent	**2aa**[%] [b]	**3aa**[%][b]
1	$[Pd(allyl)Cl]_2$	P^tBu_3	LiO^tBu	$p\text{-}NO_2PhCHO$ (1)	PhMe	10	28
2	$[Pd(allyl)Cl]_2$	P^tBu_3	LiO^tBu	KBr (2)	PhMe	34	20
3	$[Pd(allyl)Cl]_2$	P^tBu_3	LiO^tBu	AgBr (2)	PhMe	45	Trace
4	$[Pd(allyl)Cl]_2$	P^tBu_3	LiO^tBu	NaBr (2)	PhMe	20	25
5	$Pd(P^tBu)_3$	-	LiO^tBu	AgBr (2)	PhMe	Trace	40
6	$Pd(dppf)Cl_2$	-	LiO^tBu	AgBr (2)	PhMe	47	34
**7**	**[Pd]-1**	-	LiO^tBu	AgBr (2)	PhMe	38	Trace
8	**[Pd]-1**	-	LiO^tBu	Ag_3PO_4 (2)	PhMe	57	12
9	**[Pd]-1**	-	LiO^tBu	Ag_2CO_3 (2)	PhMe	20	Trace
10	**[Pd]-1**	-	LiO^tBu	AgOAc (2)	PhMe	67	28
11	**[Pd]-1**	-	LiO^tBu	TcCu (1)	PhMe	40	N.D.
12	**[Pd]-1**	-	LiO^tBu	-	PhMe	20	10
13	**[Pd]-1**	-	LiO^tBu	AgOAc(2)+TcCu (1)	PhMe	72	Trace
14	**[Pd]-1**	-	LiO^tBu	AgOAc(2)+TcCu (1)	Dioxane	40	Trace
15	**[Pd]-1**	-	LiO^tBu	AgOAc(2)+TcCu (1)	PhCl	65	Trace
16	**[Pd]-1**	-	LiO^tBu	AgOAc(2)+TcCu (1)	DCE	68	Trace
17	**[Pd]-1**	-	LiO^tBu	AgOAc(2)+TcCu (1)	Cy	72[c]	Trace
18	**[Pd]-1**	-	LiO^tBu	AgOAc(2)+TcCu (0.5)	Cy	84[c]	Trace
**19**	**[Pd]-1**	**-**	**LiO^tBu**	**AgOAc(2)+TcCu (0.2)**	**Cy**	**90[c]**	**Trace**
20	**[Pd]-1**	-	LiO^tBu	AgOAc(2)+TcCu (0.5)	PhMe	85[c]	10
21	**[Pd]-1**	-	NaO^tBu	AgOAc(2)+TcCu (0.2)	Cy	N.R.	-
22	**[Pd]-1**	-	KO^tBu	AgOAc(2)+TcCu (0.2)	Cy	N.R.	-

[a] Reactions were carried out by using **[Pd]-1** catalyst (5 mol%), ligand (10 mol%), base (0.6 mmol, 3.0 equiv.), **1aa** (0.2 mmol) in solvent (0.4 M) for 12 h at 125 °C under an N_2 atmosphere. [b] Yield was determined by NMR analysis of the mixture. [c] Isolated Yield. N.D.: No Detected. N.R.: No Reaction.

General procedure

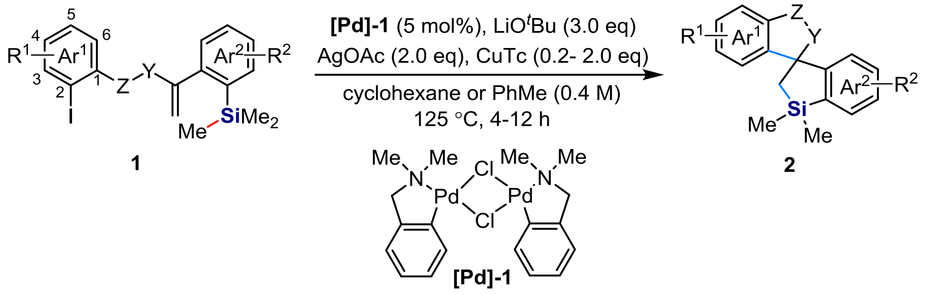

In a nitrogen-filled glovebox, an oven-dried 15 mL screw capped sealed tube was charged with a magnetic stir bar, **1** (0.20 mmol), **[Pd]-1** (5 mmol%), AgOAc (2 equiv), LiO*t*Bu (3 equiv), additive and cyclohexane or PhMe (0.5 mL). The tube was sealed, then removed from the glovebox, and the formed mixture was stirred at 125 ˚C under N_2 for 12 h. After being cooled to room temperature, Saturated aqueous NH_4Cl (5 mL) was added and the mixture was extracted with EA (3 × 5 mL). The combined organic phases were washed with water and brine, dried ($MgSO_4$) and evaporated. The crude product was purified by preparative RP-HPLC on reversed phase column (C18(ODS)) (eluent: CH_3CN) to afford the corresponding product.

Characterization of Products 2

1',1'-Dimethyl-1',2'-dihydro-2*H*-spiro[benzofuran-3,3'-benzo[*b*]silole (2aa)

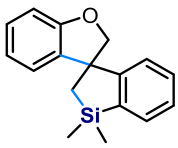

2aa

Following the general procedure, the reaction was carried out with **1aa** (81.6 mg, 0.2 mmol), [Pd]-1 (5.5 mg, 0.01 mmol), AgOAc (66.8 mg, 0.4 mmol), LiO*t*Bu (48 mg,

0.6 mmol), and TcCu (7.7 mg, 0.04 mmol) in cyclohexane (0.5 mL) at 125 °C for 12 h. The title compound was obtained as a white soild (47.9 mg, 0.180 mmol, 90% yield). R_f (petroleum ether/dichloromethane = 20:1) = 0.3. **^{1}H NMR (400 MHz, CDCl$_3$)** δ 7.56 (m, 1H), 7.38 – 7.27 (m, 2H), 7.17 (m, 1H), 7.08 – 7.04 (m, 1H), 6.98 – 6.75 (m, 3H), 4.43 (m, 1H), 4.40 (m, 1H), 1.53 (d, *J* = 15.2 Hz, 1H), 1.35 (d, *J* = 15.2 Hz, 1H), 0.43 (s, 3H), 0.36 (s, 3H). **^{13}C NMR (101 MHz, CDCl$_3$)** δ 160.40, 156.56, 140.40, 138.25, 132.24, 130.98, 128.68, 127.48, 126.18, 123.99, 121.88, 110.26, 87.70, 58.25, 29.33, -0.00, -0.80. **HRMS (EI)** calcd. for $C_{17}H_{18}OSi$ [M]$^+$: 266.1127. Found: 266.1129.

1',1',5-Trimethyl-1',2'-dihydro-2*H*-spiro[benzofuran-3,3'-benzo[*b*]silole] (2ab)

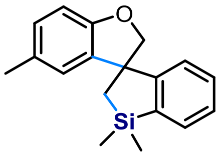

2ab

Following the general procedure, the reaction was carried out with **1ab** (84.4mg, 0.2 mmol), [Pd]-1 (5.5 mg, 0.01 mmol), AgOAc (66.8 mg, 0.4 mmol), LiO^{t}Bu (48 mg, 0.6 mmol), and TcCu (14.7 mg, 0.1 mmol) in PhMe (0.5 mL) at 125 °C for 12 h. The title compound was obtained as a white soild (47 mg, 0.166 mmol, 83% yield). R_f (petroleum ether/dichloromethane = 20:1) = 0.3. **^{1}H NMR (400 MHz, CDCl$_3$)** δ 7.56 (m, 1H), 7.38 – 7.27 (m, 2H), 7.07 (m, 1H), 6.97 (m, 1H), 6.79 (m, 1H), 6.71 – 6.66 (m, 1H), 4.42 (d, *J* = 8.4 Hz, 1H), 4.38 (d, *J* = 8.4 Hz, 1H), 2.25 (s, 3H), 1.52 (d, *J* = 15.1 Hz, 1H), 1.34 (d, *J* = 15.1 Hz, 1H), 0.44 (s, 3H), 0.36 (s, 3H).**^{13}C NMR (101 MHz, CDCl$_3$)** δ 158.32, 156.57, 140.42, 138.17, 132.20, 131.19, 130.92, 129.09, 127.43, 126.22, 124.44, 109.75, 87.74, 58.31, 29.16, 21.49, -0.81, -0.88. **HRMS (EI)** calcd. for $C_{18}H_{20}OSi$ [M]$^+$: 280.1283. Found: 280.1277.

5-Fluoro-1',1'-dimethyl-1',2'-dihydro-2*H*-spiro[benzofuran-3,3'-benzo[*b*]silole] (2ac)

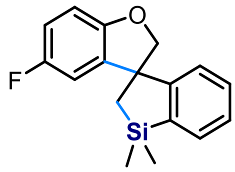

2ac

Following the general procedure, the reaction was carried out with **1ac** (85.2 mg, 0.2 mmol), [Pd]-1 (5.5 mg, 0.01 mmol), AgOAc (66.8 mg, 0.4 mmol), LiO^{t}Bu (48 mg, 0.6 mmol), and TcCu (7.7 mg, 0.04 mmol) in cyclohexane (0.5 mL) at 125 °C for 12 h. The title compound was obtained as a white soild (46 mg, 0.160 mmol, 80% yield). R_f (petroleum ether/dichloromethane = 20:1) = 0.3. **^{1}H NMR (400 MHz, CDCl$_3$)** δ 7.64 – 7.53 (m, 1H), 7.38 – 7.27 (m, 2H), 7.08 (m, 1H), 6.89 – 6.77 (m, 2H), 6.63 (m, 1H), 4.45 (d, *J* = 8.5 Hz, 1H), 4.41 (d, *J* = 8.5 Hz, 1H), 1.55 (d, *J* = 15.2 Hz, 1H), 1.30 (d, *J* = 15.2 Hz, 1H), 0.44 (s, 3H), 0.36 (s, 3H). **^{13}C NMR (101 MHz, CDCl$_3$)** δ 158.70 (d, *J* = 237.6 Hz), 156.30, 155.69, 140.45, 139.67 (d, *J* = 7.5 Hz), 132.35, 131.07, 127.72, 126.08, 114.90 (d, *J* = 24.3 Hz), 111.01 (d, *J* = 24.5 Hz), 110.47 (d, *J* = 8.6 Hz), 88.17, 58.60, 29.09, -0.00, -0.81. **^{19}F NMR (376 MHz, CDCl$_3$)** δ -123.14. **HRMS (EI)** calcd. for $C_{17}H_{17}FOSi$ $[M]^+$: 284.1033. Found: 284.1028.

5-(tert-Butyl)-1',1'-dimethyl-1',2'-dihydro-2*H*-spiro[benzofuran-3,3'-benzo[*b*]-silole] (2ad)

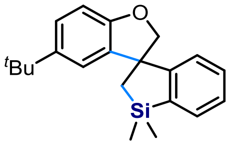

2ad

Following the general procedure, the reaction was carried out with **1ad** (92.8 mg, 0.2 mmol), [Pd]-1 (5.5 mg, 0.01 mmol), AgOAc (66.8 mg, 0.4 mmol), LiO^{t}Bu (48 mg, 0.6 mmol), and TcCu (7.7 mg, 0.04 mmol) in cyclohexane (0.5 mL) at 125 °C for 12 h. The title compound was obtained as a white soild (61.2 mg, 0.190 mmol, 95% yield). R_f (petroleum ether/dichloromethane = 20:1) = 0.3. **^{1}H NMR (400 MHz, CDCl$_3$)** δ 7.62 – 7.54 (m, 1H), 7.37 – 7.26 (m, 2H), 7.20 (m, 1H), 7.07 (m, 1H), 6.97 (m, 1H), 6.82 (m,

1H), 4.41 (d, $J = 8.3$ Hz, 1H), 4.36 (d, $J = 8.3$ Hz, 1H), 1.53 (d, $J = 15.1$ Hz, 1H), 1.35 (d, $J = 15.1$ Hz, 1H), 1.25 (s, 9H), 0.45 (s, 3H), 0.37 (s, 3H). **^{13}C NMR (101 MHz, CDCl$_3$)** δ 158.23, 156.64, 145.00, 140.44, 137.49, 132.23, 130.93, 127.38, 126.13, 125.47, 120.80, 109.28, 87.71, 58.51, 35.08, 32.35, 29.23, 0.00, -0.86. **HRMS (EI)** calcd. for $C_{21}H_{26}OSi$ $[M]^+$: 322.1753. Found: 322.1750.

5-Chloro-1',1'-dimethyl-1',2'-dihydro-2*H*-spiro[benzofuran-3,3'-benzo[*b*]silole] (2ae)

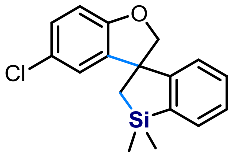

2ae

Following the general procedure, the reaction was carried out with **1ae** (88.4 mg, 0.2 mmol), [Pd]-1 (5.5 mg, 0.01 mmol), AgOAc (66.8 mg, 0.4 mmol), LiO^{t}Bu (48 mg, 0.6 mmol), and TcCu (14.7 mg, 0.1 mmol) in PhMe (0.5 mL) at 125 °C for 12 h. The title compound was obtained as a white soild (44 mg, 0.146 mmol, 73% yield). R_f (petroleum ether/dichloromethane = 20:1) = 0.3. **^{1}H NMR (400 MHz, CDCl$_3$)** δ 7.60 – 7.54 (m, 1H), 7.34 (m, 1H), 7.30 (m, 1H), 7.11 (m, 1H), 7.05 (m, 1H), 6.87 (m, 1H), 6.81 (m, 1H), 4.45 (d, $J = 8.5$ Hz, 1H), 4.41 (d, $J = 8.5$ Hz, 1H), 1.54 (d, $J = 15.1$ Hz, 1H), 1.30 (d, $J = 15.1$ Hz, 1H), 0.44 (s, 3H), 0.35 (s, 3H). **^{13}C NMR (101 MHz, CDCl$_3$)** δ 159.10, 155.65, 140.49, 140.23, 132.38, 131.14, 128.62, 127.78, 126.53, 126.14, 124.23, 111.31, 88.21, 58.43, 29.36, 0.00, -0.82. **HRMS (EI)** calcd. for $C_{17}H_{17}ClOSi$ $[M]^+$: 300.0737. Found: 300.0729.

1',1'-Dimethyl-5-(trifluoromethyl)-1',2'-dihydro-2*H*-spiro[benzofuran-3,3'-benzo [*b*]silole] (2af)

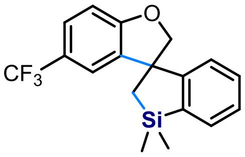

2af

Following the general procedure, the reaction was carried out with **1af** (95.2 mg, 0.2 mmol), [Pd]-1 (5.5 mg, 0.01 mmol), AgOAc (66.8 mg, 0.4 mmol), LiO^{t}Bu (48 mg, 0.6 mmol), and TcCu (14.7 mg, 0.1 mmol) in PhMe (0.5 mL) at 125 °C for 12 h. The title compound was obtained as a white soild (32 mg, 0.096 mmol, 48% yield). R_f (petroleum ether/dichloromethane = 20:1) = 0.2. **^{1}H NMR (400 MHz, CDCl$_3$)** δ 7.61 – 7.54 (m, 1H), 7.48 – 7.42 (m, 1H), 7.39 – 7.28 (m, 2H), 7.18 (m, 1H), 7.01 (m, 1H), 6.95 (m, 1H), 4.51 (d, *J* = 8.5 Hz, 1H), 4.46 (d, *J* = 8.5 Hz, 1H), 1.56 (d, *J* = 15.1 Hz, 1H), 1.32 (d, *J* = 15.1 Hz, 1H), 0.45 (s, 3H), 0.37 (s, 3H). **^{13}C NMR (101 MHz, CDCl$_3$)** δ 163.05, 155.50, 140.54, 139.07, 132.47, 131.23, 127.87, 126.71 (q, *J* = 3.8 Hz), 125.17 (q, *J* = 272.7 Hz), 126.04, 124.36 (q, *J* = 32.3 Hz), 121.49 (q, *J* = 3.5 Hz), 110.38, 88.41, 57.95, 29.76, 0.00, -0.91. **^{19}F NMR (376 MHz, CDCl$_3$)** δ -60.92. **HRMS (EI)** calcd. for $C_{18}H_{17}F_3OSi$ $[M]^+$: 334.1001. Found: 334.1028.

1',1'-Dimethyl-5-phenyl-1',2'-dihydro-2*H*-spiro[benzofuran-3,3'-benzo[*b*]silole] (2ag)

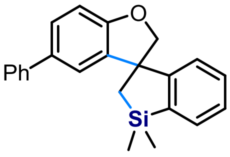

2ag

Following the general procedure, the reaction was carried out with **1ag** (95.2 mg, 0.2 mmol), [Pd]-1 (5.5 mg, 0.01 mmol), AgOAc (66.8 mg, 0.4 mmol), LiO^{t}Bu (48 mg, 0.6 mmol), and TcCu (7.7 mg, 0.04 mmol) in cyclohexane (0.5 mL) at 125 °C for 12 h. The title compound was obtained as a white soild (50.6 mg, 0.148 mmol, 74% yield). R_f (petroleum ether/dichloromethane = 20:1) = 0.3. **^{1}H NMR (400 MHz, CDCl$_3$)** δ 7.63 – 7.56 (m, 1H), 7.52 – 7.45 (m, 2H), 7.43 (m, 1H), 7.39 – 7.23 (m, 5H), 7.17 (m, 1H), 7.15 – 7.10 (m, 1H), 6.97 (m, 1H), 4.49 (d, *J* = 8.5 Hz, 1H), 4.45 (d, *J* = 8.5 Hz, 1H), 1.58 (d, *J* = 15.2 Hz, 1H), 1.40 (d, *J* = 15.2 Hz, 1H), 0.46 (s, 3H), 0.38 (s, 3H). **^{13}C NMR (101 MHz, CDCl$_3$)** δ 159.55, 155.67, 141.23, 139.94, 138.32, 134.83, 131.69,

130.43, 128.64, 127.13, 126.96, 126.75, 126.53, 125.64, 122.18, 109.80, 87.45, 57.74, 28.91, -0.57, -1.40. **HRMS (EI)** calcd. for $C_{23}H_{22}OSi$ $[M]^+$: 342.1440. Found: 342.1470.

6-Methoxy-1',1'-dimethyl-1',2'-dihydro-2*H*-spiro[benzofuran-3,3'-benzo[*b*]silole] (2ah)

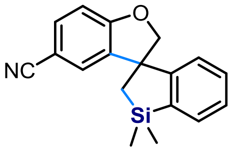

2ah

Following the general procedure, the reaction was carried out with **1ah** (86.6 mg, 0.2 mmol), [Pd]-1 (5.5 mg, 0.01 mmol), AgOAc (66.8 mg, 0.4 mmol), LiO^{t}Bu (48 mg, 0.6 mmol), and TcCu (7.7 mg, 0.04 mmol) in cyclohexane (0.5 mL) at 125 ℃ for 12 h. The title compound was obtained as a white solid (35 mg, 0.120 mmol, 60% yield). R_f (petroleum ether/dichloromethane = 20:1) = 0.2. **^{1}H NMR (400 MHz, $CDCl_3$)** δ 7.58 (m, 1H), 7.49 (m, 1H), 7.38 – 7.28 (m, 2H), 7.20 (m, 1H), 6.98 (m, 1H), 6.94 (m, 1H), 4.54 (d, J = 8.8 Hz, 1H), 4.49 (d, J = 8.8 Hz, 1H), 1.55 (d, J = 15.1 Hz, 1H), 1.29 (d, J = 15.1 Hz, 1H), 0.46 (s, 3H), 0.37 (s, 3H). **^{13}C NMR (101 MHz, $CDCl_3$)** δ 161.45, 152.55, 138.00, 137.60, 131.59, 130.07, 128.82, 125.89, 125.56, 123.41, 117.62, 108.83, 102.63, 86.14, 55.29, 27.43, -2.51, -3.34. **HRMS (EI)** calcd. for $C_{18}H_{17}NOSi$ $[M]^+$: 291.1079. Found: 291.1059.

6-Methoxy-1',1'-dimethyl-1',2'-dihydro-2*H*-spiro[benzofuran-3,3'-benzo[*b*]silole] (2ai)

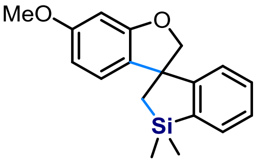

2ai

Following the general procedure, the reaction was carried out with **1ai** (87.6mg, 0.2 mmol), [Pd]-1 (5.5 mg, 0.01 mmol), AgOAc (66.8 mg, 0.4 mmol), LiO^{t}Bu (48 mg,

0.6 mmol), and TcCu (7.7 mg, 0.04 mmol) in cyclohexane (0.5 mL) at 125 °C for 12 h. The title compound was obtained as a white solid (40 mg, 0.136 mmol, 68% yield). R_f (petroleum ether/dichloromethane = 20:1) = 0.3. **^{1}H NMR (400 MHz, $CDCl_3$)** δ 7.57 (m, 1H), 7.38– 7.26 (m, 2H), 7.08 (m, 1H), 6.83 (m, 1H), 6.51 (s, 1H), 6.46 (m, 1H), 4.47 (d, *J* = 8.5 Hz, 1H), 4.43 (d, *J* = 8.5 Hz, 1H), 3.81 (s, 3H), 1.53 (d, *J* = 15.1, 1H), 1.34 (d, *J* = 15.1, 1H), 0.44 (s, 3H), 0.37 (s, 3H). **^{13}C NMR (101 MHz, $CDCl_3$)** δ 161.70, 160.98, 156.86, 140.31, 132.25, 130.98, 130.46, 127.44, 126.10, 124.12, 107.54, 96.72, 88.50, 57.73, 56.17, 29.43, -0.00, -0.80. **HRMS (EI)** calcd. for $C_{18}H_{20}O_2Si$ $[M]^+$: 296.1233. Found: 296.1225.

6-(tert-Butyl)-1',1'-dimethyl-1',2'-dihydro-2*H*-spiro[benzofuran-3,3'-benzo[*b*]silole] (2aj)

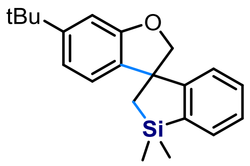

2aj

Following the general procedure, the reaction was carried out with **1aj** (92.8mg, 0.2 mmol), [Pd]-1 (5.5 mg, 0.01 mmol), AgOAc (66.8 mg, 0.4 mmol), LiO^{t}Bu (48 mg, 0.6 mmol), and TcCu (7.7 mg, 0.04 mmol) in cyclohexane (0.5 mL) at 125 °C for 12 h. The title compound was obtained as a white solid (51.5 mg, 0.160 mmol, 80% yield). R_f (petroleum ether/dichloromethane = 20:1) = 0.3. **^{1}H NMR (400 MHz, $CDCl_3$)** δ 7.57 (m, 1H), 7.40 – 7.26 (m, 2H), 7.10 (m, 1H), 7.01 – 6.90 (m, 2H), 6.86 (m, 1H), 4.45 (d, *J* = 8.4 Hz, 1H), 4.41 (d, *J* = 8.4 Hz, 1H), 1.53 (d, *J* = 15.0, 1H), 1.38 (d, *J* = 15.0, 1H), 1.34 (s, 9H), 0.44 (s, 3H), 0.37 (s, 3H). **^{13}C NMR (101 MHz, $CDCl_3$)** δ 161.30, 157.58, 153.30, 141.15, 135.88, 132.99, 131.70, 128.19, 126.99, 123.93, 119.72, 108.27, 88.70, 58.85, 36.26, 32.95, 30.03, 0.81, -0.00. **HRMS (EI)** calcd. for $C_{21}H_{26}OSi$ $[M]^+$: 322.1753. Found: 322.1740.

4-Fluoro-1',1'-dimethyl-1',2'-dihydro-2*H*-spiro[benzofuran-3,3'-benzo[*b*]silole] (2ak)

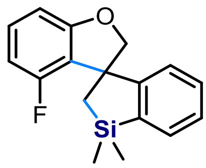

2ak

Following the general procedure, the reaction was carried out with **1ak** (85.2 mg, 0.2 mmol), [Pd]-1 (5.5 mg, 0.01 mmol), AgOAc (66.8 mg, 0.4 mmol), LiO^{t}Bu (48 mg, 0.6 mmol), and TcCu (14.7 mg, 0.2 mmol) in PhMe (0.5 mL) at 125 °C for 12 h. The title compound was obtained as a white solid; (40.5 mg, 0.142 mmol, 71% yield). R$_f$ (petroleum ether/dichloromethane = 20:1) = 0.3. **^{1}H NMR (400 MHz, CDCl$_3$)** δ 7.56 (m, 1H), 7.32 (m, 2H), 7.04 (d, *J* = 7.6 Hz, 1H), 6.83 (m, 1H), 6.68 – 6.50 (m, 2H), 4.48 (d, *J* = 8.5 Hz, 1H), 4.44 (d, *J* = 8.5 Hz, 1H), 1.52 (d, *J* = 15.1 Hz, 1H), 1.30 (d, *J* = 15.1 Hz, 1H), 0.43 (s, 3H), 0.35 (s, 3H). **^{13}C NMR (101 MHz, CDCl$_3$)** δ 163.73 (d, *J* = 243.3 Hz), 161.52 (d, *J* = 12.9 Hz), 156.28, 140.41, 134.99 (d, *J* = 2.7 Hz), 132.36, 131.08, 127.64, 126.06, 124.35 (d, *J* = 10.2 Hz), 108.51 (d, *J* = 22.8 Hz), 98.67 (d, *J* = 26.6 Hz), 88.82, 57.70, 29.57, -0.00, -0.80. **^{19}F NMR (376 MHz, CDCl$_3$)** δ -114.37. **HRMS (EI)** calcd. for $C_{17}H_{17}FOSi$ [M]$^+$: 284.1033. Found: 284.1030.

1',1',4,6-Tetramethyl-1',2'-dihydro-2*H*-spiro[benzofuran-3,3'-benzo[*b*]silole] (2al)

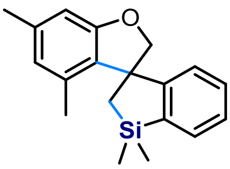

2al

Following the general procedure, the reaction was carried out with **1al** (87.2 mg, 0.2 mmol), [Pd]-1 (5.5 mg, 0.01 mmol), AgOAc (66.8 mg, 0.4 mmol), LiO^{t}Bu (48 mg, 0.6 mmol), and TcCu (14.7 mg, 0.1 mmol) in PhMe (0.5 mL) at 125 ℃ for 4 h. The title compound was obtained as a white solid (43 mg, 0.146 mmol, 73% yield). R$_f$ (petroleum ether/dichloromethane = 20:1) = 0.3. **^{1}H NMR (400 MHz, CDCl$_3$)** δ 7.58

– 7.53 (m, 1H), 7.37 – 7.28 (m, 2H), 7.12 (m, 1H), 6.57 (s, 1H), 6.49 (s, 1H), 4.36 (d, *J* = 8.4 Hz, 1H), 4.26 (d, *J* = 8.4 Hz, 1H), 2.31 (s, 3H), 1.74 (s, 3H), 1.51 (d, *J* = 15.2 Hz, 1H), 1.27 (d, *J* = 15.2 Hz, 1H), 0.39 (s, 3H), 0.35 (s, 3H). **^{13}C NMR (101 MHz, $CDCl_3$)** δ 160.71, 155.65, 140.18, 138.79, 133.99, 132.01, 131.70, 130.65, 126.98, 125.93, 124.52, 108.33, 87.96, 58.11, 26.70, 21.74, 18.26, 0.00, -1.23. **HRMS (EI)** calcd. For $C_{19}H_{22}OSi$ $[M]^+$: 294.1440. Found: 294.1443.

1',1',5,7-Tetramethyl-1',2'-dihydro-2*H*-spiro[benzofuran-3,3'-benzo[*b*]silole] (2am)

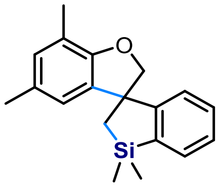

2am

Following the general procedure, the reaction was carried out with **1am** (87.2 mg, 0.2 mmol), [Pd]-1 (5.5 mg, 0.01 mmol), AgOAc (66.8 mg, 0.4 mmol), LiO^{t}Bu (48 mg, 0.6 mmol), and TcCu (7.7 mg, 0.04 mmol) in cyclohexane (0.5 mL) at 125 °C for 12 h. The title compound was obtained as a white solid (40.5 mg, 0.138 mmol, 69% yield). R$_f$ (petroleum ether/dichloromethane = 20:1) = 0.3. **^{1}H NMR (400 MHz, $CDCl_3$)** δ 7.61 – 7.49 (m, 1H), 7.40 – 7.22 (m, 2H), 7.08 (m, 1H), 6.81 (s, 1H), 6.56 (s, 1H), 4.42 (d, *J* = 8.4 Hz, 1H), 4.36 (d, *J* = 8.4 Hz, 1H), 2.26 (s, 3H), 2.21 (s, 3H), 1.51 (d, *J* = 15.1 Hz, 1H), 1.31 (d, *J* = 15.1 Hz, 1H), 0.42 (s, 3H), 0.35 (s, 3H). **^{13}C NMR (101 MHz, $CDCl_3$)** δ 157.47, 141.24, 138.29, 134.38, 132.94, 131.86, 131.66, 131.31, 128.16, 127.06, 122.47, 120.59, 88.30, 59.43, 29.96, 22.22, 16.56, 0.81, -0.00. **HRMS (EI)** calcd. For $C_{19}H_{22}OSi$ $[M]^+$: 294.1440. Found: 294.1429.

5,7-Difluoro-1',1'-dimethyl-1',2'-dihydro-2*H*-spiro[benzofuran-3,3'-benzo[*b*]-silole] (2an)

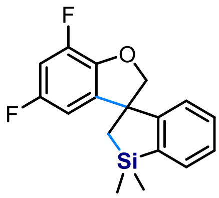

2an

Following the general procedure, the reaction was carried out with **1an** (87.2 mg, 0.2 mmol), [Pd]-1 (5.5 mg, 0.01 mmol), AgOAc (66.8 mg, 0.4 mmol), LiO^{t}Bu (48 mg, 0.6 mmol), and TcCu (7.7 mg, 0.04 mmol) in cyclohexane (0.5 mL) at 125 °C for 12 h. The title compound was obtained as a white solid (35.0 mg, 0.116 mmol, 58% yield). R_f (petroleum ether/dichloromethane = 20:1) = 0.2. **^{1}H NMR (400 MHz, CDCl$_3$)** δ 7.61 – 7.55 (m, 1H), 7.33 (m, 2H), 7.07 (m, 1H), 6.72 (m, 1H), 6.44 (m, 1H), 4.53 (d, *J* = 8.6 Hz, 1H), 4.48 (d, *J* = 8.6 Hz, 1H), 1.57 (d, *J* = 15.1 Hz, 1H), 1.29 (d, *J* = 15.1 Hz, 1H), 0.44 (s, 3H), 0.36 (s, 3H). **^{13}C NMR (101 MHz, CDCl$_3$)** δ 158.60 (dd, *J* = 241.1, 8.8 Hz), 148.00 (dd, *J* = 249.2, 12.6 Hz), 144.06 (dd, *J* = 10.7, 2.7 Hz), 143.05 (dd, *J* = 8.7, 3.6 Hz), 141.33, 133.28, 133.26, 132.02, 128.78, 126.86, 107.22 (dd, *J* = 24.3, 3.8 Hz), 104.79 (dd, *J* = 28.1, 20.9 Hz), 89.78, 59.95, 29.83, 0.79, -0.00. **^{19}F NMR (376 MHz, CDCl$_3$)** δ -119.48, -135.12. **HRMS (EI)** calcd. For $C_{17}H_{16}F_2OSi$ $[M]^+$: 302.0938. Found: 302.0935.

5,7-Difluoro-1',1'-dimethyl-1',2'-dihydro-2*H*-spiro[benzofuran-3,3'-benzo[*b*]-silole] (2ao)

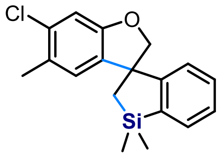

2ao

Following the general procedure, the reaction was carried out with **1ao** (91.2 mg, 0.2 mmol), [Pd]-1 (5.5 mg, 0.01 mmol), AgOAc (66.8 mg, 0.4 mmol), LiO^{t}Bu (48 mg, 0.6

mmol), and TcCu (7.7 mg, 0.04 mmol) in cyclohexane (0.5 mL) at 125 ℃ for 12 h. The title compound was obtained as a white solid (41 mg, 0.130 mmol, 65% yield). R_f (petroleum ether/dichloromethane = 20:1) = 0.3. **^{1}H NMR (400 MHz, CDCl$_3$)** δ 7.61 – 7.51 (m, 1H), 7.38 – 7.26 (m, 2H), 7.05 (m, 1H), 6.90 (s, 1H), 6.76 (s, 1H), 4.43 (d, *J* = 8.5 Hz, 1H), 4.39 (d, *J* = 8.5 Hz, 1H), 2.25 (s, 3H), 1.52 (d, *J* = 15.2 Hz, 1H), 1.30 (d, *J* = 15.2 Hz, 1H), 0.44 (s, 3H), 0.35 (s, 3H). **^{13}C NMR (101 MHz, CDCl$_3$)** δ 159.24, 156.05, 140.47, 137.20, 133.66, 132.34, 131.07, 129.06, 127.65, 126.14, 125.65, 111.01, 88.26, 58.04, 29.36, 20.33, 0.01, -0.80. **HRMS (EI)** calcd. For $C_{18}H_{19}ClOSi$ $[M]^+$: 314.0894. Found: 314.0893.

1,1-Dimethyl-1,2-dihydro-2'*H*-spiro[benzo[b]silole-3,3'-naphtho[2,3-*b*]furan] (2ap)

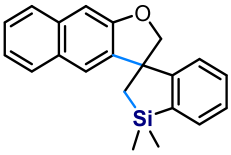

2ap

Following the general procedure, the reaction was carried out with **1ap** (91.2 mg, 0.2 mmol), [Pd]-1 (5.5 mg, 0.01 mmol), AgOAc (66.8 mg, 0.4 mmol), LiO^{t}Bu (48 mg, 0.6 mmol), and TcCu (7.7 mg, 0.04 mmol) in cyclohexane (0.5 mL) at 125 °C for 12 h. The title compound was obtained as a white solid (50 mg, 0.158 mmol, 79% yield). R_f (petroleum ether/dichloromethane = 20:1) = 0.3. **^{1}H NMR (400 MHz, CDCl$_3$)** δ 7.74 (d, *J* = 8.2 Hz, 1H), 7.65 (d, *J* = 8.2 Hz, 1H), 7.62 – 7.57 (m, 1H), 7.43 – 7.34 (m, 2H), 7.32 – 7.28 (m, 2H), 7.26 (m, 1H), 7.21 (s, 1H), 7.13 – 7.05 (m, 1H), 4.51 (d, *J* = 8.4 Hz, 1H), 4.47 (d, *J* = 8.4 Hz, 1H), 1.64 (d, *J* = 15.1 Hz, 1H), 1.42 (d, *J* = 15.1 Hz, 1H), 0.48 (s, 3H), 0.39 (s, 3H). **^{13}C NMR (101 MHz, CDCl$_3$)** δ 160.03, 156.74, 142.20, 141.37, 135.75, 133.05, 131.75, 131.46, 129.19, 128.37, 128.18, 127.13, 127.02, 124.60, 123.45, 105.33, 88.51, 58.67, 30.56, 0.76, 0.00. **HRMS (EI)** calcd. For $C_{21}H_{20}OSi$ $[M]^+$: 316.1283. Found: 316.1275.

1,1-dimethyl-1,2-dihydro-2'*H*-spiro[benzo[*b*]silole-3,3'-thieno[3,2-b]furan] (2aq)

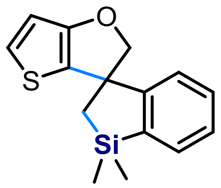

2aq

Following the general procedure, the reaction was carried out with **1aq** (82.8 mg, 0.2 mmol), [Pd]-1 (5.5 mg, 0.01 mmol), AgOAc (66.8 mg, 0.4 mmol), LiO^{t}Bu (48 mg, 0.6 mmol), and TcCu (14.7 mg, 0.1 mmol) in PhMe (0.5 mL) at 125 °C for 12 h. The title compound was obtained as a white solid (38 mg, 0.140 mmol, 70% yield). R_f (petroleum ether/dichloromethane = 20:1) = 0.3. **^{1}H NMR (400 MHz, CDCl$_3$)** δ 7.57 – 7.50 (m, 1H), 7.37 (m, 1H), 7.31 – 7.28 (m, 1H), 7.27 (m, 1H), 7.10 (d, *J* = 5.1 Hz, 1H), 6.63 (d, *J* = 5.1 Hz, 1H), 4.76 (s, 2H), 1.55 (d, *J* = 15.1 Hz, 1H), 1.42 (d, *J* = 15.1 Hz, 1H), 0.41 (s, 3H), 0.34 (s, 3H). **^{13}C NMR (101 MHz, CDCl3)** δ162.54, 156.71, 139.88, 132.52, 131.39, 128.85, 128.08, 127.97, 125.95, 112.59, 94.18, 58.52, 29.03, -0.00, -0.69. **HRMS (EI)** calcd. For $C_{15}H_{16}OSSi$ [M]$^+$: 272.0691. Found: 272.0686.

6'-Fluoro-1',1'-dimethyl-1',2'-dihydro-2*H*-spiro[benzofuran-3,3'-benzo[*b*]silole] (2ba)

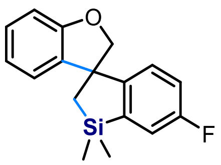

2ba

Following the general procedure, the reaction was carried out with **1ba** (85.2 mg, 0.2 mmol), [Pd]-1 (5.5 mg, 0.01 mmol), AgOAc (66.8 mg, 0.4 mmol), LiO^{t}Bu (48 mg, 0.6 mmol), and TcCu (7.7 mg, 0.04 mmol) in cyclohexane (0.5 mL) at 125 °C for 12 h. The title compound was obtained as a white solid (49.5 mg, 0.174 mmol, 87% yield). R_f (petroleum ether/dichloromethane = 20:1) = 0.3. **^{1}H NMR (400 MHz, CDCl$_3$)** δ 7.22 – 7.13 (m, 2H), 6.99 (m, 2H), 6.93 – 6.86 (m, 3H), 4.43 (d, *J* = 8.5 Hz, 1H), 4.35 (d, *J*

= 8.5 Hz, 1H), 1.55 (d, *J* = 15.2 Hz, 1H), 1.37 (d, *J* = 15.2 Hz, 1H), 0.44 (s, 3H), 0.36 (s, 3H). **^{13}C NMR (101 MHz, CDCl$_3$)** δ 163.78 (d, *J* = 247.8 Hz), 161.26, 152.90, 144.12 (d, *J* = 4.9 Hz)., 138.96, 129.80, 128.80 (d, *J* = 7.5 Hz), 124.80, 122.94, 119.10 (d, *J* = 22.5 Hz), 118.84 (d, *J* = 18.9 Hz), 111.33, 88.51, 58.60, 30.59, 0.77, 0.00. **^{19}F NMR (376 MHz, CDCl$_3$)** δ -116.92. **HRMS (EI)** calcd. For $C_{17}H_{17}FOSi$ [M]$^+$: 284.1033. Found: 284.1028.

1',1',6'-Trimethyl-1',2'-dihydro-2*H*-spiro[benzofuran-3,3'-benzo[*b*]silole] (2bb)

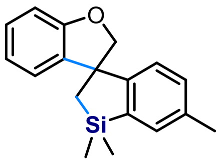

2bb

Following the general procedure, the reaction was carried out with **1bb** (84.4 mg, 0.2 mmol), [Pd]-1 (5.5 mg, 0.01 mmol), AgOAc (66.8 mg, 0.4 mmol), LiO^{t}Bu (48 mg, 0.6 mmol), and TcCu (14.7 mg, 0.04 mmol) in PhMe (0.5 mL) at 125 °C for 12 h. The title compound was obtained as a white solid (51.5 mg, 0.184 mmol, 92% yield). R$_f$ (petroleum ether/dichloromethane = 20:1) = 0.3. **^{1}H NMR (400 MHz, CDCl$_3$)** δ 7.39 – 7.34 (m, 1H), 7.16 (m, 2H), 6.99 – 6.80 (m, 4H), 4.43 (d, *J* = 8.4 Hz, 1H), 4.38 (d, *J* = 8.4 Hz, 1H), 2.36 (s, 3H), 1.52 (d, *J* = 15.1 Hz, 1H), 1.33 (d, *J* = 15.1 Hz, 1H), 0.43 (s, 3H), 0.35 (s, 3H). **^{13}C NMR (101 MHz, CDCl3)** δ160.32, 153.67, 140.49, 138.34, 136.95, 132.66, 131.97, 128.57, 125.96, 123.91, 121.83, 110.19, 87.71, 57.82, 29.50, 21.67, -0.00, -0.81. **HRMS (EI)** calcd. For $C_{18}H_{20}OSi$ [M]$^+$: 280.1283. Found: 280.1278.

1',1',6'-Trimethyl-1',2'-dihydro-2*H*-spiro[benzofuran-3,3'-benzo[*b*]silole] (2bc)

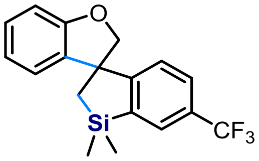

2bc

Following the general procedure, the reaction was carried out with **1bc** (95.2 mg, 0.2 mmol), [Pd]-1 (5.5 mg, 0.01 mmol), AgOAc (66.8 mg, 0.4 mmol), LiO^{t}Bu (48 mg, 0.6 mmol), and TcCu (7.7 mg, 0.04 mmol) in cyclohexane (0.5 mL) at 125 °C for 12 h. The title compound was obtained as a white solid (48 mg, 0.144 mmol, 72% yield). R$_f$ (petroleum ether/dichloromethane = 20:1) = 0.3. **^{1}H NMR (400 MHz, CDCl$_3$)** δ 7.91 – 7.72 (m, 1H), 7.54 (m, 1H), 7.20 (m, 1H), 7.14 (m, 1H), 6.97 – 6.86 (m, 3H), 4.45 (d, *J* = 8.6 Hz, 1H), 4.38 (d, *J* = 8.6 Hz, 1H), 1.57 (d, *J* = 15.3 Hz, 1H), 1.41 (d, *J* = 15.3 Hz, 1H), 0.48 (s, 3H), 0.39 (s, 3H). **^{13}C NMR (101 MHz, CDCl$_3$)** δ160.61, 160.52, 141.57, 137.61, 129.97 (q, *J* = 31.7 Hz), 129.27, 129.22 (q, *J* = 3.5 Hz), 128.09 (q, *J* = 3.4 Hz), 126.63, 125.27 (q, *J* = 273.7 Hz), 124.05, 122.29, 110.68, 87.45, 58.43, 29.24, -0.00, -0.77. **^{19}F NMR (376 MHz, CDCl$_3$)** δ -62.17. **HRMS (EI)** calcd. For $C_{18}H_{17}F_3OSi$ [M]$^+$: 334.1001. Found: 334.0084.

5'-Fluoro-1',1'-dimethyl-1',2'-dihydro-2*H*-spiro[benzofuran-3,3'-benzo[*b*]silole] (2bd)

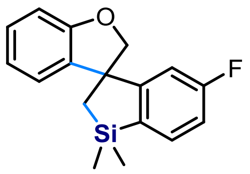

2bd

Following the general procedure, the reaction was carried out with **1bd** (85.2 mg, 0.2 mmol), [Pd]-1(5.5 mg, 0.01 mmol), AgOAc (66.8 mg, 0.4 mmol), LiO^{t}Bu (48 mg, 0.6 mmol), and TcCu (7.7 mg, 0.04 mmol) in cyclohexane (0.5 mL) at 125 °C for 12 h. The title compound was obtained as a white solid (35 mg, 0.122 mmol, 61% yield). R$_f$ (petroleum ether/dichloromethane = 20:1) = 0.3. **^{1}H NMR (400 MHz, CDCl$_3$)** δ 7.50 (m, 1H), 7.22 – 7.15 (m, 1H), 7.02 – 6.88 (m, 4H), 6.72 (m, 1H), 4.44 (d, *J* = 8.5 Hz, 1H), 4.38 (d, *J* = 8.5 Hz, 1H), 1.54 (d, *J* = 15.2 Hz, 1H), 1.39 (d, *J* = 15.2 Hz, 1H), 0.43 (s, 3H), 0.35 (s, 3H). **^{13}C NMR (101 MHz, CDCl$_3$)** δ 164.96 (d, *J* = 248.6 Hz), 159.72, 159.02, 136.85, 134.94 (d, *J* = 3.0 Hz), 133.26 (d, *J* = 8.5 Hz), 128.38, 123.32, 121.42, 114.60 (d, *J* = 21.1 Hz), 112.40 (d, *J* = 20.6 Hz), 109.83, 86.72, 57.53, 28.87, -0.55, -

1.32. **^{19}F NMR (376 MHz, CDCl$_3$)** δ -110.72. **HRMS (EI)** calcd. For $C_{17}H_{17}FOSi$ $[M]^+$: 284.1033. Found: 284.1025.

5'-Chloro-1',1'-dimethyl-1',2'-dihydro-2*H*-spiro[benzofuran-3,3'-benzo[*b*]silole] (2be)

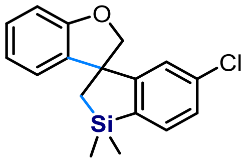

2be

Following the general procedure, the reaction was carried out with **1be** (88.4 mg, 0.2 mmol), [Pd]-1 (5.5 mg, 0.01 mmol), AgOAc (66.8 mg, 0.4 mmol), LiO^{t}Bu (48 mg, 0.6 mmol), and TcCu (14.7 mg, 0.1 mmol) in PhMe (0.5 mL) at 125 °C for 12 h. The title compound was obtained as a white solid (52 mg, 0.174 mmol, 87% yield). R_f (petroleum ether/dichloromethane = 20:1) = 0.3. **^{1}H NMR (400 MHz, CDCl$_3$)** δ 7.47 (m, 1H), 7.26 – 7.23 (m, 1H), 7.20 (m, 1H), 7.02 (m, 1H), 6.91 (m, 3H), 4.43 (d, J = 8.6 Hz, 1H), 4.38 (d, J = 8.6 Hz, 1H), 1.54 (d, J = 15.2 Hz, 1H), 1.37 (d, J = 15.2 Hz, 1H), 0.44 (s, 3H), 0.36 (s, 3H). **^{13}C NMR (101 MHz, CDCl$_3$)** δ 160.41, 158.77, 138.69, 137.46, 137.40, 133.51, 129.08, 128.02, 126.35, 124.00, 122.15, 110.54, 87.42, 58.26, 29.46, -0.00, -0.77. **HRMS (EI)** calcd. For $C_{17}H_{17}FOSi$ $[M]^+$: 300.0737. Found: 300.0733.

1',1',5'-Trimethyl-1',2'-dihydro-2*H*-spiro[benzofuran-3,3'-benzo[*b*]silole] (2bf)

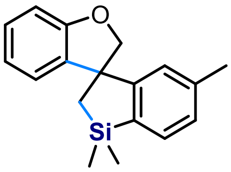

2bf

Following the general procedure, the reaction was carried out with **1bf** (84.4 mg, 0.2 mmol), [Pd]-1 (5.5 mg, 0.01 mmol), AgOAc (66.8 mg, 0.4 mmol), LiO^{t}Bu (48 mg, 0.6 mmol), and TcCu (14.7 mg, 0.1 mmol) in PhMe (0.5 mL) at 125 °C for 12 h. The title

compound was obtained as a white solid (34 mg, 0.122 mmol, 61% yield). R_f (petroleum ether/dichloromethane = 20:1) = 0.3. **^{1}H NMR (400 MHz, $CDCl_3$)** δ 7.46 (m, 1H), 7.22 – 7.15 (m, 1H), 7.11 (m, 1H), 6.98 – 6.84 (m, 4H), 4.43 (d, *J* = 8.5 Hz, 1H), 4.40 (d, *J* = 8.5 Hz, 1H), 2.27 (s, 3H), 1.52 (d, *J* = 15.1 Hz, 1H), 1.33 (d, *J* = 15.1 Hz, 1H), 0.42 (s, 3H), 0.34 (s, 3H). **^{13}C NMR (101 MHz, $CDCl_3$)** δ 159.77, 156.28, 140.49, 137.69, 136.36, 131.54, 128.08, 128.00, 126.11, 123.44, 121.26, 109.62, 87.14, 57.52, 29.01, 21.62, -0.50, -1.29. **HRMS (EI)** calcd. For $C_{18}H_{20}OSi$ $[M]^+$: 280.1283. Found: 280.1277.

5'-Methoxy-1',1'-dimethyl-1',2'-dihydro-2*H*-spiro[benzofuran-3,3'-benzo[*b*]silole] (2bg)

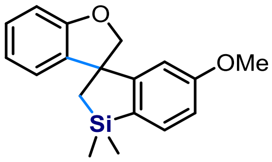

2bg

Following the general procedure, the reaction was carried out with **1bg** (87.6 mg, 0.2 mmol), [Pd]-1 (5.5 mg, 0.01 mmol), AgOAc (66.8 mg, 0.4 mmol), LiO^{t}Bu (48 mg, 0.6 mmol), and TcCu (7.7 mg, 0.04 mmol) in cyclohexane (0.5 mL) at 125 °C for 12 h. The title compound was obtained as a white solid (35.5 mg, 0.120 mmol, 60% yield). R_f (petroleum ether/dichloromethane = 20:1) = 0.3. **^{1}H NMR (400 MHz, $CDCl_3$)** δ 7.47 (m, 1H), 7.21 – 7.13 (m, 1H), 7.01 – 6.76 (m, 4H), 6.57 (m, 1H), 4.44 (d, *J* = 8.5 Hz, 1H), 4.40 (d, *J* = 8.5 Hz, 1H), 3.71 (s, 3H), 1.53 (d, *J* = 15.1 Hz, 1H), 1.34 (d, *J* = 15.1 Hz, 1H), 0.41 (s, 3H), 0.34 (s, 3H). **^{13}C NMR (101 MHz, $CDCl_3$)** δ 162.20, 160.13, 158.57, 137.72, 133.11, 131.20, 128.45, 123.78, 121.66, 114.03, 110.91, 110.01, 87.31, 58.04, 55.51, 29.54, 0.00, -0.81. **HRMS (EI)** calcd. For $C_{18}H_{20}O2Si$ $[M]^+$: 296.1233. Found: 296.1220.

5',6'-Dimethoxy-1',1'-dimethyl-1',2'-dihydro-2*H*-spiro[benzofuran-3,3'-benzo[*b*]-silole] (2bh)

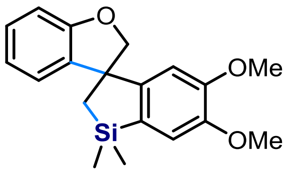

2bh

Following the general procedure, the reaction was carried out with **1bh** (93.6 mg, 0.2 mmol), [Pd]-1 (5.5 mg, 0.01 mmol), AgOAc (66.8 mg, 0.4 mmol), LiO^{t}Bu (48 mg, 0.6 mmol), and TcCu (7.7 mg, 0.04 mmol) in cyclohexane (0.5 mL) at 125 °C for 12 h. The title compound was obtained as a white solid (28 mg, 0.086mmol, 43% yield). R_f (petroleum ether/dichloromethane = 20:1) = 0.3. **^{1}H NMR (400 MHz, CDCl$_3$)** δ 7.17 (m, 1H), 6.98 – 6.92 (m, 2H), 6.89 (m, 2H), 6.55 (s, 1H), 4.41 (d, *J* = 8.5 Hz, 1H), 4.39 (d, *J* = 8.5 Hz, 1H), 3.92 (s, 3H), 3.71 (s, 3H), 1.53 (d, *J* = 15.1 Hz, 1H), 1.30 (d, *J* = 15.1 Hz, 1H), 0.42 (s, 3H), 0.35 (s, 3H). **^{13}C NMR (101 MHz, CDCl$_3$)** δ 160.75, 152.40, 149.82, 149.72, 138.75, 131.92, 129.12, 124.39, 122.41, 113.58, 110.73, 109.26, 87.97, 58.57, 57.02, 56.87, 30.33, 0.74, 0.00. **HRMS (EI)** calcd. For $C_{19}H_{22}O_3Si$ $[M]^+$: 326.1338. Found: 326.1326.

5',6'-Dimethoxy-1',1'-dimethyl-1',2'-dihydro-2*H*-spiro[benzofuran-3,3'-benzo[*b*]-silole] (2bi)

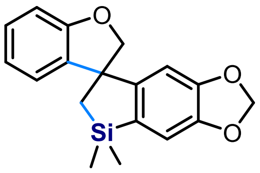

2bi

Following the general procedure, the reaction was carried out with **1bi** (90.4 mg, 0.2 mmol), [Pd]-1 (5.5 mg, 0.01 mmol), AgOAc (66.8 mg, 0.4 mmol), LiO^{t}Bu (48 mg, 0.6 mmol), and TcCu (7.7 mg, 0.04 mmol) in cyclohexane (0.5 mL) at 125 °C for 4 h. The title compound was obtained as a white solid (34.1 mg, 0.11 mmol, 55% yield). R_f

(petroleum ether/dichloromethane = 20:1) = 0.3. **^{1}H NMR (400 MHz, $CDCl_3$)** δ 7.21 – 7.10 (m, 1H), 6.99 – 6.79 (m, 4H), 6.51 (s, 1H), 5.92 (s, 2H), 4.40 (d, *J* = 8.5 Hz, 1H), 4.36 (d, *J* = 8.5 Hz, 1H), 1.52 (d, *J* = 15.1 Hz, 1H), 1.34 (d, *J* = 15.1 Hz, 1H), 0.40 (s, 3H), 0.32 (s, 3H). **^{13}C NMR (101 MHz, $CDCl_3$)** δ 160.09, 150.81, 150.56, 147.77, 137.90, 132.60, 128.55, 123.72, 121.73, 110.15, 110.04, 106.71, 101.50, 87.29, 57.80, 29.57, 0.00, -0.77. **HRMS (EI)** calcd. For $C_{18}H_{18}O_3Si$ $[M]^+$: 310.1025. Found: 310.1018.

1',1',7'-Trimethyl-1',2'-dihydro-2H-spiro[benzofuran-3,3'-benzo[*b*]silole] (2bj)

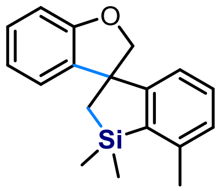

2bj

Following the general procedure, the reaction was carried out with **1bj** (84.4 mg, 0.2 mmol), [Pd]-1 (5.5 mg, 0.01 mmol), AgOAc (66.8 mg, 0.4 mmol), LiO^{t}Bu (48 mg, 0.6 mmol), and TcCu (7.7 mg, 0.04 mmol) in cyclohexane (0.5 mL) at 125 °C for 4 h. The title compound was obtained as a white solid (37.5 mg, 0.134 mmol, 67% yield). R$_f$ (petroleum ether/dichloromethane = 20:1) = 0.3. **^{1}H NMR (400 MHz, $CDCl_3$)** δ 7.38 (s, 1H), 7.16 (m, 2H), 7.03 – 6.84 (m, 4H), 4.42 (m, 1H), 4.38 (m, 1H), 2.36 (s, 3H), 1.53 (d, *J* = 15.1 Hz, 1H), 1.33 (d, *J* = 15.1 Hz, 1H), 0.43 (s, 3H), 0.35 (s, 3H). **^{13}C NMR (101 MHz, $CDCl_3$)** δ 160.32, 153.67, 140.49, 138.34, 136.96, 132.66, 131.97, 128.57, 125.96, 123.91, 121.83, 110.19, 87.72, 57.82, 29.50, 21.67, -0.00, -0.81. **HRMS (EI)** calcd. For $C_{18}H_{20}OSi$ $[M]^+$: 280.1283. Found: 280.1277.

1,1-Dimethyl-1,2-dihydrospiro[benzo[*b*]silole-3,4'-isochromane] (2ca)

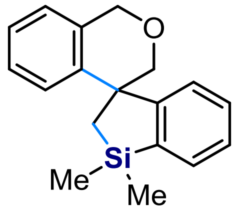

2ca

Following the general procedure, the reaction was carried out with **1ca** (84.4 mg, 0.2 mmol), [Pd]-1 (5.5 mg, 0.01 mmol), AgOAc (66.8 mg, 0.4 mmol), LiO^{t}Bu (48 mg, 0.6 mmol), and TcCu (38.2 mg, 0.2 mmol) in cyclohexane (0.5 mL) at 125 °C for 12 h. The title compound was obtained as a white solid (25.2 mg, 0.090 mmol, 45% yield). R_f (petroleum ether/dichloromethane = 20:1) = 0.3. **^{1}H NMR (400 MHz, $CDCl_3$)** δ 7.63 – 7.55 (m, 1H), 7.33 – 7.21 (m, 2H), 7.21 – 7.08 (m, 2H), 7.04 – 6.99 (m, 1H), 6.96 – 6.89 (m, 1H), 6.85 (m, 1H), 4.94 (m, 2H), 3.72 (m, 2H), 1.84 (d, *J* = 15.6 Hz, 1H), 1.16 (d, *J* = 15.6 Hz, 1H), 0.41(s, 3H), 0.38 (s, 3H). **^{13}C NMR (101 MHz, $CDCl_3$)** δ 157.04, 145.42, 142.16, 134.56, 132.62, 130.50, 129.01, 127.89, 127.72, 127.53, 126.62, 124.48, 78.70, 69.70, 52.33, 29.16, 0.75, 0.00. **HRMS (EI)** calcd. For $C_{18}H_{20}OSi$ $[M]^+$: 280.1283. Found: 280.1281.

1,1-dimethyl-1'-tosyl-1,2-dihydrospiro[benzo[*b*]silole-3,3'-indoline] (2da)

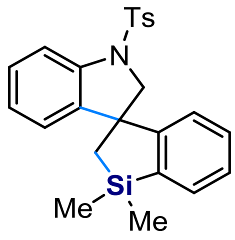

2da

Following the general procedure, the reaction was carried out with **1da** (112.2 mg, 0.2 mmol), [Pd]-1 (5.5 mg, 0.01 mmol), AgOAc (66.8 mg, 0.4 mmol), LiO^{t}Bu (48 mg, 0.6 mmol), and TcCu (38.2 mg, 0.2 mmol) in cyclohexane (0.5 mL) at 125 °C for 12 h. The title compound was obtained as a white solid (25.2 mg, 0.090 mmol, 47% yield). R_f

(petroleum ether/ethyl acetate = 10:1) = 0.5. **^{1}H NMR (400 MHz, $CDCl_3$)** δ 7.73 (m, 3H), 7.58 – 7.46 (m, 1H), 7.25 – 7.20 (m, 4H), 7.14 (m, 1H), 6.98 (m, 1H), 6.79 (m, 1H), 6.53 (m, 1H), 3.93 (d, J = 10.2 Hz, 1H), 3.73 (d, J = 10.2 Hz, 1H), 2.40 (s, 3H), 1.27 (d, J = 15.2 Hz, 1H), 1.10 (d, J = 15.2 Hz, 1H), 0.37 (s, 3H), 0.37 (s, 3H). **^{13}C NMR (101 MHz, $CDCl_3$)** δ 157.37, 145.64, 143.20, 142.96, 141.48, 135.48, 133.18, 131.77, 131.21, 129.45, 129.03, 128.47, 126.96, 125.74, 125.39, 115.71, 68.22, 57.10, 31.33, 23.10, 0.94, 0.00. **HRMS (EI)** calcd. For $C_{24}H_{25}NO_2Si$ $[M]^+$: 419.1375. Found: 419.1369.

1,1-Dimethyl-1'-(methylsulfonyl)-1,2-dihydrospiro[benzo[*b*]silole-3,3'-indoline] (2db)

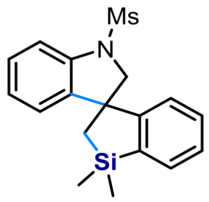

2db

Following the general procedure, the reaction was carried out with **1db** (89.8 mg, 0.2 mmol), [Pd]-1 (5.5 mg, 0.01 mmol), AgOAc (66.8 mg, 0.4 mmol), LiO^{t}Bu (48 mg, 0.6 mmol), and TcCu (7.7 mg, 0.04 mmol) in cyclohexane (0.5 mL) at 125 °C for 12 h. The title compound was obtained as a white solid (34.3 mg, 0.100 mmol, 50% yield). R_f (petroleum ether/ethyl acetate = 10:1) = 0.5. **^{1}H NMR (400 MHz, $CDCl_3$)** δ 7.58 (m, 1H), 7.49 (m, 1H), 7.31 (m, 2H), 7.28 – 7.22 (m, 1H), 7.05 (m, 1H), 7.00 – 6.89 (m, 2H), 3.95 (d, J = 9.9 Hz, 1H), 3.82 (d, J = 9.9 Hz, 1H), 2.94 (s, 3H), 1.58 (d, J = 15.1 Hz, 1H), 1.27 (d, J = 15.1 Hz, 1H), 0.43 (s, 3H), 0.39 (s, 3H). **^{13}C NMR (101 MHz, $CDCl_3$)** δ 156.01, 142.07, 141.92, 140.69, 132.51, 130.99, 128.81, 127.83, 125.98, 124.91, 124.71, 113.70, 67.84, 56.21, 35.13, 30.03, -0.00, -0.86. **HRMS (EI)** calcd. For $C_{18}H_{21}NO_2SSi$ $[M]^+$: 343.1062. Found: 343.1055.

1-(1,1-Dimethyl-1,2-dihydrospiro[benzo[*b*]silole-3,3'-indolin]-1'-yl)ethan-1-one (2dc)

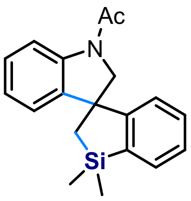

2dc

Following the general procedure, the reaction was carried out with **1dc** (89.8 mg, 0.2 mmol), [Pd]-1 (5.5 mg, 0.01 mmol), AgOAc (66.8 mg, 0.4 mmol), LiO^{t}Bu (48 mg, 0.6 mmol), and TcCu (38.2 mg, 0.2 mmol) in cyclohexane (0.5 mL) at 125 °C for 12 h. The title compound was obtained as a white solid (31.3 mg, 0.102 mmol, 51% yield). R_f (petroleum ether/ethyl acetate = 10:1) = 0.5. **^{1}H NMR (400 MHz, $CDCl_3$)** δ 8.27 (m, 1H), 7.64 – 7.50 (m, 1H), 7.36 – 7.26 (m, 2H), 7.25 – 7.22 (m, 1H), 7.04 (m, 1H), 6.92 (m, 2H), 4.01 (d, *J* = 10.1 Hz, 1H), 3.99 (d, *J* = 10.1 Hz, 1H), 2.18 (s, 3H), 1.48 (d, *J* = 15.2 Hz, 1H), 1.38 (d, *J* = 15.2 Hz, 1H), 0.44 (s, 3H), 0.40 (s, 3H). **^{13}C NMR (101 MHz, $CDCl_3$)** δ 170.03, 157.88, 143.73, 142.96, 140.95, 133.22, 131.99, 129.28, 128.52, 126.85, 125.88, 124.63, 118.26, 68.37, 57.14, 31.49, 25.68, 1.00, 0.00. **HRMS (EI)** calcd. For $C_{19}H_{21}NOSi$ $[M]^+$: 307.1392. Found: 307.1383.

5'-Fluoro-1,1-dimethyl-1'-tosyl-1,2-dihydrospiro[benzo[*b*]silole-3,3'-indoline] (2dd)

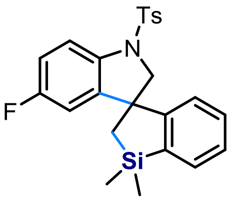

2dd

Following the general procedure, the reaction was carried out with **1dd** (115.8 mg, 0.2 mmol), [Pd]-1 (5.5 mg, 0.01 mmol), AgOAc (66.8 mg, 0.4 mmol), LiO^{t}Bu (48 mg, 0.6 mmol), and TcCu (7.64 mg, 0.04 mmol) in cyclohexane (0.5 mL) at 125 °C for 12 h. The title compound was obtained as a white solid (50.7 mg, 0.116 mmol, 58% yield).

R$_f$ (petroleum ether/ethyl acetate = 10:1) = 0.4. **^{1}H NMR (400 MHz, $CDCl_3$)** δ 7.68 (m, 3H), 7.56 – 7.49 (m, 1H), 7.25 (m, 3H), 7.15 (m, 1H), 6.92 (m, 1H), 6.57 – 6.43 (m, 2H), 3.92 (d, *J* = 10.4 Hz, 1H), 3.73 (d, *J* = 10.4 Hz, 1H), 2.42 (s, 3H), 1.22 (d, *J* = 15.1 Hz, 1H), 1.02 (d, *J* = 15.1 Hz, 1H), 0.36 (s, 3H), 0.35 (s, 3H). **^{13}C NMR (101 MHz, $CDCl_3$)** δ 160.78 (d, *J* = 242.6 Hz), 155.71, 144.93, 144.44 (d, *J* = 7.5 Hz), 140.60, 138.12, 134.25, 132.38, 130.99, 130.36, 128.15, 127.80, 125.94, 115.98(d, *J* = 8.6 Hz), 115.19 (d, *J* = 23.6 Hz), 111.62 (d, *J* = 24.0 Hz), 67.61, 56.27, 30.40, 22.21, 0.00, -0.95. **^{19}F NMR (376 MHz, $CDCl_3$)** δ -118.63. **HRMS (EI)** calcd. For $C_{24}H_{24}FNO_2SSi$ $[M]^+$: 437.1281. Found: 437.1273.

5'-Fluoro-1,1-dimethyl-1'-tosyl-1,2-dihydrospiro[benzo[*b*]silole-3,3'-indoline] (2de)

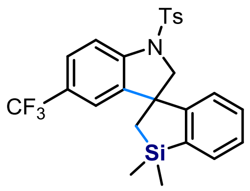

2de

Following the general procedure, the reaction was carried out with **1de** (125.8 mg, 0.2 mmol), [Pd]-1 (5.5 mg, 0.01 mmol), AgOAc (66.8 mg, 0.4 mmol), LiO^{t}Bu (48 mg, 0.6 mmol), and TcCu (14.7 mg, 0.1 mmol) in PhMe (0.5 mL) at 125 °C for 12 h. The title compound was obtained as a white solid (39.0 mg, 0.080 mmol, 40% yield). R$_f$ (petroleum ether/ethyl acetate = 10:1) = 0.4. **^{1}H NMR (400 MHz, $CDCl_3$)** δ 7.80 (m, 1H), 7.73 (m, 2H), 7.53 (m, 2H), 7.33 – 7.24 (m, 3H), 7.18 (m, 1H), 7.05 (m, 1H), 6.53 (m, 1H), 3.98 (d, *J* = 10.2, 1H), 3.78 (d, *J* = 10.2 Hz, 1H), 2.42 (s, 3H), 1.33 (d, *J* = 15.1, 1H), 1.10 (d, *J* = 15.1 Hz, 1H), 0.40 (s, 3H), 0.38 (s, 3H). **^{13}C NMR (101 MHz, $CDCl_3$)** δ 156.42, 146.30, 146.00, 143.89, 141.67, 135.35, 133.54, 132.13, 131.54, 129.01, 128.96, 127.90 (q, *J* = 32.6 Hz), 127.24 (q, *J* = 3.7 Hz), 126.87, 125.85 (q, *J* = 273.1 Hz), 122.71 (q, *J* = 3.3 Hz), 115.41, 68.51, 57.02, 31.59, 23.24, 1.03, 0.00. **^{19}F NMR (376 MHz, $CDCl_3$)** δ -63.85. **HRMS (EI)** calcd. For $C_{25}H_{24}F_3NO_2SSi$ $[M]^+$: 487.1249. Found: 487.1223.

1,1,5'-Trimethyl-1'-tosyl-1,2-dihydrospiro[benzo[*b*]silole-3,3'-indoline] (2df)

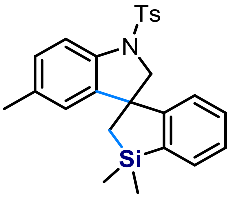

2df

Following the general procedure, the reaction was carried out with **1df** (120.2 mg, 0.2 mmol), [Pd]-1 (5.5 mg, 0.01 mmol), AgOAc (66.8 mg, 0.4 mmol), LiO^{t}Bu (48 mg, 0.6 mmol), and TcCu (7.64 mg, 0.04 mmol) in cyclohexane (0.5 mL) at 125 °C for 12 h. The title compound was obtained as a white solid (55.4 mg, 0.128 mmol, 64% yield). R_f (petroleum ether/ethyl acetate = 10:1) = 0.5. **^{1}H NMR (400 MHz, CDCl$_3$)** δ 7.70 (m, 2H), 7.61 (m, 1H), 7.51 (m, 1H), 7.21 (m, 3H), 7.14 (m, 1H), 7.03 (m, 1H), 6.59 (s, 1H), 6.52 (m, 1H), 3.89 (d, *J* = 10.2 Hz, 1H), 3.69 (d, *J* = 10.2 Hz, 1H), 2.40 (s, 3H), 2.21 (s, 3H), 1.23 (d, *J* = 15.2 Hz, 1H), 1.07 (d, *J* = 15.2 Hz, 1H), 0.36 (s, 3H), 0.35 (s, 3H). **^{13}C NMR (101 MHz, CDCl$_3$)** δ 157.48, 145.53, 143.26, 141.51, 140.70, 135.48, 135.35, 133.15, 131.74, 131.18, 130.09, 129.08, 128.43, 127.04, 125.89, 115.57, 68.34, 57.11, 31.28, 23.12, 22.51, 0.95, 0.00. **HRMS (EI)** calcd. For $C_{25}H_{27}NO_2SSi$ [M]$^+$: 433.1532. Found: 433.1523.

5'-Methoxy-1,1-dimethyl-1'-tosyl-1,2-dihydrospiro[benzo[*b*]silole-3,3'-indoline] (2dg)

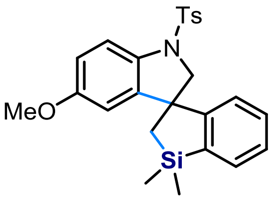

2dg

Following the general procedure, the reaction was carried out with **1dg** (118.2 mg, 0.2 mmol), [Pd]-1(5.5 mg, 0.01 mmol), AgOAc (66.8 mg, 0.4 mmol), LiO^{t}Bu (48 mg, 0.6 mmol), and TcCu (7.64 mg, 0.04 mmol) in cyclohexane (0.5 mL) at 125 °C for 12 h. The title compound was obtained as a white solid (62.9 mg, 0.140 mmol, 70% yield).

R$_f$ (petroleum ether/ethyl acetate = 10:1) = 0.5. **^{1}H NMR (400 MHz, CDCl$_3$)** δ 7.67 (m, 3H), 7.51 (m, 1H), 7.25 – 7.19 (m, 3H), 7.14 (m, 1H), 6.78 (m, 1H), 6.58 – 6.45 (m, 1H), 6.31 (m, 1H), 3.89 (d, J = 10.4 Hz, 1H), 3.70 (d, J = 10.4 Hz, 1H), 3.68 (s, 3H), 2.40 (s, 3H), 1.18 (d, J = 15.1 Hz, 1H), 1.04 (d, J = 15.1 Hz, 1H), 0.36 (s, 3H), 0.35 (s, 3H). **^{13}C NMR (101 MHz, CDCl$_3$)** δ 157.76, 156.28, 144.60, 143.83, 140.59, 135.68, 134.33, 132.25, 130.89, 130.24, 128.22, 127.57, 126.08, 115.98, 113.96, 109.92, 67.52, 56.50, 56.22, 30.34, 22.19, 0.00, -0.94. **HRMS (EI)** calcd. For $C_{25}H_{27}NO_3SSi$ [M]$^+$: 449.1481. Found: 449.1474.

6'-Chloro-1,1-dimethyl-1'-tosyl-1,2-dihydrospiro[benzo[*b*]silole-3,3'-indoline] (2dh)

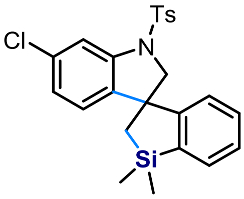

2dh

Following the general procedure, the reaction was carried out with **1dh** (119.0 mg, 0.2 mmol), [Pd]-1(5.5 mg, 0.01 mmol), AgOAc (66.8 mg, 0.4 mmol), LiO^{t}Bu (48 mg, 0.6 mmol), and TcCu (7.64 mg, 0.04 mmol) in cyclohexane (0.5 mL) at 125 °C for 12 h. The title compound was obtained as a white solid (47.1mg, 0.104 mmol, 52% yield). R$_f$ (petroleum ether/ethyl acetate = 10:1) = 0.5. **^{1}H NMR (400 MHz, CDCl$_3$)** δ 7.79 – 7.64 (m, 3H), 7.52 (m, 1H), 7.34 – 7.27 (m, 2H), 7.25 – 7.20 (m, 1H), 7.15 (m, 1H), 6.94 (m, 1H), 6.70 (m, 1H), 6.52 (m, 1H), 3.93 (d, J = 10.2 Hz, 1H), 3.73 (d, J = 10.2 Hz, 1H), 2.42 (s, 3H), 1.26 (d, J = 15.1 Hz, 1H), 1.05 (d, J = 15.1 Hz, 1H), 0.37 (s, 3H), 0.35 (s, 3H). **^{13}C NMR (101 MHz, CDCl$_3$)** δ 156.80, 146.03, 144.12, 141.80, 141.51, 135.29, 135.19, 133.32, 131.91, 131.41, 129.01, 128.70, 126.83, 126.25, 125.83, 115.97, 68.56, 56.76, 31.41, 23.17, 0.93, 0.00. **HRMS (EI)** calcd. For $C_{24}H_{24}ClNO_2SSi$ [M]$^+$: 453.0986. Found: 453.0979.

1'-Benzyl-1'-methyl-1',2'-dihydro-2*H*-spiro[benzofuran-3,3'-benzo[*b*]silole] (2ea)

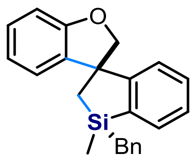

2ea

Following the general procedure, the reaction was carried out with **1ea** (96.8 mg, 0.2 mmol), [Pd]-1(5.5 mg, 0.01 mmol), AgOAc (66.8 mg, 0.4 mmol), LiO^{t}Bu (48 mg, 0.6 mmol), and TcCu (7.64 mg, 0.04 mmol) in cyclohexane (0.5 mL) at 125 °C for 12 h. The title compound was obtained as a white solid (26 mg, 0.076 mmol, 38% yield, dr = 1:1). R_f (petroleum ether/dichloromethane = 20:1) = 0.3. For the first diastereoisomer: **^{1}H NMR (400 MHz, CDCl$_3$)** δ7.45 (m, 1H), 7.32 (m, 1H), 7.25 – 7.17 (m, 3H), 7.17 – 7.07 (m, 2H), 7.03 (m, 3H), 6.88 (m, 1H), 6.84 (m, 1H), 6.64 (m, 1H), 4.39 (d, *J* = 8.7 Hz, 1H), 4.37 (d, *J* = 8.7 Hz, 1H), 2.52 (d, *J* = 13.9 Hz, 1H), 2.45 (d, *J* = 13.9 Hz, 1H), 1.46 (d, *J* = 15.4 Hz, 1H), 1.36 (d, *J* = 15.4 Hz, 1H), 0.35 (s, 3H). **^{13}C NMR (101 MHz, CDCl$_3$)** δ 159.63, 156.24, 138.68, 138.05, 137.66, 132.11, 130.62, 128.44, 128.35, 128.06, 126.84, 125.74, 124.53, 123.31, 121.32, 109.58, 87.45, 57.35, 26.63, 25.30, -2.53. For the second diastereoisomer: **^{1}H NMR (400 MHz, CDCl$_3$)** δ 7.50 – 7.44 (m, 1H), 7.36 – 7.26 (m, 2H), 7.19 (m, 2H), 7.16 – 7.12 (m, 1H), 7.12 – 7.06 (m, 1H), 6.98 (m, 1H), 6.94 (m, 2H), 6.89 – 6.81 (m, 3H), 3.98 (d, *J* = 8.7 Hz, 1H), 3.83 (d, *J* = 8.7 Hz, 1H), 2.45 (d, *J* = 13.8 Hz, 1H), 2.35 (d, *J* = 13.8 Hz, 1H), 1.54 (d, *J* = 15.5 Hz, 1H), 1.26 (d, *J* = 15.5 Hz, 1H), 0.41 (s, 3H). **^{13}C NMR (101 MHz, CDCl$_3$)** δ 159.70, 156.45, 138.80, 137.81, 137.79, 132.06, 130.63, 128.45, 128.27, 128.08, 126.87, 125.73, 124.64, 123.26, 121.27, 109.56, 86.87, 57.51, 26.48, 25.73, -2.93. **HRMS (EI)** calcd. For $C_{23}H_{22}OSi$ [M]$^+$: 342.1440. Found: 342.1435.

1'-Ethyl-1'-methyl-1',2'-dihydro-2*H*-spiro[benzofuran-3,3'-benzo[*b*]silole] (2eb)

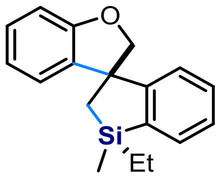

2eb

Following the general procedure, the reaction was carried out with **1eb** (84.4 mg, 0.2 mmol), [Pd]-1(5.5 mg, 0.01 mmol), AgOAc (66.8 mg, 0.4 mmol), LiO^{t}Bu (48 mg, 0.6 mmol), and TcCu (7.64 mg, 0.04 mmol) in cyclohexane (0.5 mL) at 125 °C for 12 h. The title compound was obtained as a white solid (38.1 mg, 0.136 mmol, 68% yield, dr = 1:1). R_f (petroleum ether/dichloromethane = 20:1) = 0.3. **^{1}H NMR (400 MHz, CDCl$_3$)** δ 7.56 (m, 2H), 7.54 – 7.41 (m, 1H), 7.31 (m, 4H), 7.17 (m, 2H), 7.09 – 7.03 (m, 2H), 6.98 – 6.84 (m, 5H), 4.49 – 4.41 (m, 3H), 4.41 – 4.35 (m, 1H), 1.55 (d, *J* = 15.3 Hz, 1H), 1.48 (d, *J* = 15.3 Hz, 1H), 1.35 (d, *J* = 17.0 Hz, 1H), 1.28 (d, *J* = 17.0 Hz, 1H), 1.14 – 1.01 (m, 6H), 0.99 – 0.87 (m, 4H), 0.41 (s, 3H), 0.35 (s, 3H). **^{13}C NMR (101 MHz, CDCl$_3$)** δ 159.82, 159.72, 156.26, 156.21, 139.77, 139.07, 138.95, 137.80, 131.97, 131.90, 130.38, 130.35, 128.06, 126.79, 126.77, 125.70, 125.67, 123.82, 123.37, 123.32, 121.90, 121.30, 121.28, 119.73, 111.22, 109.63, 87.39, 87.33, 57.59, 27.11, 26.70, 10.29, 7.70, 7.61, 7.57, 7.29, 6.97, 6.79, -2.81, -3.71. **HRMS (EI)** calcd. For $C_{18}H_{20}OSi$ $[M]^+$: 280.1283. Found: 280.1275.

1'-Methyl-1'-phenyl-1',2'-dihydro-2*H*-spiro[benzofuran-3,3'-benzo[*b*]-silole] (2ec)

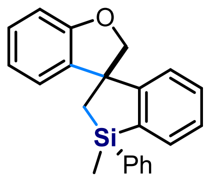

2ec

Following the general procedure, the reaction was carried out with **1ec** (94.0 mg, 0.2 mmol), [Pd]-1(5.5 mg, 0.01 mmol), AgOAc (66.8 mg, 0.4 mmol), LiO^{t}Bu (48 mg, 0.6 mmol), and TcCu (7.64 mg, 0.04 mmol) in cyclohexane (0.5 mL) at 125 °C for 12 h.

The title compound was obtained as a white solid (14.4 mg, 0.044 mmol, 22% yield, dr = 1:1). R_f (petroleum ether/dichloromethane = 20:1) = 0.3. **^{1}H NMR (400 MHz, CDCl$_3$)** δ 7.71 – 7.65 (m, 1H), 7.60 (m, 3H), 7.50 (m, 2H), 7.45 – 7.26 (m, 10H), 7.22 – 7.09 (m, 4H), 6.99 (m, 1H), 6.94 – 6.80 (m, 5H), 4.53 (d, *J* = 8.5 Hz, 1H), 4.49 (d, *J* = 8.5 Hz, 1H), 4.29 (m, 2H), 1.79 (d, *J* = 15.3 Hz, 1H), 1.70 (d, *J* = 15.3 Hz, 1H), 1.63 (d, *J* = 15.3 Hz, 1H), 1.50 (d, *J* = 15.3 Hz, 1H), 0.70 (s, 3H), 0.67 (s, 3H). **^{13}C NMR (101 MHz, CDCl$_3$)** δ 162.29, 162.13, 159.25, 140.17, 139.90, 139.84, 139.51, 138.75, 136.74, 136.56, 134.84, 133.21, 132.19, 132.03, 130.57, 130.50, 129.58, 129.52, 128.20, 128.16, 125.92, 125.75, 123.75, 112.12, 112.09, 89.62, 89.20, 60.25, 59.99, 30.84, 30.71, -0.00, -0.16. **HRMS (EI)** calcd. For $C_{22}H_{20}OSi$ [M]$^+$: 328.1283. Found: 328.1275.

1',1'-diethyl-1',2'-dihydro-2*H*-spiro[benzofuran-3,3'-benzo[*b*]silole] (2ed)

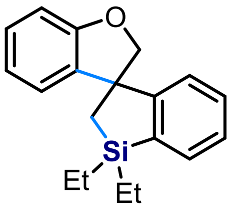

2ed

Following the general procedure, the reaction was carried out with **1ed** (90.0 mg, 0.2 mmol), [Pd]-1(5.5 mg, 0.01 mmol), AgOAc (66.8 mg, 0.4 mmol), LiO^{t}Bu (48 mg, 0.6 mmol), and TcCu (7.64 mg, 0.04 mmol) in cyclohexane (0.5 mL) at 125 ℃ for 12 h. The title compound was obtained as a white solid (35.2 mg, 0.120 mmol, 60% yield). R_f (petroleum ether/dichloromethane = 20:1) = 0.3. **^{1}H NMR (400 MHz, CDCl$_3$)** δ 7.55 (dd, *J* = 7.3, 1.6 Hz, 1H), 7.32 (dd, *J* = 7.3, 1.6 Hz, 1H), 7.28 (m, 1H), 7.17 (m, 1H), 7.05 (m, 1H), 6.94 – 6.82 (m, 3H), 4.43 (d, *J* = 8.5 Hz, 1H), 4.39 (d, *J* = 8.5 Hz, 1H), 1.48 (d, *J* = 15.4 Hz, 1H), 1.31 (d, *J* = 15.4 Hz, 1H), 1.04 (m, 6H), 0.95 – 0.87 (m, 2H), 0.86 – 0.76 (m, 2H). **^{13}C NMR (101 MHz, CDCl$_3$)** δ 159.71, 156.55, 138.10, 138.03, 132.20, 130.34, 128.02, 126.66, 125.80, 123.28, 121.30, 109.58, 87.69, 57.54, 24.90,

7.79, 7.63, 5.77, 4.92. **HRMS (EI)** calcd. For $C_{19}H_{22}OSi$ $[M]^+$: 294.1440. Found: 294.1434.

Removal of the Protecting Group (-Ts) in Spirosilacycle 2dd

General Procedure

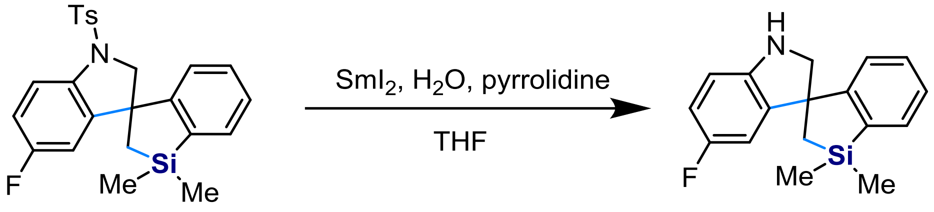

2dd **5**, 95% yield

The compound **5** was prepared according to the literature with modification.[8]

Preparation of SmI₂ in THF

Diiodoethane (13 mmol, 3.65 g) was added to THF (100 ml). To this was added samarium metal (19 mmol, 2,85 g, powder). The mixture was allowed to stir at room temperature under a nitrogen atmosphere for one hour. This yields a deep-blue 0.13 M solution of SmI_2.

Experiment:

To a solution of SmI_2 (13 mL, 0.13 M, 1 mmol) in THF was added the sulfonamide (0.1 mmol) followed by water (60 µL, 0.3 mmol) and pyrrolidine (173 µL, 2 mmol) under a nitrogen atmosphere. The reaction mixture immediately turned white upon addition of amine. The resulting mixture was diluted with diethyl ether (16 mL) and treated with a solution of potassium sodium tartrate and potassium carbonate (10% w/v each). The aqueous phase was extracted with two portions of diethyl ether. The organic extracts was pooled, dried and evaporated to yield the crude amine. The crude product was purified by preparative RP-HPLC on reversed phase column (C18(ODS)) (eluent: CH_3CN) to afford the corresponding product **5** (27mg, 0.095 mmol, 95% yield).

Characterization of Product 5

5'-Fluoro-1,1-dimethyl-1,2-dihydrospiro[benzo[b]silole-3,3'-indoline]. (5)

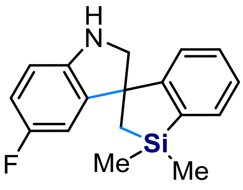

5

Compound **5** was obtained as colorless oil (R_f = 0.3, petroleum ether: ethyl acetate = 5:1) in 95% yield **^{1}H NMR (400 MHz, $CDCl_3$)** δ 7.51 – 7.41 (m, 1H), 7.22 (m, 1H), 7.19 – 7.13 (m, 1H), 7.00 (m, 1H), 6.66 (m, 1H), 6.55 (m, 1H), 6.45 (m, 1H), 3.45 (d, *J* = 8.9 Hz, 1H), 3.39 (d, *J* = 8.9 Hz, 1H), 1.51 (d, *J* = 15.1 Hz, 1H), 1.05 (d, *J* = 15.1 Hz, 1H), 0.30 (s, 3H), 0.25 (s, 3H). **^{13}C NMR (101 MHz, $CDCl_3$)** δ 159.01 (d, *J* = 241.0 Hz), 148.11, 142.64, 141.33, 133.02, 131.43, 128.12, 127.06, 115.01, 114.77, 111.86 (d, *J* = 23.5 Hz), 111.56 (d, *J* = 8.3 Hz), 66.91, 59.36, 29.13, 0.88, 0.01. **^{19}F NMR (376 MHz, $CDCl_3$)** δ -125.05. **HRMS (EI)** calcd. For $C_{17}H_{18}FNSi$ $[M]^+$: 283.1193. Found: 283.1188.

3. Supplementary Discussion

Experimental Mechanistic Study

GC Analyse of the Gas Composition of the Reactions

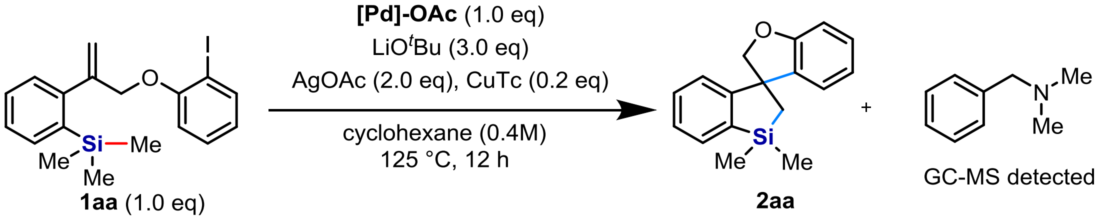

Experiment: Under the protection of nitrogen, the reaction was carried out with **1aa** (81.6 mg, 0.2 mmol), **[Pd]-OAc** (0.2 mmol), AgOAc (66.8 mg, 0.4 mmol), LiO^{t}Bu (48 mg, 0.6 mmol), and CuTc (7.64 mg, 0.04 mmol) in cyclohexane (0.5 mL) at 125 °C for

12 h. Then, the crude product was analyzed by GC-MS.

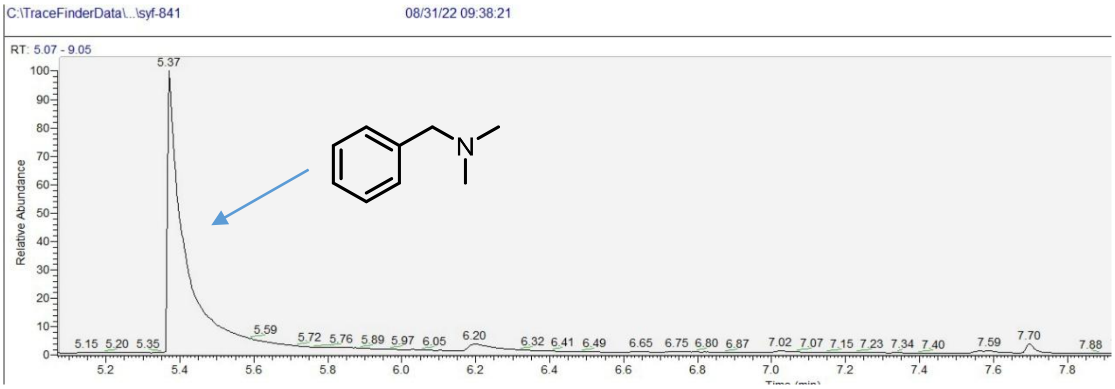

Supplementary Figure 1. GC analyses of the reaction of **1aa** in the presence of stoichiometric amount of [Pd]-OAc complex

X-ray Photoelectron Spectroscopy Analysis of Palladium Catalysts

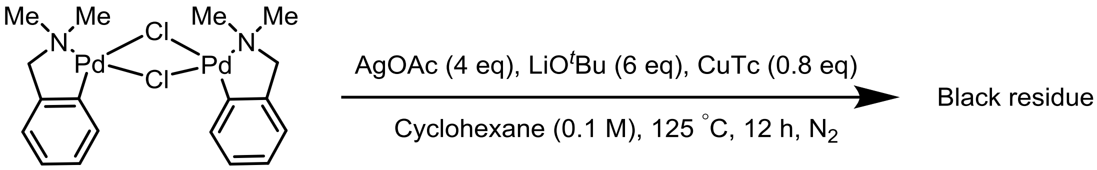

In a nitrogen-filled glovebox, an oven-dried 15 mL screw capped sealed tube was charged with a magnetic stir bar, [Pd]-1 (55.2 mg, 0.1 mmol, 1.0 equiv), AgOAc (66.8 mg, 0.4 mmol, 4.0 equiv), LiO^{t}Bu (48 mg, 0.6 mmol, 6.0 equiv), additive and cyclohexane (1.0 mL). The tube was sealed, then removed from the glovebox, and the formed mixture was stirred at 125 °C under N_2 for 12 h. After being cooled to room temperature, the organic phases were evaporated and the residues obtained for XPS test.

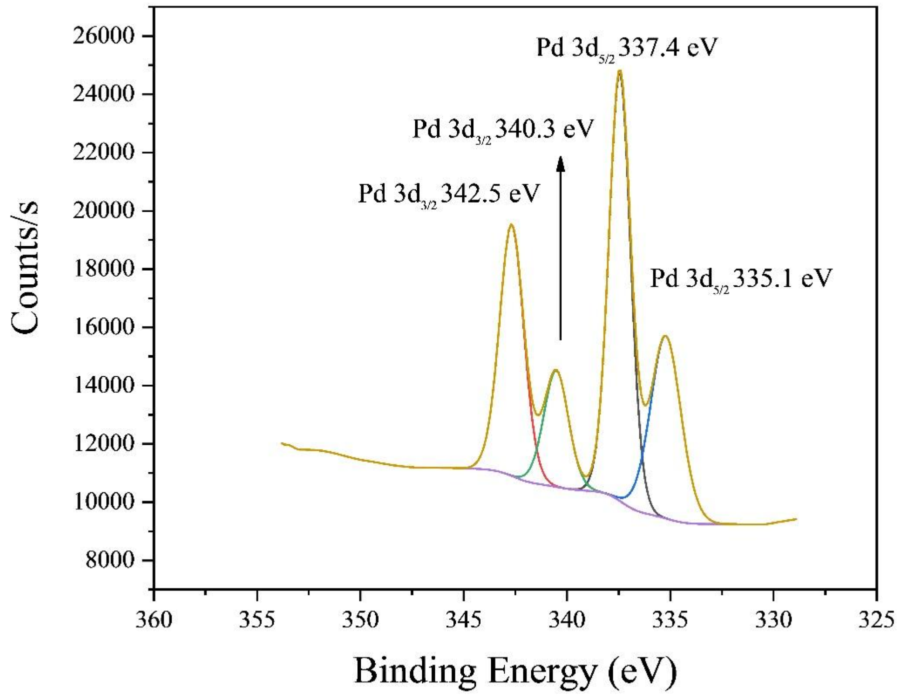

Supplementary Figure 2. XPS analysis of reaction residue to identify Pd(0) species

To gain information about the oxidation state of the Pd species, analysis of Pd 3d by X-ray photoelectron spectroscopy (XPS) was performed. All measurements were performed on a Thermo Scientific ESCALAB 250Xi spectrometer equipped with a monochromatic Al Kα X-ray source. The pressure throughout the analysis chamber was less than 10-7 mbar. Samples were finely ground in a glovebox and pressed into aluminum foil before being placed on a sample holder and transferred to the XPS instrument in a sealed container to avoid exposure to air during transport. All results were analyzed with use of the Avantage software package. Charge correction was calibrated with C 1s (284.8 eV) as reference.

The spectra showed that the electron binding energies of Pd $3d_{3/2}$ and $3d_{5/2}$ in black residue were 340.3 eV and 335.1 eV (see Supplementary Figure 2), respectively, which is consistent with the position for metallic Pd.[10] Further analysis showed that the electron binding energy (342.5 eV and 337.4 eV) were very close to the corresponding electron binding energy in PdO reported in the literature.[11] In summary, we deduced that the palladium complex for this Pd-catalyzed sila-spirocyclization of **1aa** can produce Pd(0).

Computational Studies

Quantum Mechanical Studies

Method. Density functional theory calculations were carried out in Gaussian 16.[12] Geometry optimization and frequency calculation employed the M06-L functional[13] and the def2-SV(P) basis set.[14] Single-point energies were then computed with the PBE0-D3 hybrid functional,[15] the def2-TZVP basis set,[14] and the SMD implicit solvation model[16] for cyclohexane. Free energies were calculated using the GoodVibes code applying quasi-harmonic corrections.[17] We assumed a concentration of 1 mol/L and a temperature of 398.15 K in all calculations.

Ligand Exchange. Possible ligation modes were evaluated on **IM1^{II}**. Supplementary Figure 3 presents calculated energies of ligand exchanges involving ^{t}BuO$^-$, AcO$^-$ and I$^-$. The calculations show that **IM1^{II}** with the ligation of ^{t}BuO$^-$ is of the lowest-energy in the scope of our investigation.

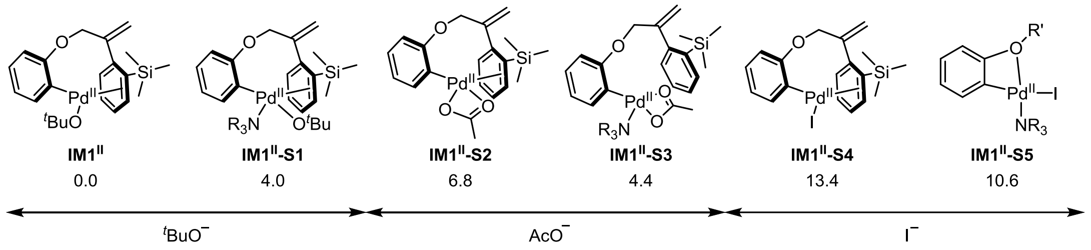

Supplementary Figure 3. Possible coordination modes of **IM1^{II}**. Relative free energies are shown in kcal/mol. Considered ligand exchanges involve AgI/AgOAc, LiO^{t}Bu/LiOAc and NR_3.

To further evaluate the influence of I$^-$/^{t}BuO$^-$ exchange, Supplementary Figure 4 compares the free-energy profiles with the ligations of these two anions. Our calculations show that the ^{t}BuO$^-$-coordinated species are substantially lower in energy than the I$^-$-coordinated counterparts. The result again supports the favorability of the ligand exchange.

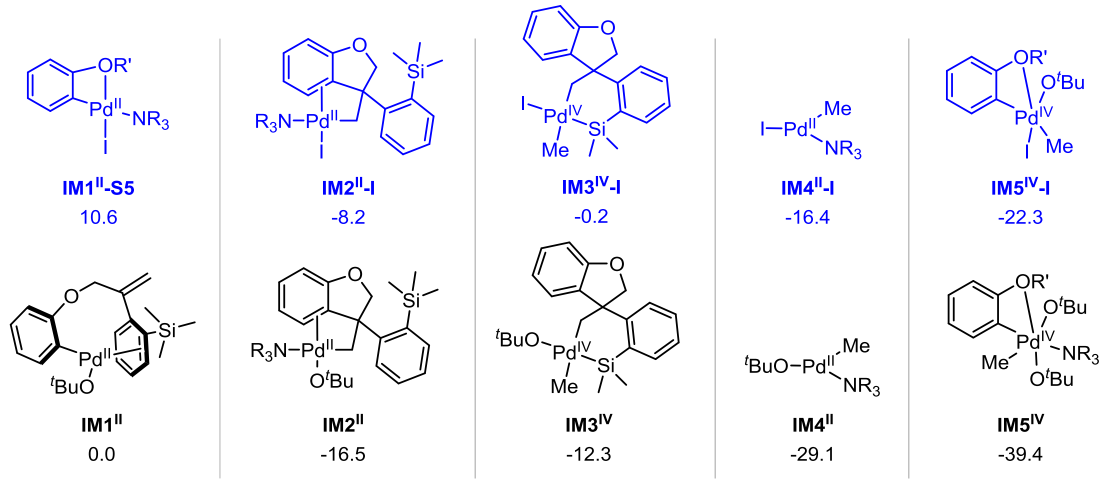

Supplementary Figure 4. Effect of I$^-$/^{t}BuO$^-$ exchange on the free-energy profile (unit: kcal/mol).

Reductive Elimination. In addition, several possibly competing reductive elimination pathways were also considered. The results in Supplementary Figure 5 suggest that the following two pathways are kinetically less likely due to elevated activation barrier: (1) formation of Ar–Me bond from reductive elimination (**TS5$_{RE}$^{ArMe}**), (2) reductive elimination with I$^-$ substituting one ^{t}BuO$^-$ (**TS5'$_{RE}$** and **TS5''$_{RE}$**).

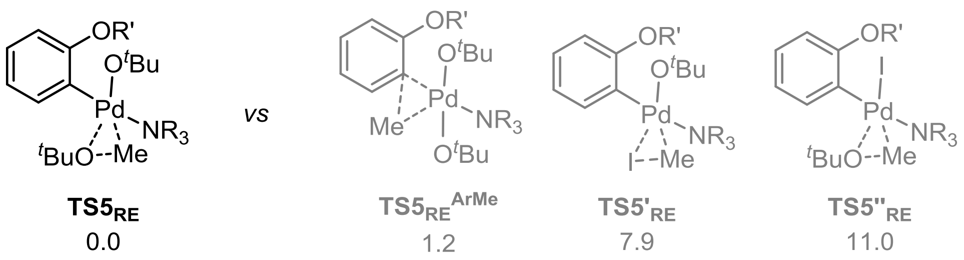

Supplementary Figure 5. Relative free energies of possible reductive-elimination pathways (unit: kcal/mol).

Energies and Coordinates of Calculated Structures

Supplementary Table 2. Energies, enthalpies, and free energies of all calculated structures. Imaginary frequencies of all transition states

	E (a.u.)	H (a.u.)	G (a.u.)	ν_{imag} (cm^{-1})
**1aa**	-1360.310142	-1359.938274	-1360.048278	

**2aa**	-1022.742602	-1022.412805	-1022.504362	
AgI	-444.764425	-444.758747	-444.796127	
AgOAc	-375.331716	-375.271678	-375.322048	
LiOAc	-235.915152	-235.854215	-235.899226	
LiO^{t}Bu	-240.436963	-240.298866	-240.352587	
MeO^{t}Bu	-272.761065	-272.584109	-272.639251	
NR_3	-174.328517	-174.198890	-174.243829	
**IM1^{II}**	-1423.387087	-1422.880765	-1423.012122	
**IM1^{II}-S1**	-1597.738929	-1597.100026	-1597.249585	
**IM1^{II}-S2**	-1418.853391	-1418.423836	-1418.547932	
**IM1^{II}-S3**	-1593.212452	-1592.650868	-1592.795610	
**IM1^{II}-S4**	-1488.269266	-1487.895407	-1488.011492	
**IM1^{II}-S5**	-1662.626090	-1662.120973	-1662.259735	
**IM2^{II}**	-1597.771892	-1597.132380	-1597.282322	
**IM3^{IV}**	-1423.409621	-1422.902375	-1423.031677	
**IM4^{II}**	-575.018218	-574.710387	-574.797999	
**IM5^{IV}**	-1870.474403	-1869.660372	-1869.842037	
**IM2^{II}-I**	-1662.662102	-1662.154915	-1662.289747	
**IM3^{IV}-I**	-1488.295490	-1487.919887	-1488.033147	
**IM4^{II}-I**	-639.902817	-639.726959	-639.798496	
**IM5^{IV}-I**	-1760.991662	-1760.442971	-1760.591549	
**TS1$_{MI}$**	-1597.730456	-1597.092329	-1597.239357	-230.48
**TS1'$_{MI}$**	-1423.363221	-1422.857303	-1422.984901	-313.26
**TS2$_{OA}$**	-1423.393699	-1422.887285	-1423.012112	-90.73
**TS3$_{RE}$**	-1423.405440	-1422.899493	-1423.026906	-44.62
**TS4$_{OA}$**	-1760.974869	-1760.427900	-1760.575767	-61.82
**TS5$_{RE}$**	-1870.435138	-1869.622279	-1869.802512	-387.89
**TS5'$_{RE}$**	-1935.323831	-1934.644489	-1934.810646	-223.73
**TS5''$_{RE}$**	-1935.319587	-1934.639725	-1934.805603	-374.44
**TS5$_{RE}^{ArMe}$**	-1870.434382	-1869.620620	-1869.800535	-363.62
**TS6$_{RE}$**	-574.955397	-574.648758	-574.734828	-511.46

4. Supplementary Figures

NMR Spectra

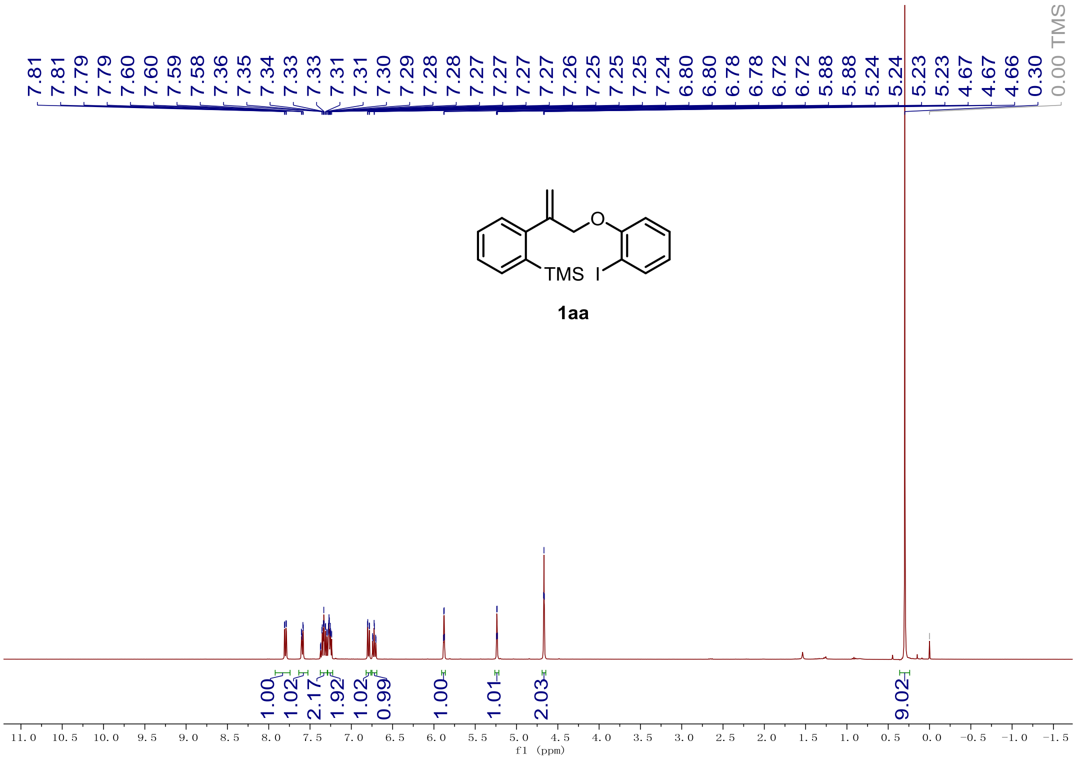

Supplementary Figure 6. ¹H NMR (400 MHz, CDCl₃) spectra of **1aa**

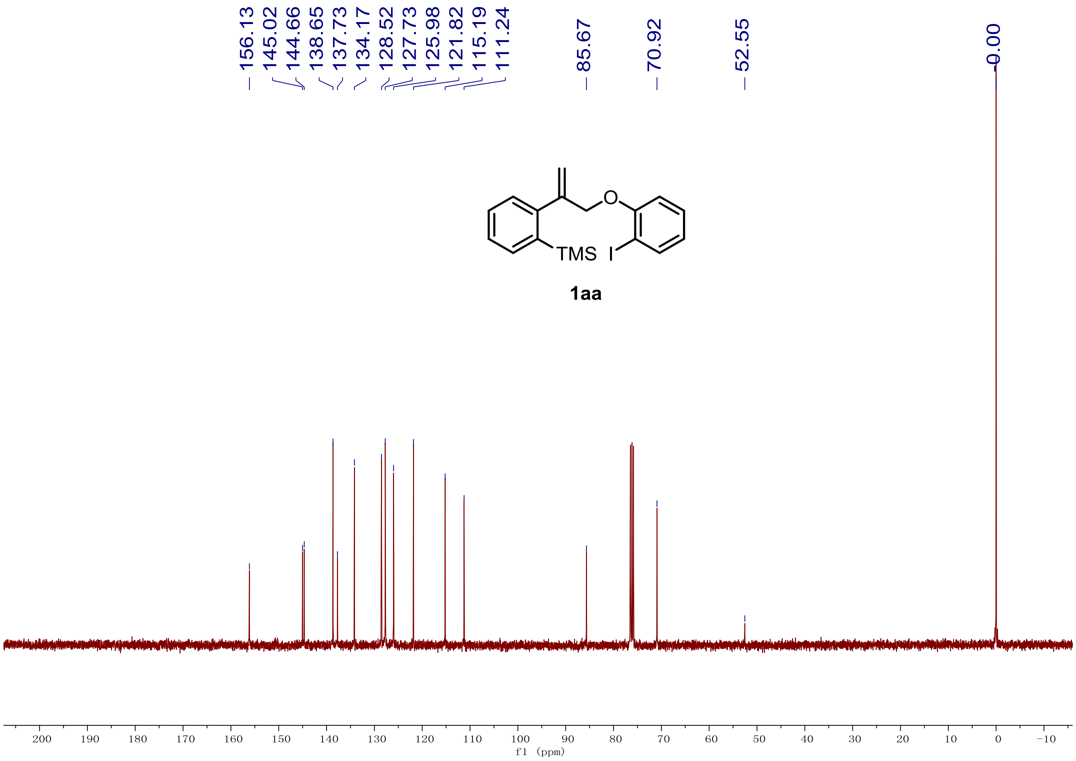

Supplementary Figure 7. ¹³C NMR (101 MHz, CDCl₃) spectra of **1aa**

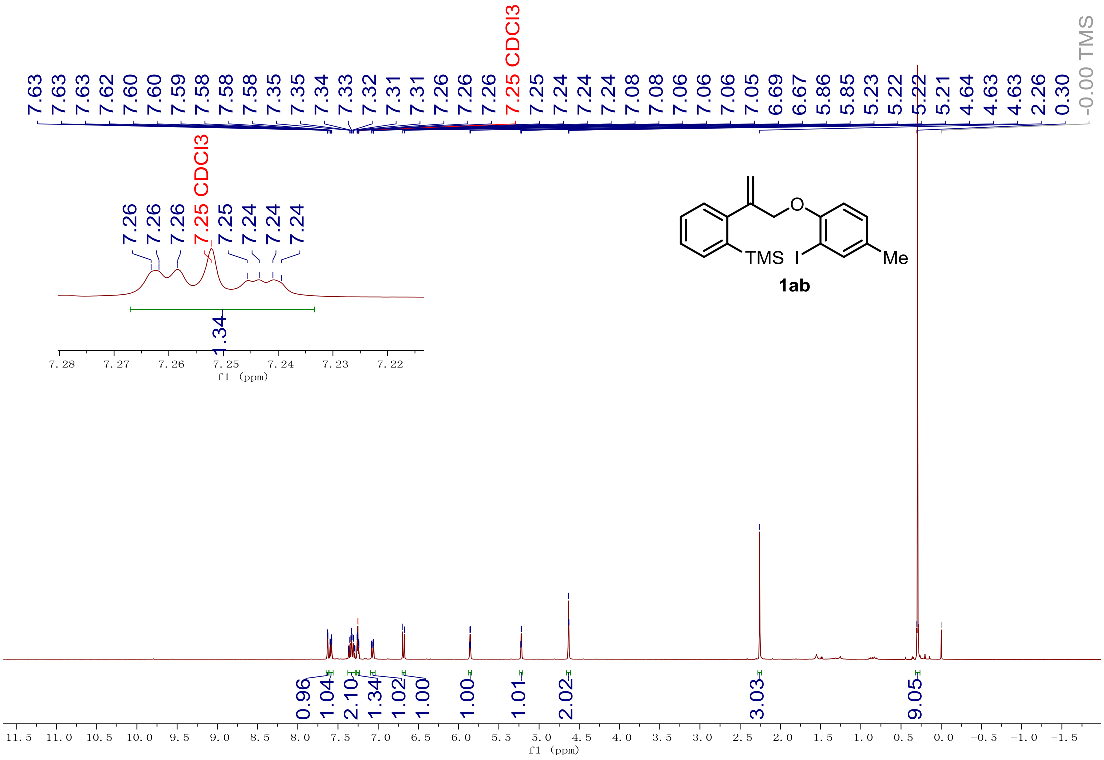

Supplementary Figure 8. ^{1}H NMR (400 MHz, $CDCl_3$) spectra of 1ab

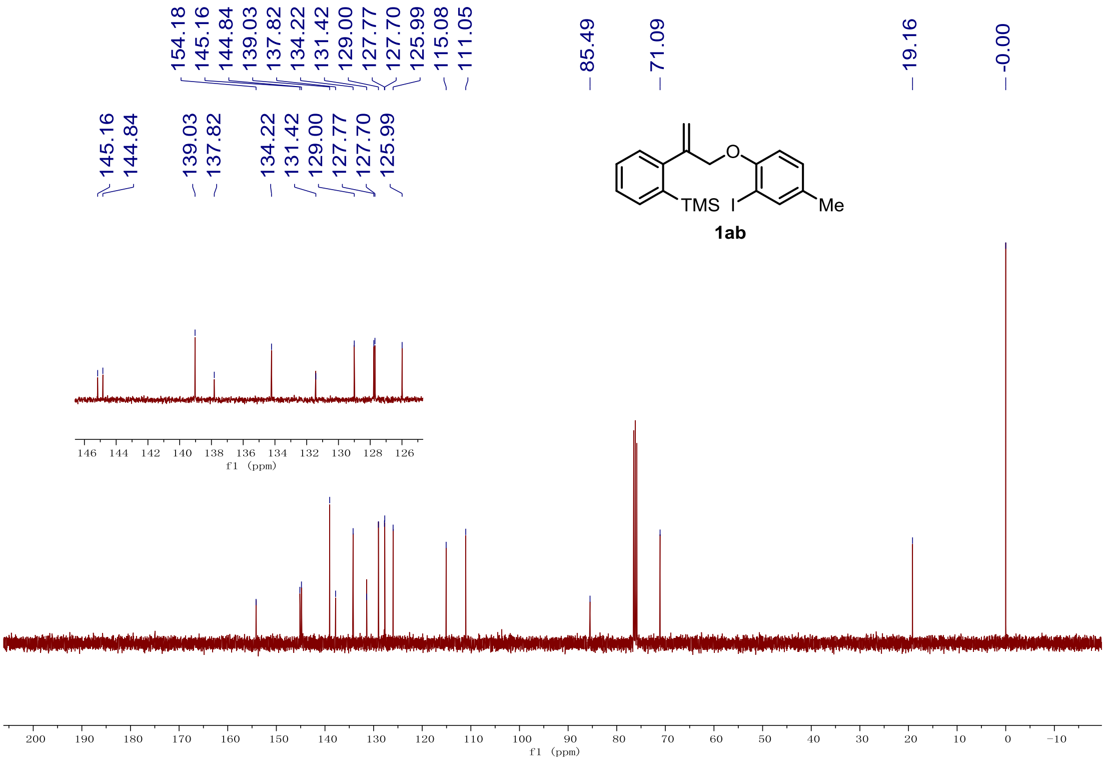

Supplementary Figure 9. ^{13}C NMR (101 MHz, $CDCl_3$) spectra of 1ab

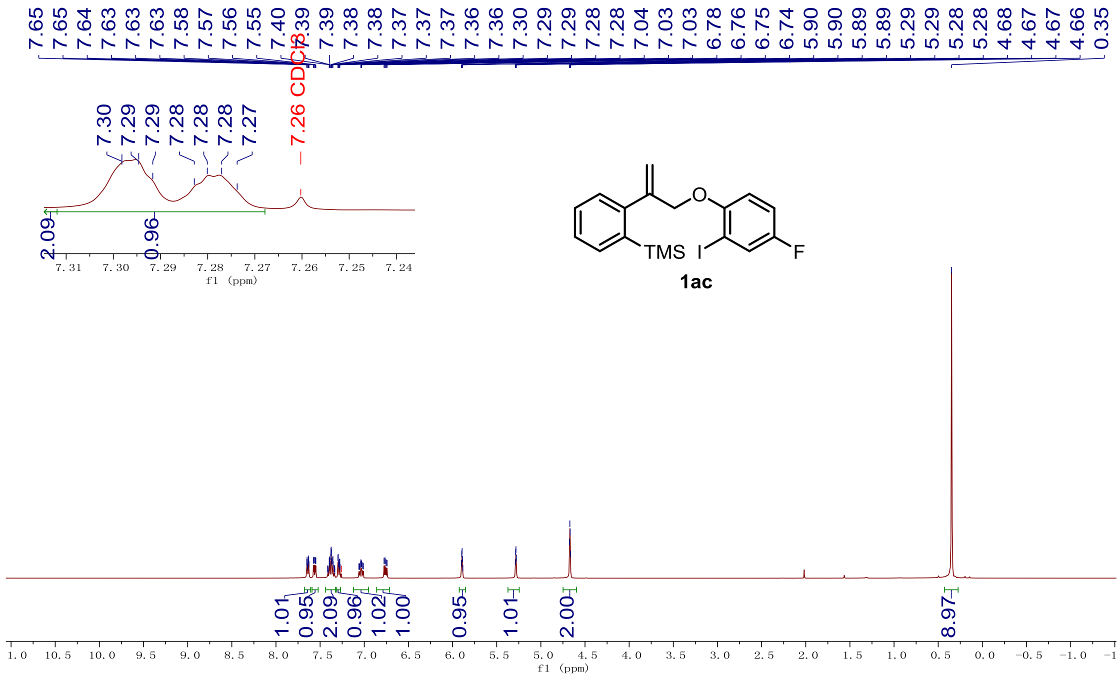

Supplementary Figure 10. ^{1}**H NMR (400 MHz, CDCl$_3$)** spectra of **1ac**

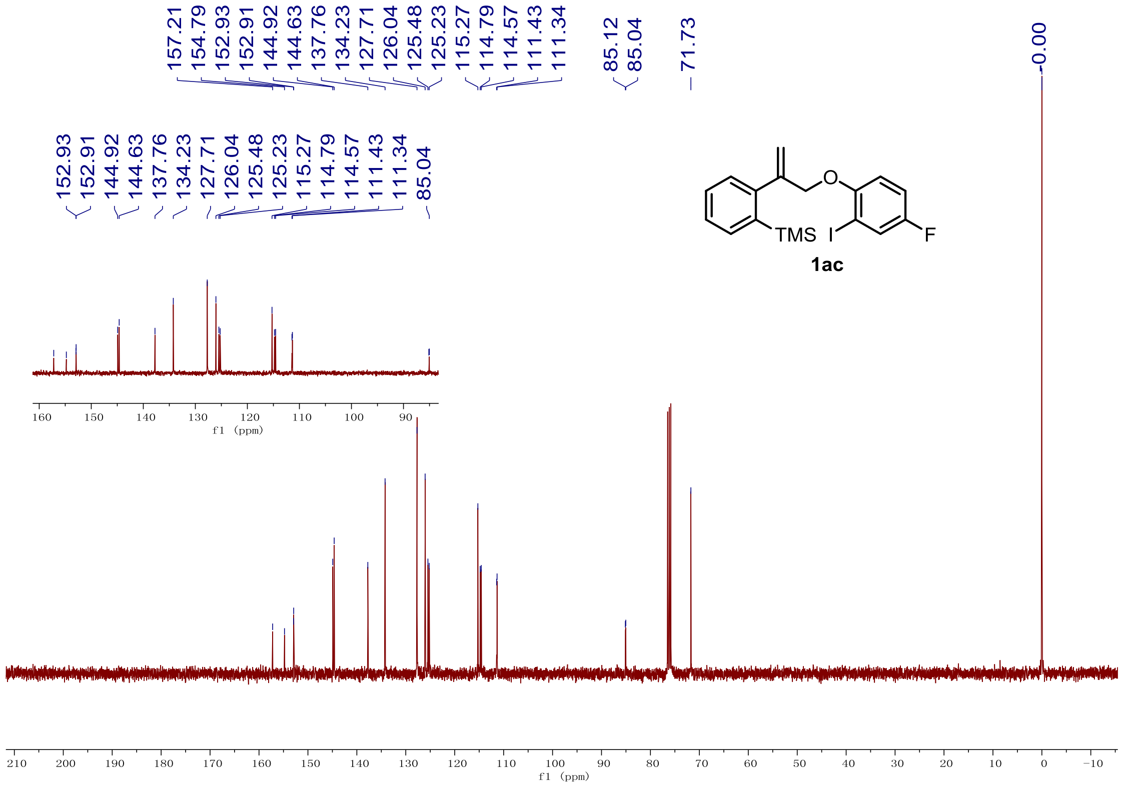

Supplementary Figure 11. ^{13}**C NMR (101 MHz, CDCl$_3$)** spectra of **1ac**

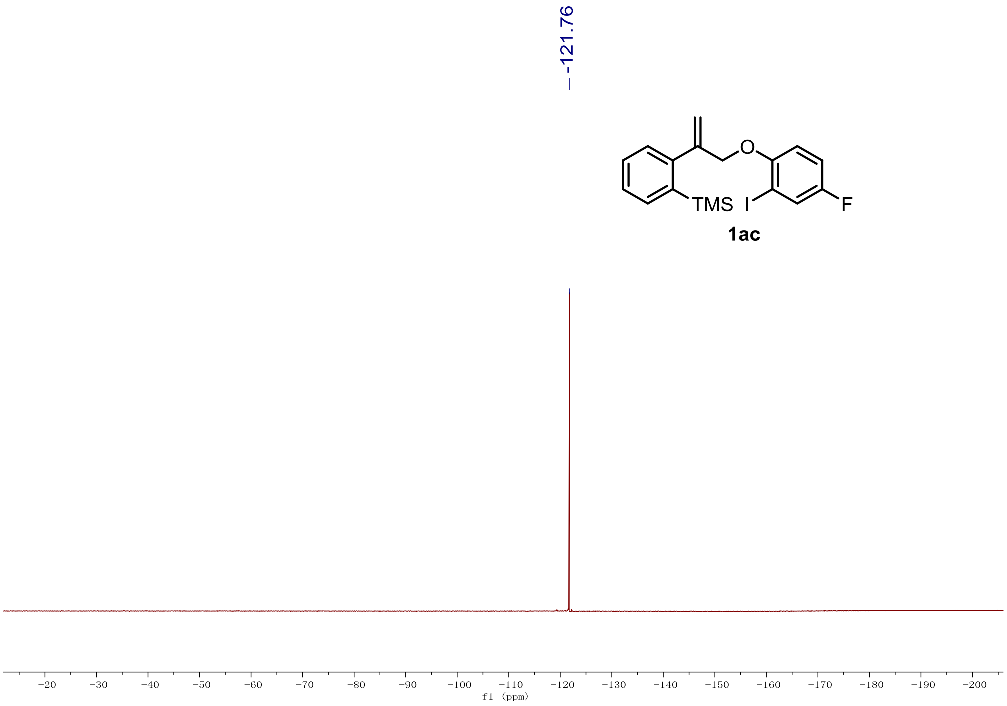

Supplementary Figure 12. ^{19}F NMR (376 MHz, $CDCl_3$) spectra of **1ac**

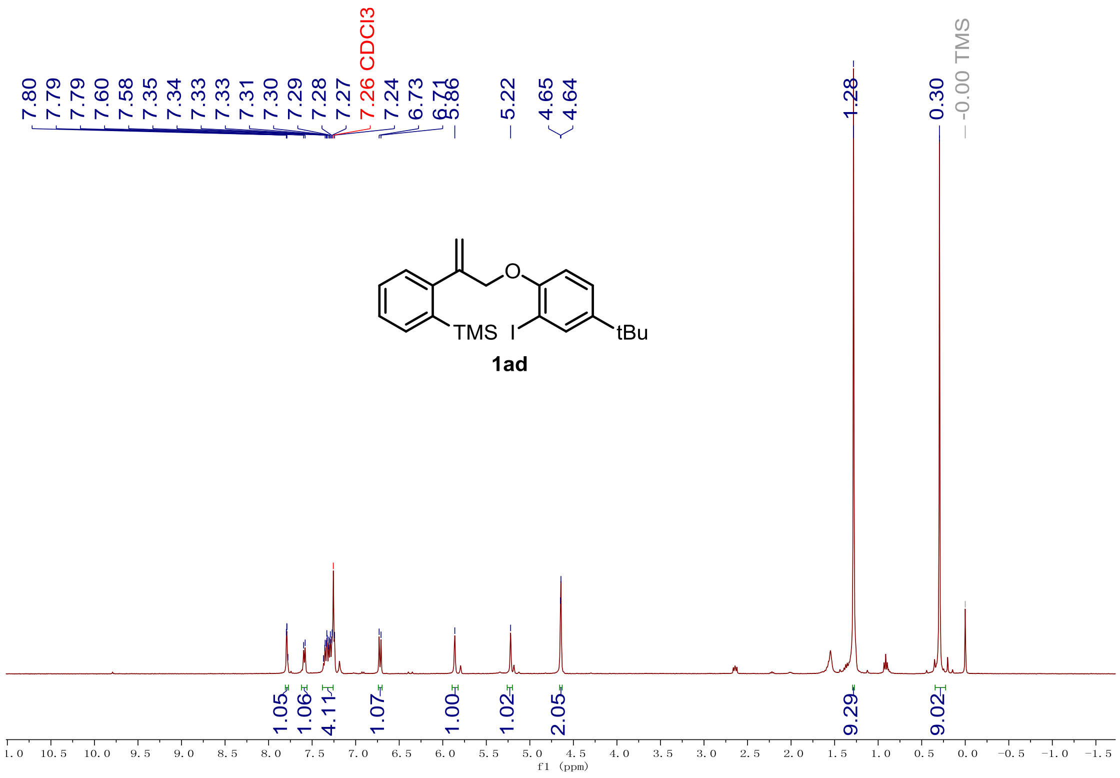

Supplementary Figure 13. ^{1}**H NMR (400 MHz, CDCl$_3$)** spectra of **1ad**

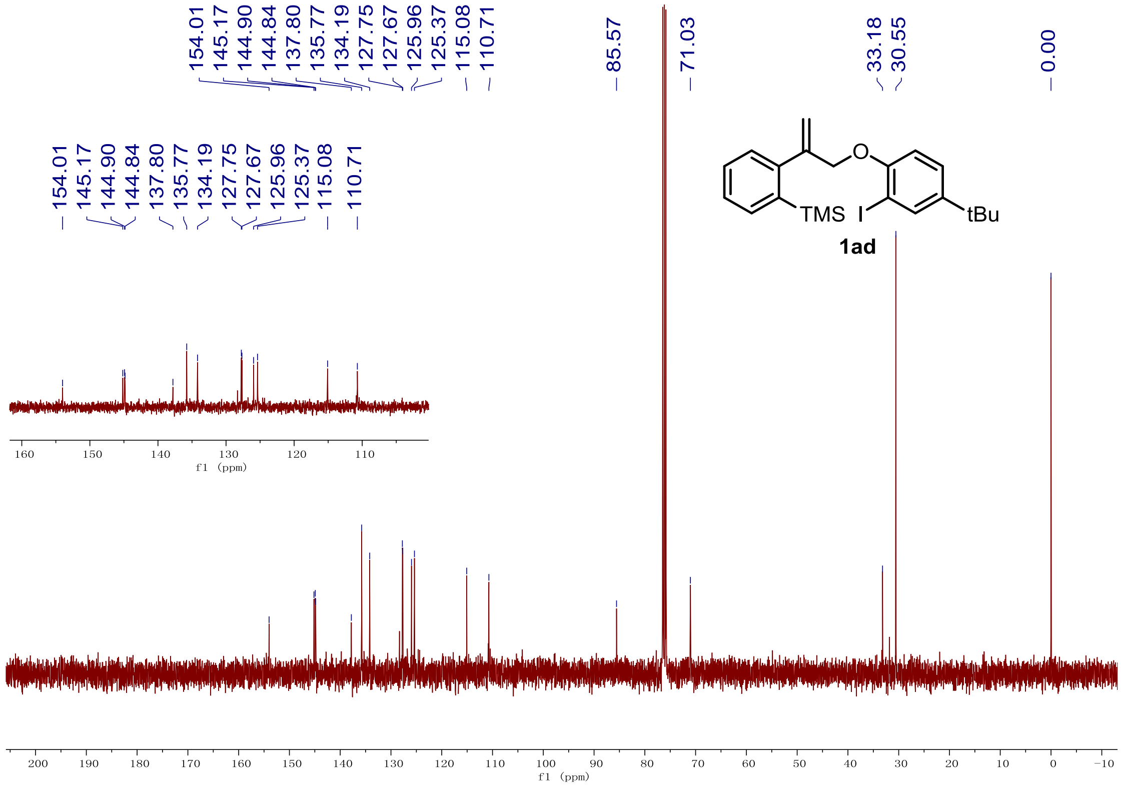

Supplementary Figure 14. ^{13}**C NMR (101 MHz, CDCl$_3$)** spectra of **1ad**

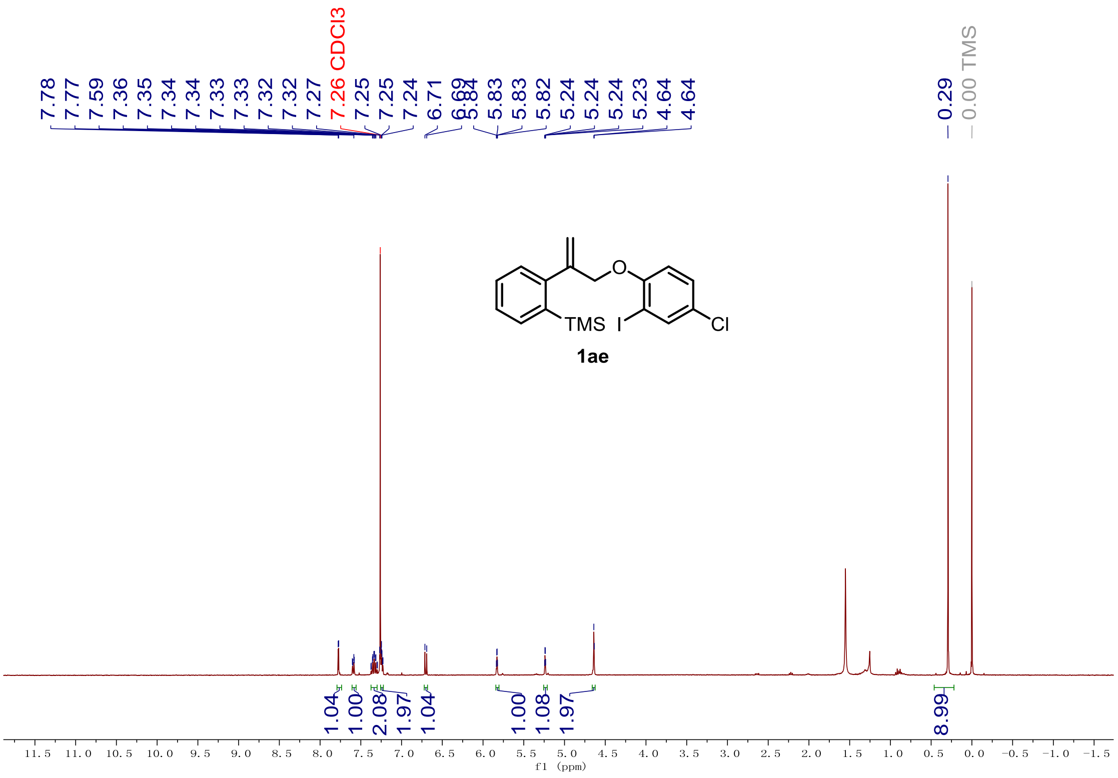

Supplementary Figure 15. ^{1}H NMR (400 MHz, $CDCl_3$) spectra of **1ae**

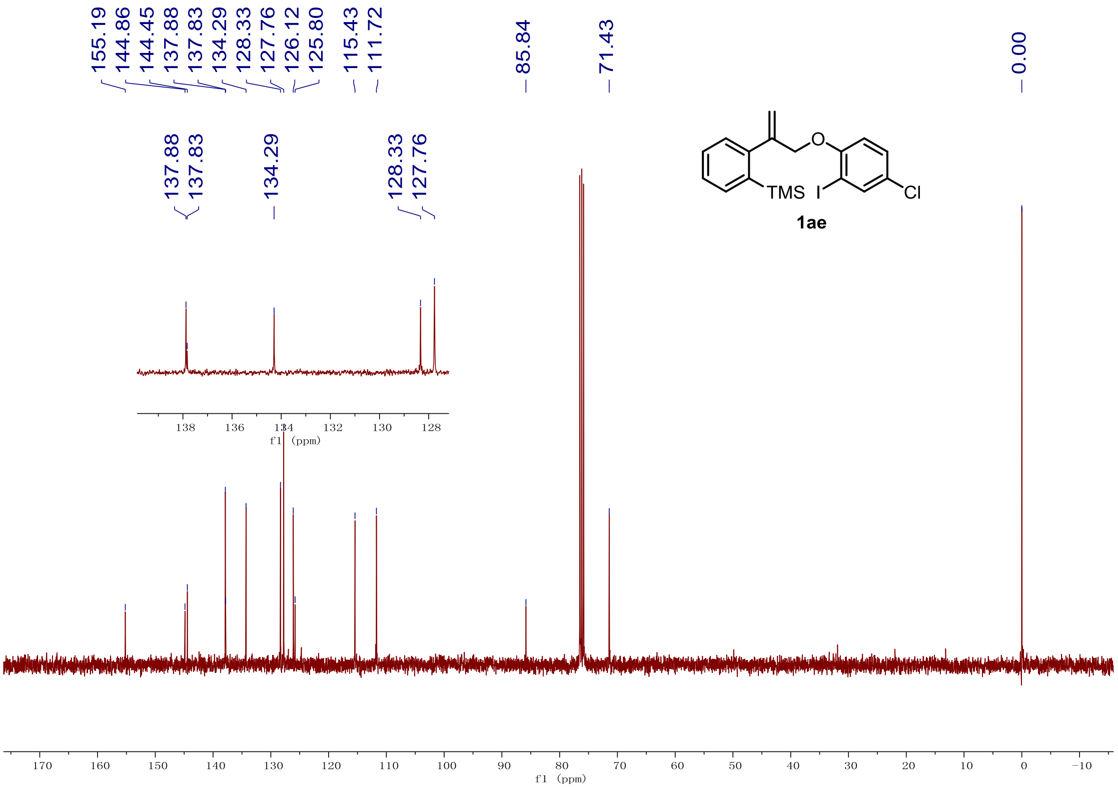

Supplementary Figure 16. ^{13}C NMR (101 MHz, $CDCl_3$) spectra of **1ae**

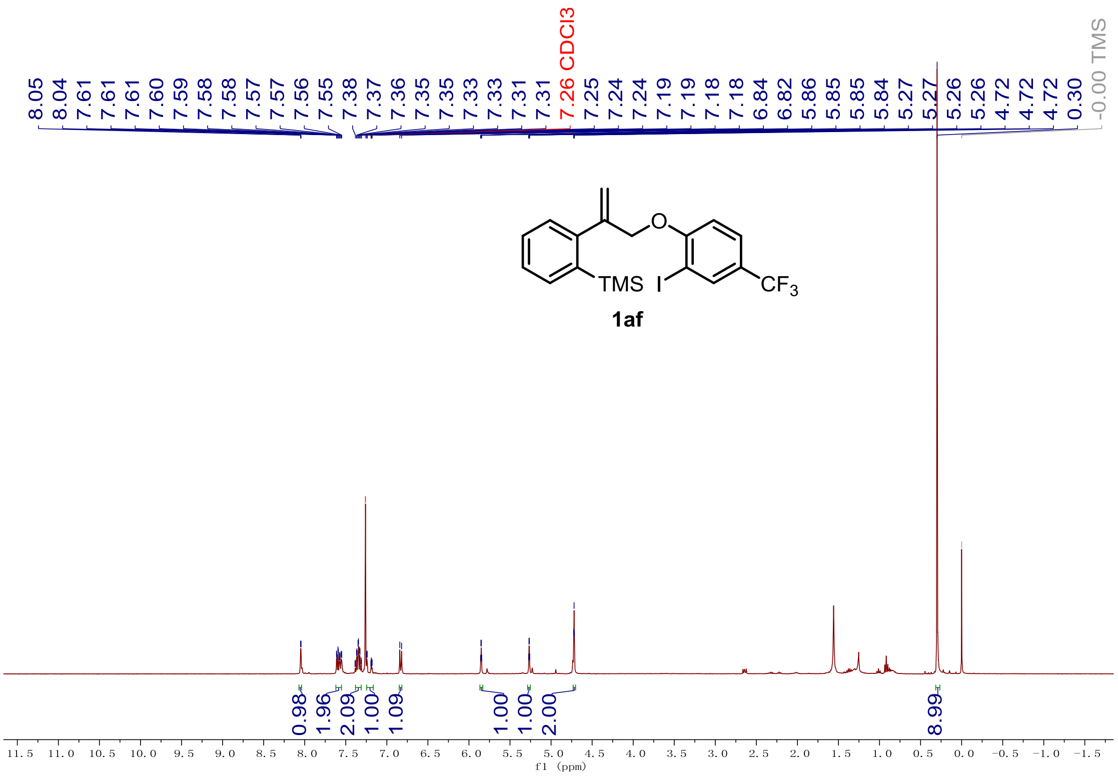

Supplementary Figure 17. ^{1}**H NMR (400 MHz, CDCl$_3$)** spectra of **1af**

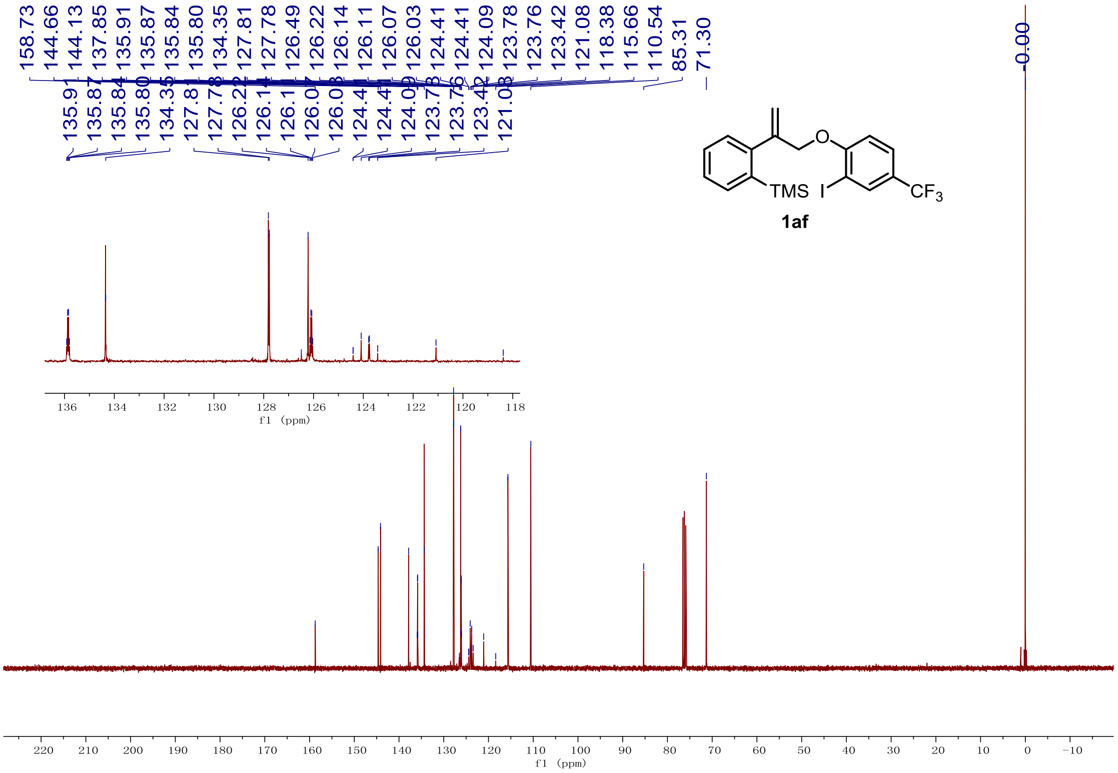

Supplementary Figure 18. ^{13}**C NMR (101 MHz, CDCl$_3$)** spectra of **1af**

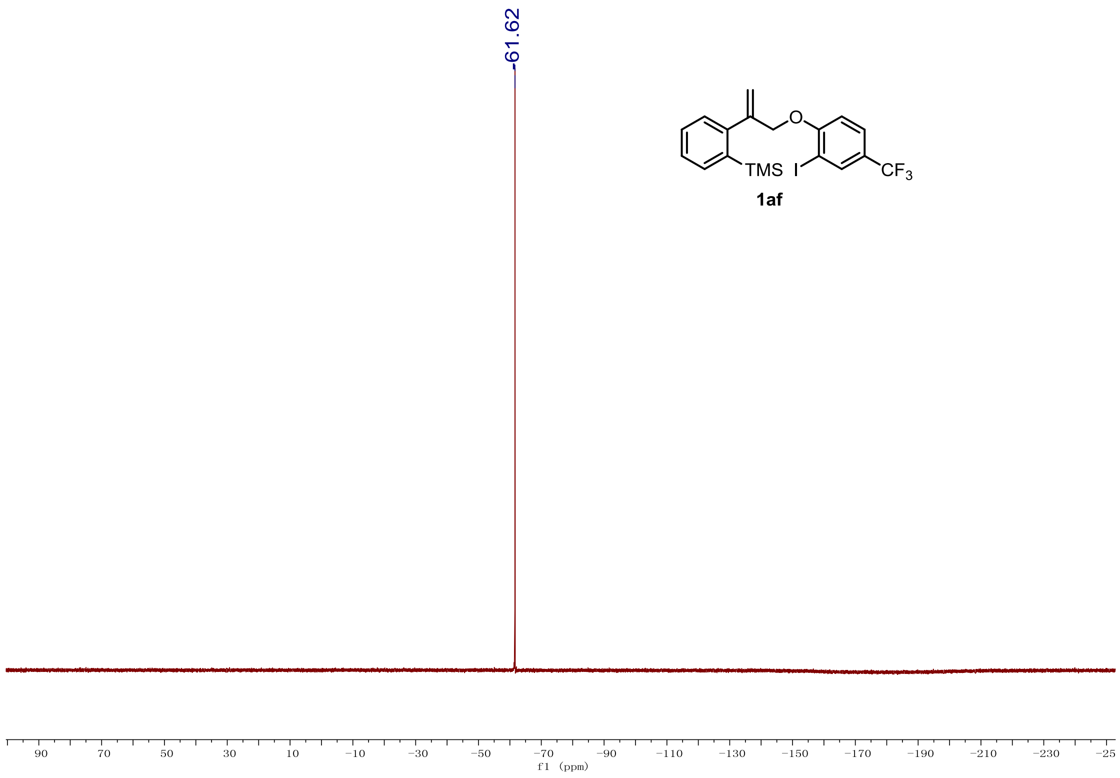

Supplementary Figure 19. ^{19}**F NMR (376 MHz,** $CDCl_3$**)** spectra of **1af**

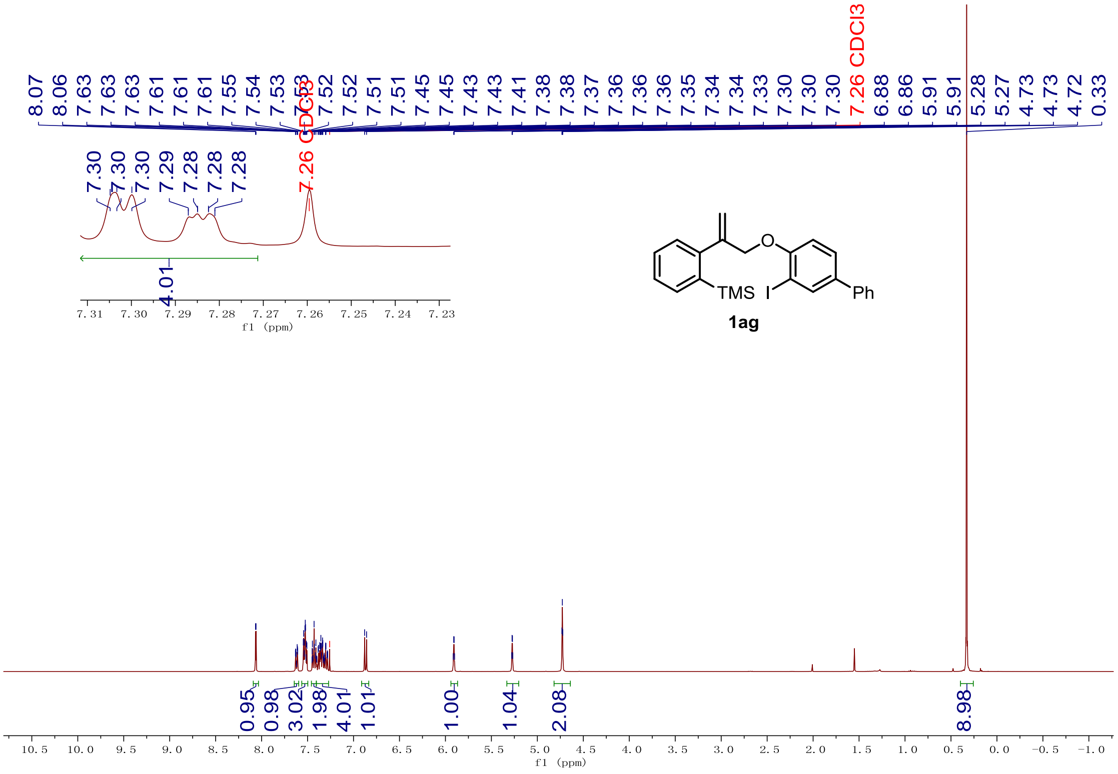

Supplementary Figure 20. ^{1}**H NMR (400 MHz, CDCl$_3$)** spectra of **1ag**

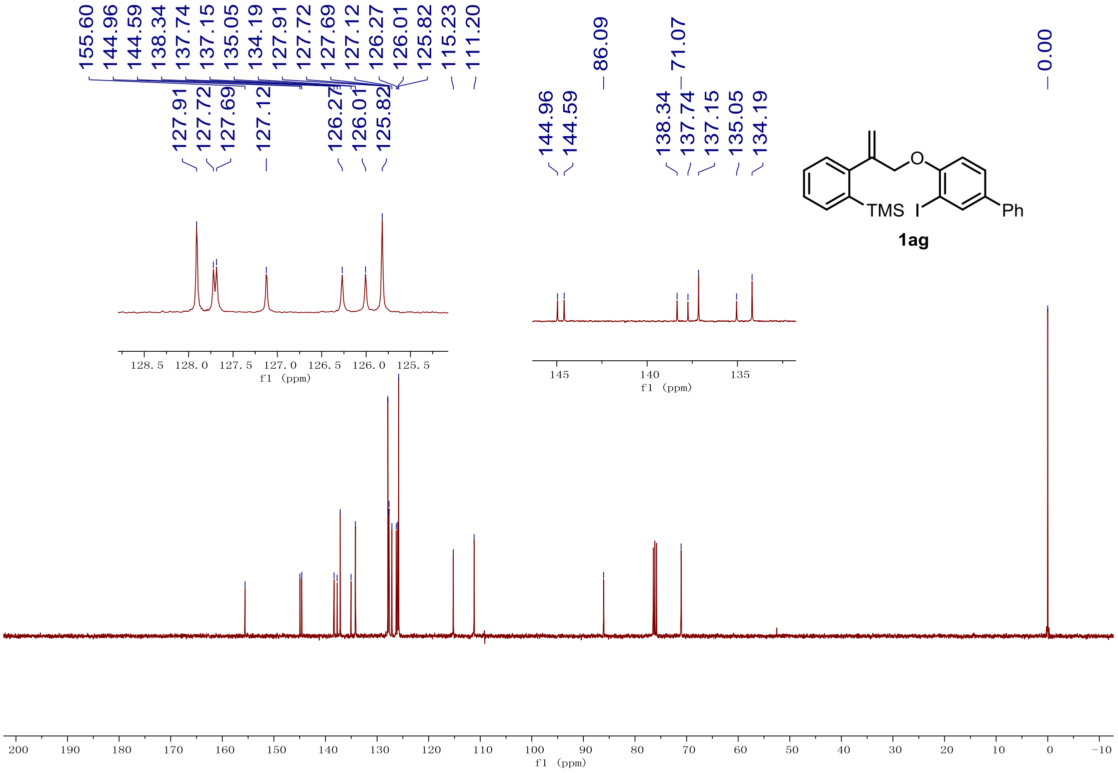

Supplementary Figure 21. ^{13}**C NMR (101 MHz, CDCl$_3$)** spectra of **1ag**

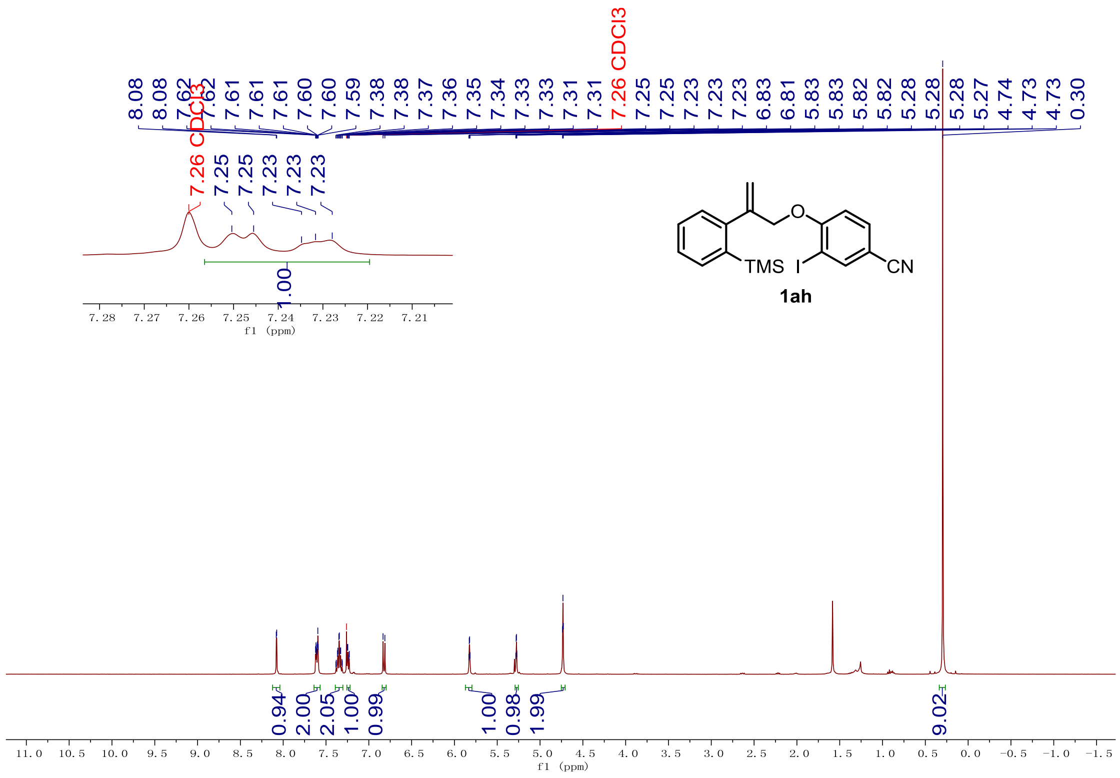

Supplementary Figure 22. ^{1}**H NMR (400 MHz, CDCl$_3$)** spectra of **1ah**

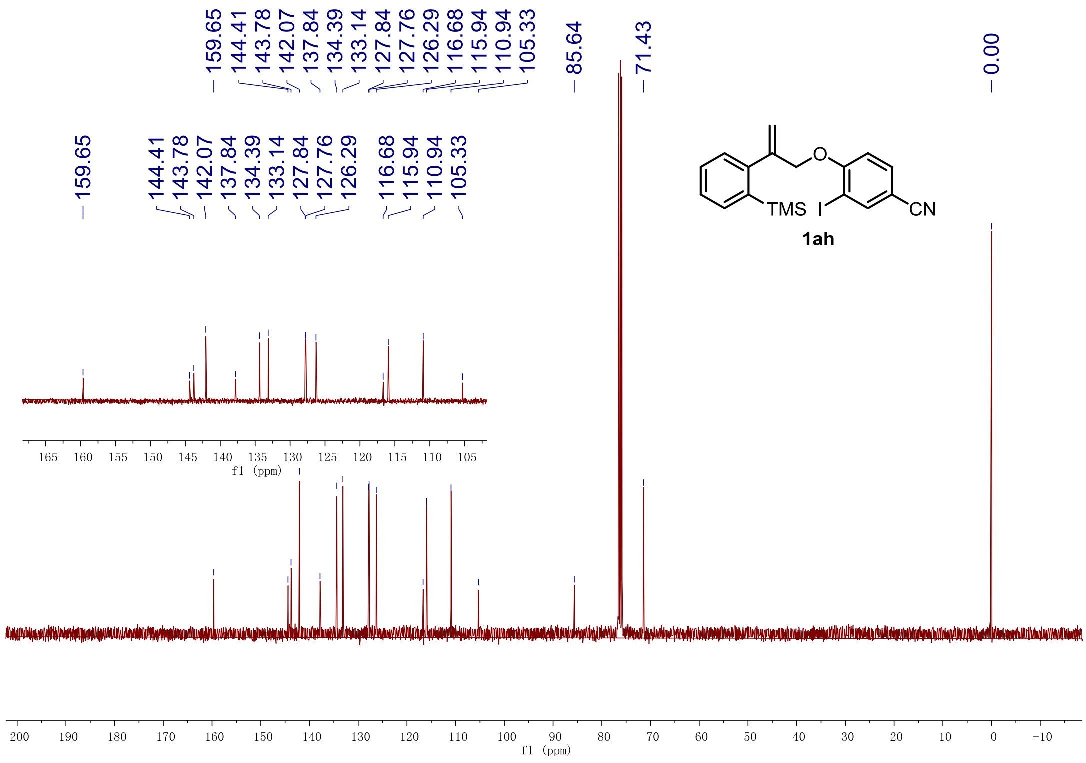

Supplementary Figure 23. ^{13}**C NMR (101 MHz, CDCl$_3$)** spectra of **1ah**

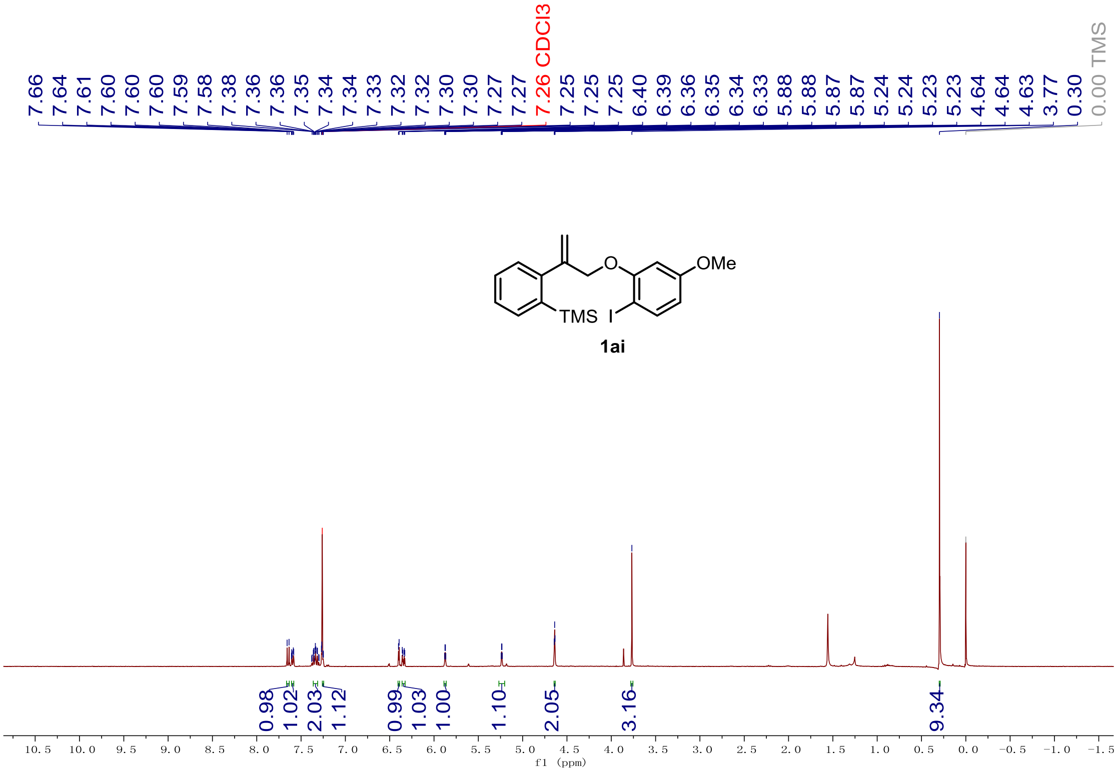

Supplementary Figure 24. ^{1}**H NMR (400 MHz, CDCl$_3$)** spectra of **1ai**

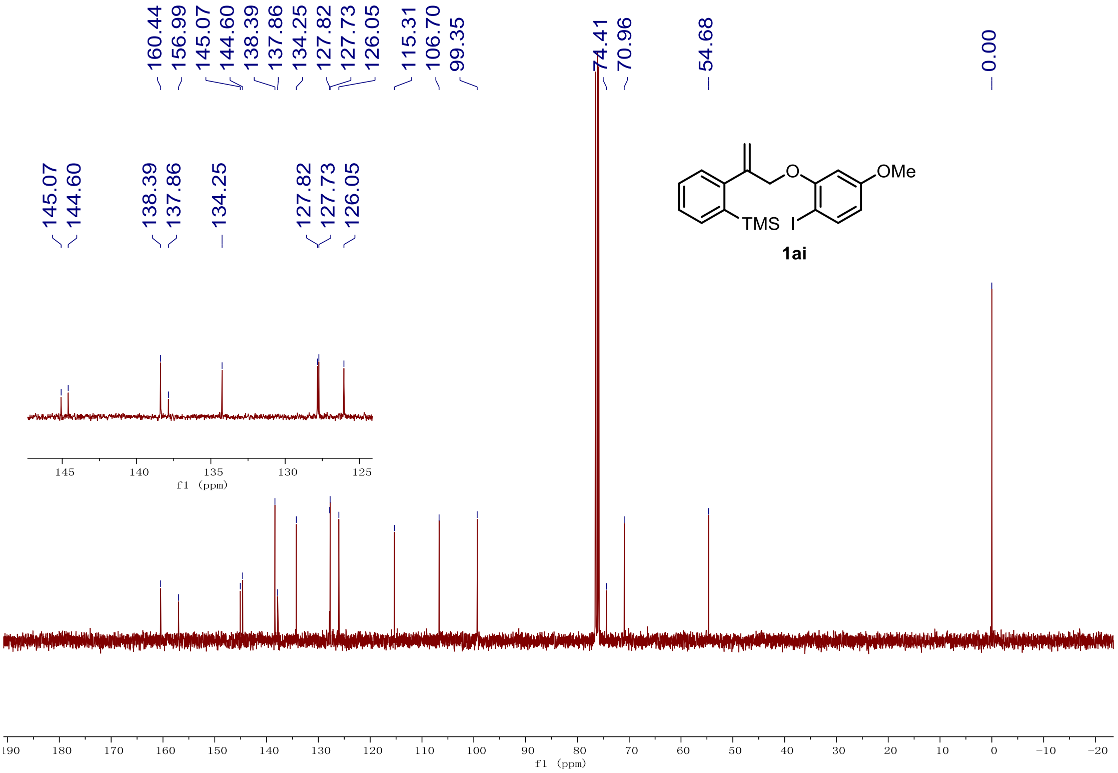

Supplementary Figure 25. ^{13}**C NMR (101 MHz, CDCl$_3$)** spectra of **1ai**

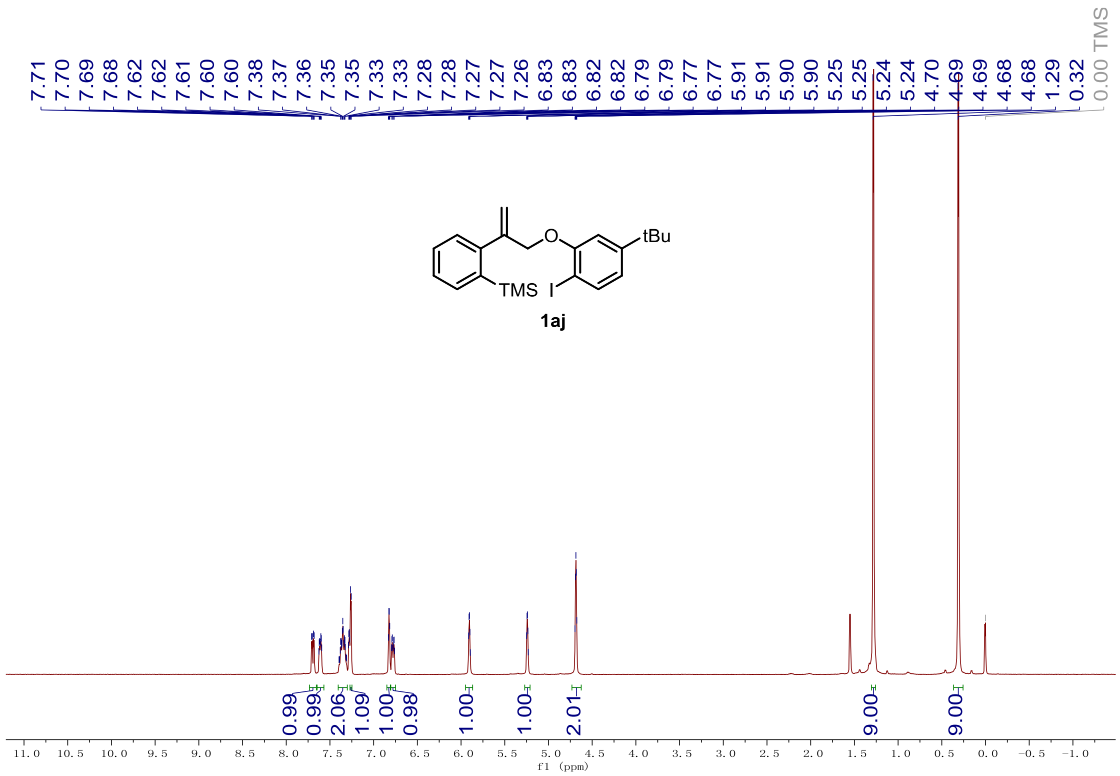

Supplementary Figure 26. ^{1}**H NMR (400 MHz, CDCl$_3$)** spectra of **1aj**

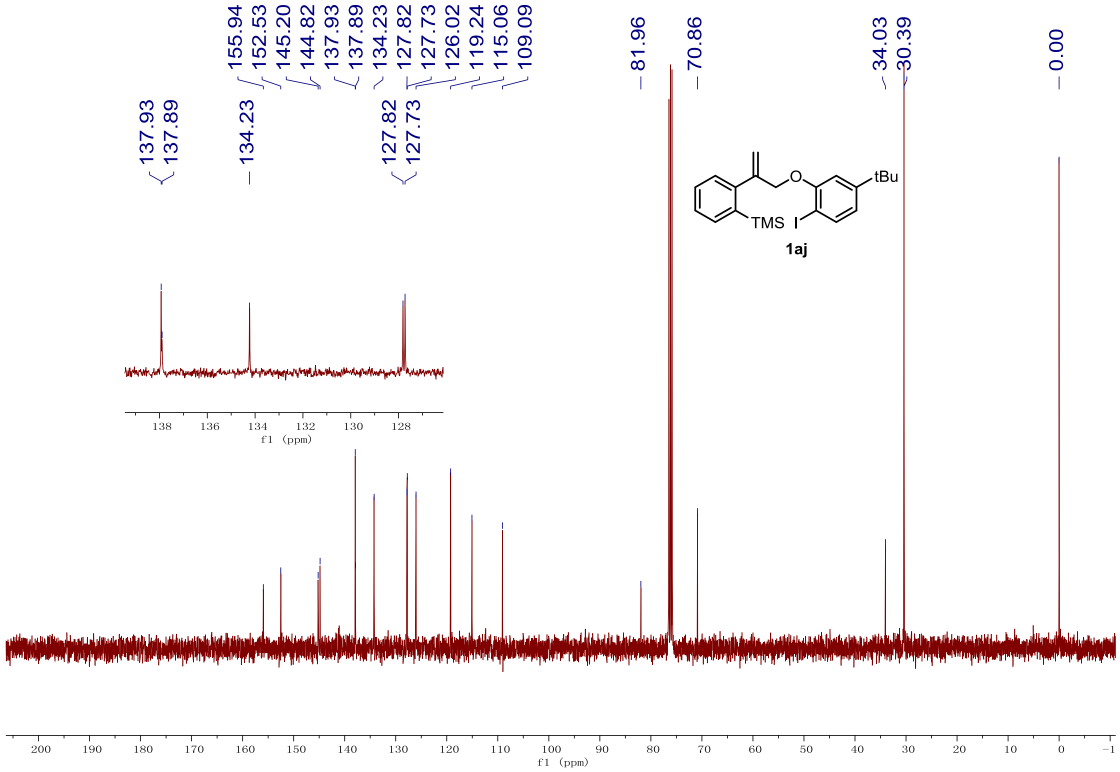

Supplementary Figure 27. ^{13}**C NMR (101 MHz, CDCl$_3$)** spectra of **1aj**

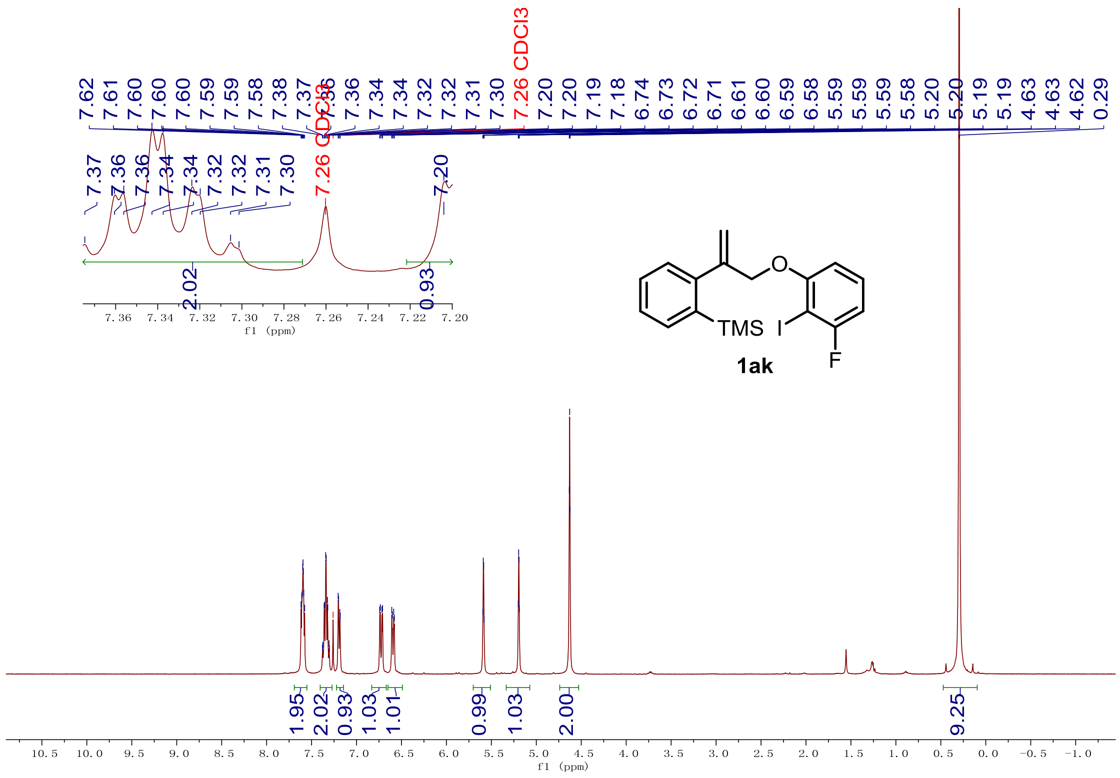

Supplementary Figure 28. ^{1}**H NMR (400 MHz, CDCl$_3$)** spectra of **1ak**

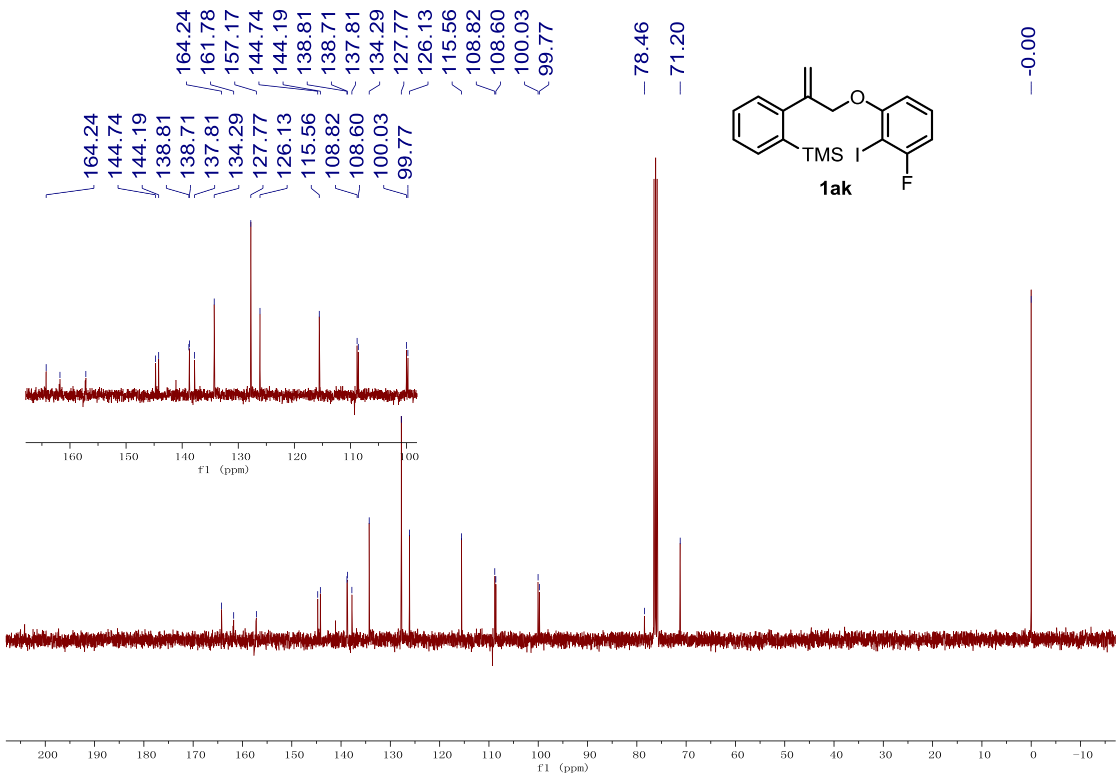

Supplementary Figure 29. ^{13}**C NMR (101 MHz, CDCl$_3$)** spectra of **1ak**

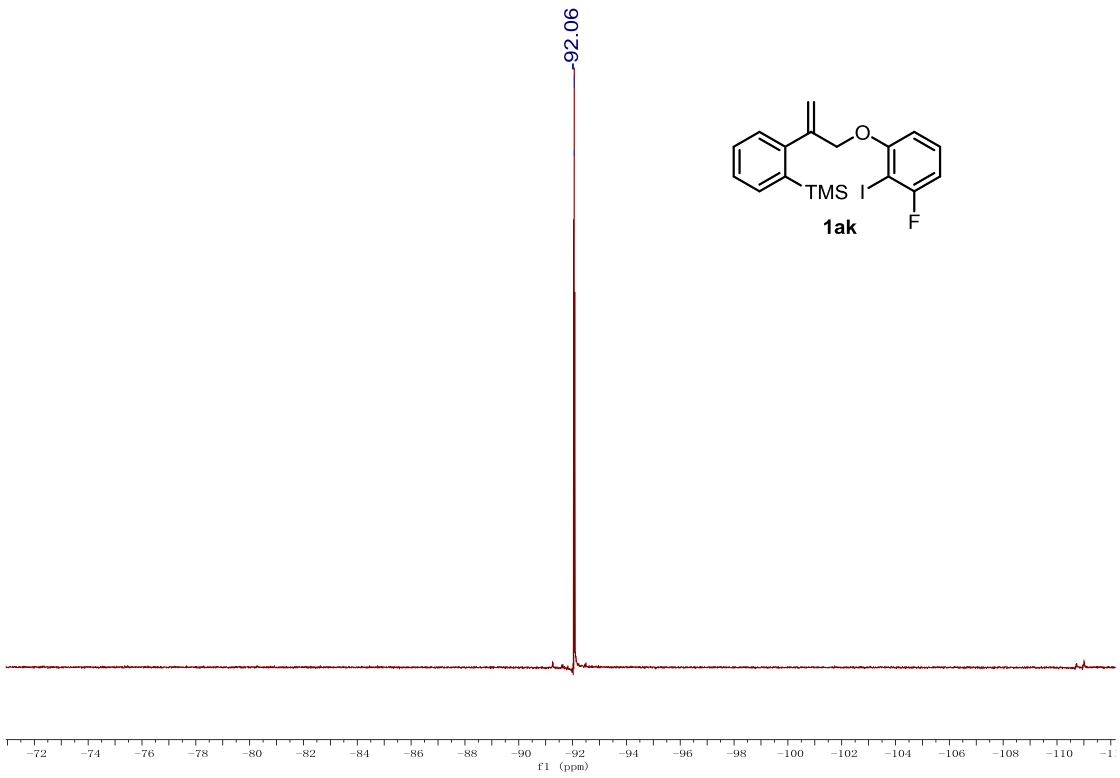

Supplementary Figure 30. ^{19}F NMR (376 MHz, $CDCl_3$) spectra of **1ak**

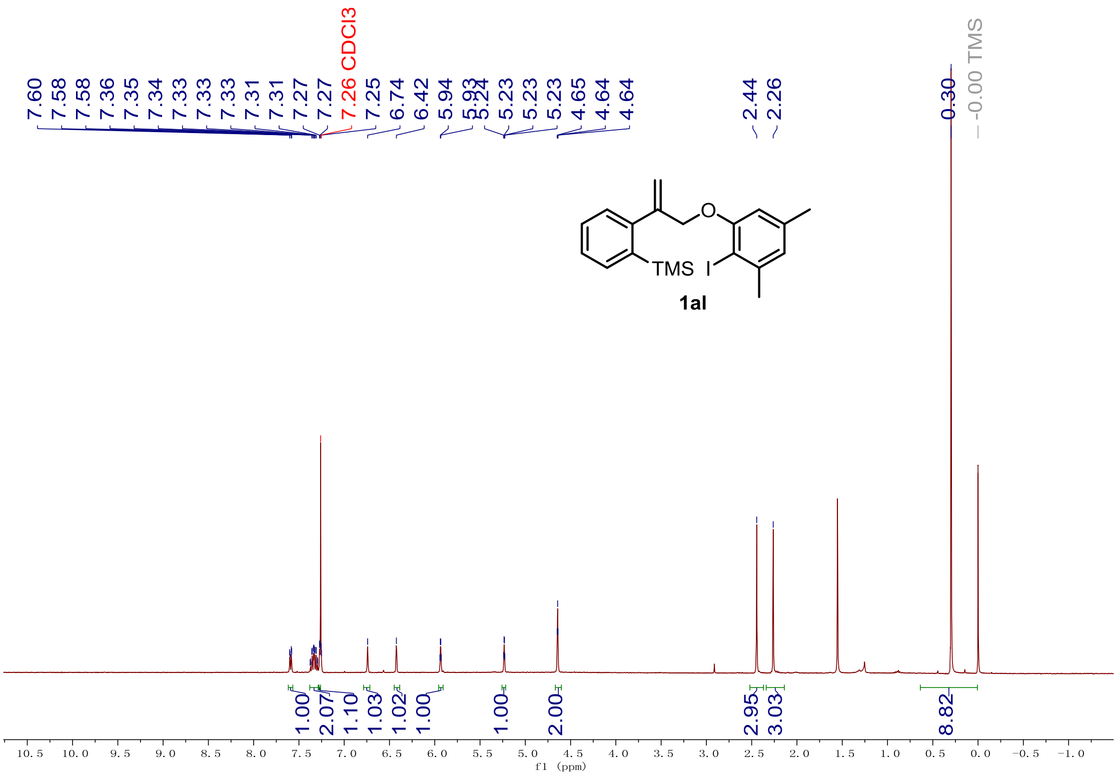

Supplementary Figure 31. ^{1}**H NMR (400 MHz, CDCl$_3$)** spectra of **1al**

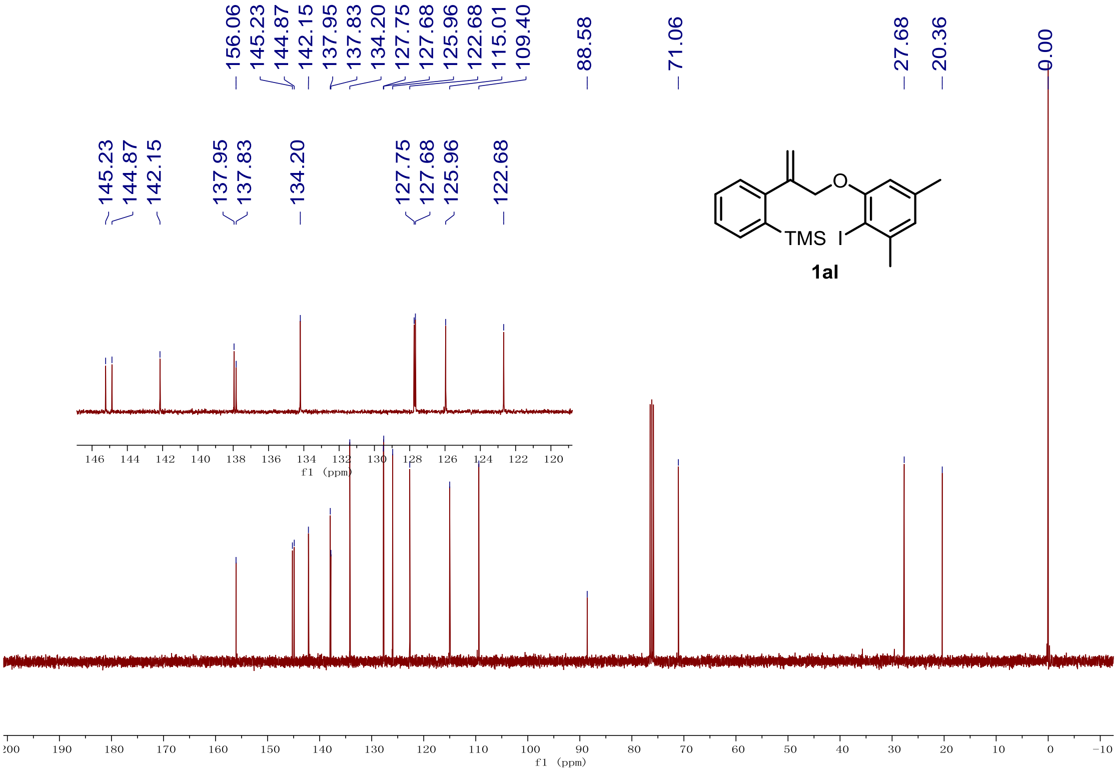

Supplementary Figure 32. ^{13}**C NMR (101 MHz, CDCl$_3$)** spectra of **1al**

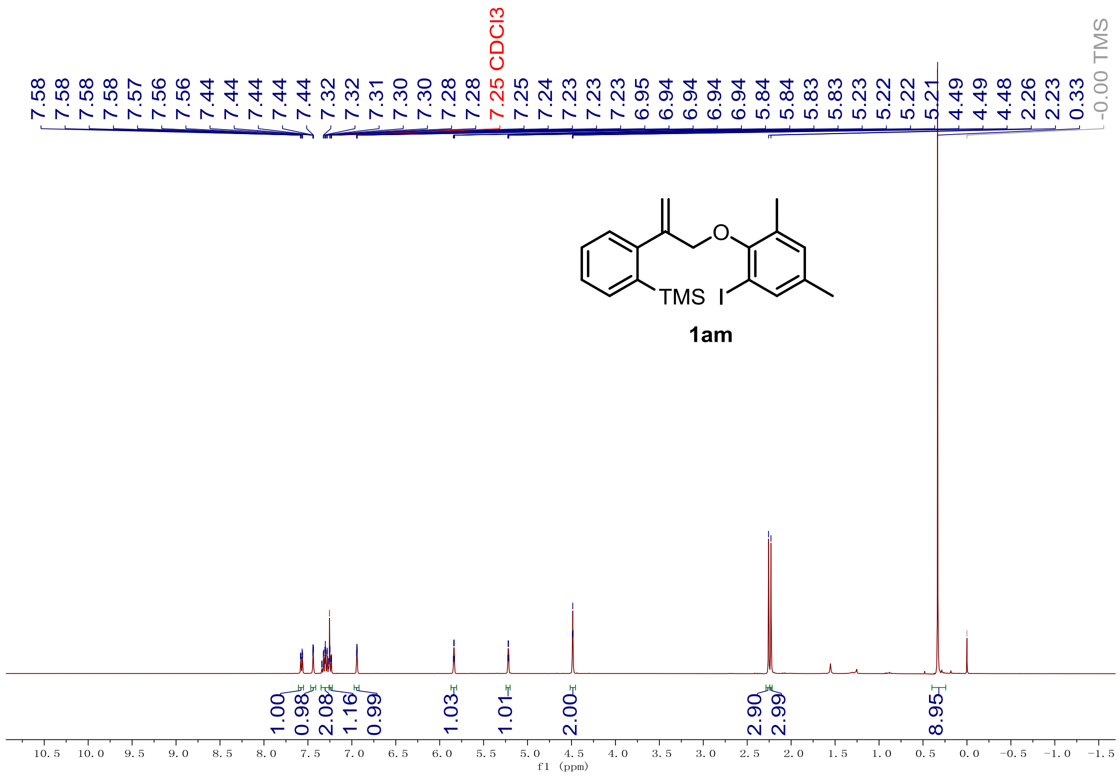

Supplementary Figure 33. ^{1}**H NMR (400 MHz, CDCl$_3$)** spectra of **1am**

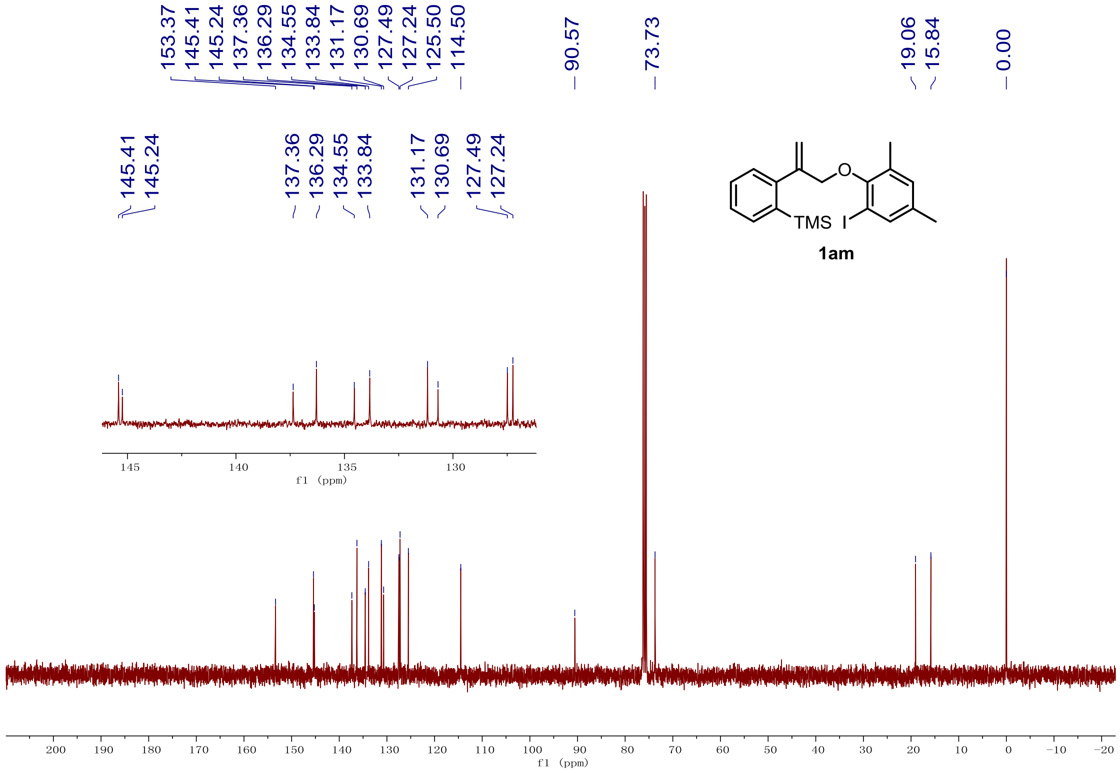

Supplementary Figure 34. ^{13}**C NMR (101 MHz, CDCl$_3$)** spectra of **1am**

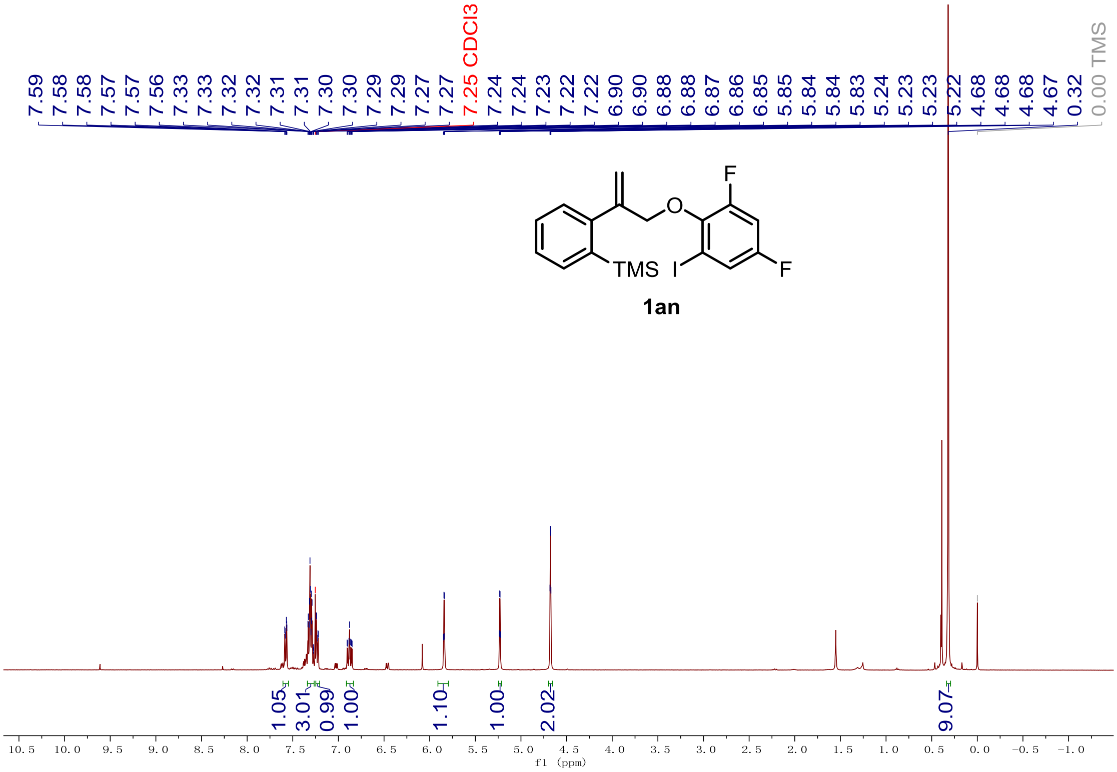

Supplementary Figure 35. ^{1}H NMR (400 MHz, $CDCl_3$) spectra of **1an**

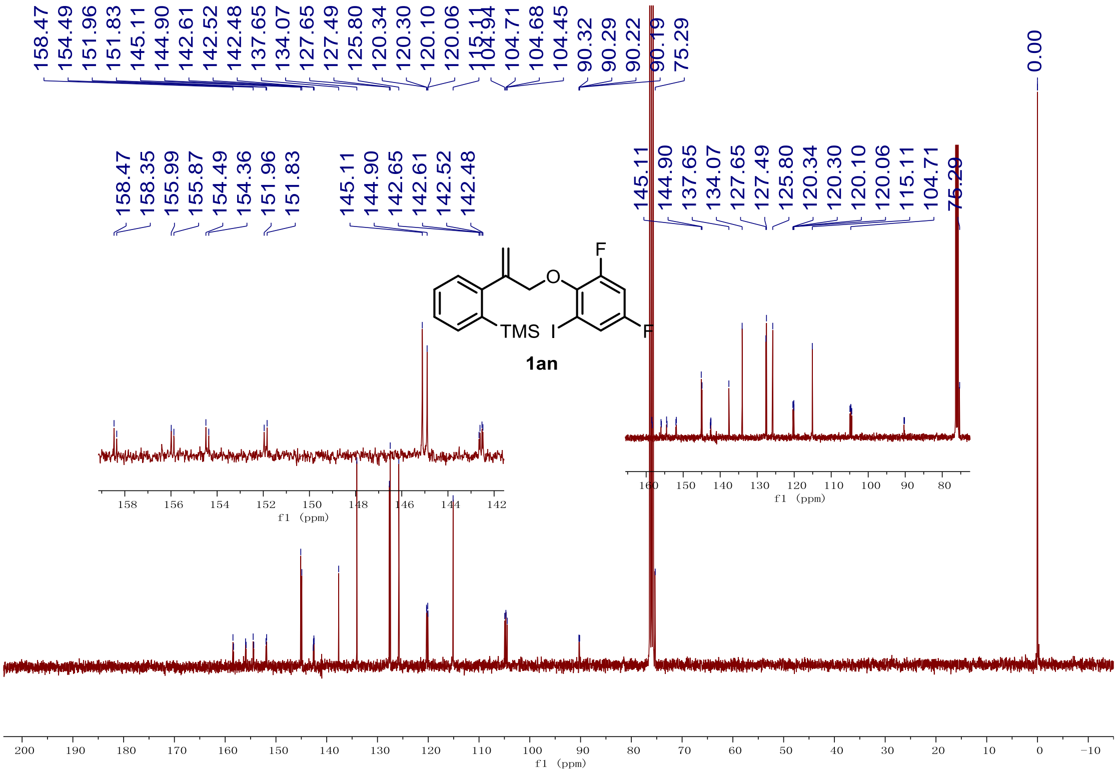

Supplementary Figure 36. ^{13}C NMR (101 MHz, $CDCl_3$) spectra of **1an**

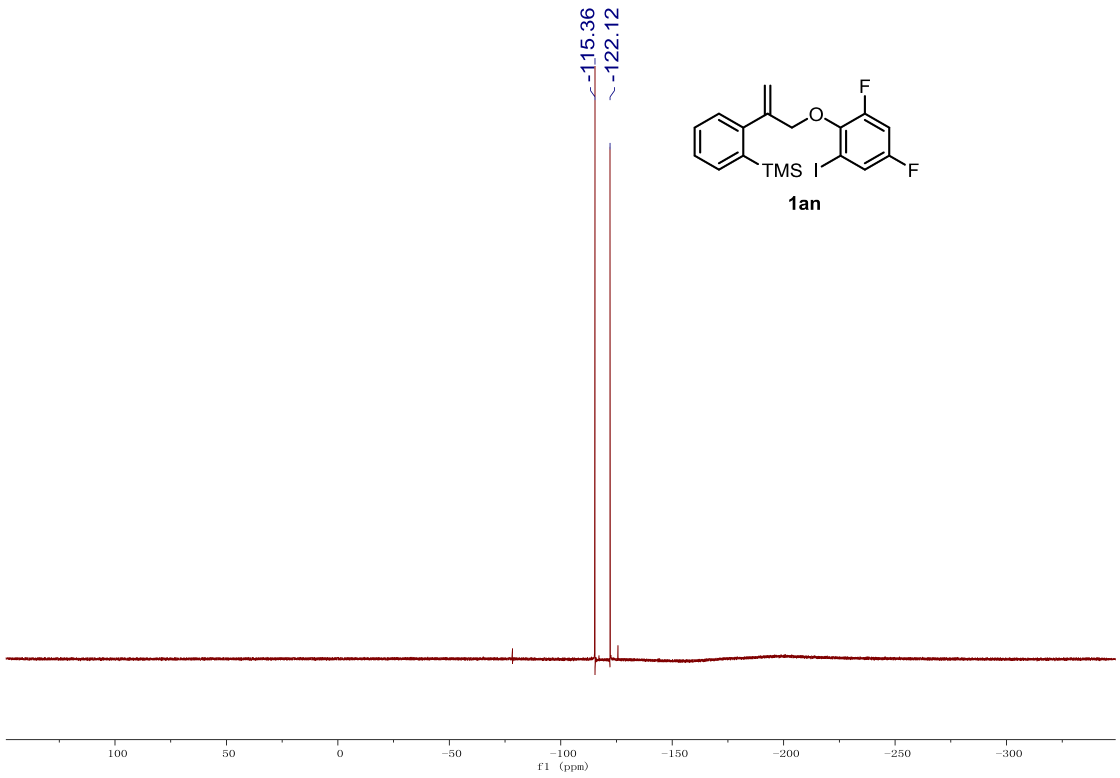

Supplementary Figure 37. ^{19}F NMR (376 MHz, $CDCl_3$) spectra of **1an**

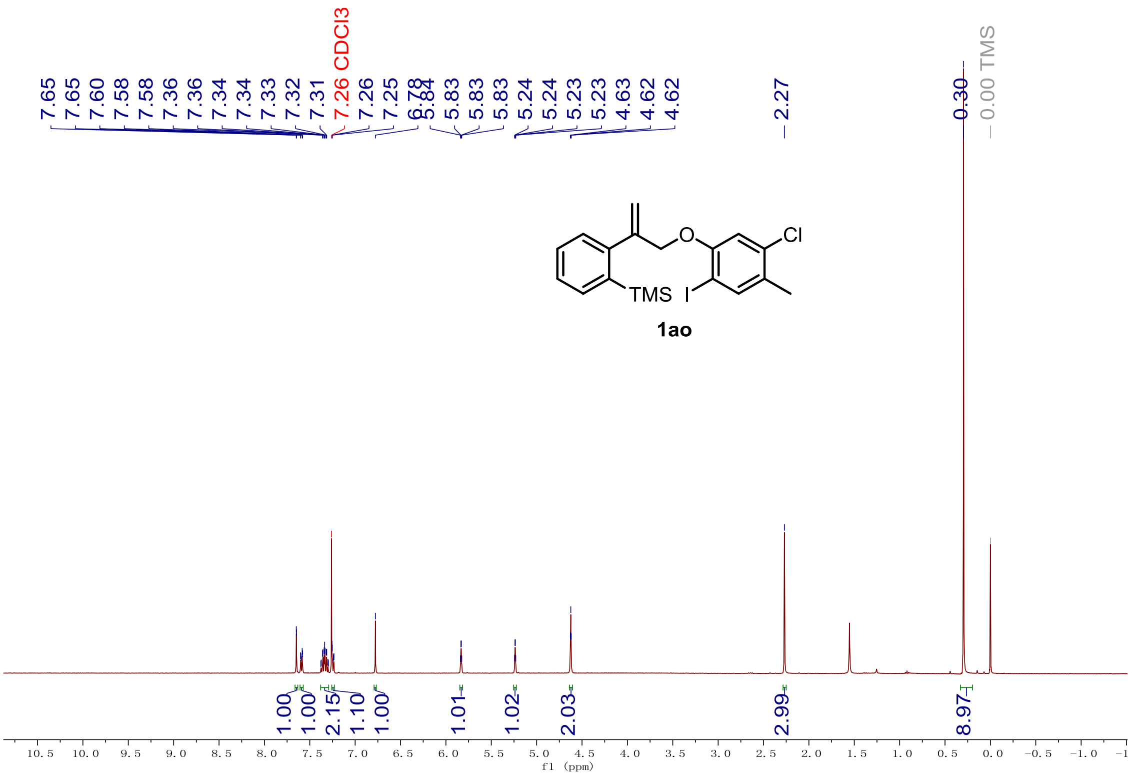

Supplementary Figure 38. **^{1}H NMR (400 MHz, $CDCl_3$)** spectra of **1ao**

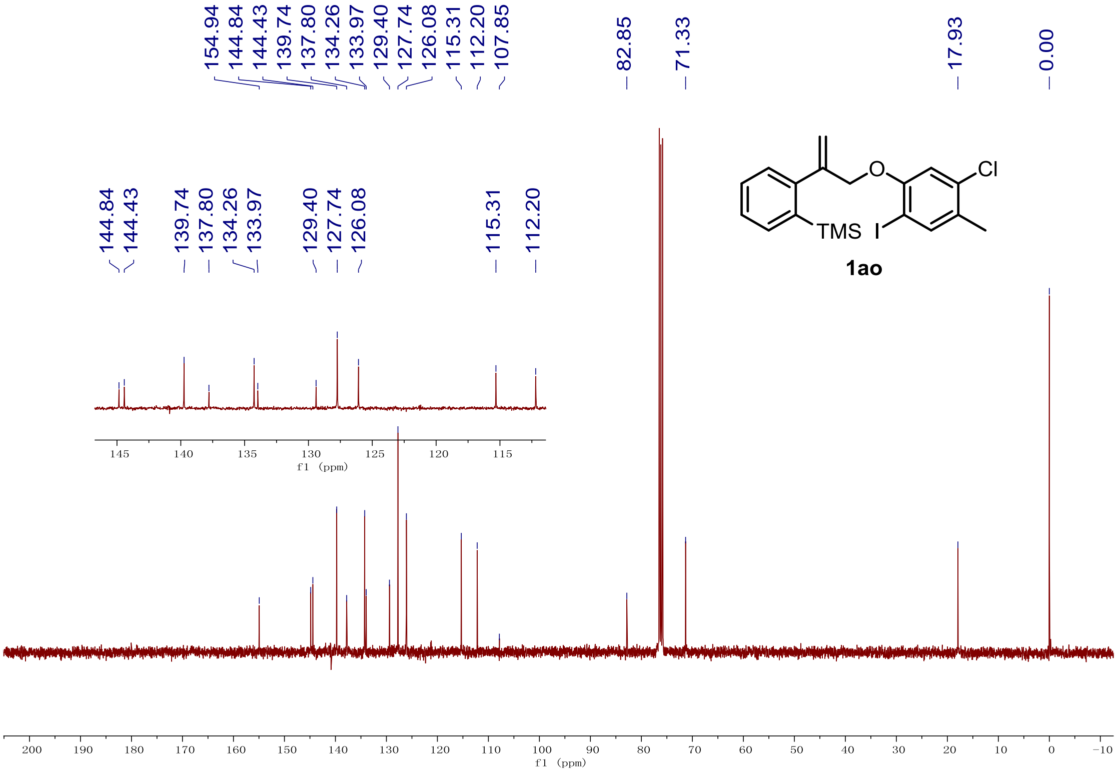

Supplementary Figure 39. **^{13}C NMR (101 MHz, $CDCl_3$)** spectra of **1ao**

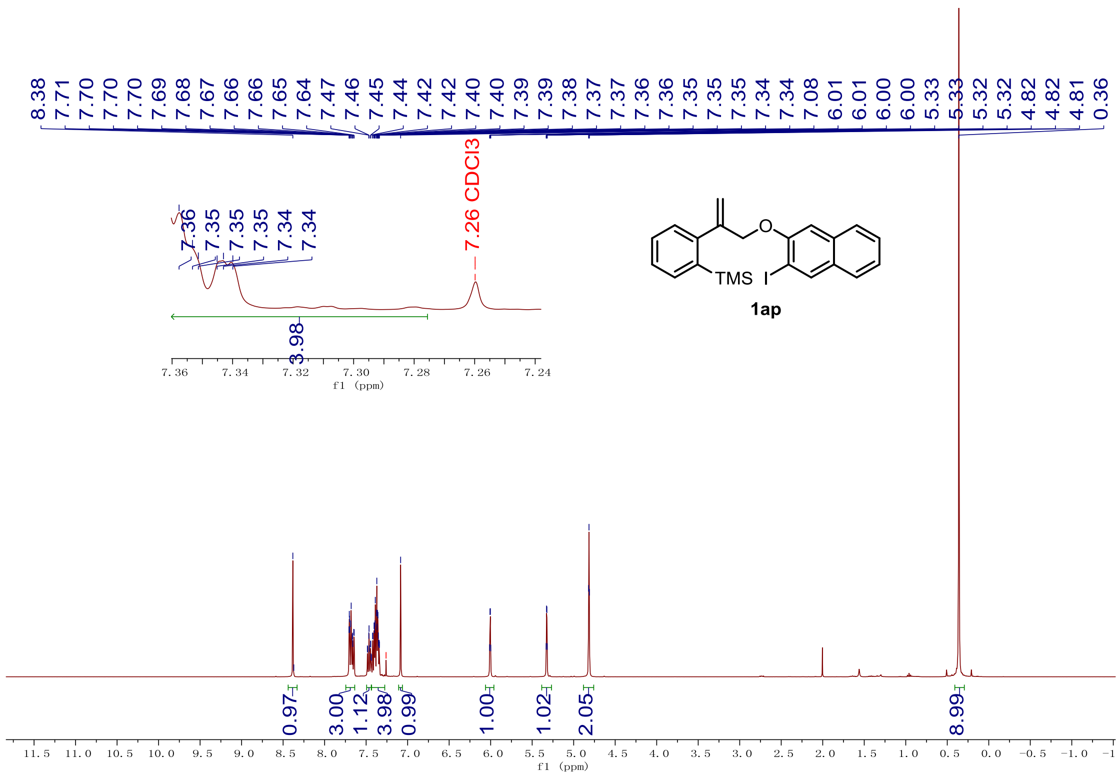

Supplementary Figure 40. ^{1}**H NMR (400 MHz, CDCl$_3$)** spectra of **1ap**

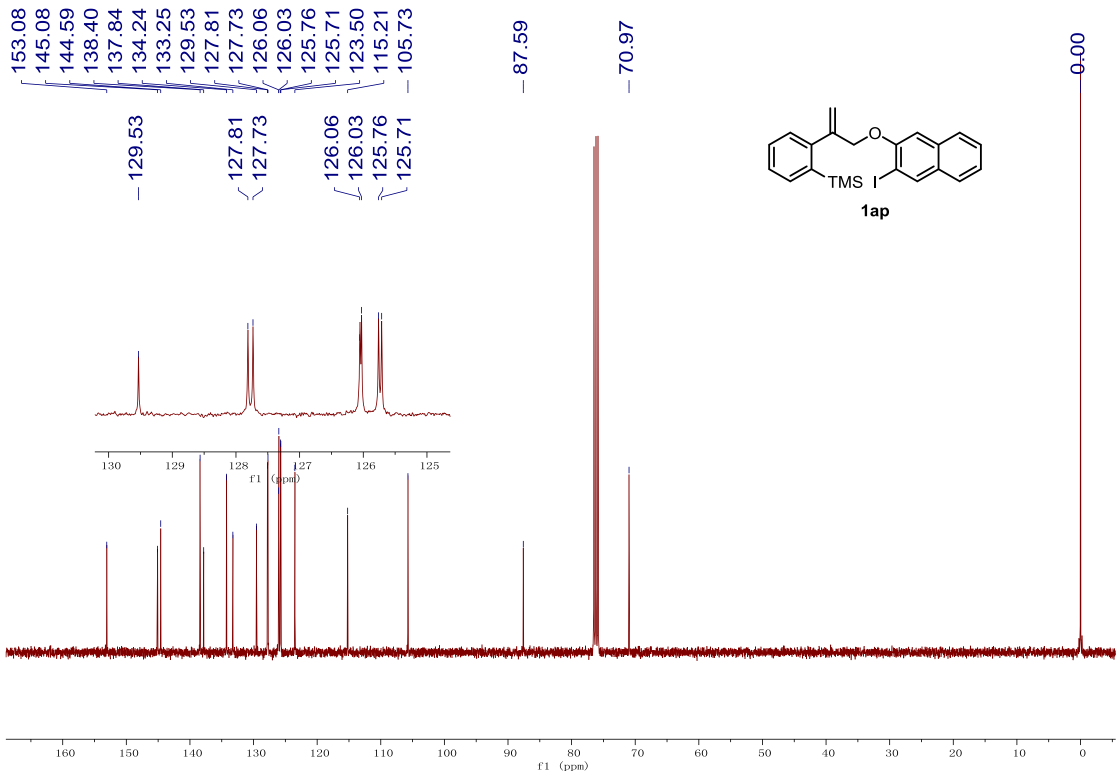

Supplementary Figure 41. ^{13}**C NMR (101 MHz, CDCl$_3$)** spectra of **1ap**

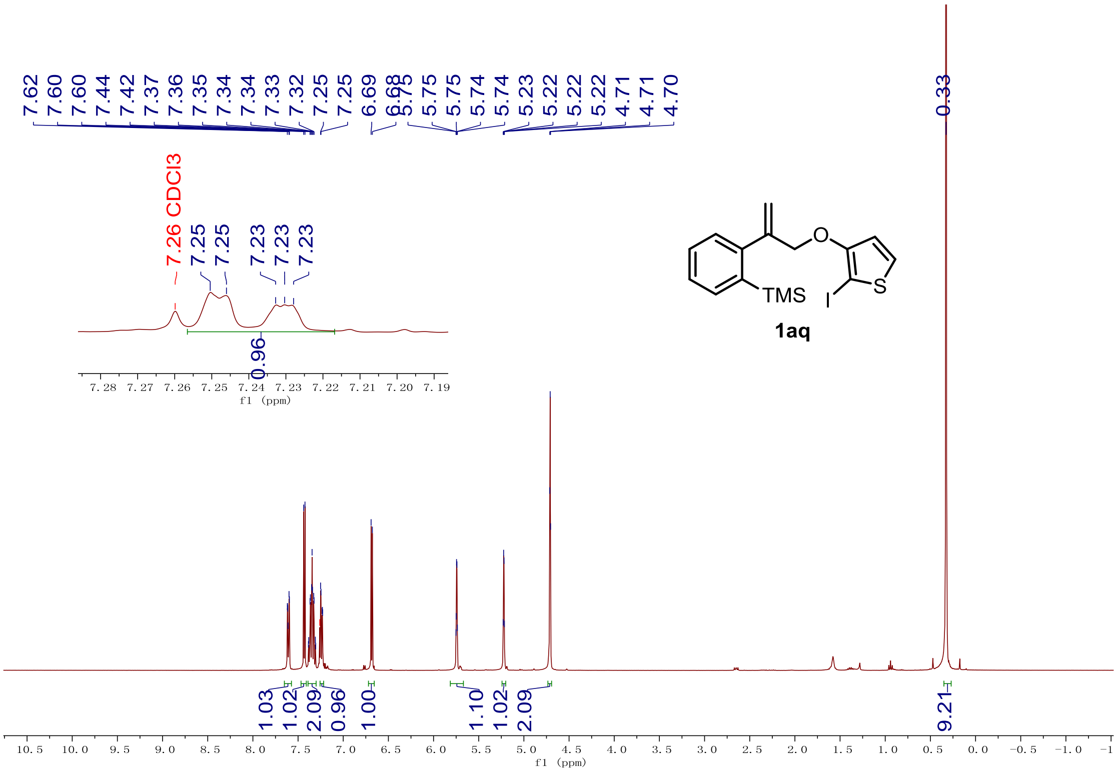

Supplementary Figure 42. **^{1}H NMR (400 MHz, $CDCl_3$)** spectra of **1aq**

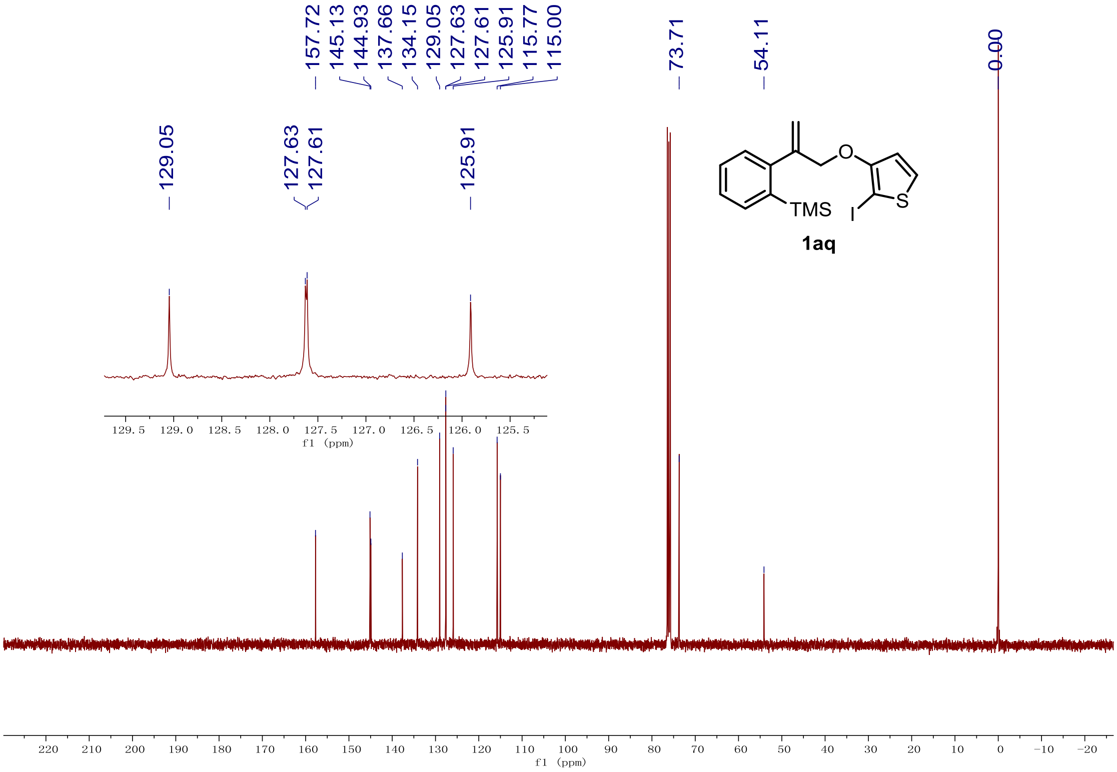

Supplementary Figure 43. **^{13}C NMR (101 MHz, $CDCl_3$)** spectra of **1aq**

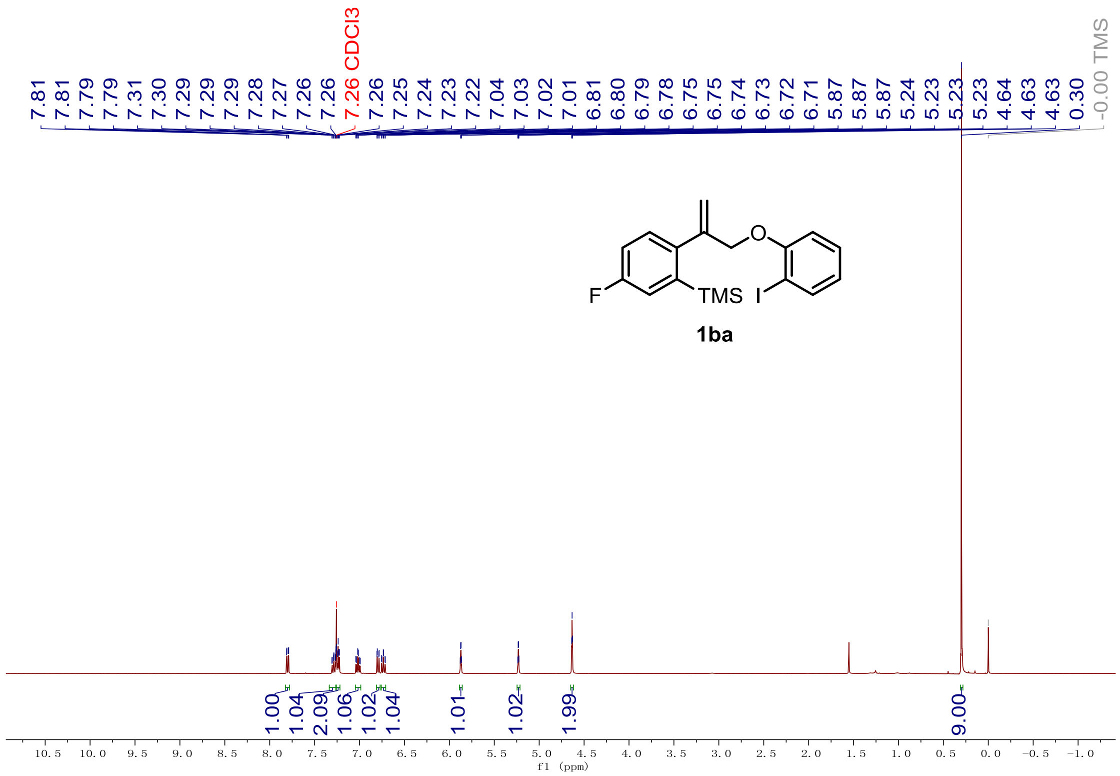

Supplementary Figure 44. ^{1}**H NMR (400 MHz, CDCl$_3$)** spectra of **1ba**

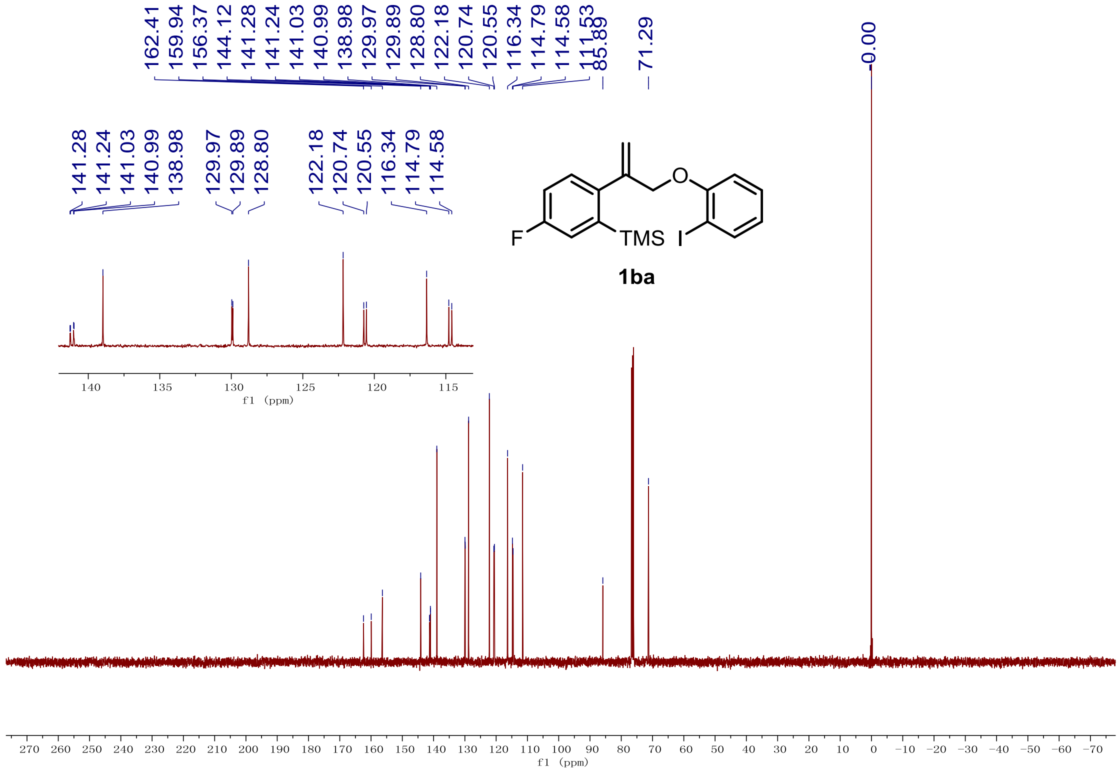

Supplementary Figure 45. ^{13}**C NMR (101 MHz, CDCl$_3$)** spectra of **1ba**

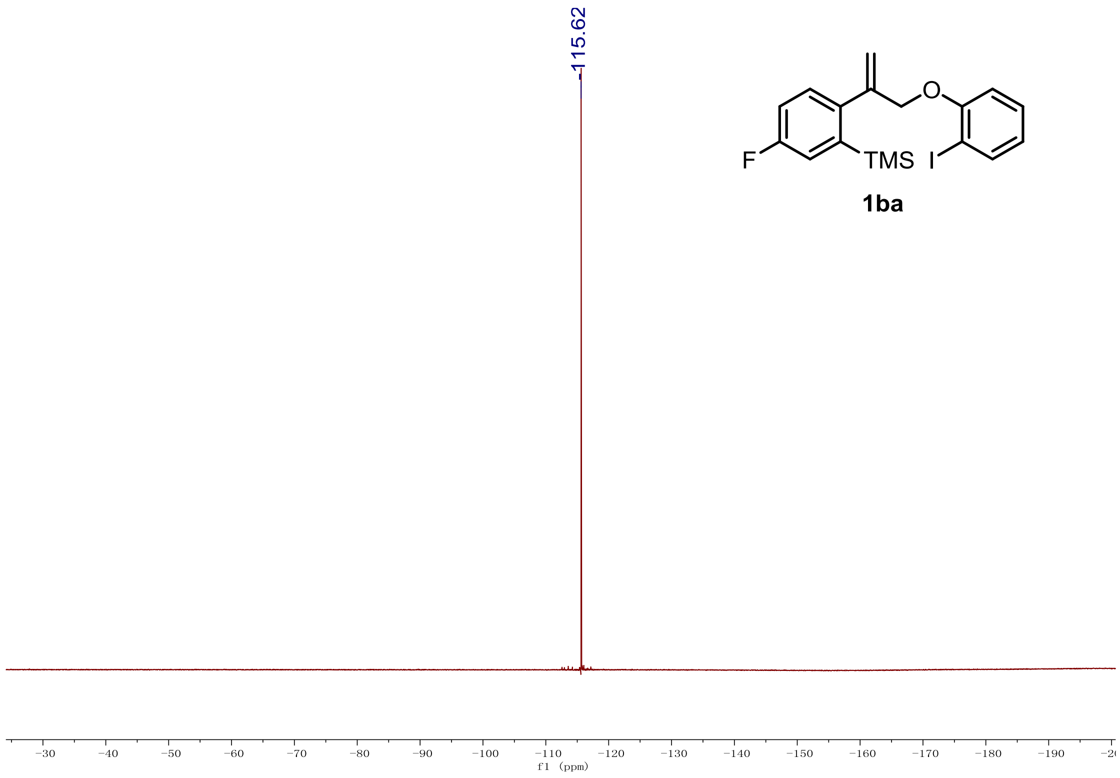

Supplementary Figure 46. ^{19}F NMR (376 MHz, CDCl$_3$) spectra of **1ba**

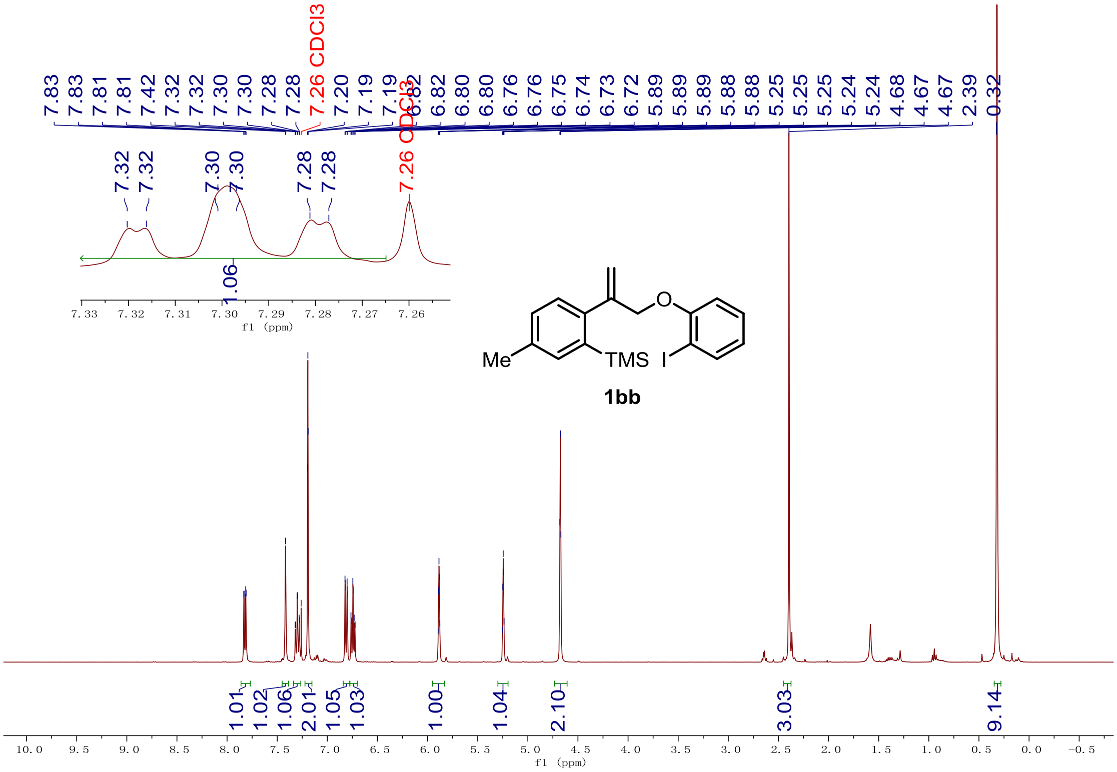

Supplementary Figure 47. ^{1}**H NMR (400 MHz, CDCl$_3$)** spectra of **1bb**

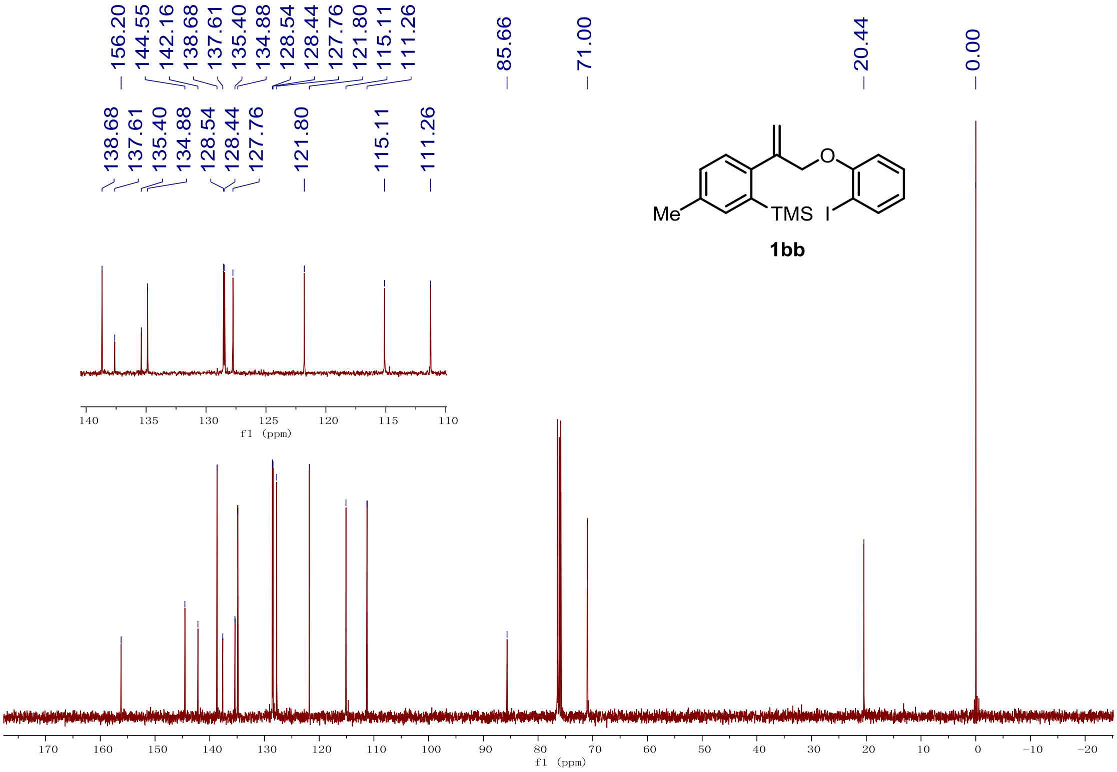

Supplementary Figure 48. ^{13}**C NMR (101 MHz, CDCl$_3$)** spectra of **1bb**

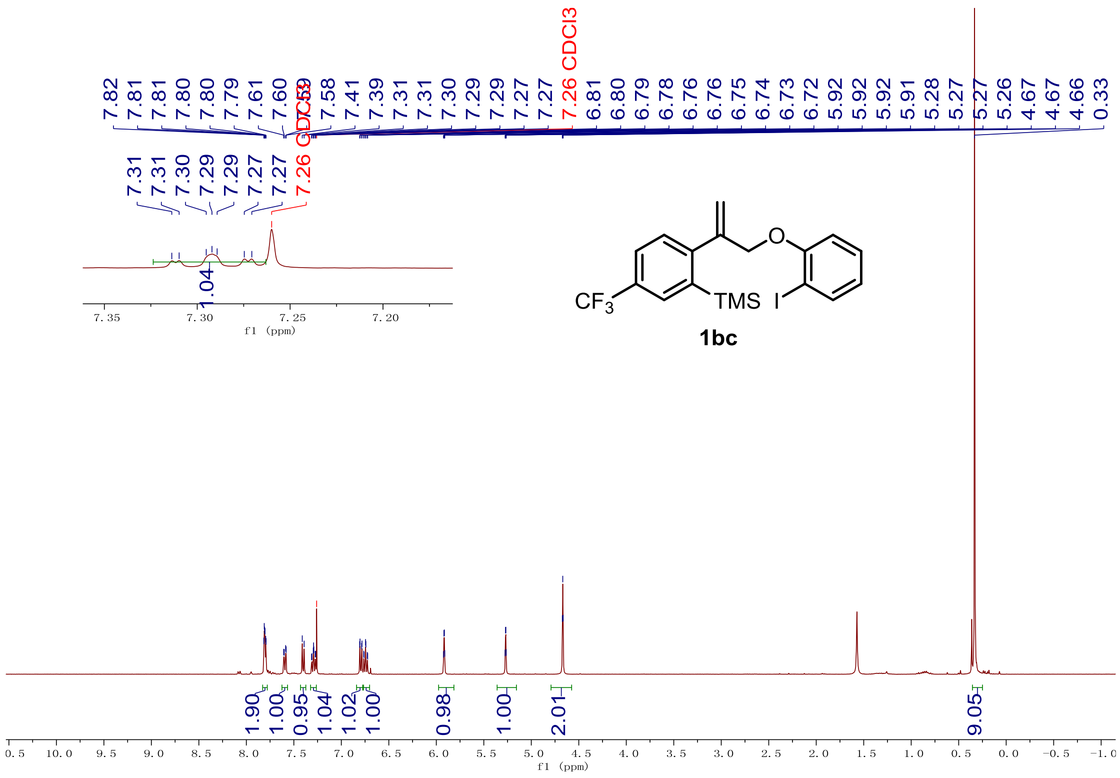

Supplementary Figure 49. ^{1}**H NMR (400 MHz, CDCl**$_3$**)** spectra of **1bc**

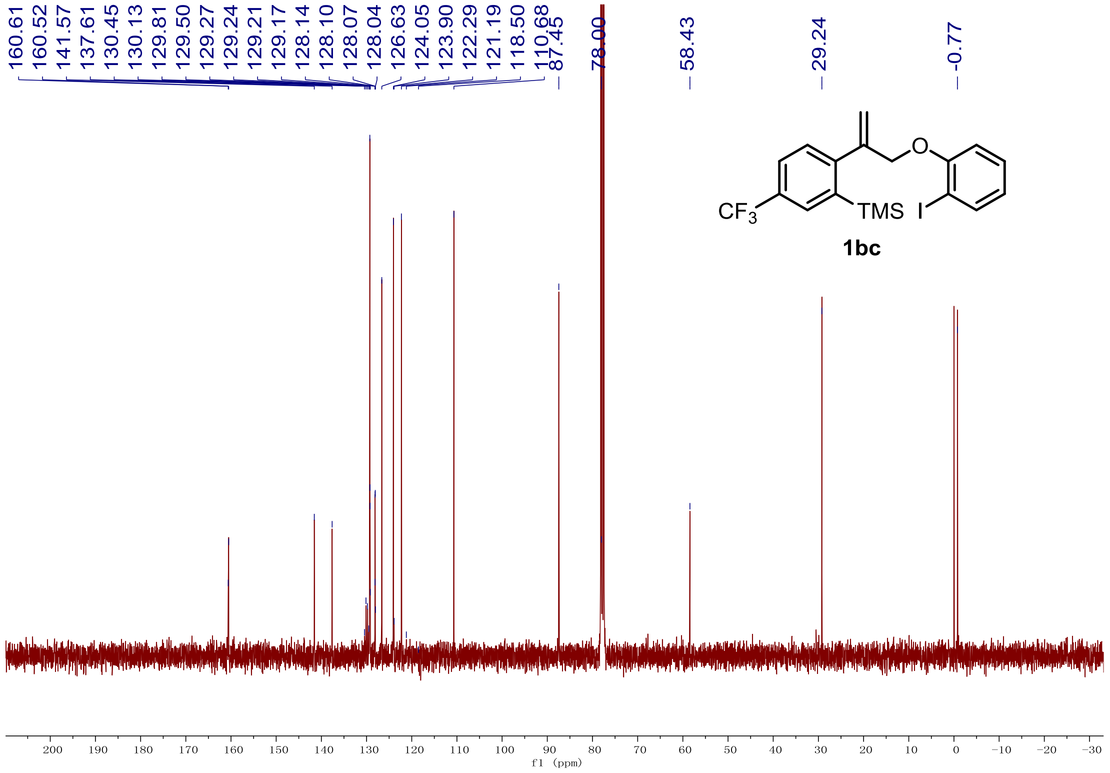

Supplementary Figure 50. ^{13}**C NMR (101 MHz, CDCl**$_3$**)** spectra of **1bc**

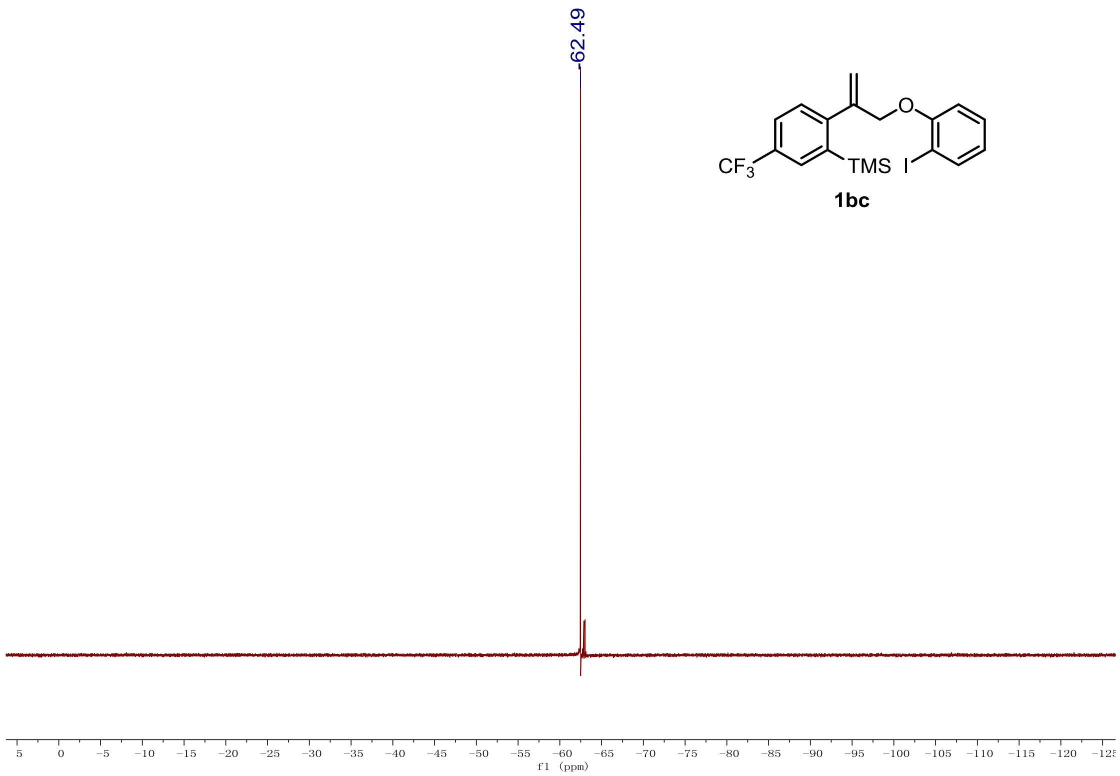

Supplementary Figure 51. **^{19}F NMR (376 MHz, $CDCl_3$)** spectra of **1bc**

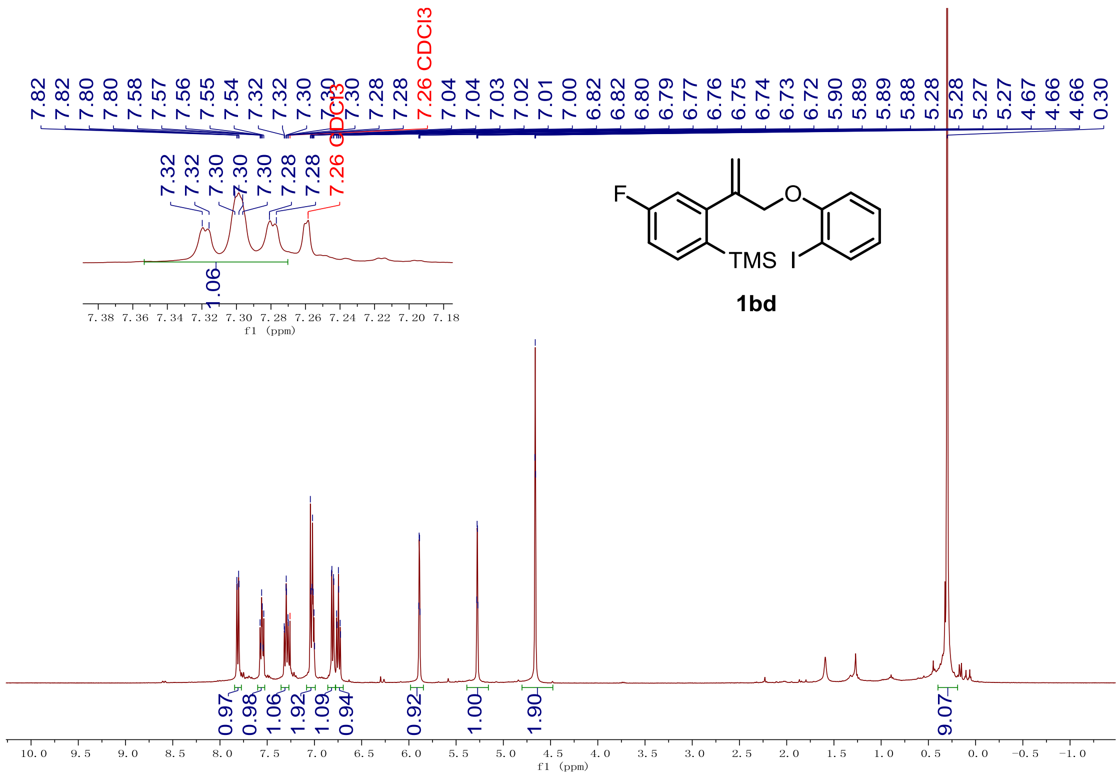

Supplementary Figure 52. ^{1}H NMR (400 MHz, $CDCl_3$) spectra of **1bd**

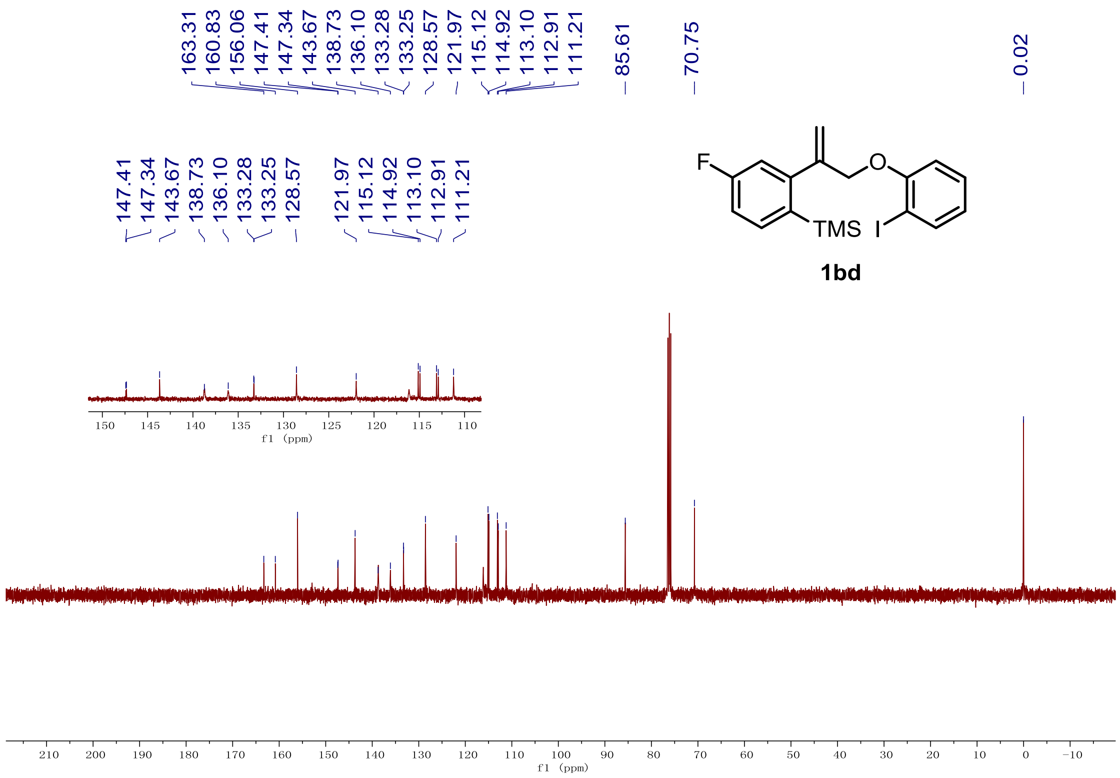

Supplementary Figure 53. ^{13}C NMR (101 MHz, $CDCl_3$) spectra of **1bd**

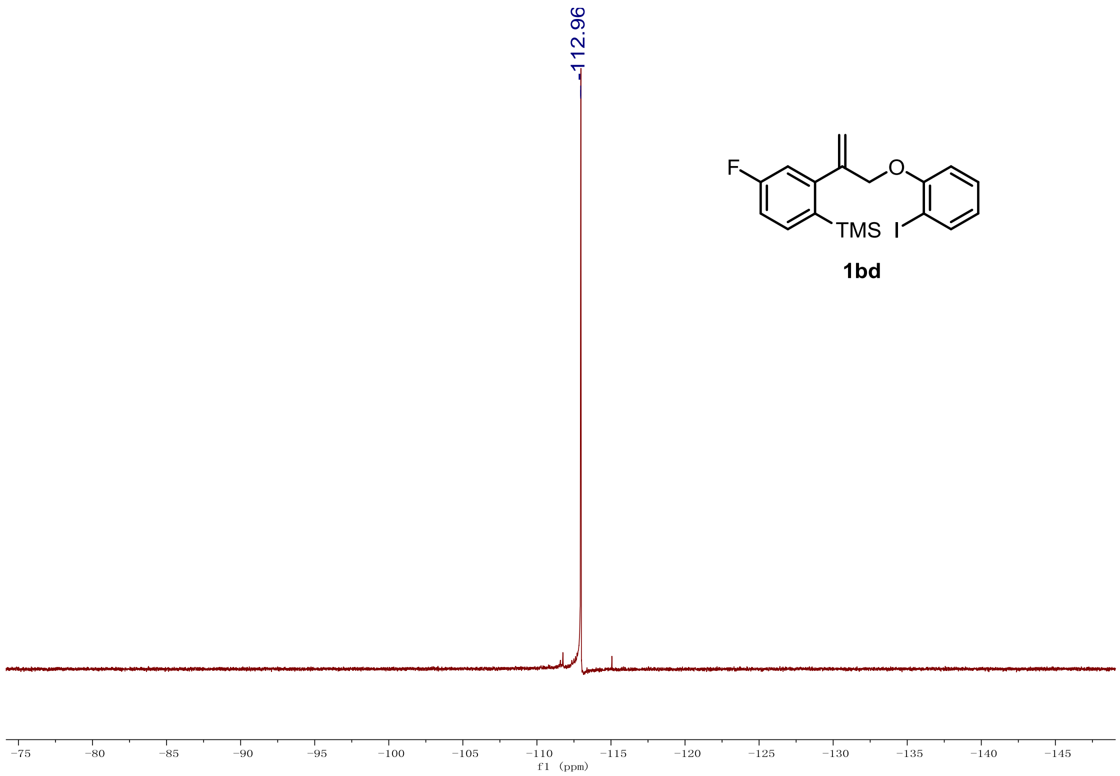

Supplementary Figure 54. ^{19}F NMR (376 MHz, $CDCl_3$) spectra of **1bd**

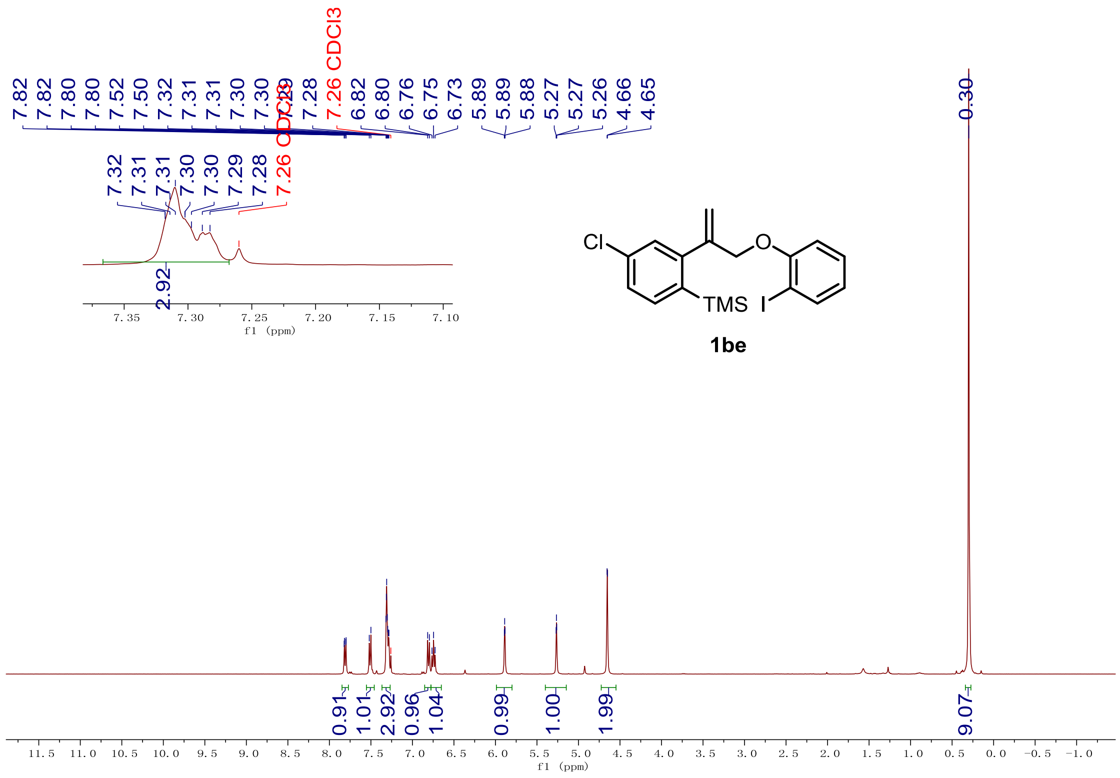

Supplementary Figure 55. ^{1}**H NMR (400 MHz, CDCl$_3$)** spectra of **1be**

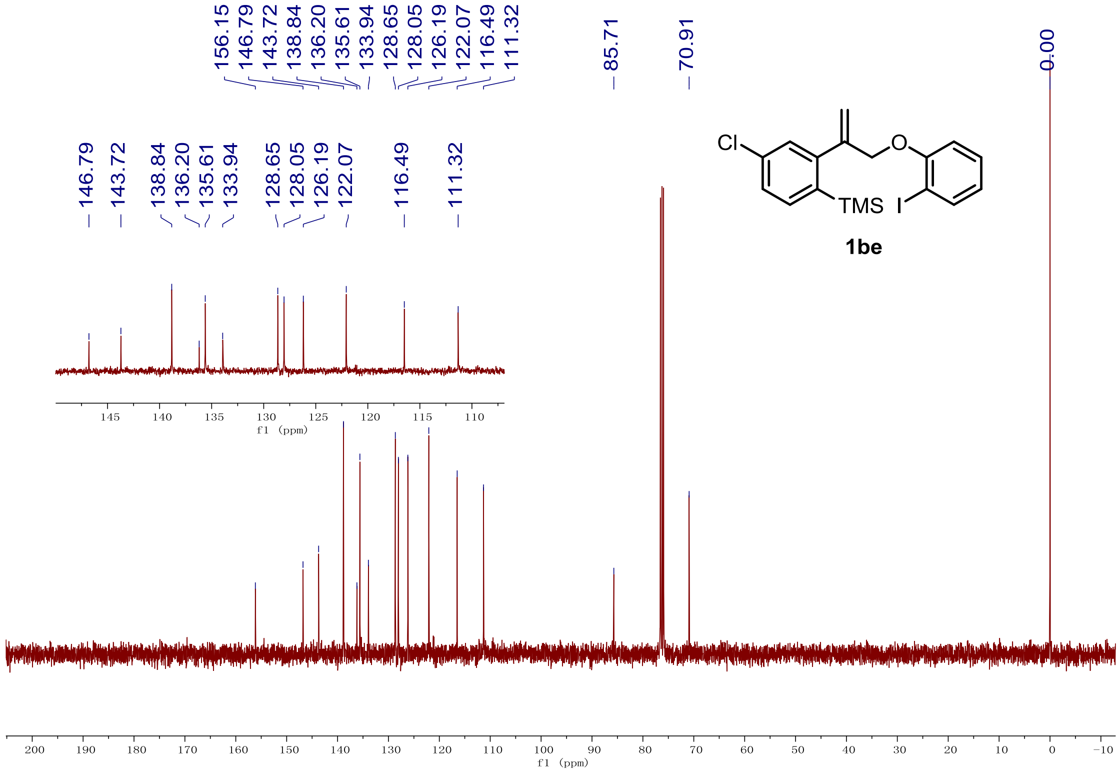

Supplementary Figure 56. ^{13}**C NMR (101 MHz, CDCl$_3$)** spectra of **1be**

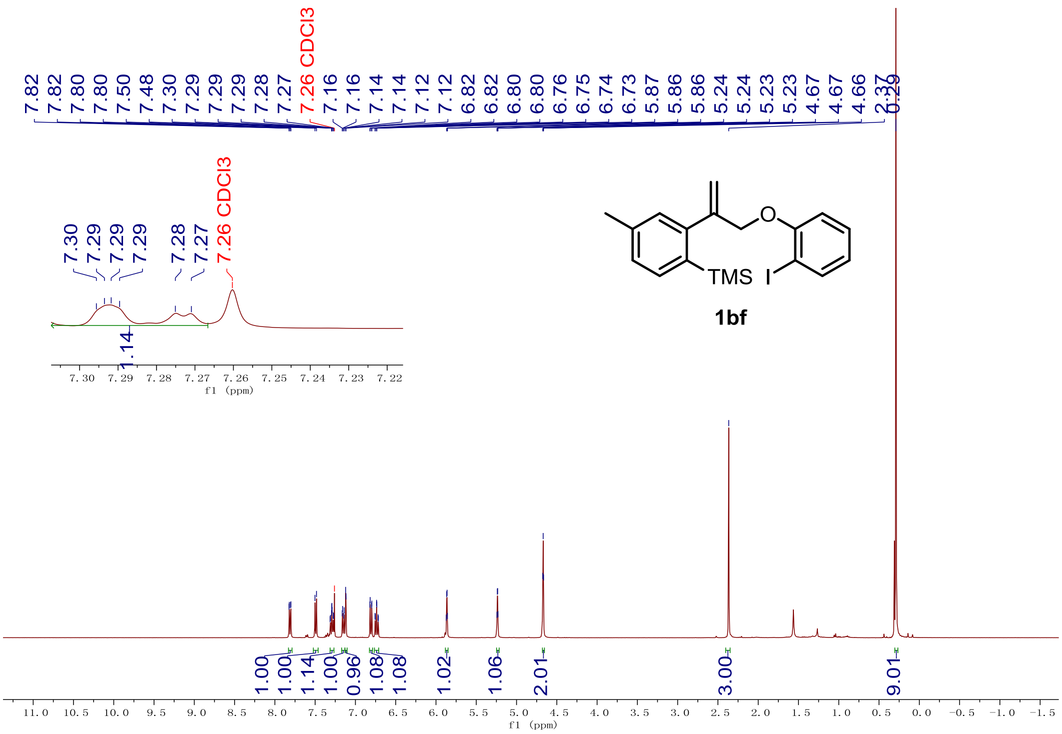

Supplementary Figure 57. ^{1}**H NMR (400 MHz, CDCl$_3$)** spectra of **1bf**

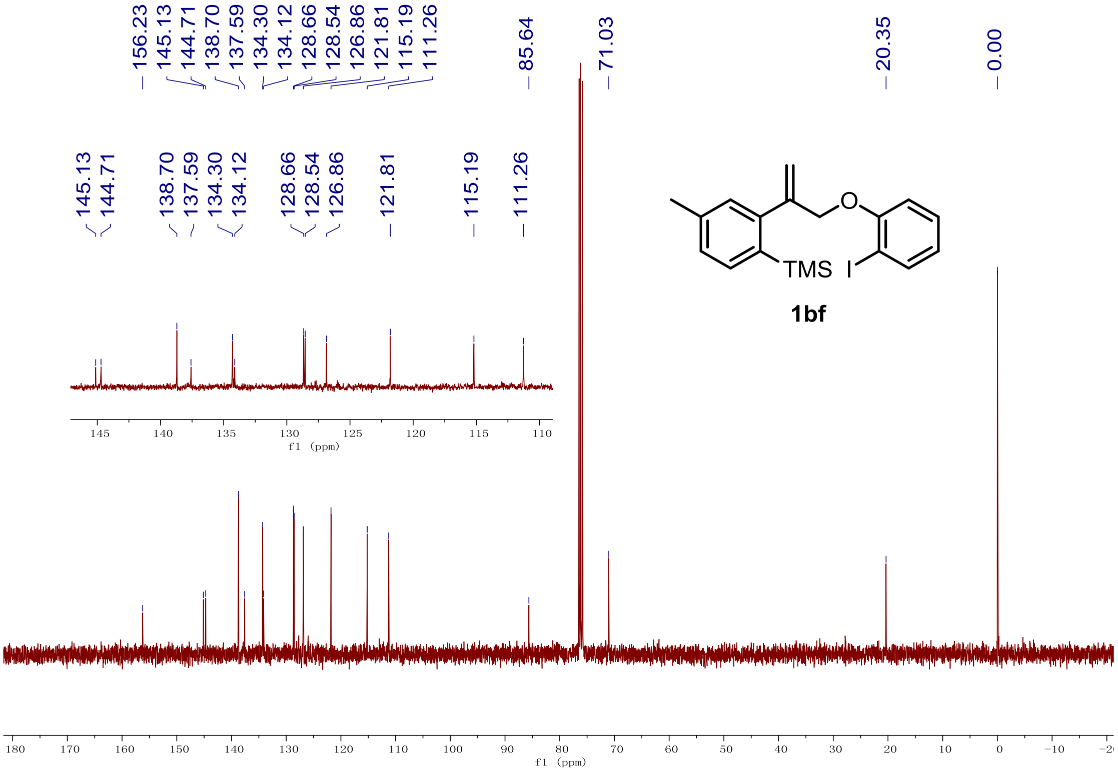

Supplementary Figure 58. ^{13}**C NMR (101 MHz, CDCl$_3$)** spectra of **1bf**

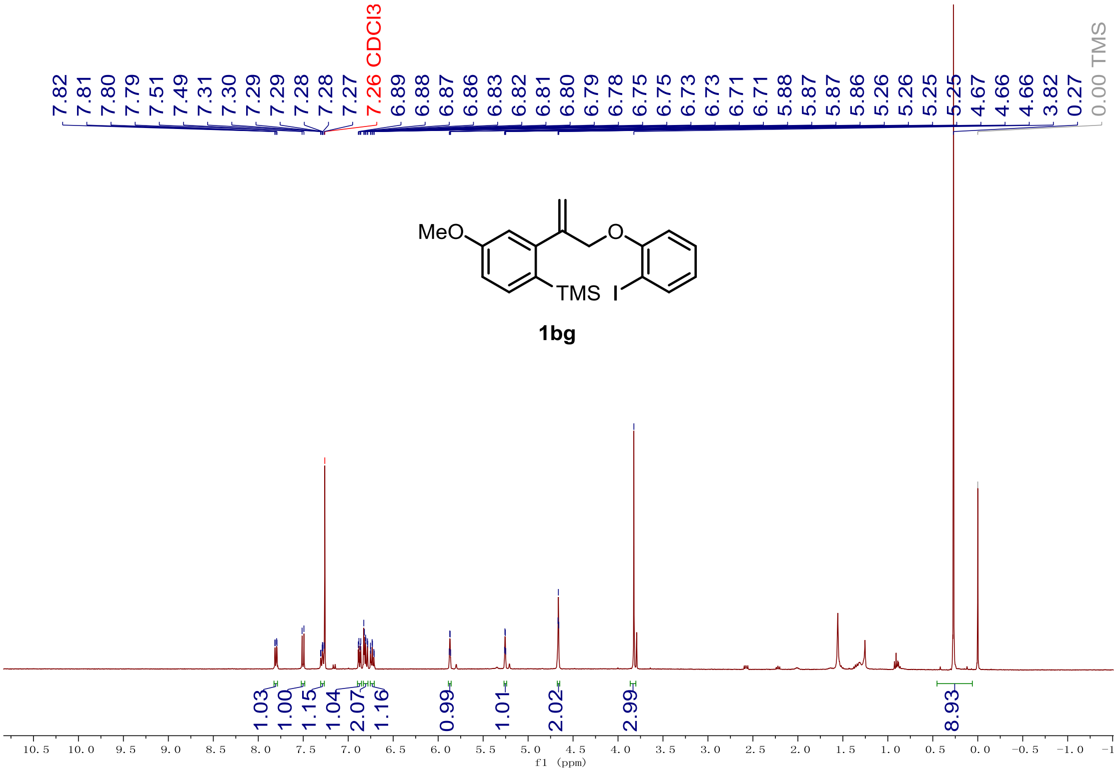

Supplementary Figure 59. **^{1}H NMR (400 MHz, $CDCl_3$)** spectra of **1bg**

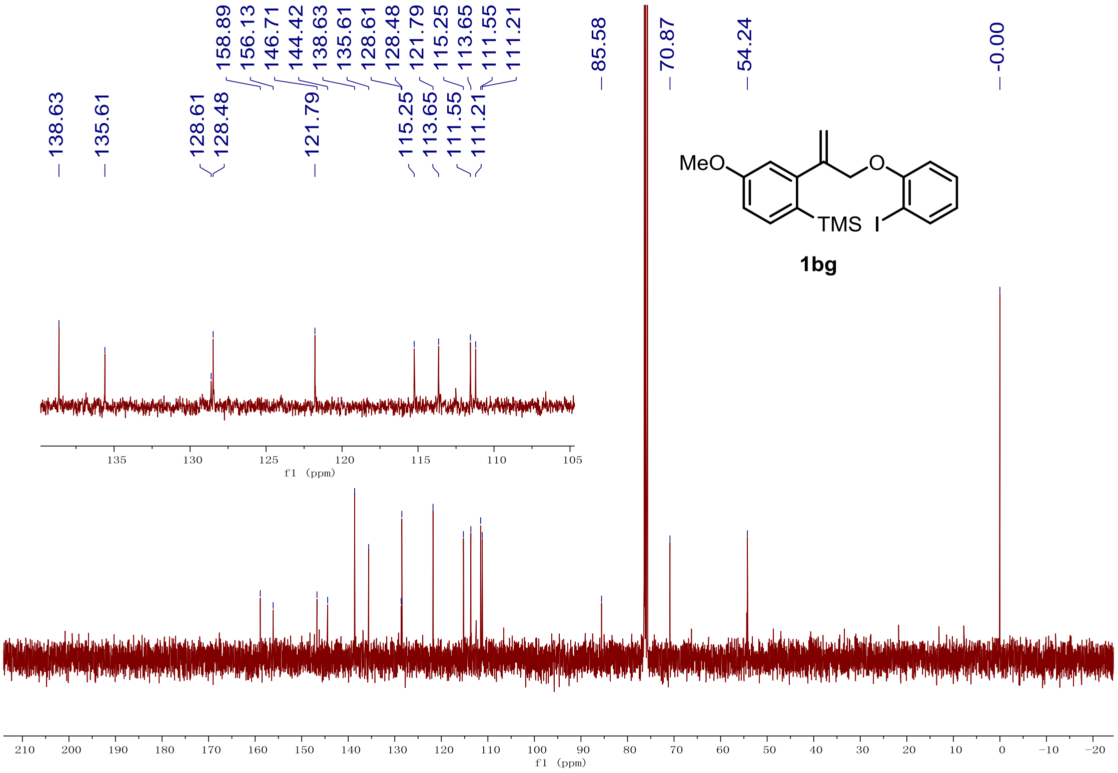

Supplementary Figure 60. **^{13}C NMR (101 MHz, $CDCl_3$)** spectra of **1bg**

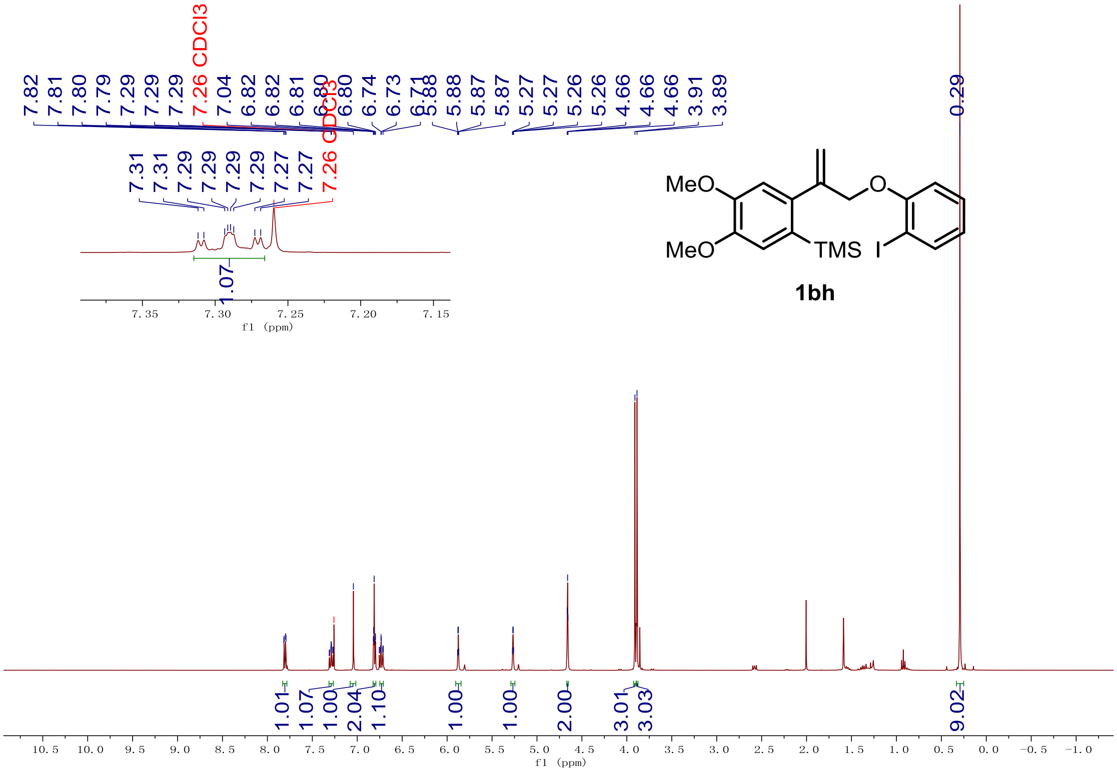

Supplementary Figure 61. ^{1}**H NMR (400 MHz, CDCl$_3$)** spectra of **1bh**

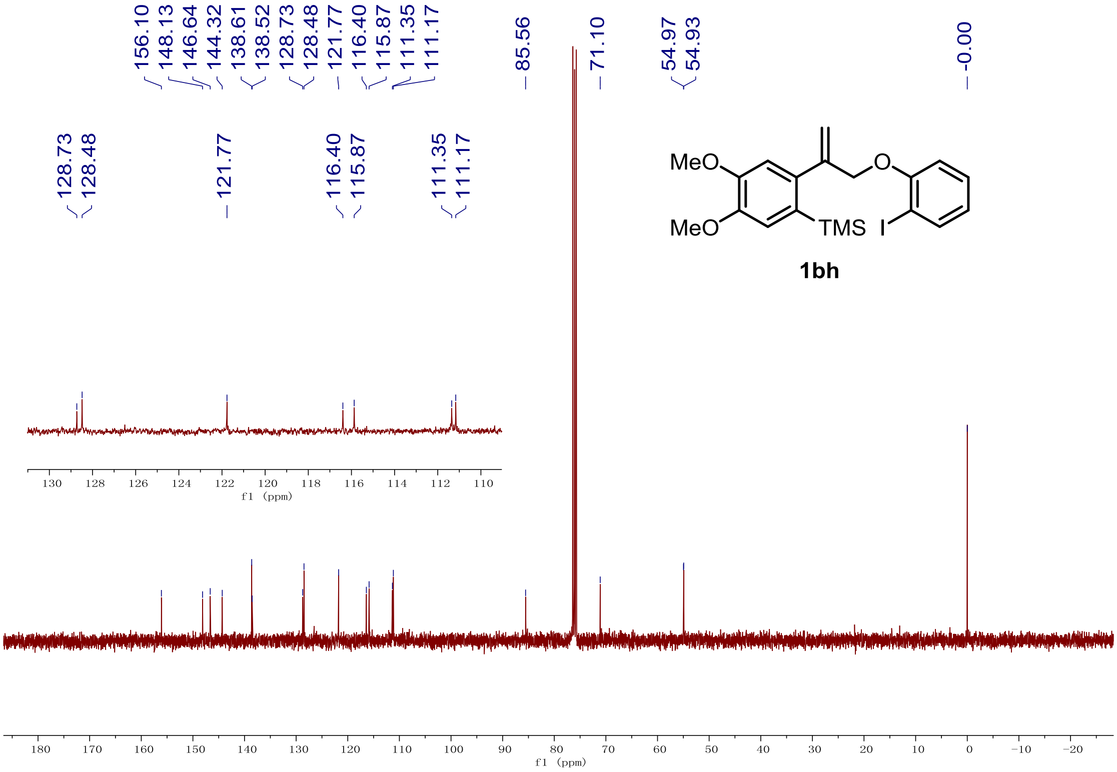

Supplementary Figure 62. ^{13}**C NMR (101 MHz, CDCl$_3$)** spectra of **1bh**

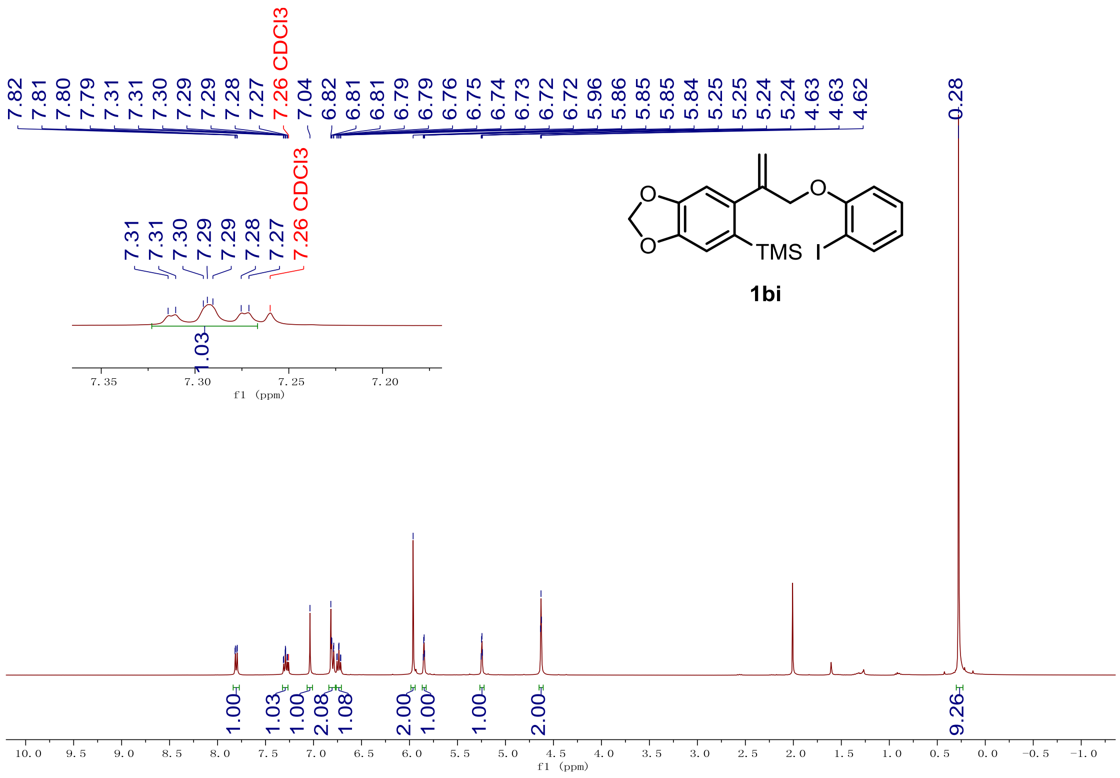

Supplementary Figure 63. ^{1}H NMR (400 MHz, $CDCl_3$) spectra of **1bi**

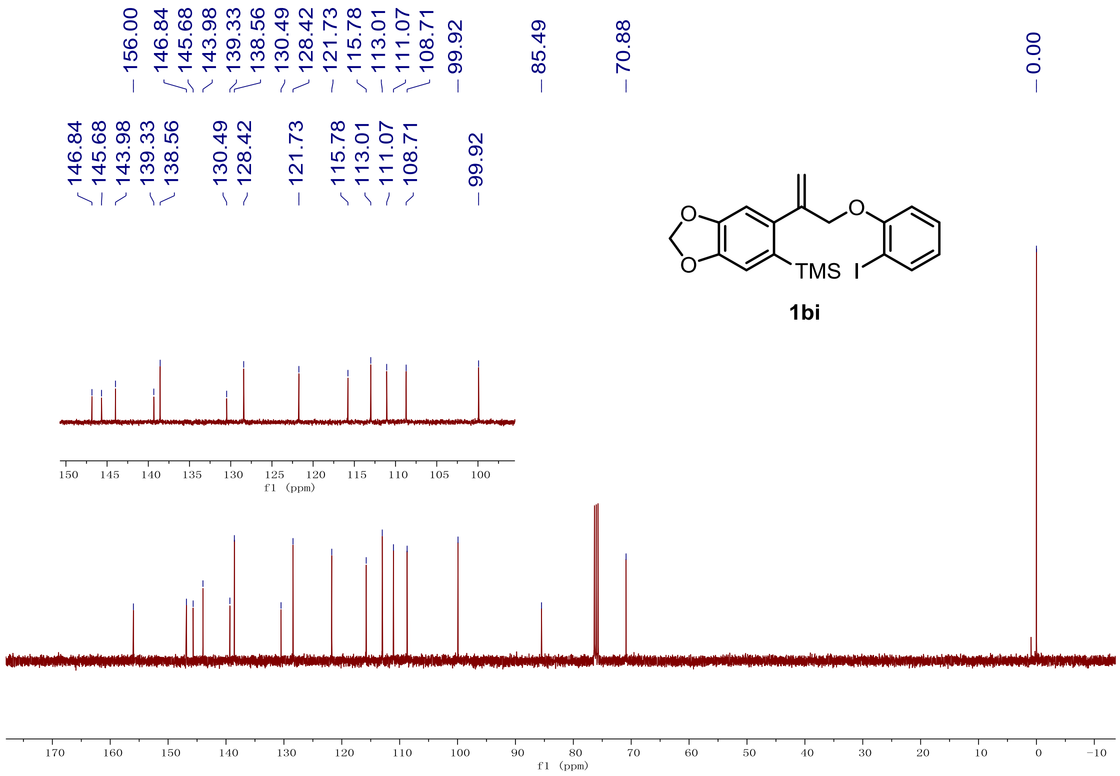

Supplementary Figure 64. ^{13}C NMR (101 MHz, $CDCl_3$) spectra of **1bi**

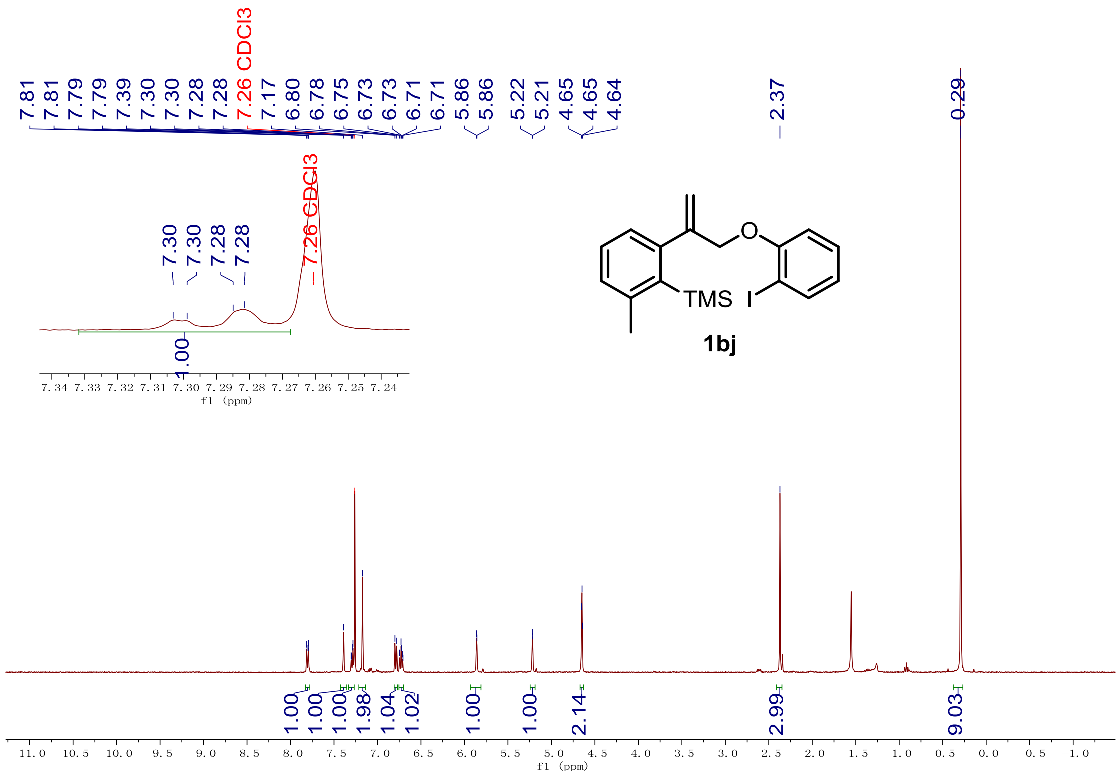

Supplementary Figure 65. **^1H NMR (400 MHz, $CDCl_3$)** spectra of **1bj**

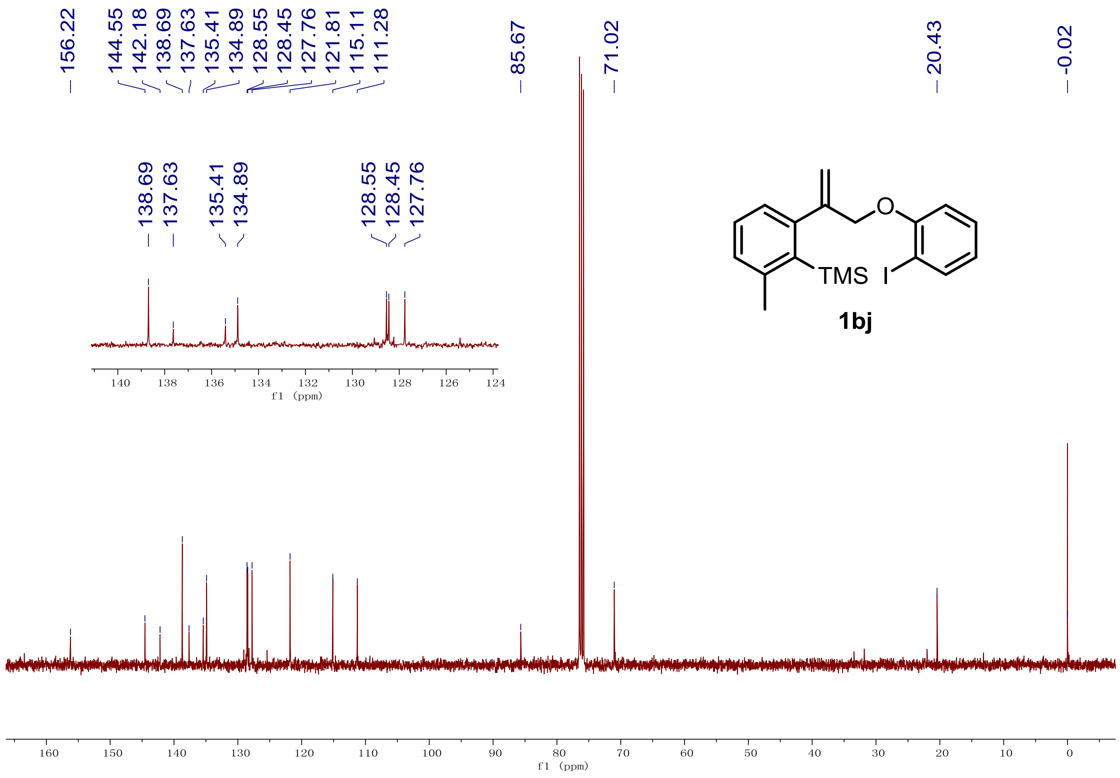

Supplementary Figure 66. **^{13}C NMR (101 MHz, $CDCl_3$)** spectra of **1bj**

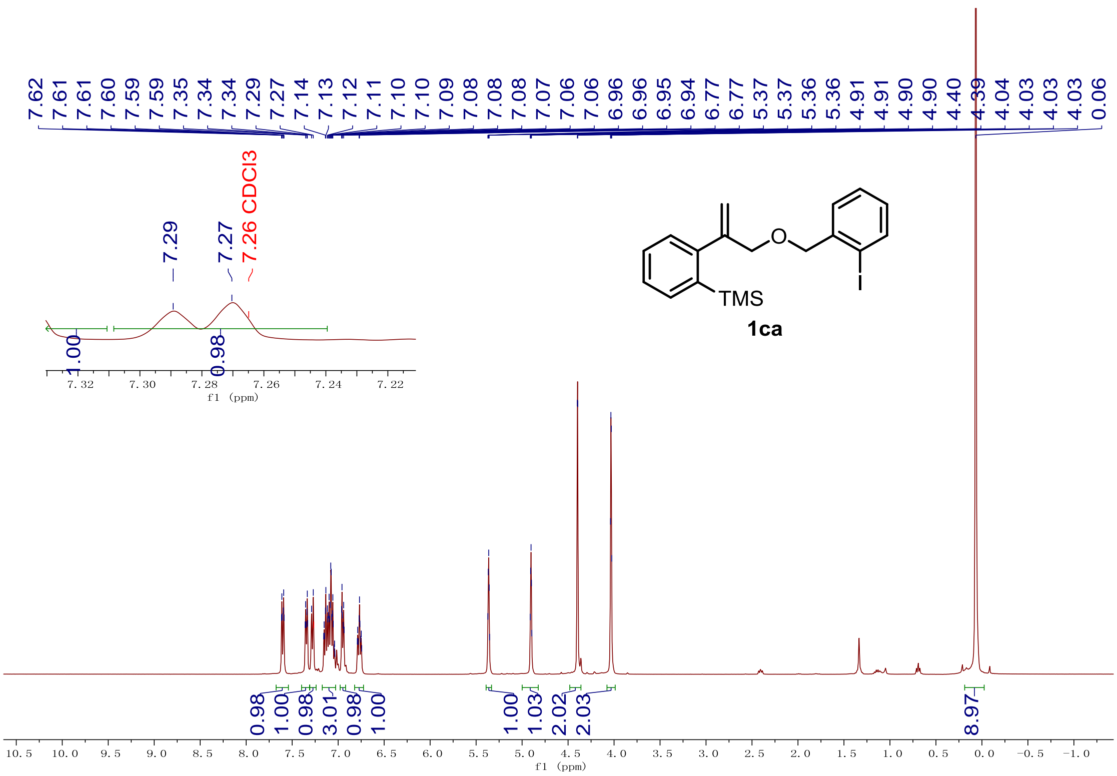

Supplementary Figure 67. ^{1}H NMR (400 MHz, $CDCl_3$) spectra of **1ca**

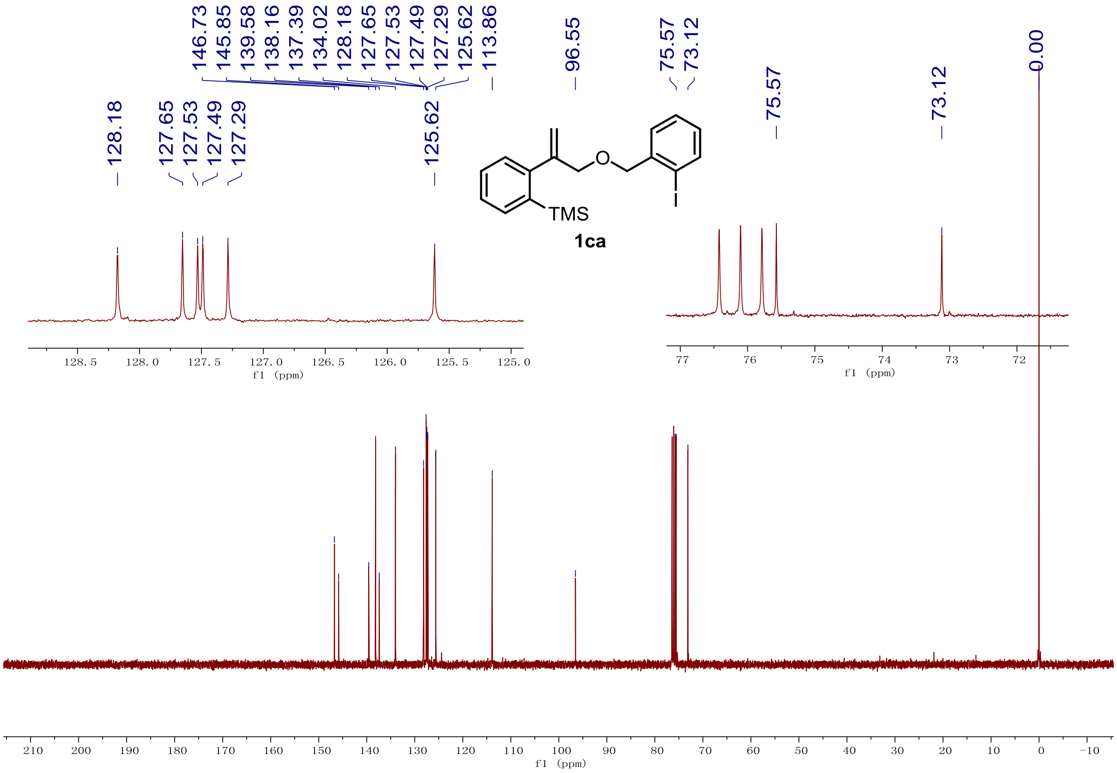

Supplementary Figure 68. ^{13}C NMR (101 MHz, $CDCl_3$) spectra of **1ca**

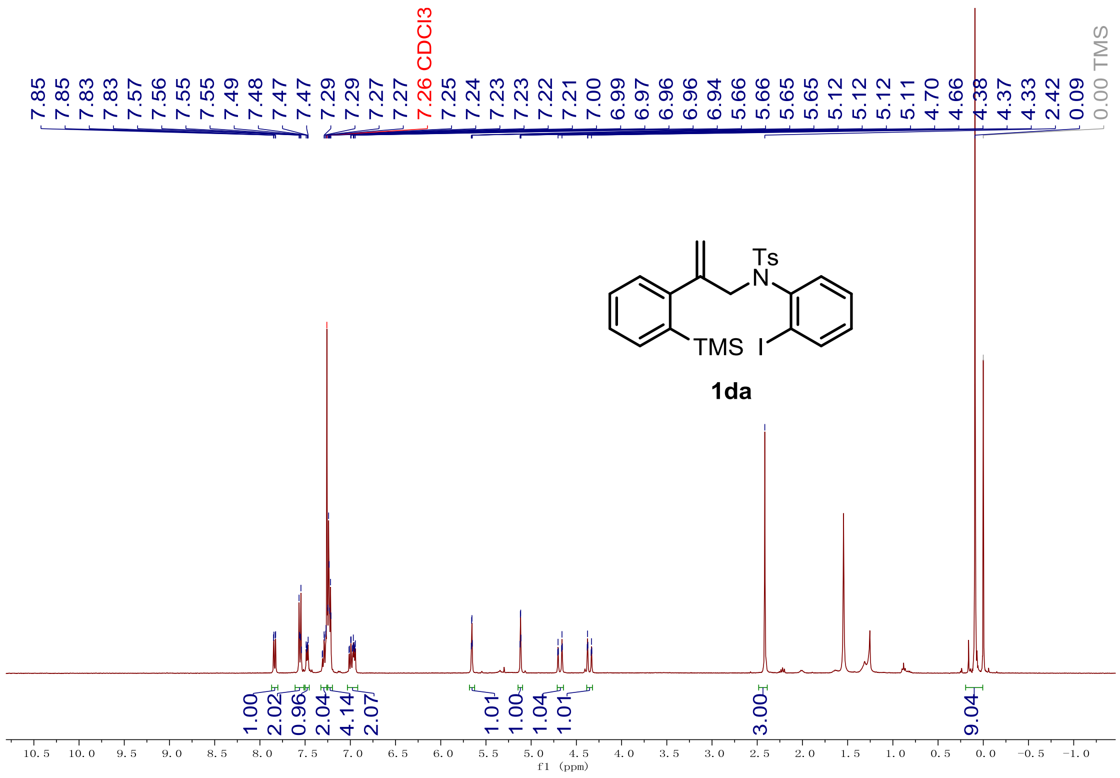

Supplementary Figure 69. ^{1}**H NMR (400 MHz, CDCl$_3$)** spectra of **1da**

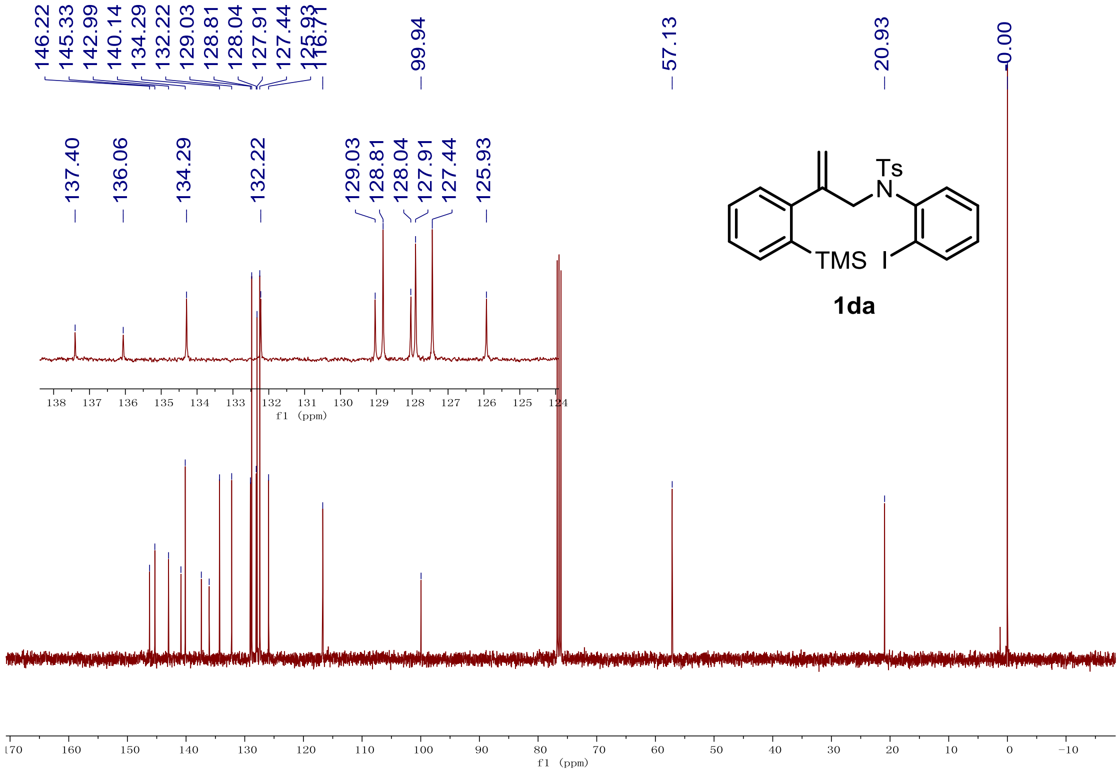

Supplementary Figure 70. ^{13}**C NMR (101 MHz, CDCl$_3$)** spectra of **1da**

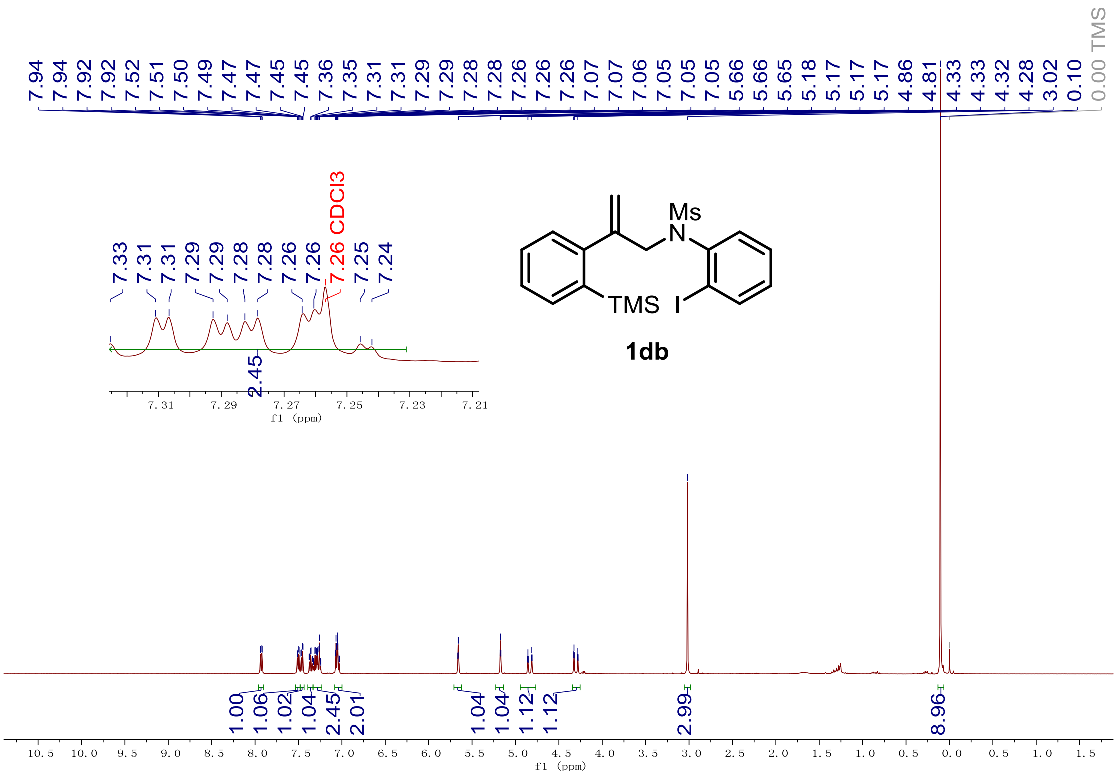

Supplementary Figure 71. ^{1}H NMR (400 MHz, CDCl$_3$) spectra of **1db**

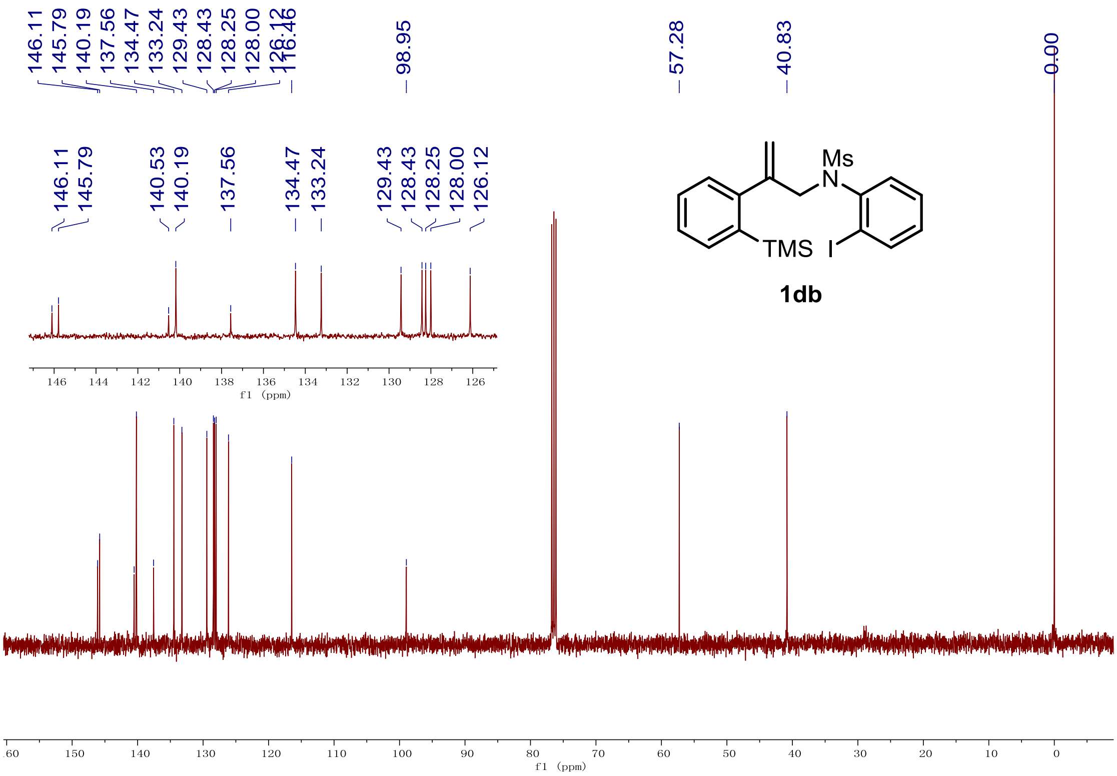

Supplementary Figure 72. ^{13}C NMR (101 MHz, CDCl$_3$) spectra of **1db**

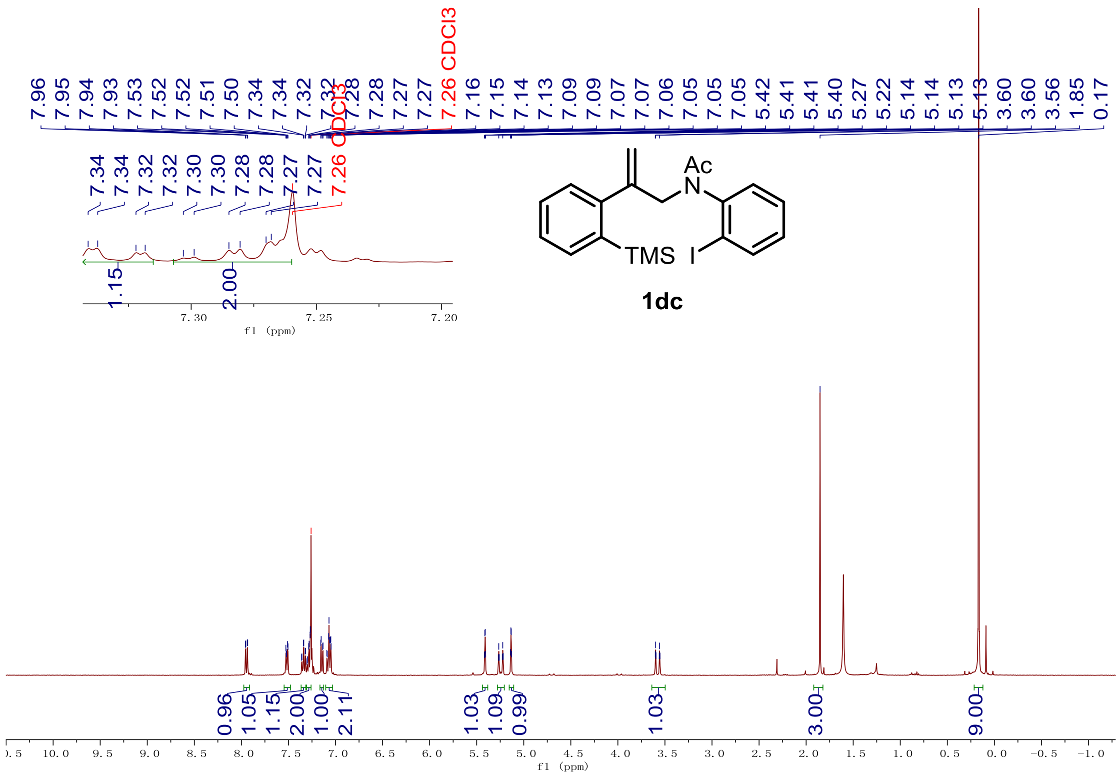

Supplementary Figure 73. ^{1}H NMR (400 MHz, $CDCl_3$) spectra of **1dc**

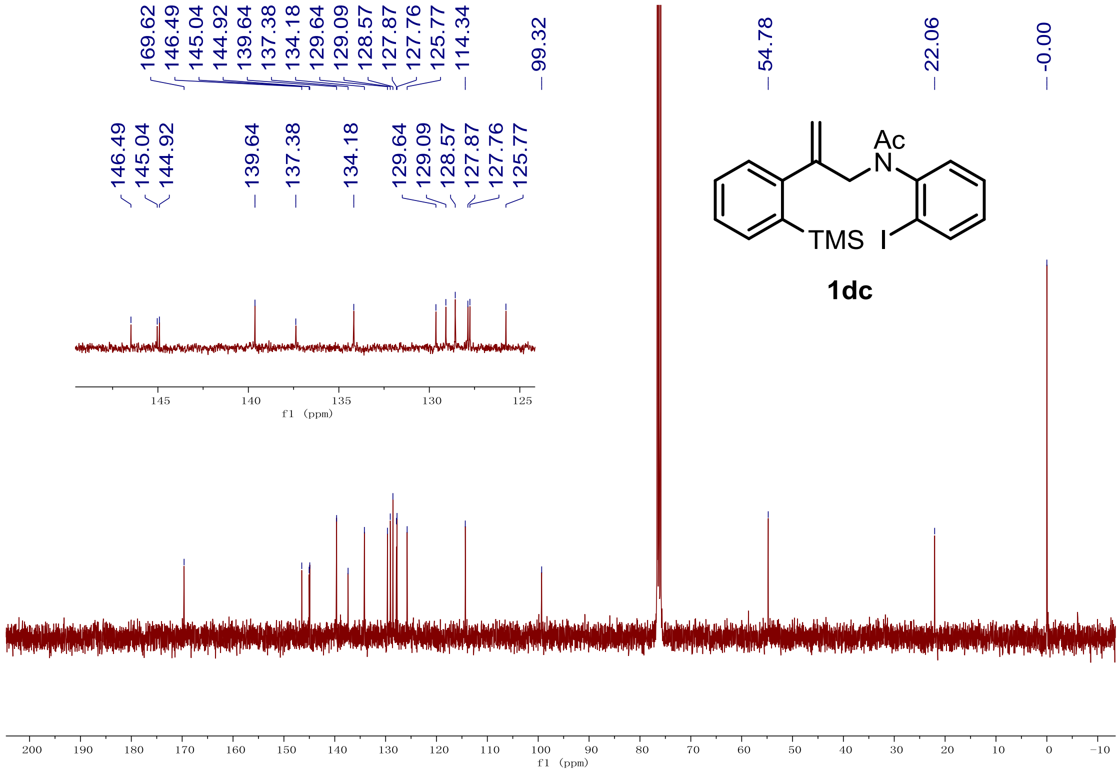

Supplementary Figure 74. ^{13}C NMR (101 MHz, $CDCl_3$) spectra of **1dc**

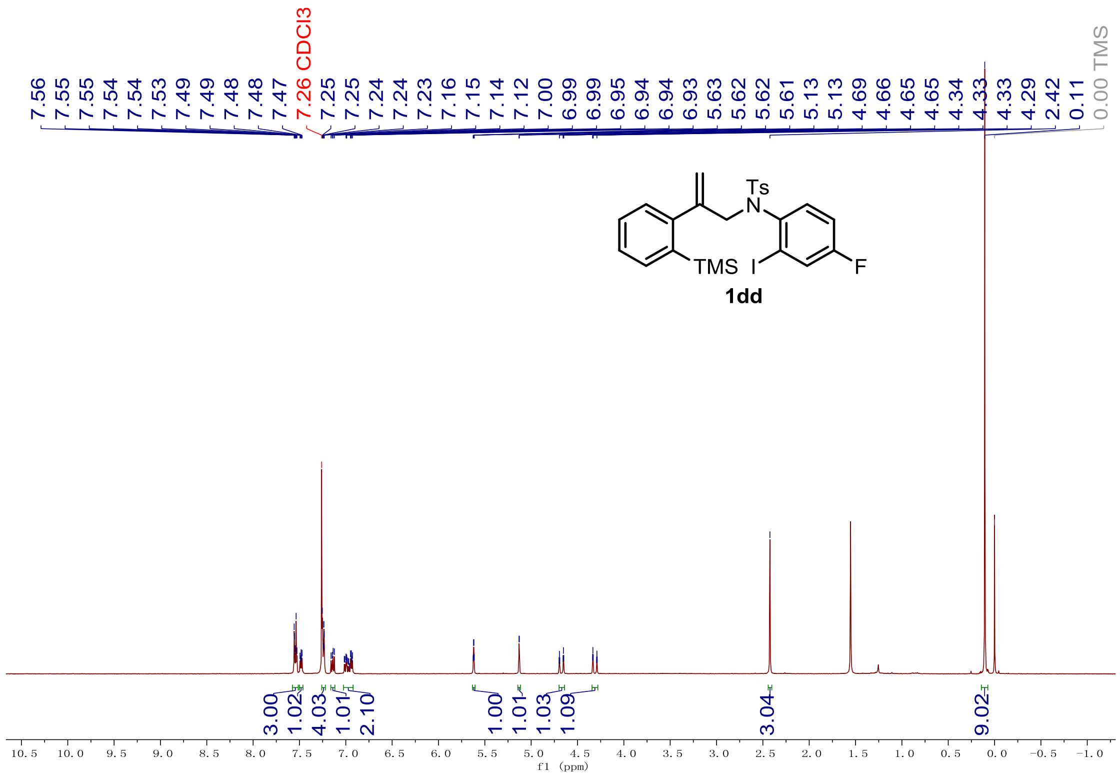

Supplementary Figure 75. ^{1}H NMR (400 MHz, CDCl$_3$) spectra of 1dd

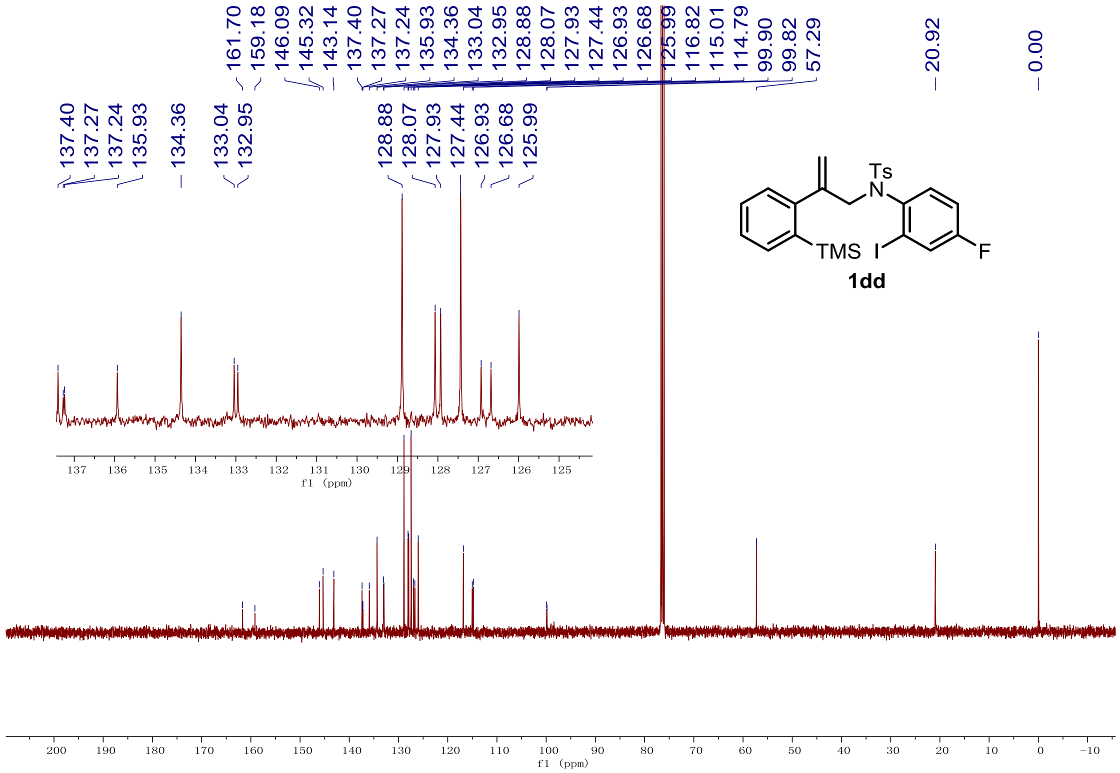

Supplementary Figure 76. ^{13}C NMR (101 MHz, CDCl$_3$) spectra of 1dd

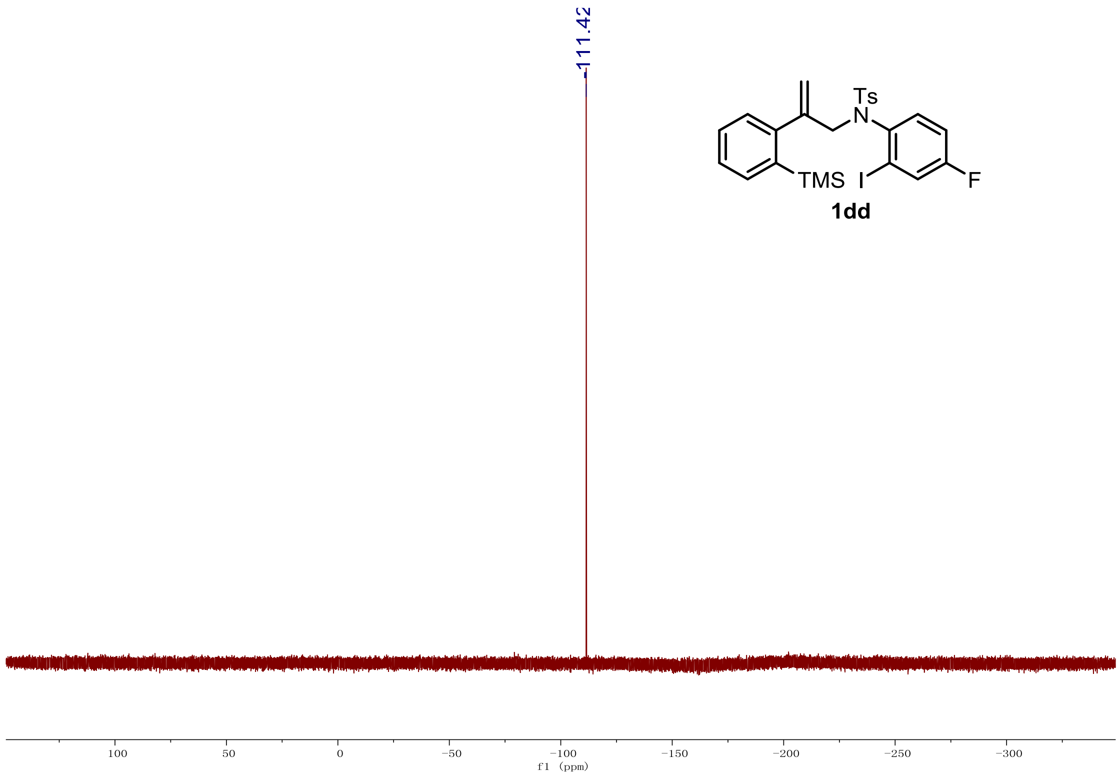

Supplementary Figure 77. ^{19}F NMR (376 MHz, $CDCl_3$) spectra of **1dd**

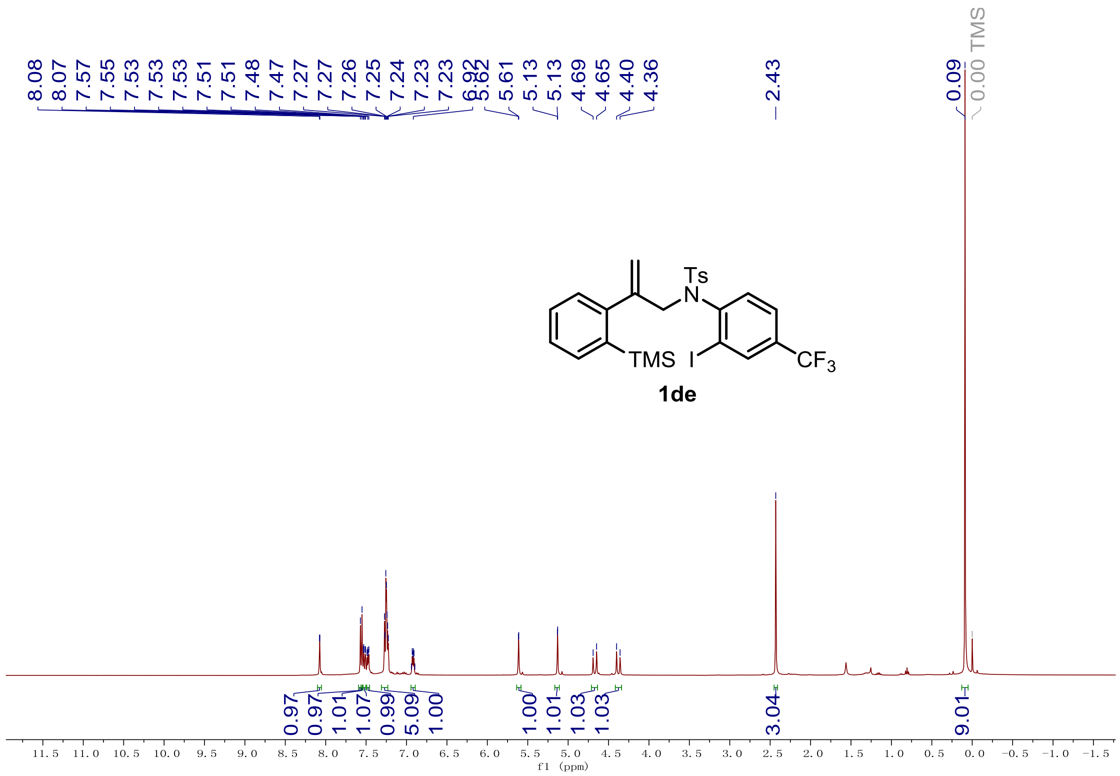

Supplementary Figure 78. ^{1}**H NMR (400 MHz, CDCl$_3$)** spectra of **1de**

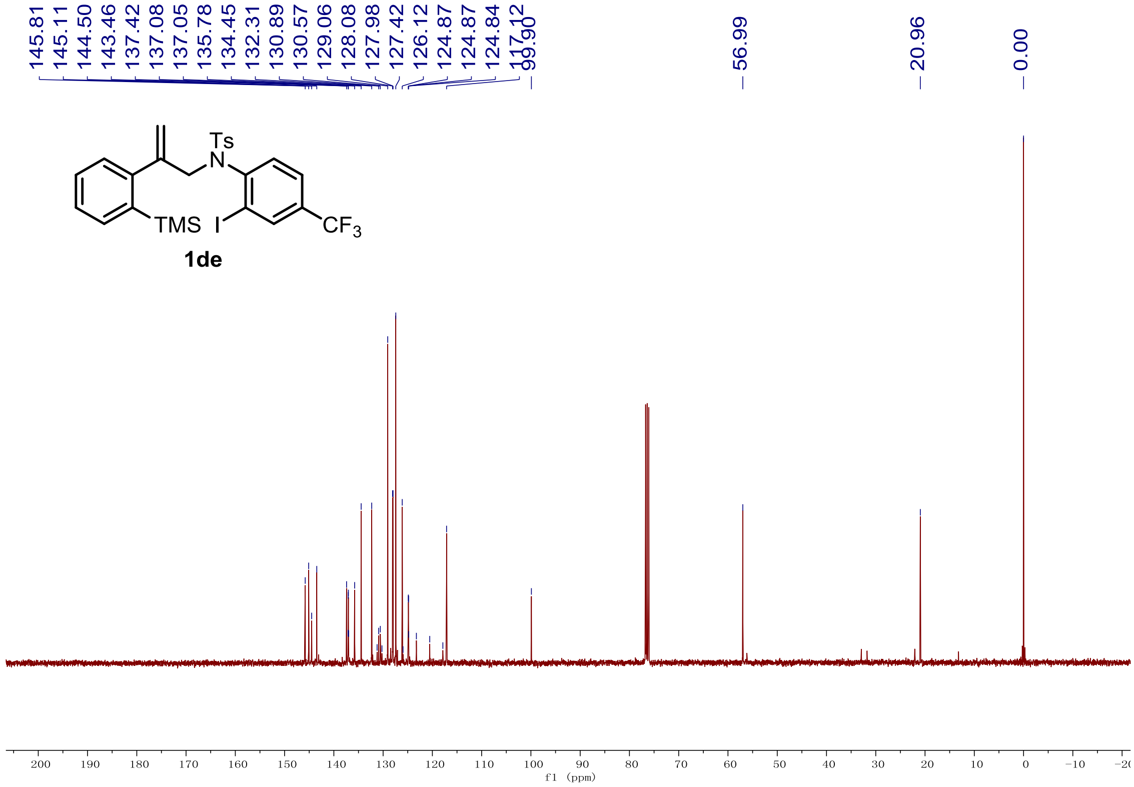

Supplementary Figure 79. ^{13}**C NMR (101 MHz, CDCl$_3$)** spectra of **1de**

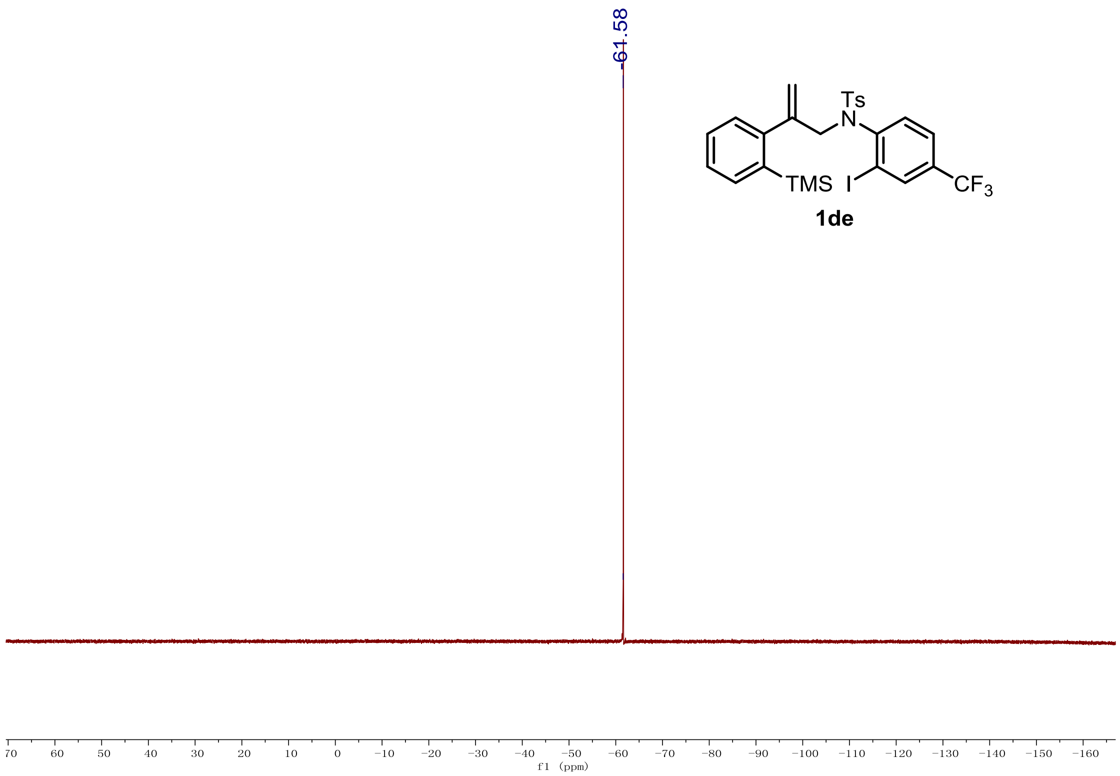

Supplementary Figure 80. ^{19}F NMR (376 MHz, $CDCl_3$) spectra of **1de**

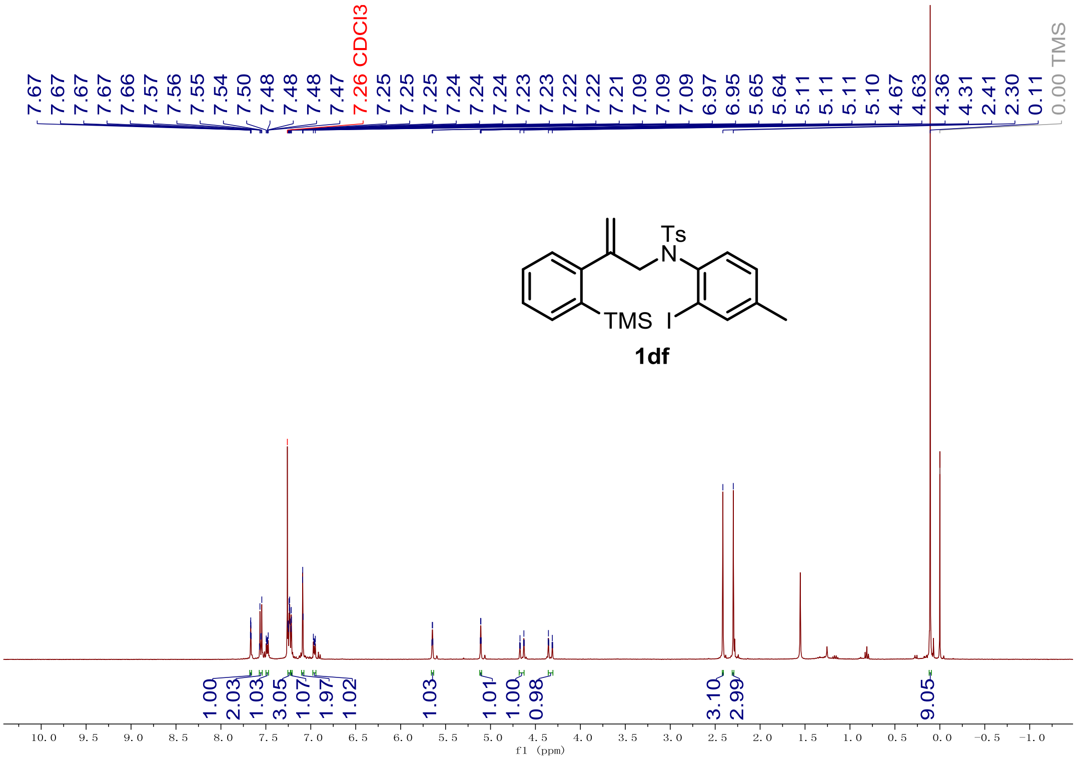

Supplementary Figure 81. **^{1}H NMR (400 MHz, $CDCl_3$)** spectra of **1df**

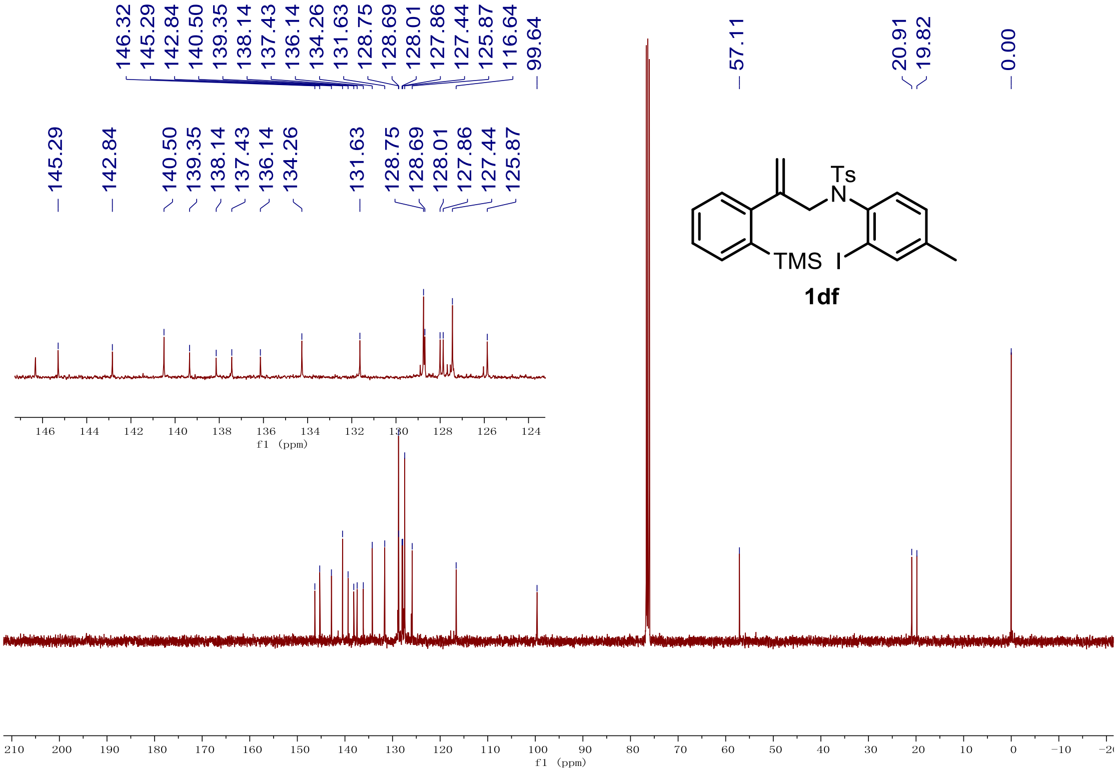

Supplementary Figure 82. **^{13}C NMR (101 MHz, $CDCl_3$)** spectra of **1df**

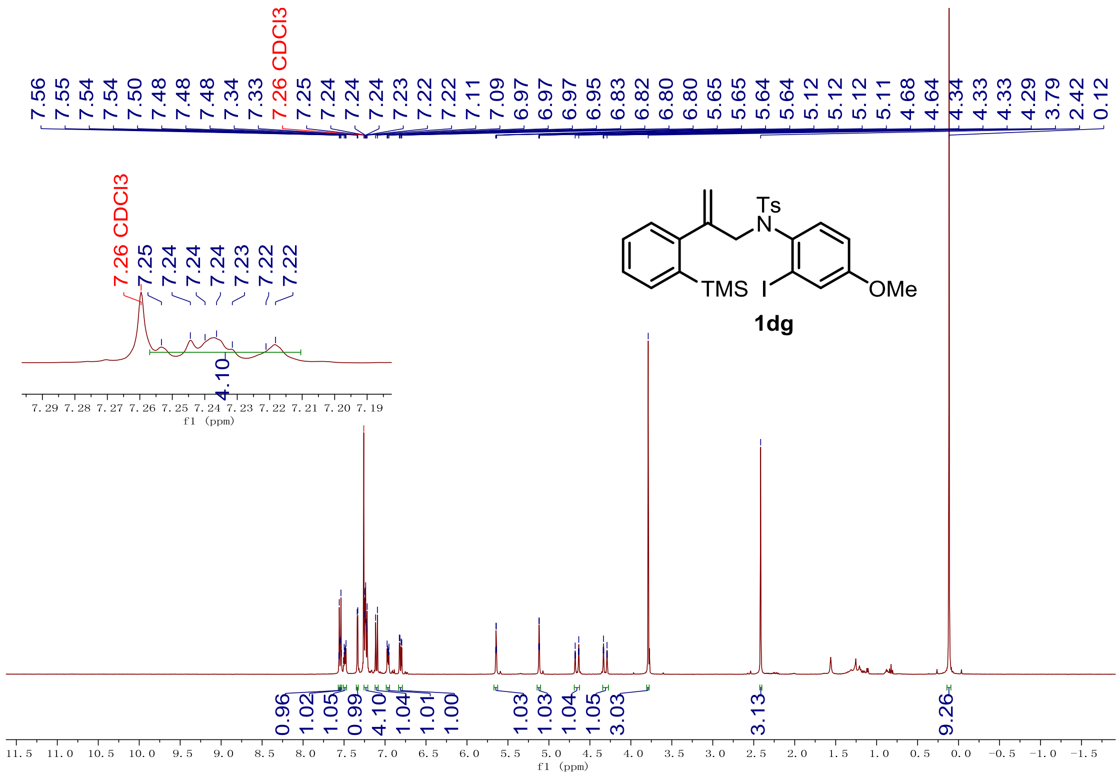

Supplementary Figure 83. ^{1}H NMR (400 MHz, CDCl$_3$) spectra of **1dg**

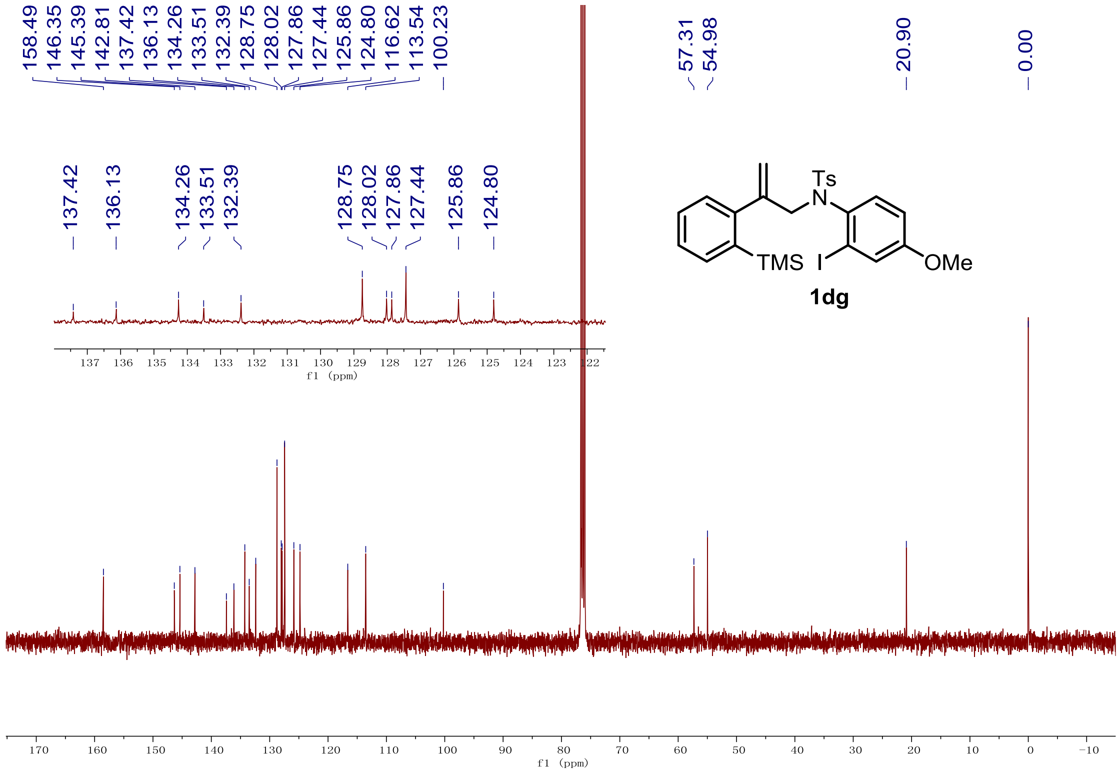

Supplementary Figure 84. ^{13}C NMR (101 MHz, CDCl$_3$) spectra of **1dg**

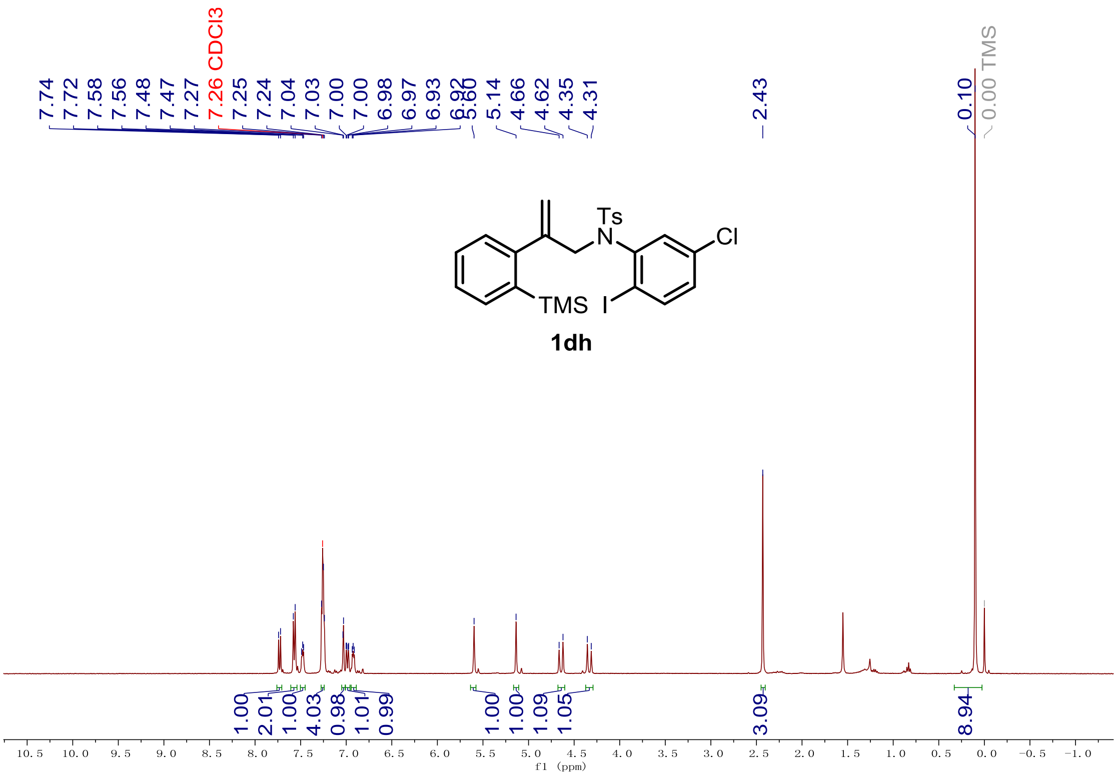

Supplementary Figure 85. ^{1}**H NMR (400 MHz, CDCl$_3$)** spectra of **1dh**

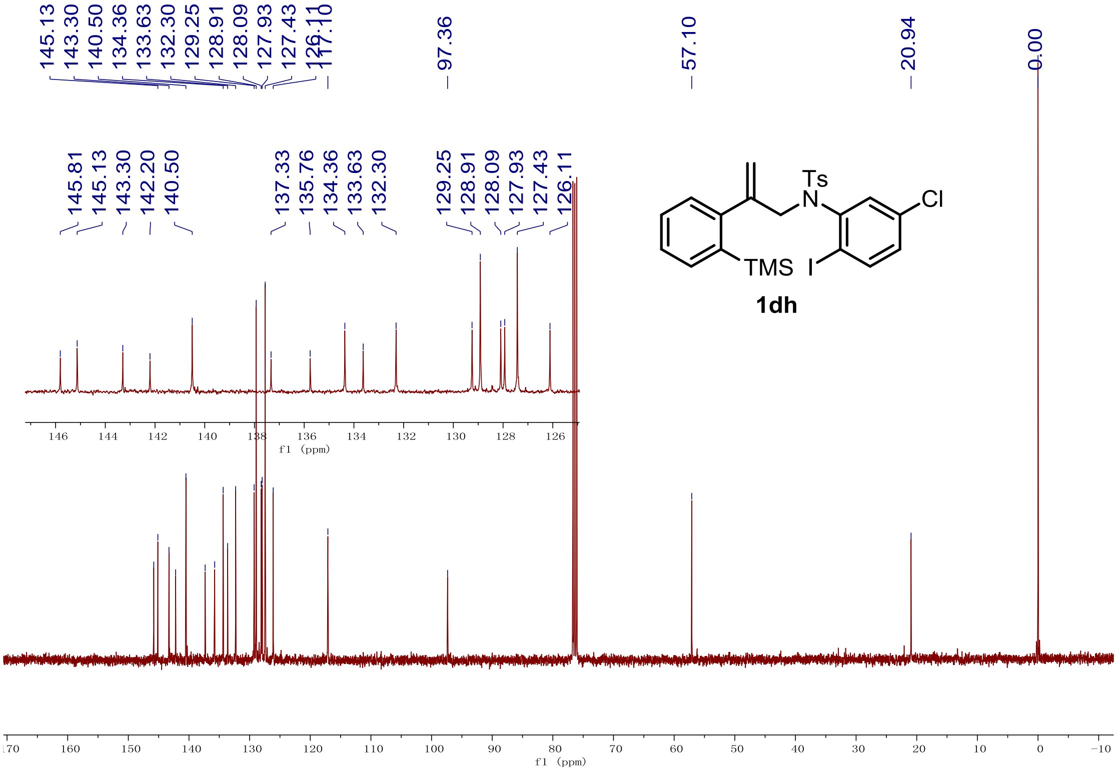

Supplementary Figure 86. ^{13}**C NMR (101 MHz, CDCl$_3$)** spectra of **1dh**

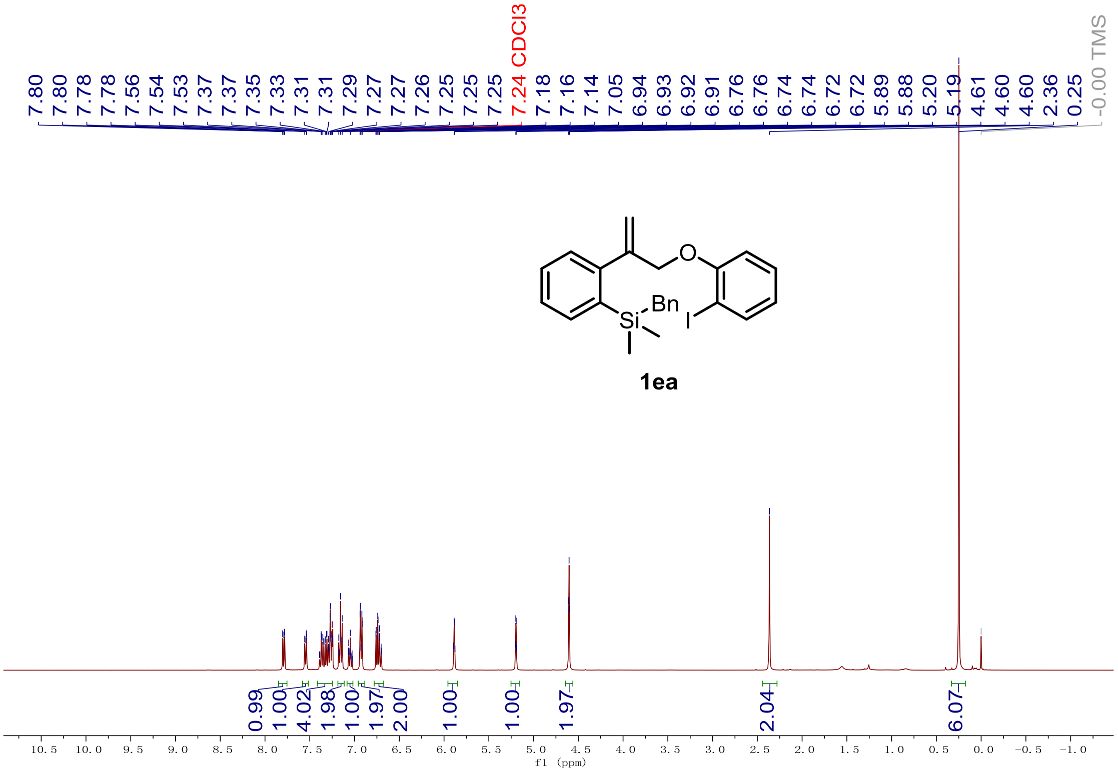

Supplementary Figure 87. ^{1}**H NMR (400 MHz, CDCl**$_{3}$**)** spectra of **1ea**

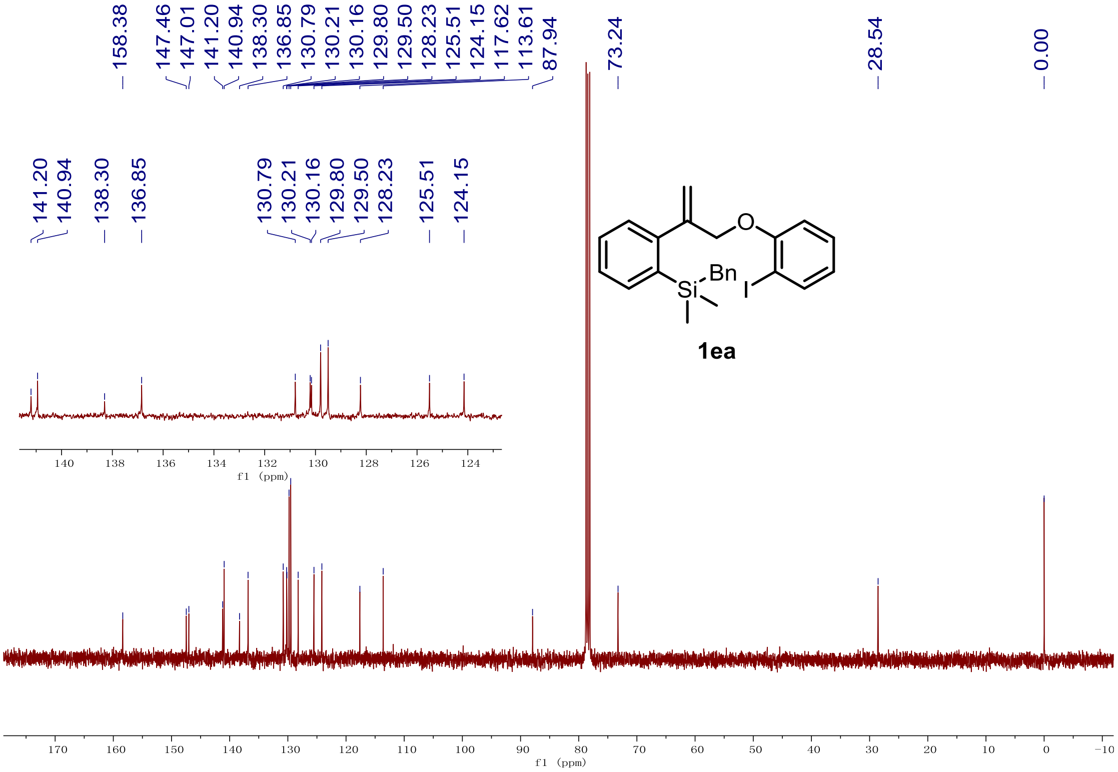

Supplementary Figure 88. ^{13}**C NMR (101 MHz, CDCl**$_{3}$**)** spectra of **1ea**

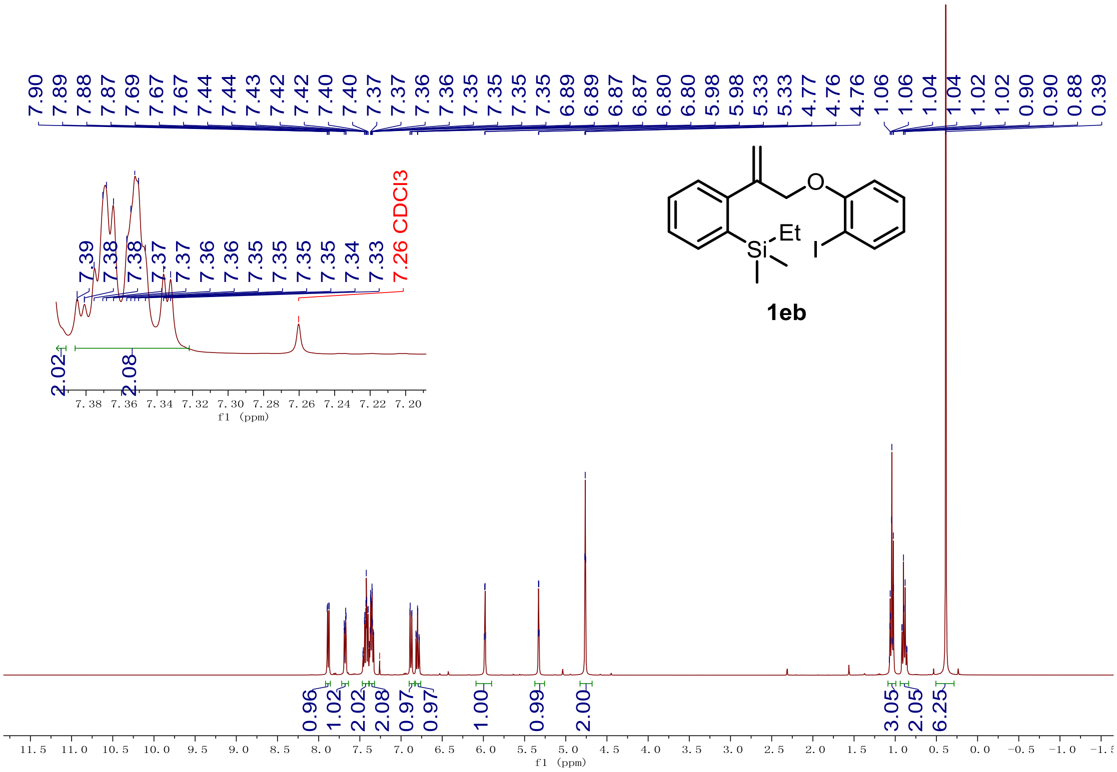

Supplementary Figure 89. **^{1}H NMR (400 MHz, $CDCl_3$)** spectra of **1eb**

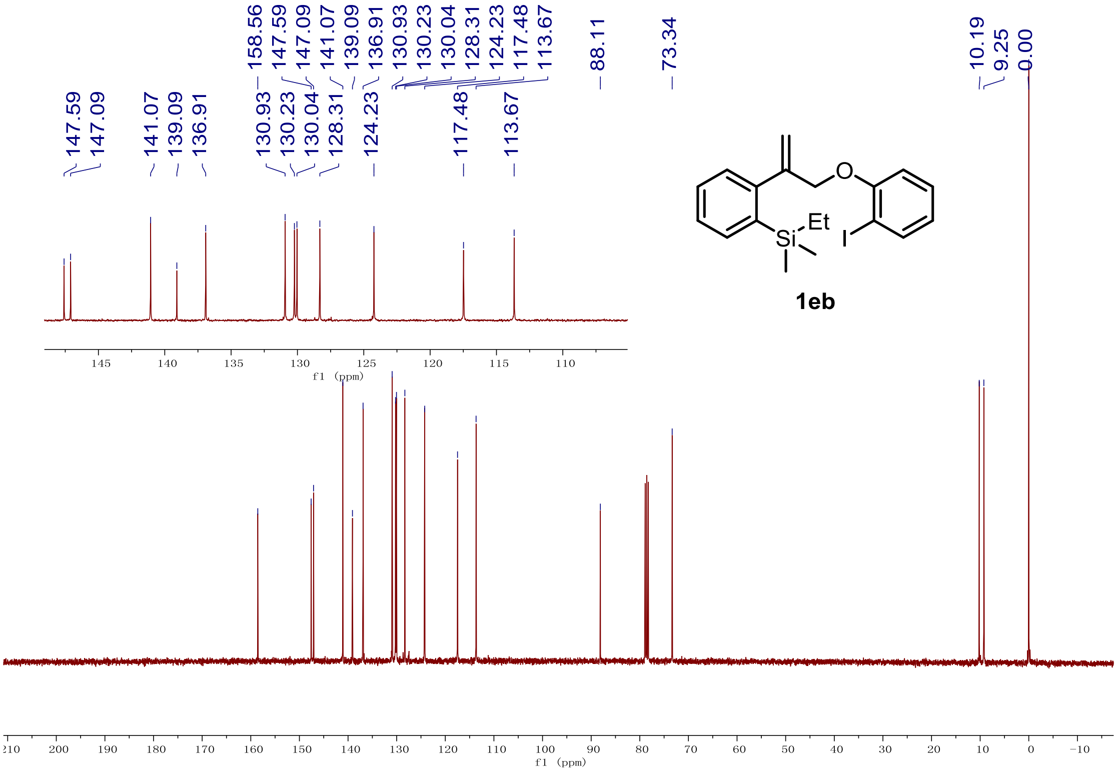

Supplementary Figure 90. **^{13}C NMR (101 MHz, $CDCl_3$)** spectra of **1eb**

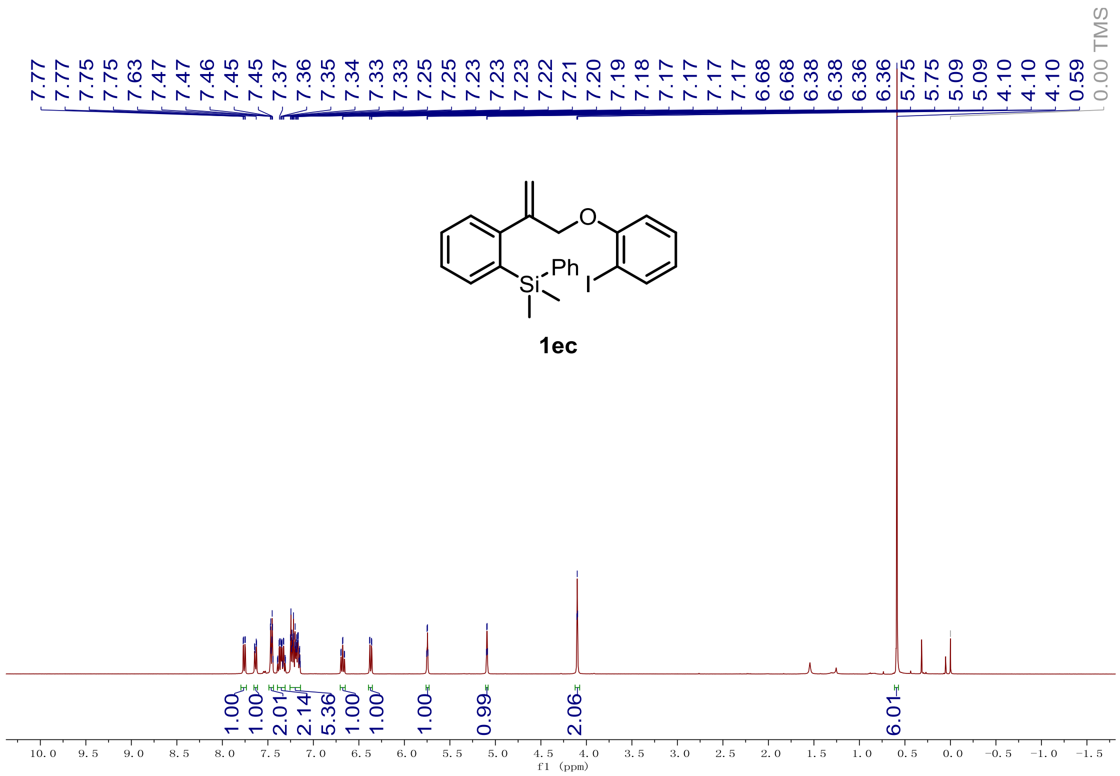

Supplementary Figure 91. ^{1}**H NMR (400 MHz, CDCl$_3$)** spectra of **1ec**

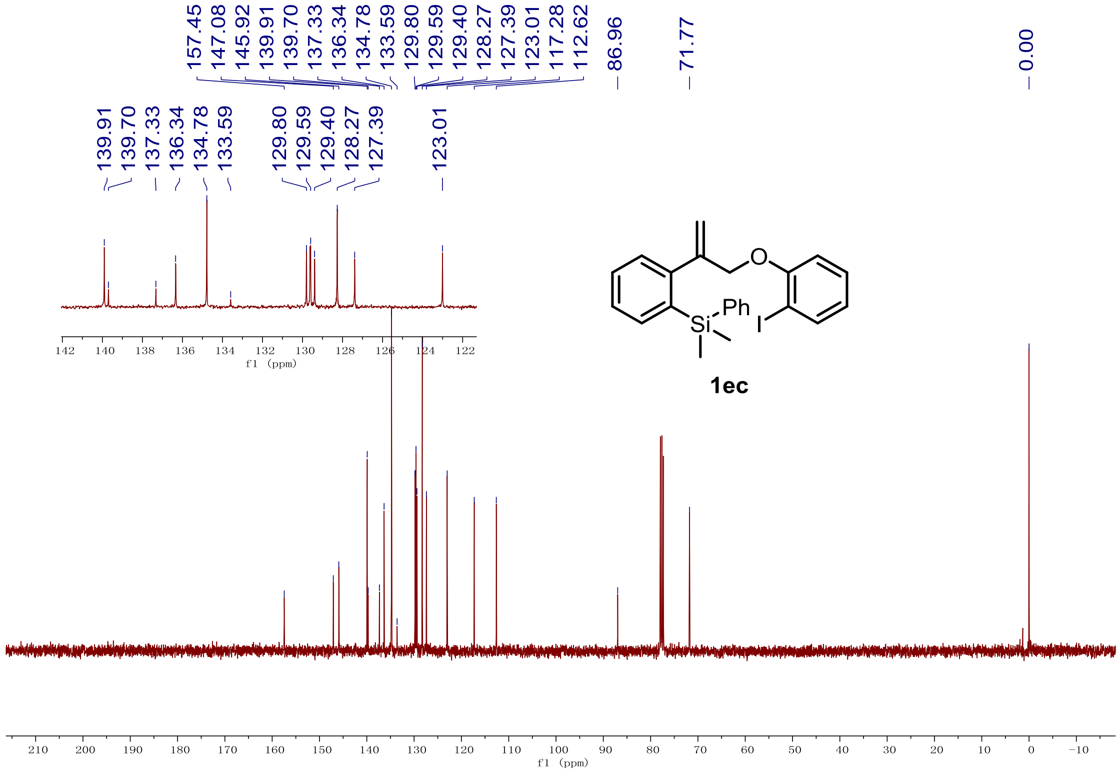

Supplementary Figure 92. ^{13}**C NMR (101 MHz, CDCl$_3$)** spectra of **1ec**

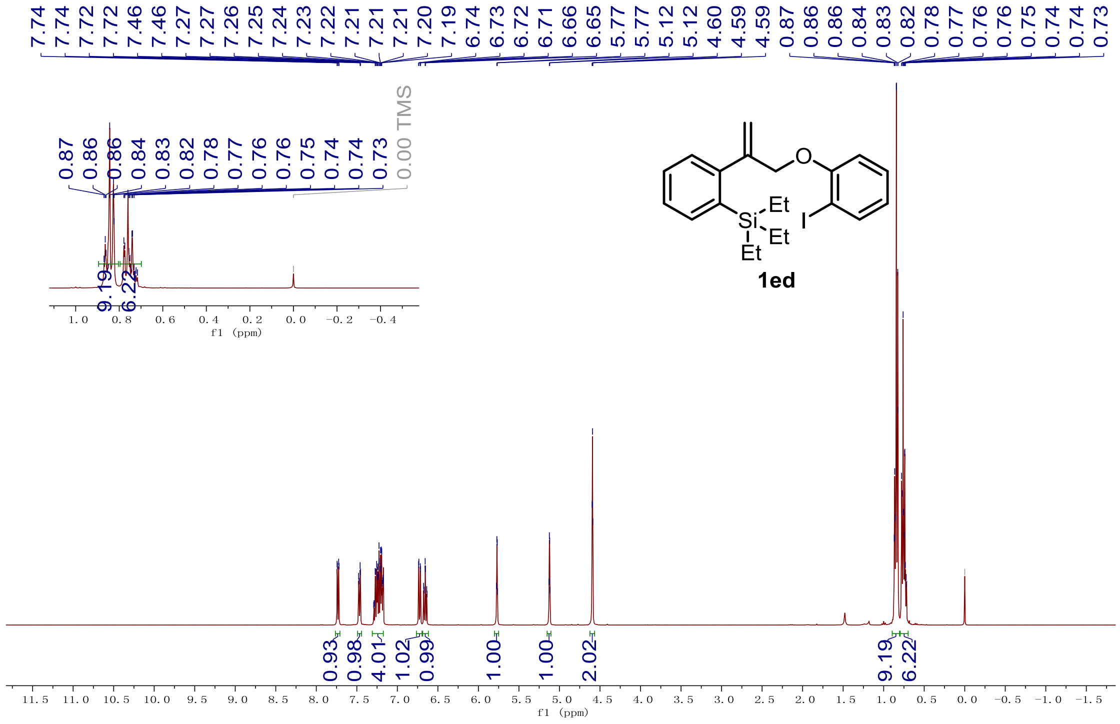

Supplementary Figure 93. ^{1}**H NMR (400 MHz, CDCl$_3$)** spectra of **1ed**

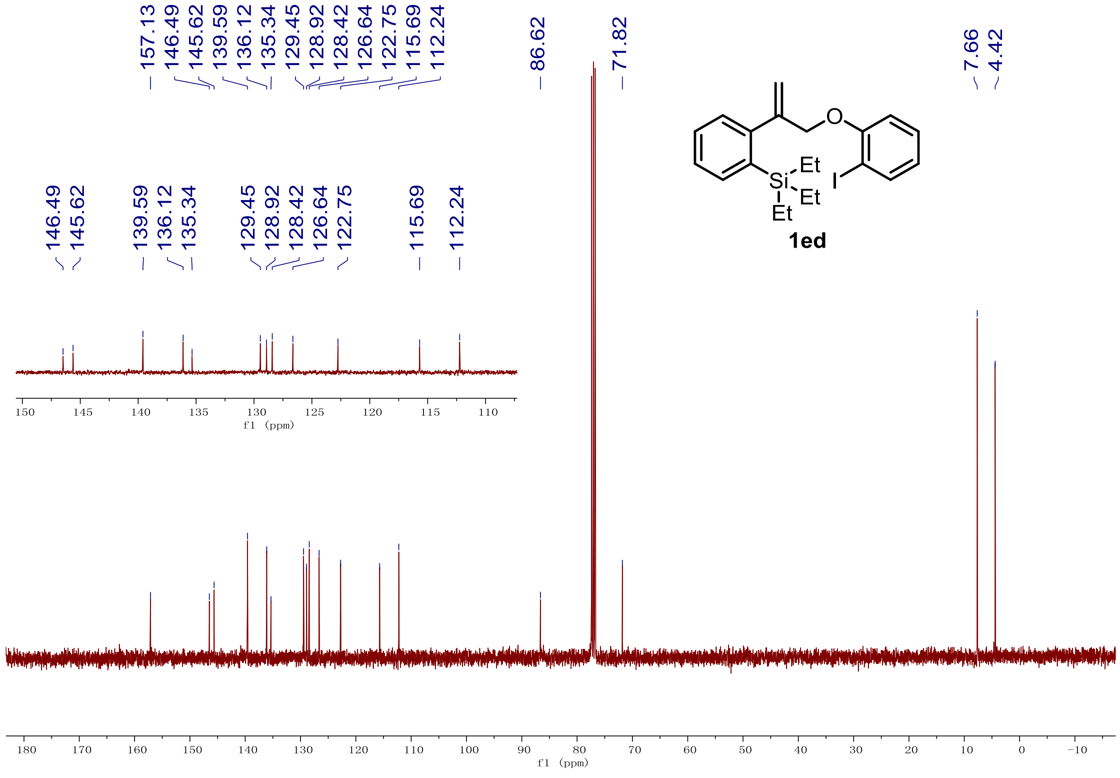

Supplementary Figure 94. ^{13}**C NMR (101 MHz, CDCl$_3$)** spectra of **1ed**

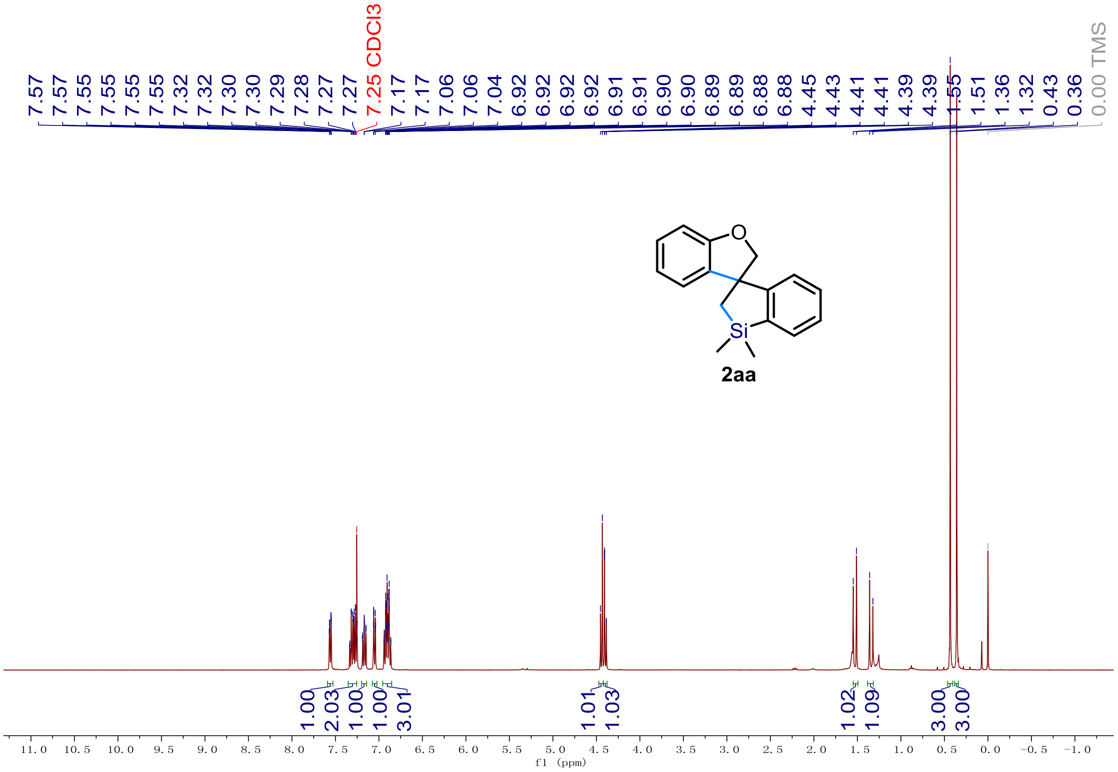

Supplementary Figure 95. ^{1}**H NMR (400 MHz, CDCl$_3$)** spectra of **2aa**

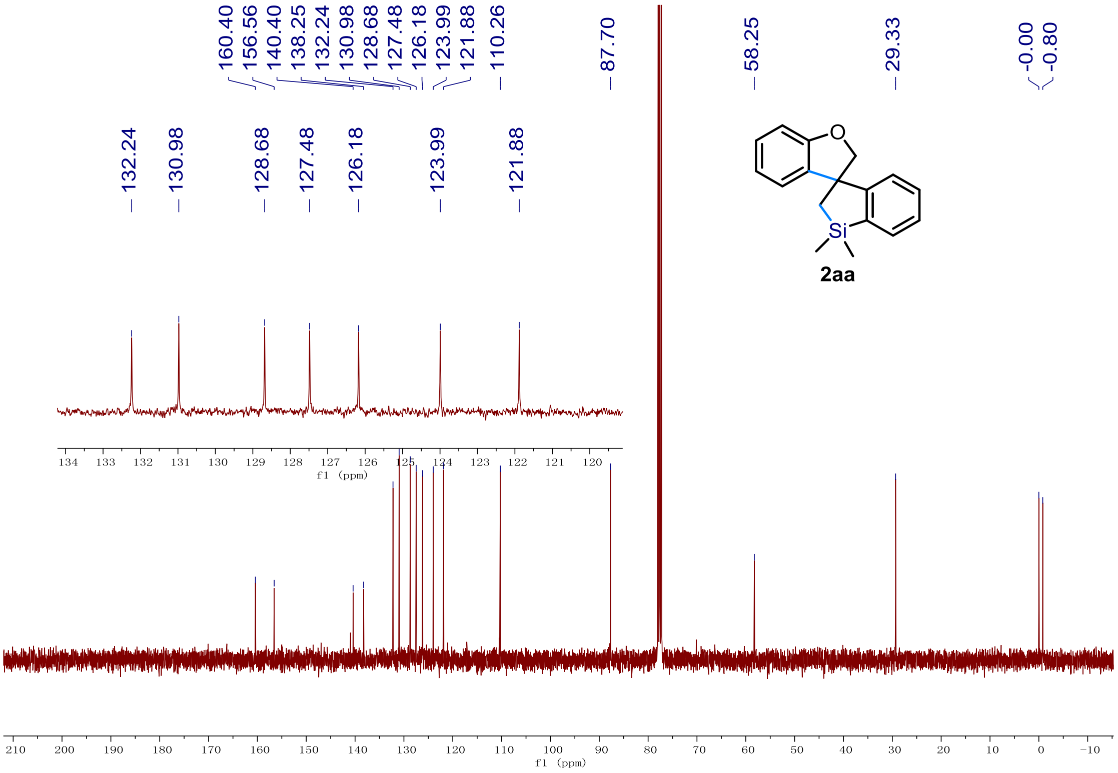

Supplementary Figure 96. ^{13}**C NMR (101 MHz, CDCl$_3$)** spectra of **2aa**

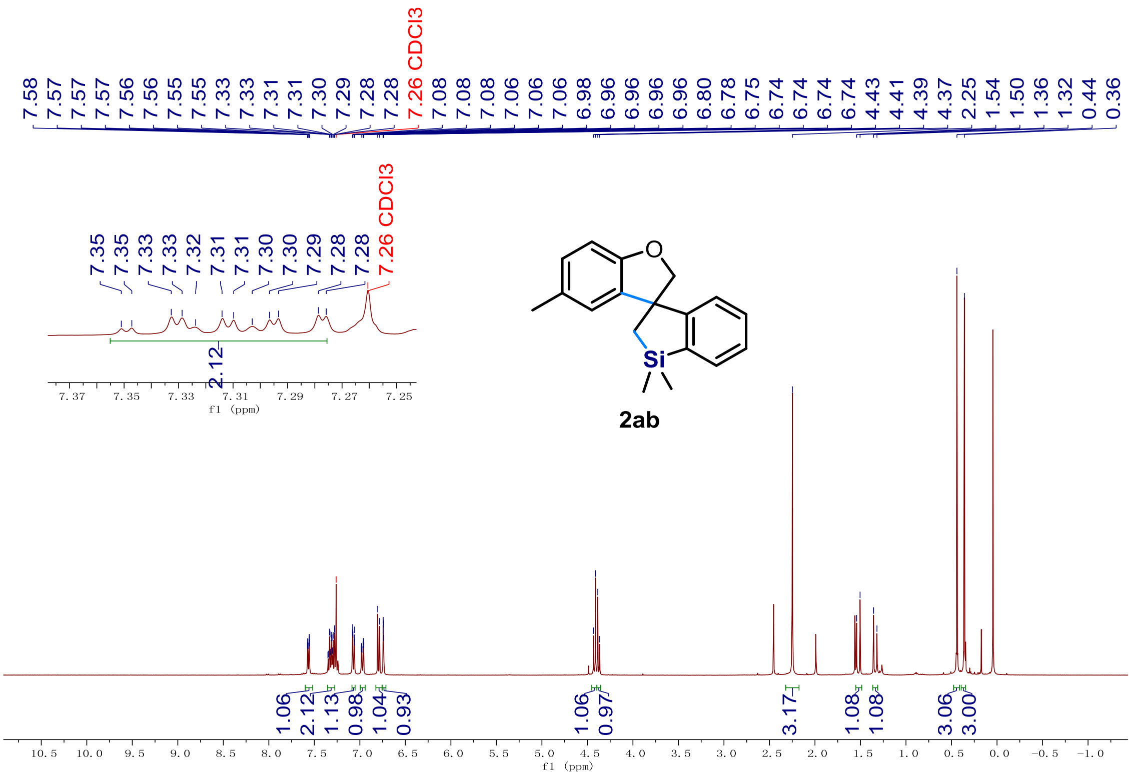

Supplementary Figure 97. **^{1}H NMR (400 MHz, $CDCl_3$)** spectra of **2ab**

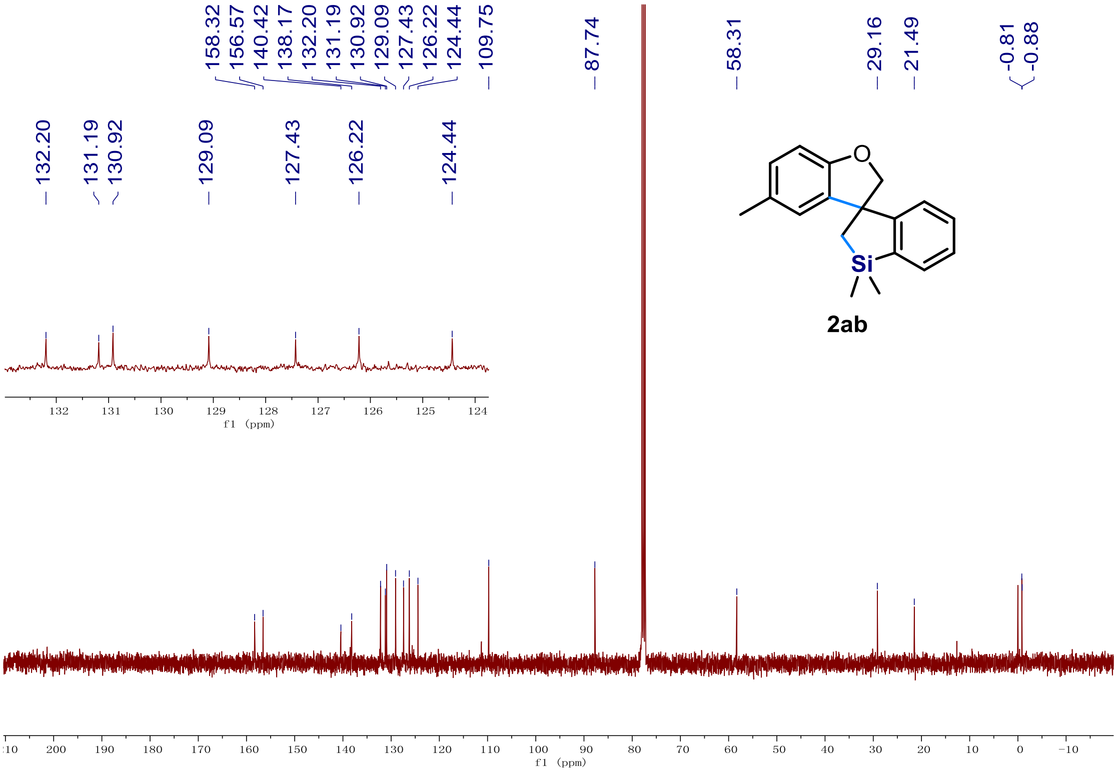

Supplementary Figure 98. **^{13}C NMR (101 MHz, $CDCl_3$)** spectra of **2ab**

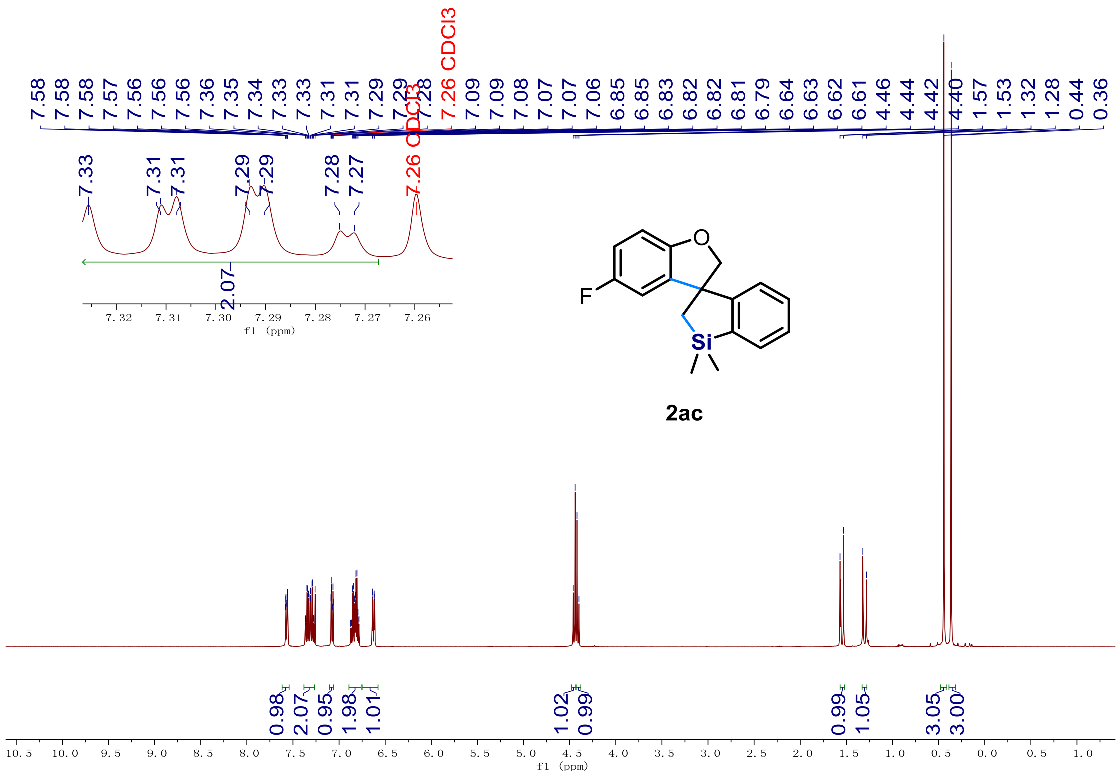

Supplementary Figure 99. ^{1}**H NMR (400 MHz, CDCl$_3$)** spectra of **2ac**

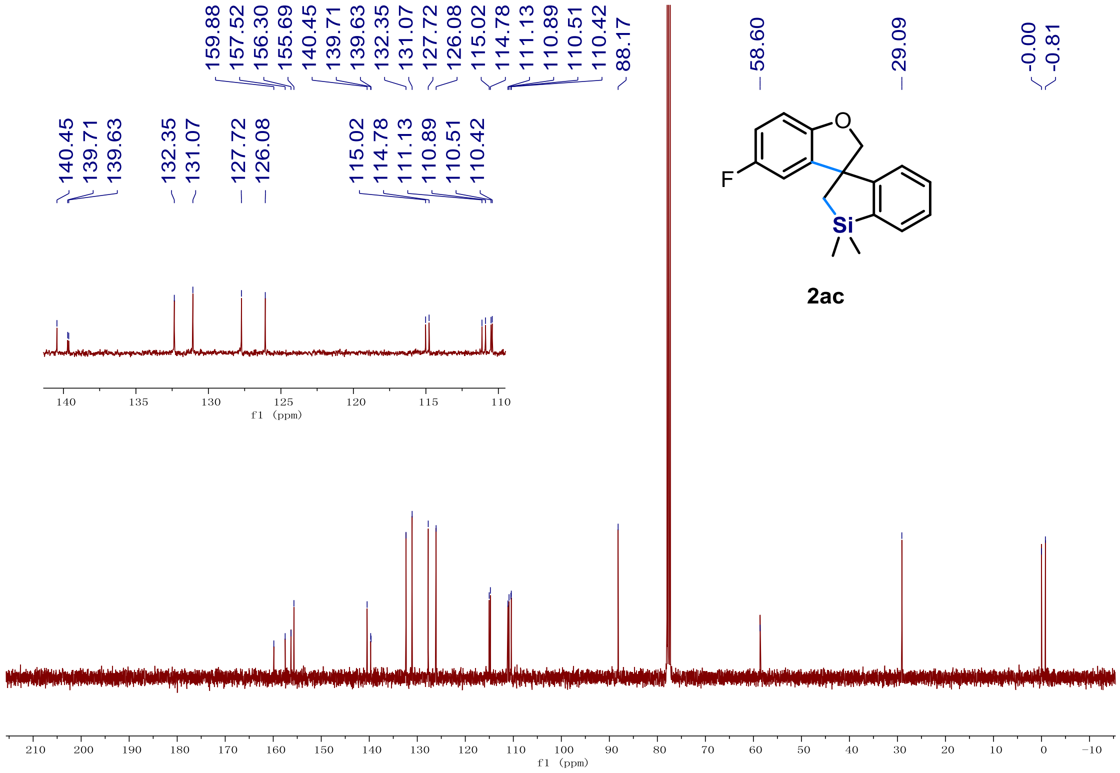

Supplementary Figure 100. ^{13}**C NMR (101 MHz, CDCl$_3$)** spectra of **2ac**

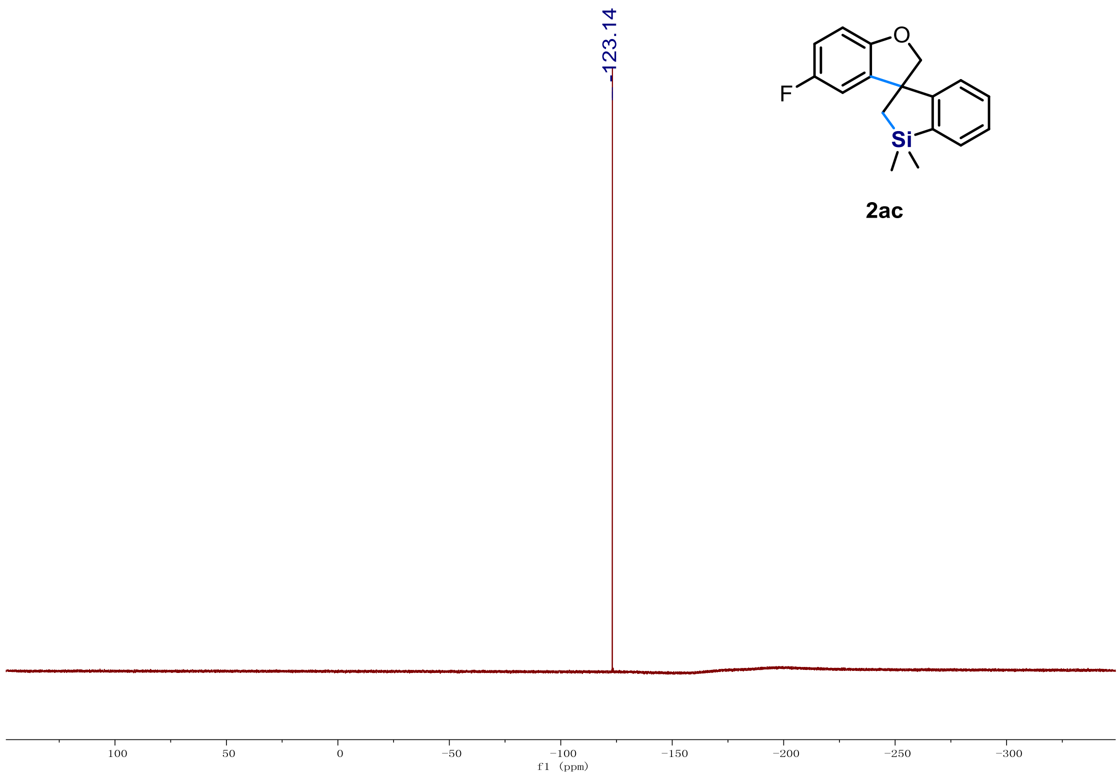

Supplementary Figure 101. ^{19}F NMR (376 MHz, $CDCl_3$) spectra of **2ac**

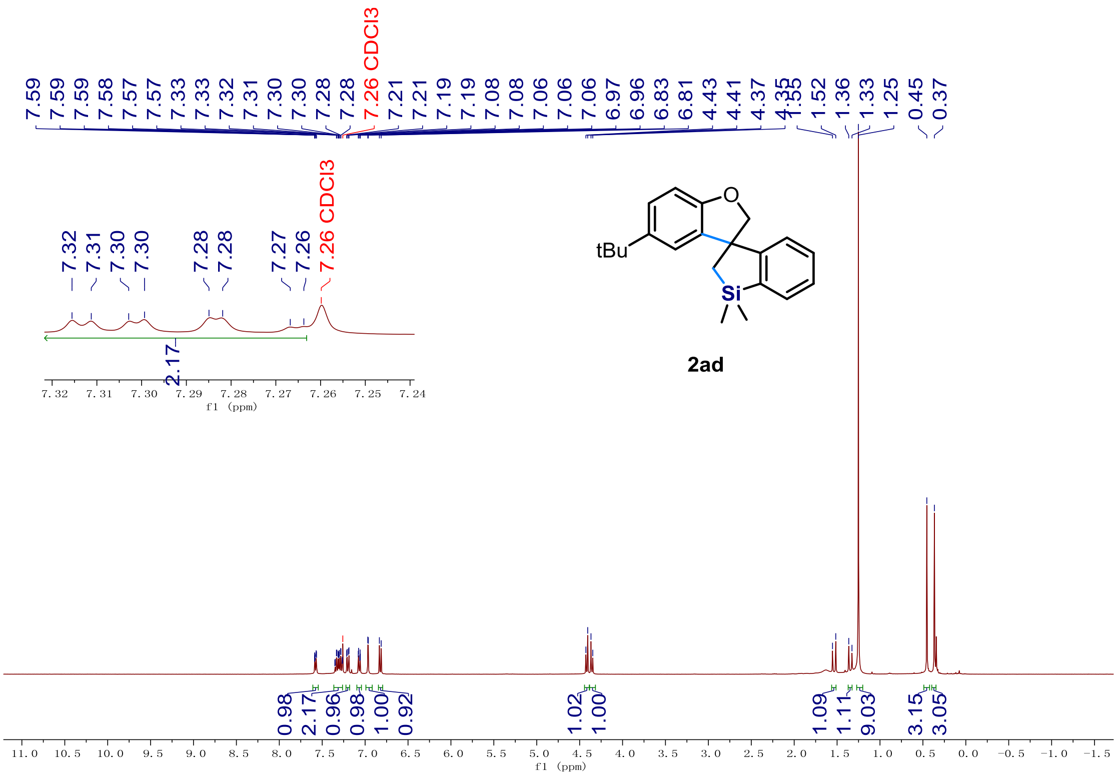

Supplementary Figure 102. ^{1}**H NMR (400 MHz, CDCl$_3$)** spectra of **2ad**

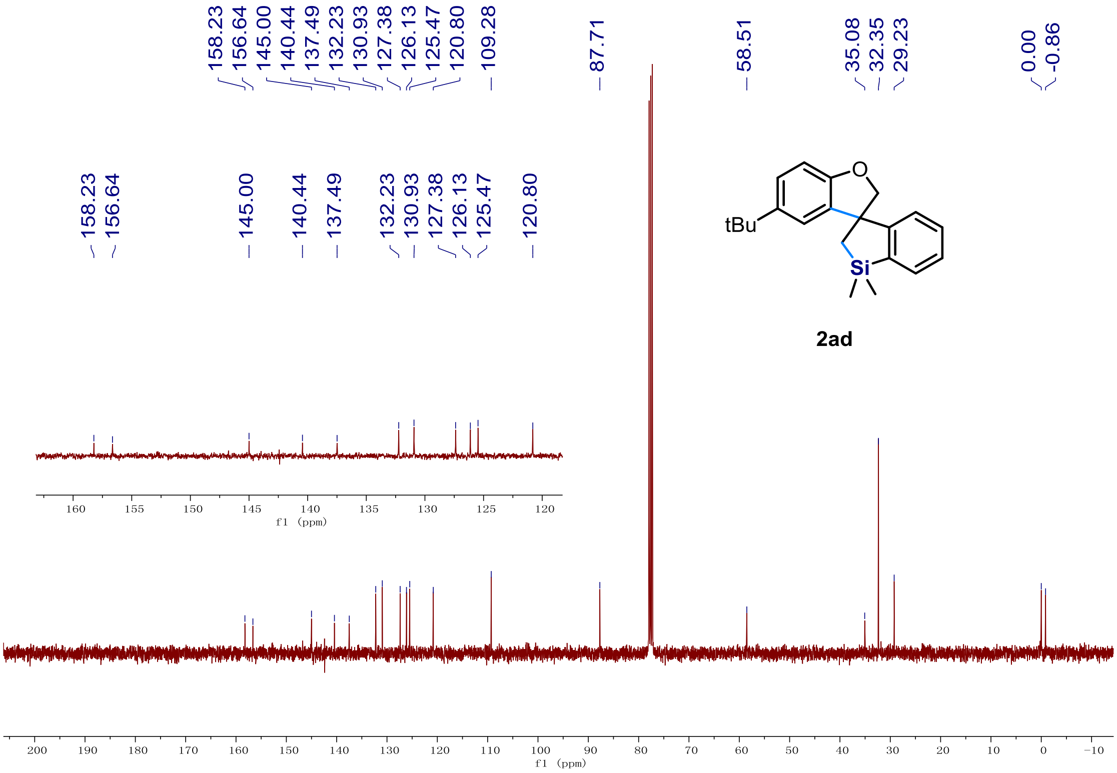

Supplementary Figure 103. ^{13}**C NMR (101 MHz, CDCl$_3$)** spectra of **2ad**

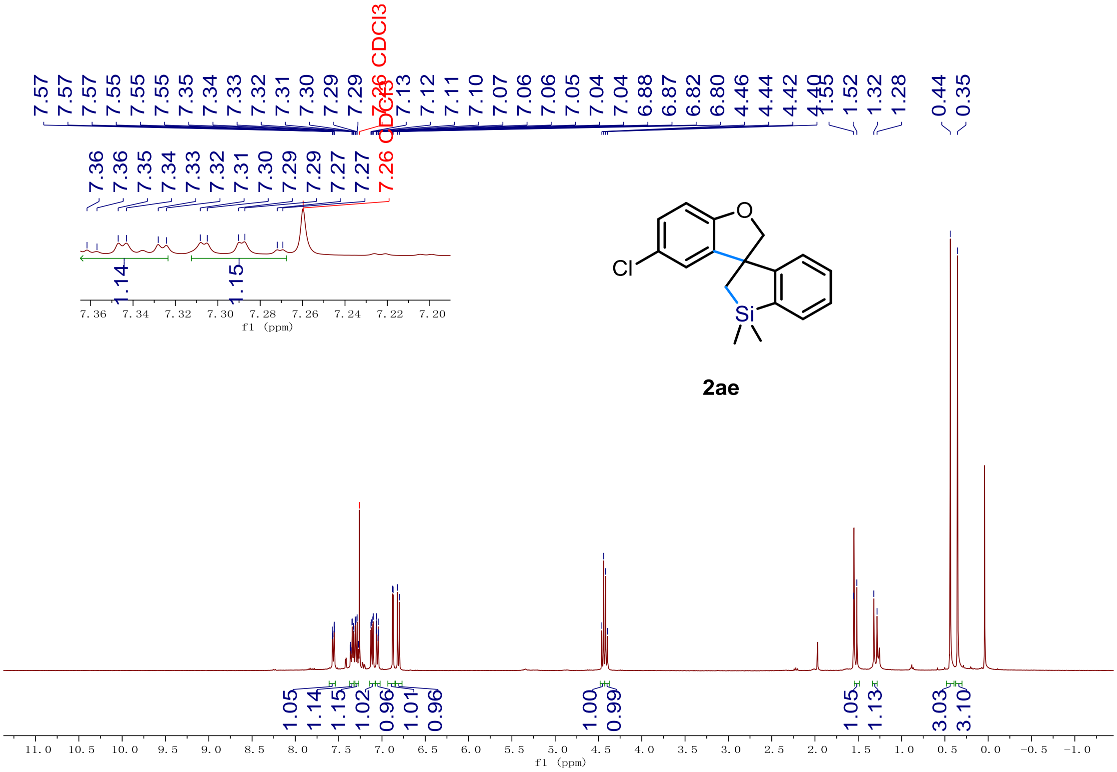

Supplementary Figure 104. ^{1}**H NMR (400 MHz, CDCl$_3$)** spectra of **2ae**

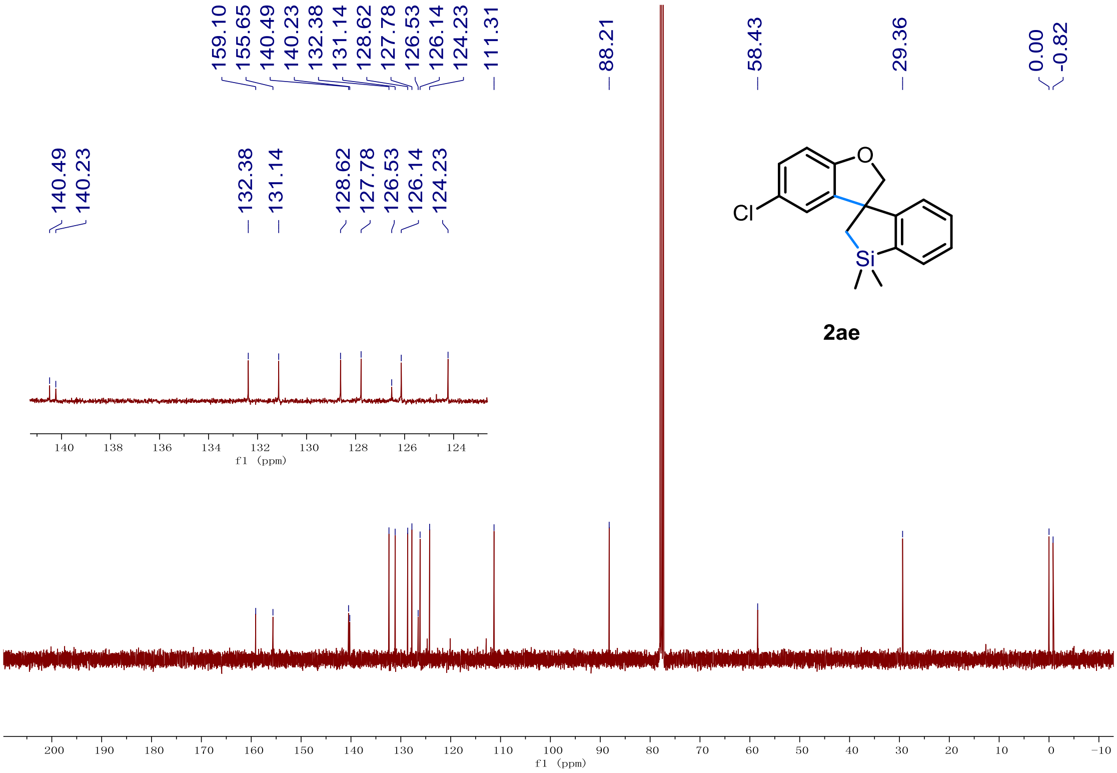

Supplementary Figure 105. ^{13}**C NMR (101 MHz, CDCl$_3$)** spectra of **2ae**

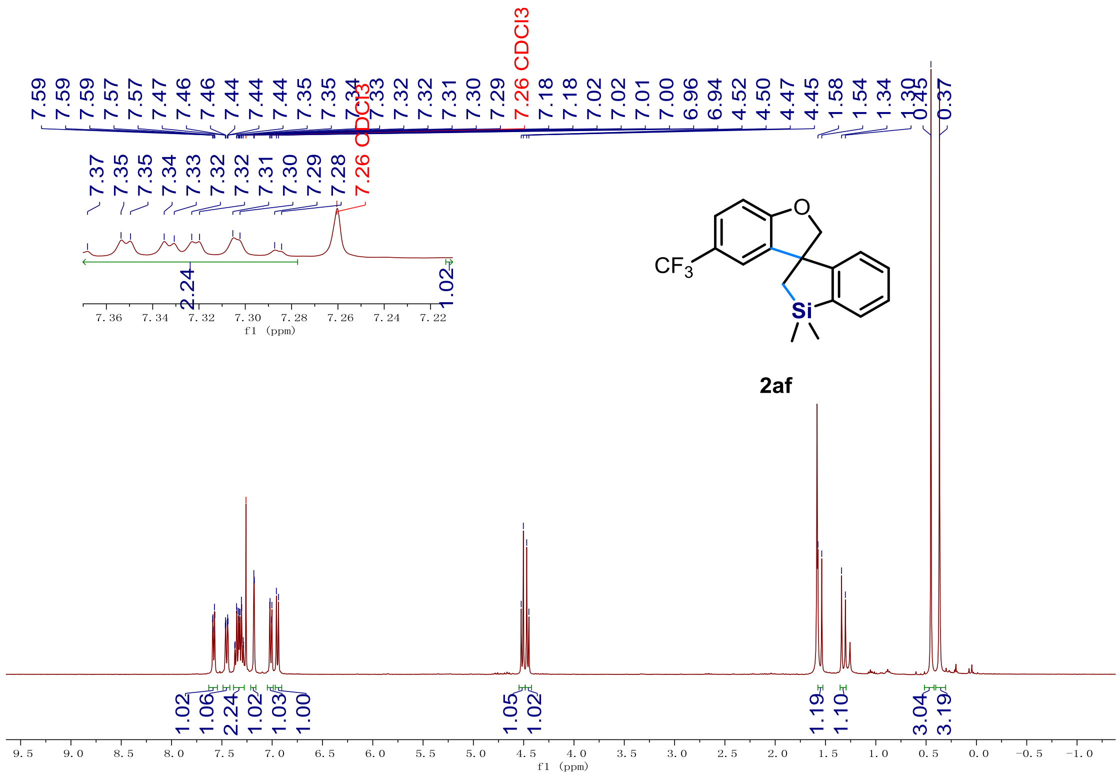

Supplementary Figure 106. ^{1}**H NMR (400 MHz, CDCl**$_3$**)** spectra of **2af**

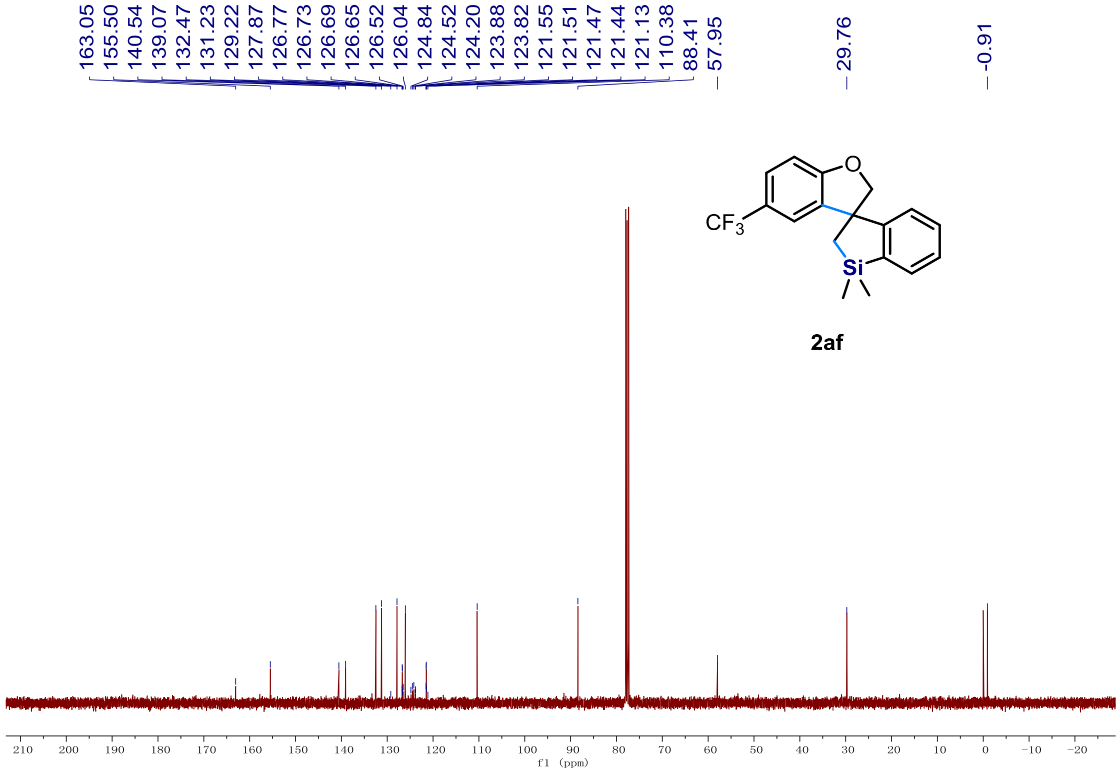

Supplementary Figure 107. ^{13}**C NMR (101 MHz, CDCl**$_3$**)** spectra of **2af**

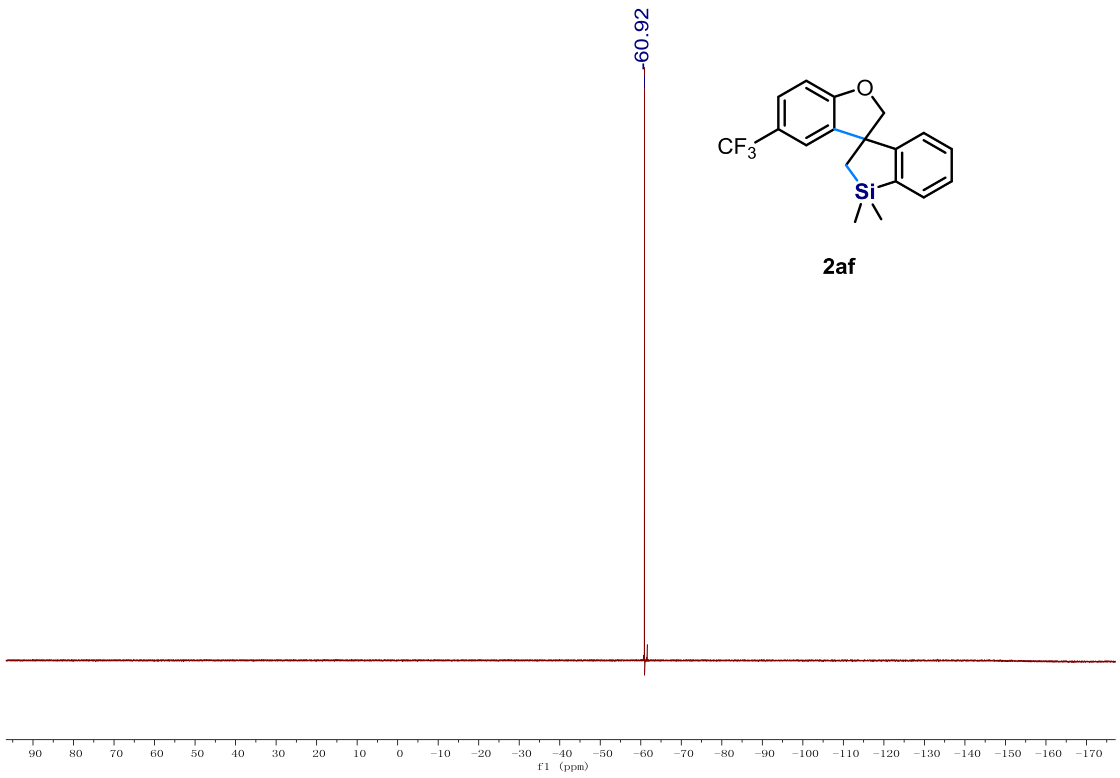

Supplementary Figure 108. ^{19}**F NMR (376 MHz, CDCl$_3$)** spectra of **2af**

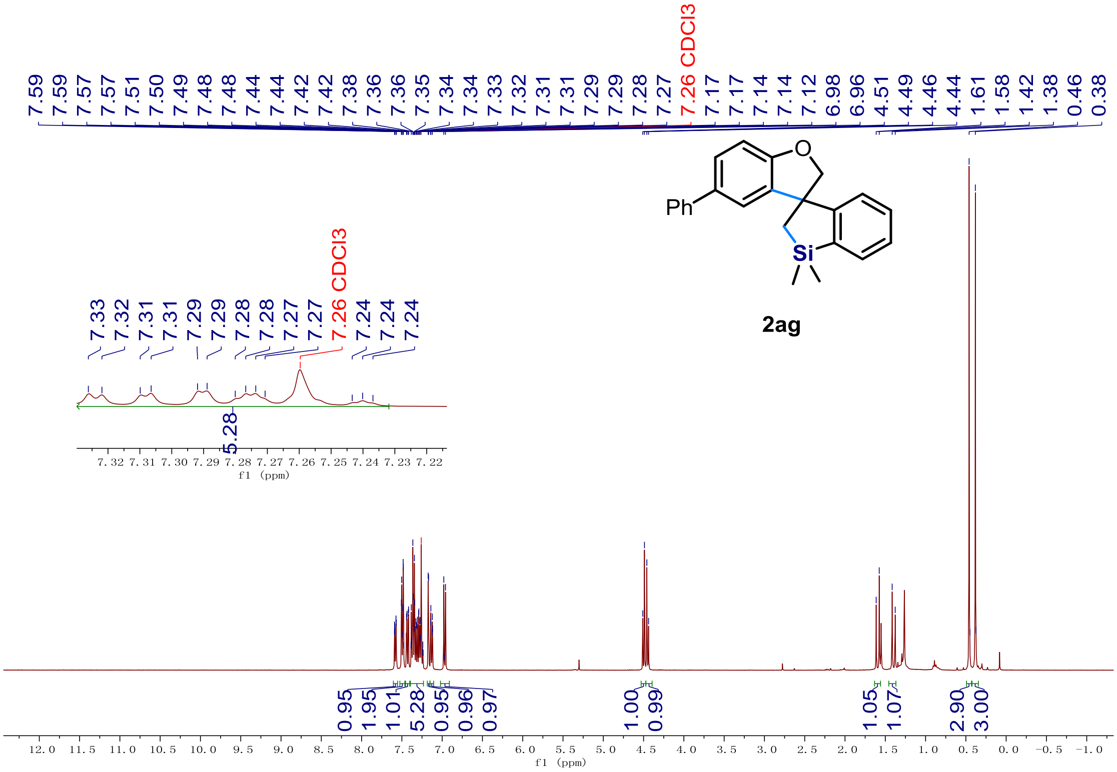

Supplementary Figure 109. ^{1}H NMR (400 MHz, $CDCl_3$) spectra of **2ag**

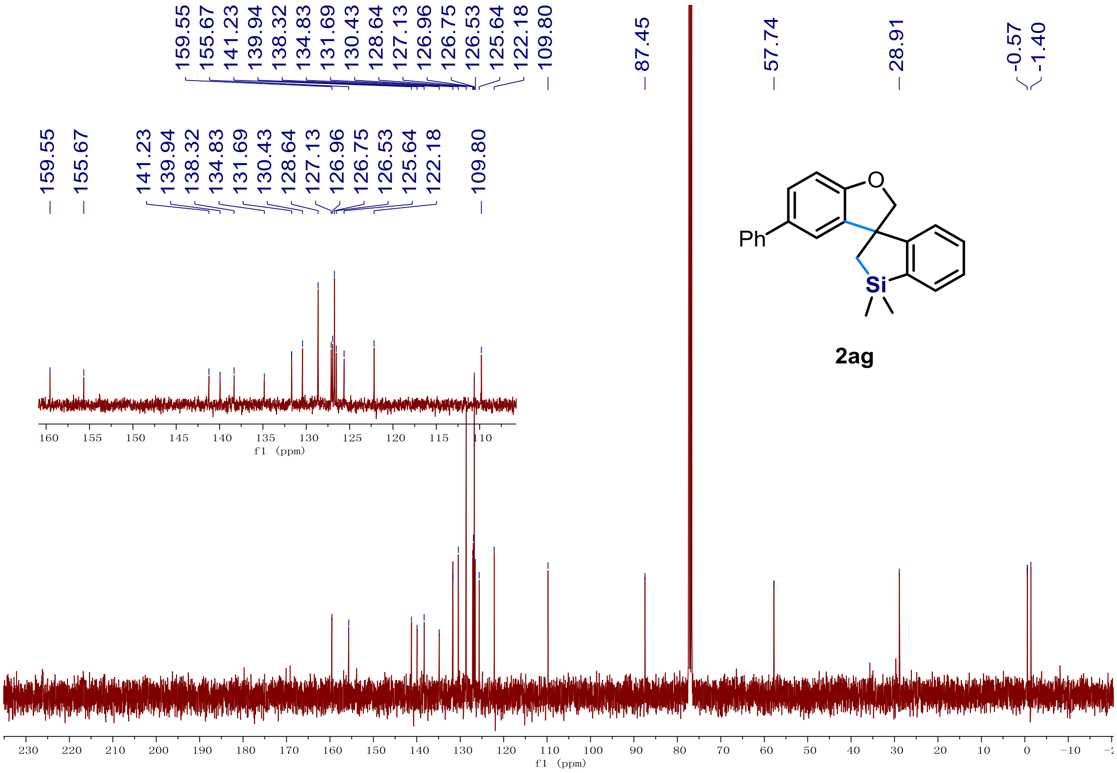

Supplementary Figure 110. ^{13}C NMR (101 MHz, $CDCl_3$) spectra of **2ag**

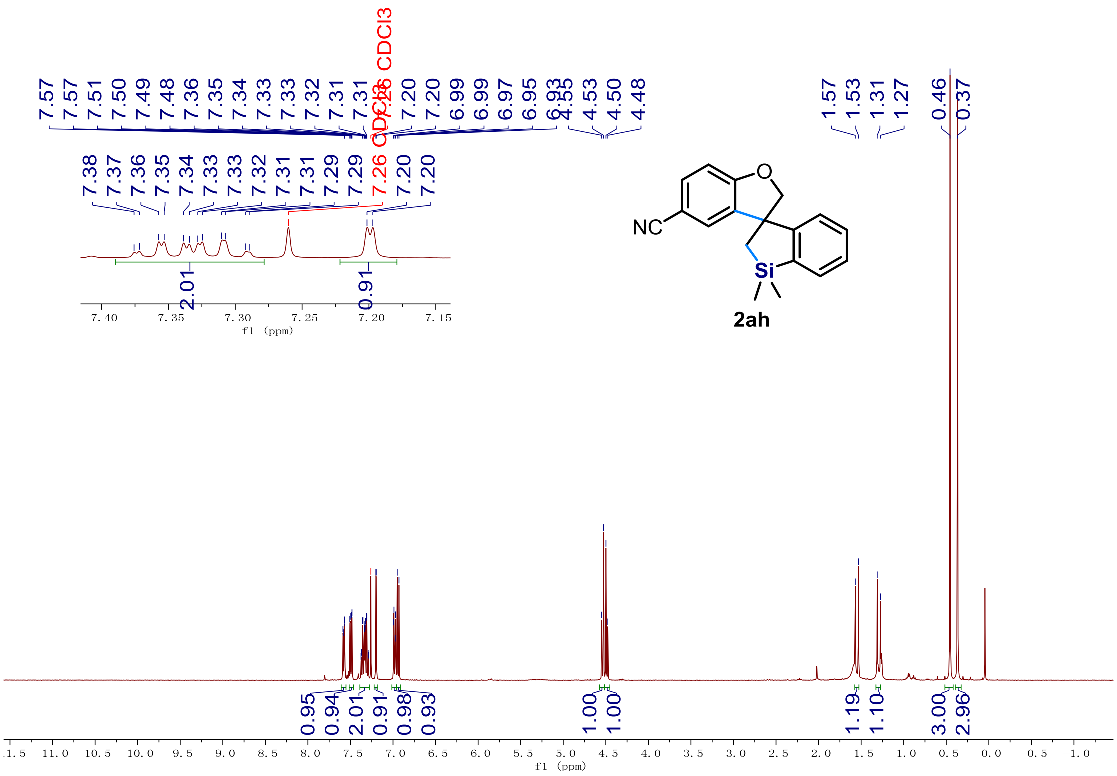

Supplementary Figure 111. ^{1}**H NMR (400 MHz, CDCl$_3$)** spectra of **2ah**

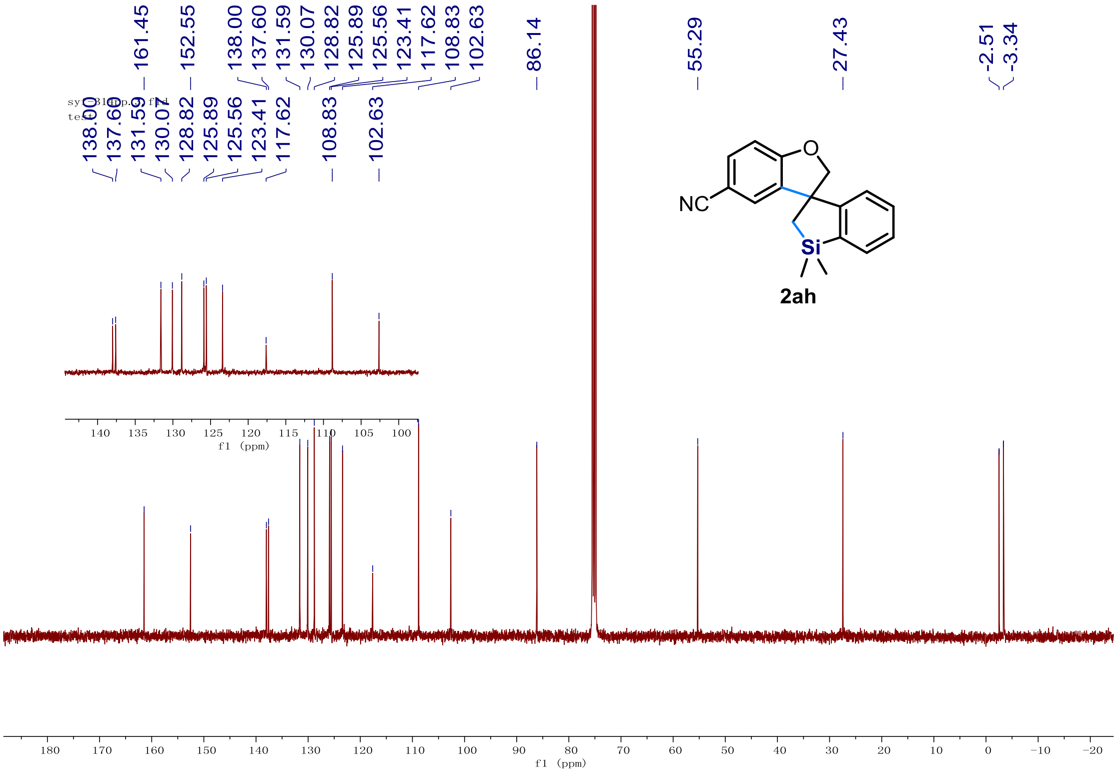

Supplementary Figure 112. ^{13}**C NMR (101 MHz, CDCl$_3$)** spectra of **2ah**

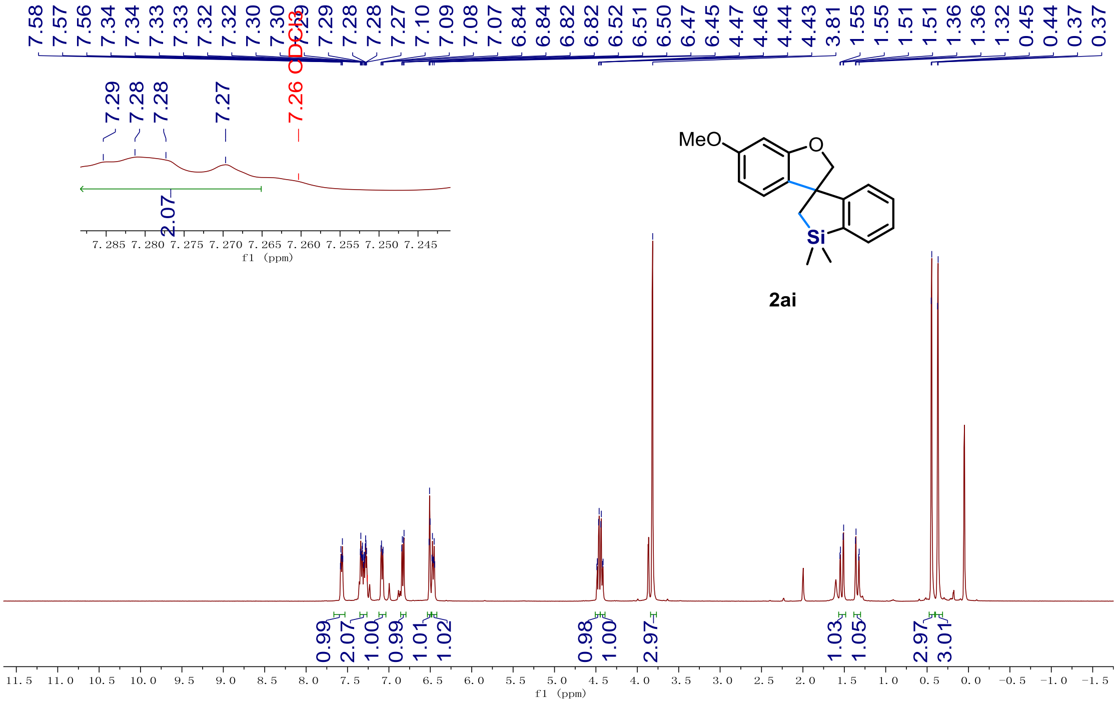

Supplementary Figure 113. ^{1}H NMR (400 MHz, CDCl$_3$) spectra of **2ai**

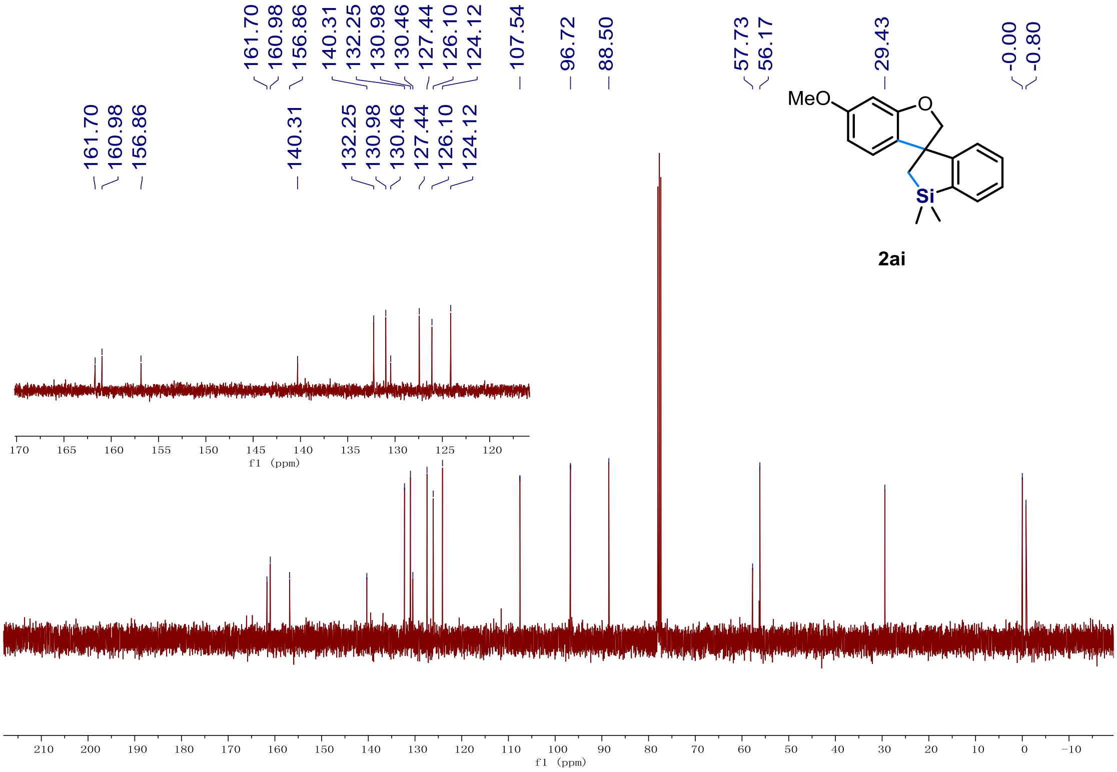

Supplementary Figure 114. ^{13}C NMR (101 MHz, CDCl$_3$) spectra of **2ai**

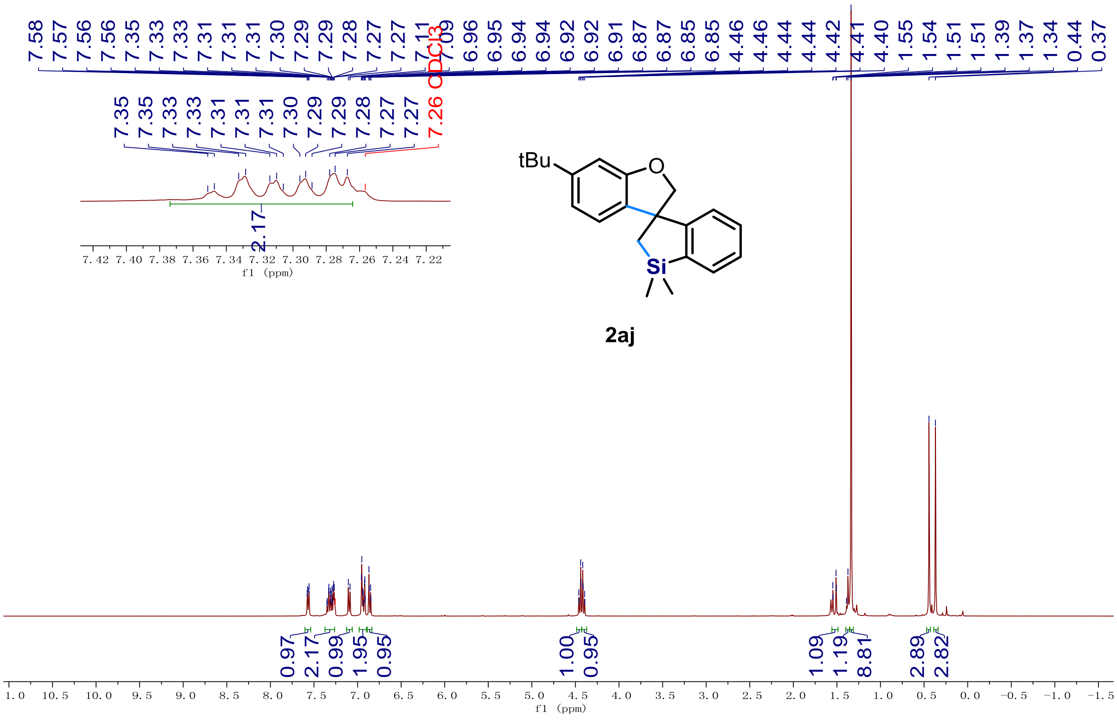

Supplementary Figure 115. **^{1}H NMR (400 MHz, $CDCl_3$)** spectra of **2aj**

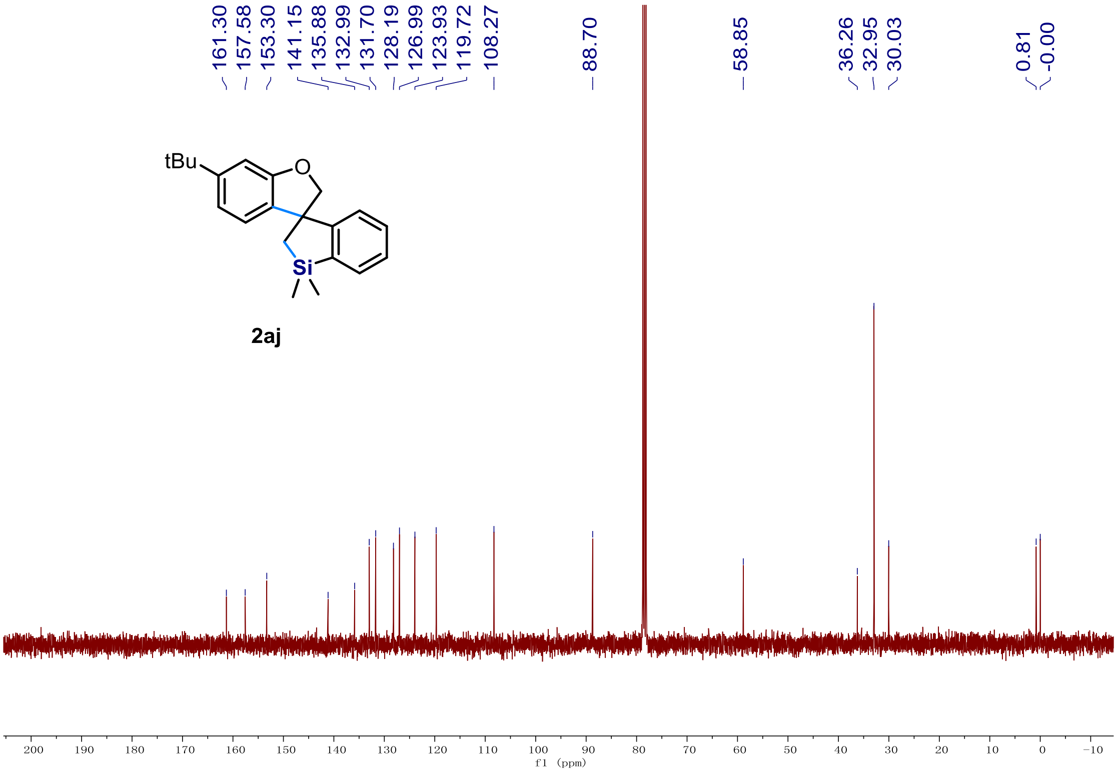

Supplementary Figure 116. **^{13}C NMR (101 MHz, $CDCl_3$)** spectra of **2aj**

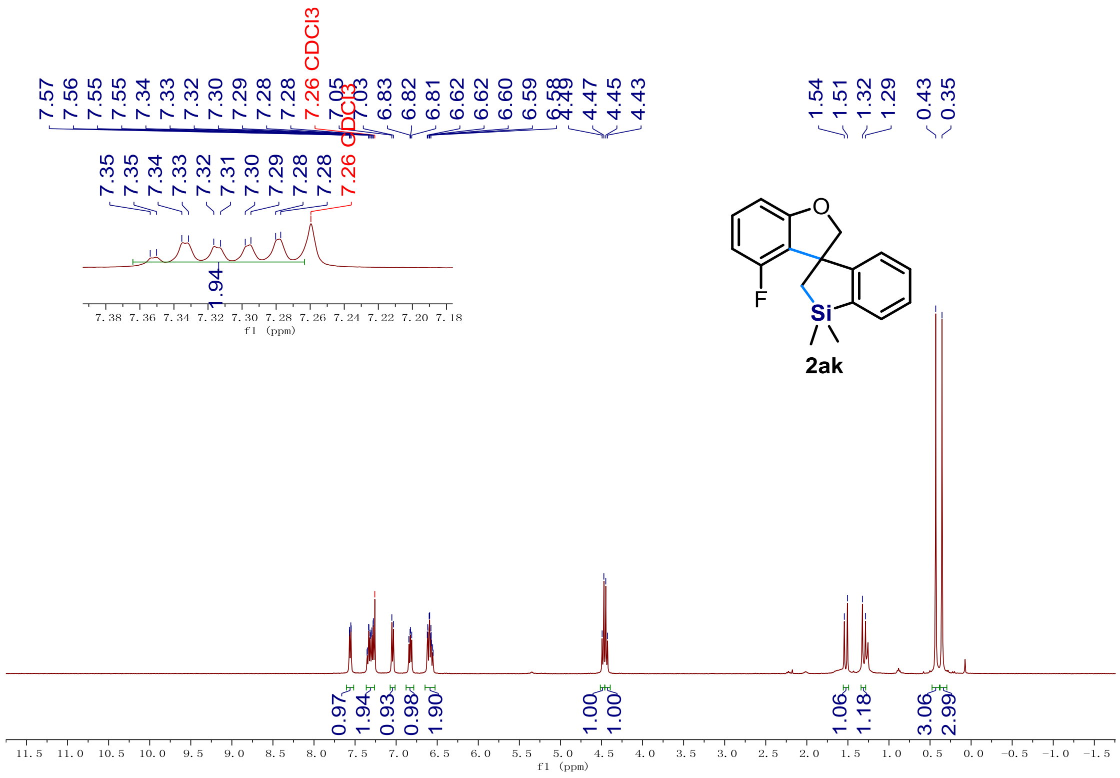

Supplementary Figure 117. **^{1}H NMR (400 MHz, CDCl$_3$)** spectra of **2ak**

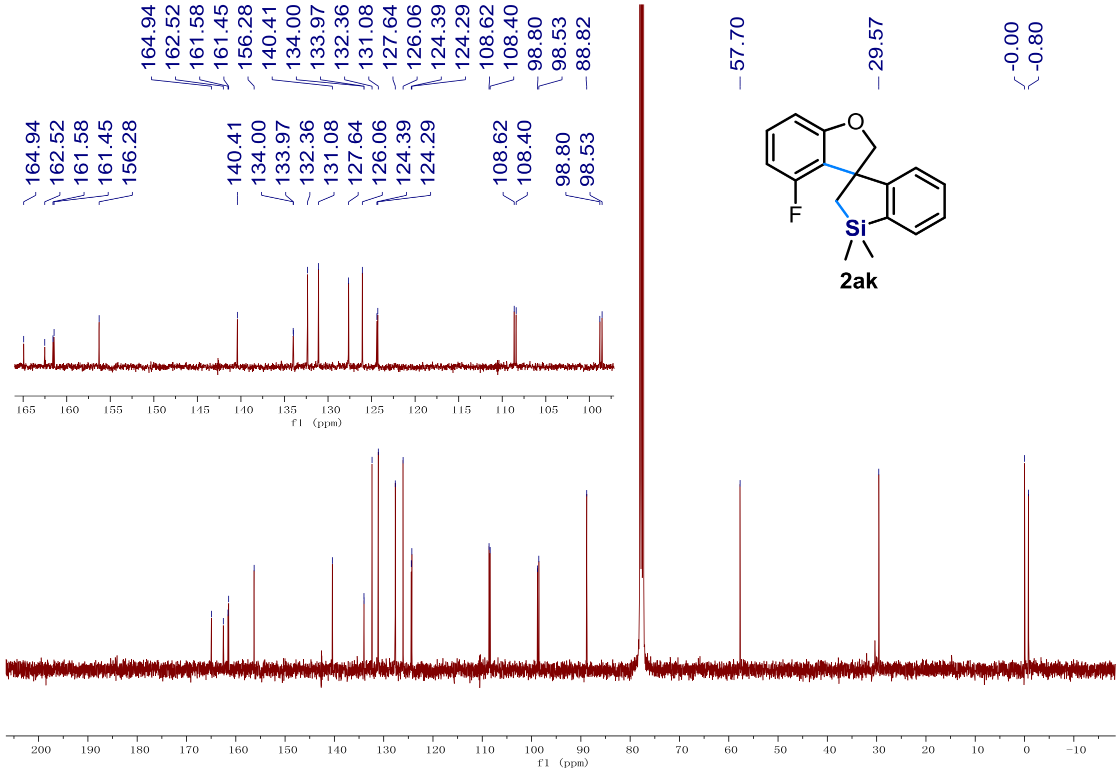

Supplementary Figure 118. **^{13}C NMR (101 MHz, CDCl$_3$)** spectra of **2aj**

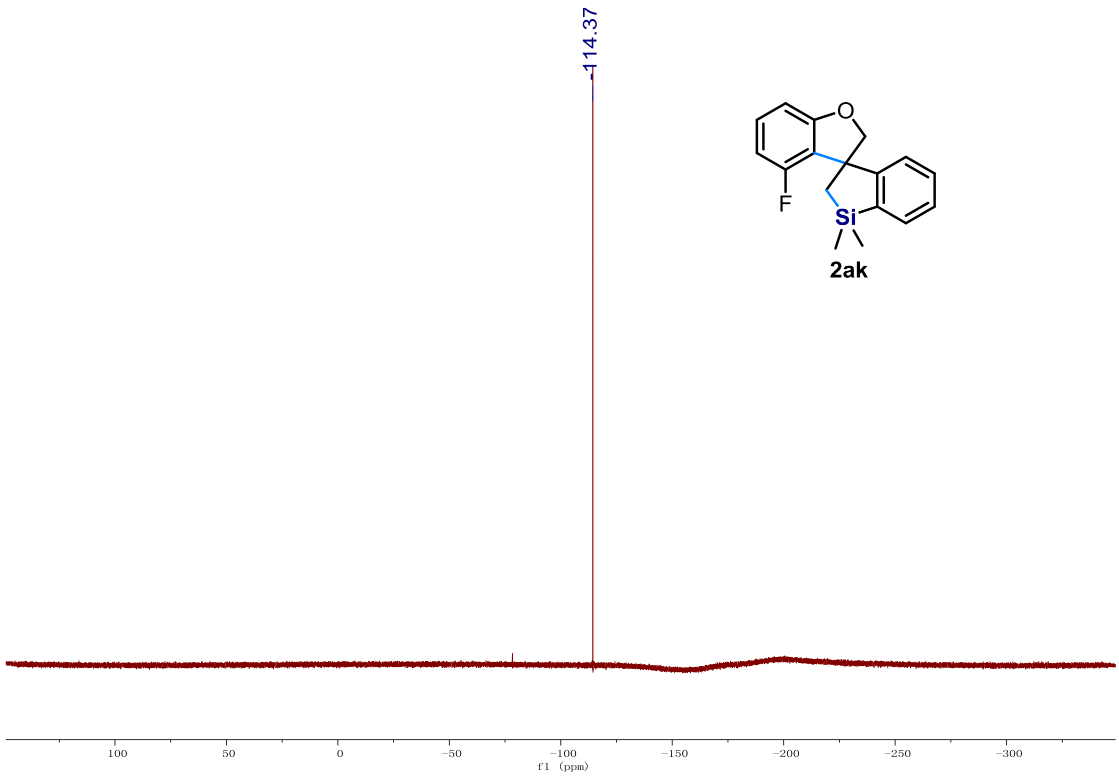

Supplementary Figure 119. ^{19}F NMR (376 MHz, $CDCl_3$) spectra of **2ak**

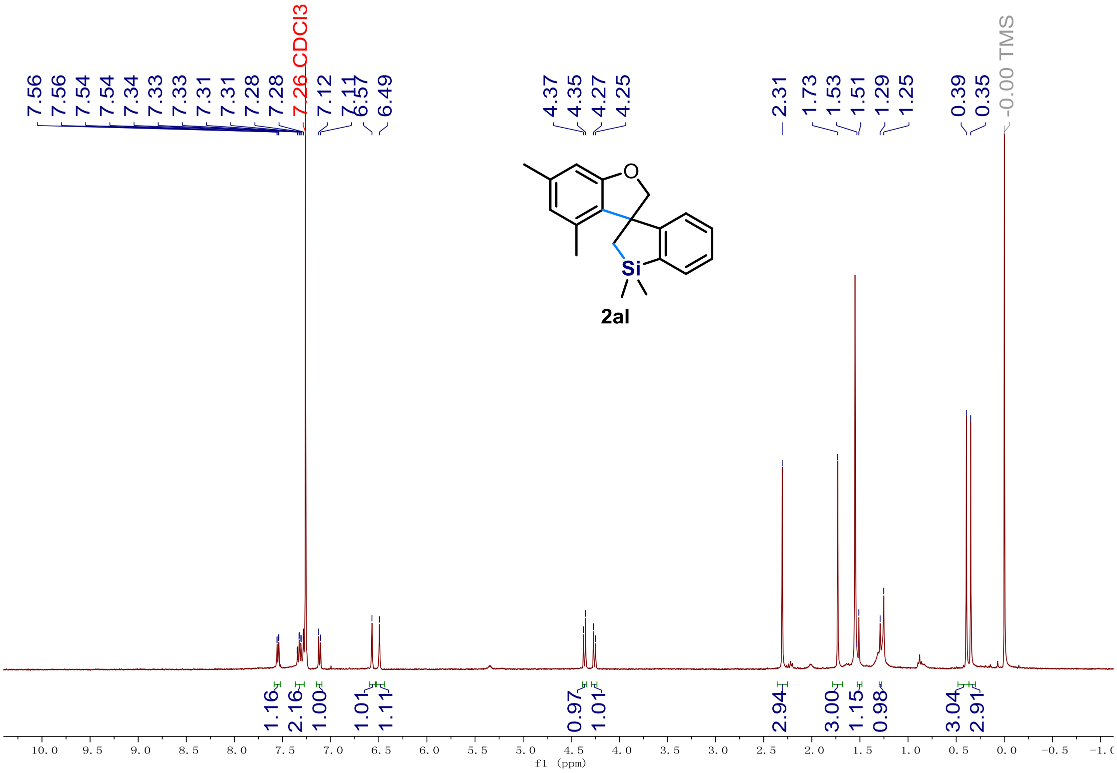

Supplementary Figure 120. ^{1}**H NMR (400 MHz, CDCl$_3$)** spectra of **2al**

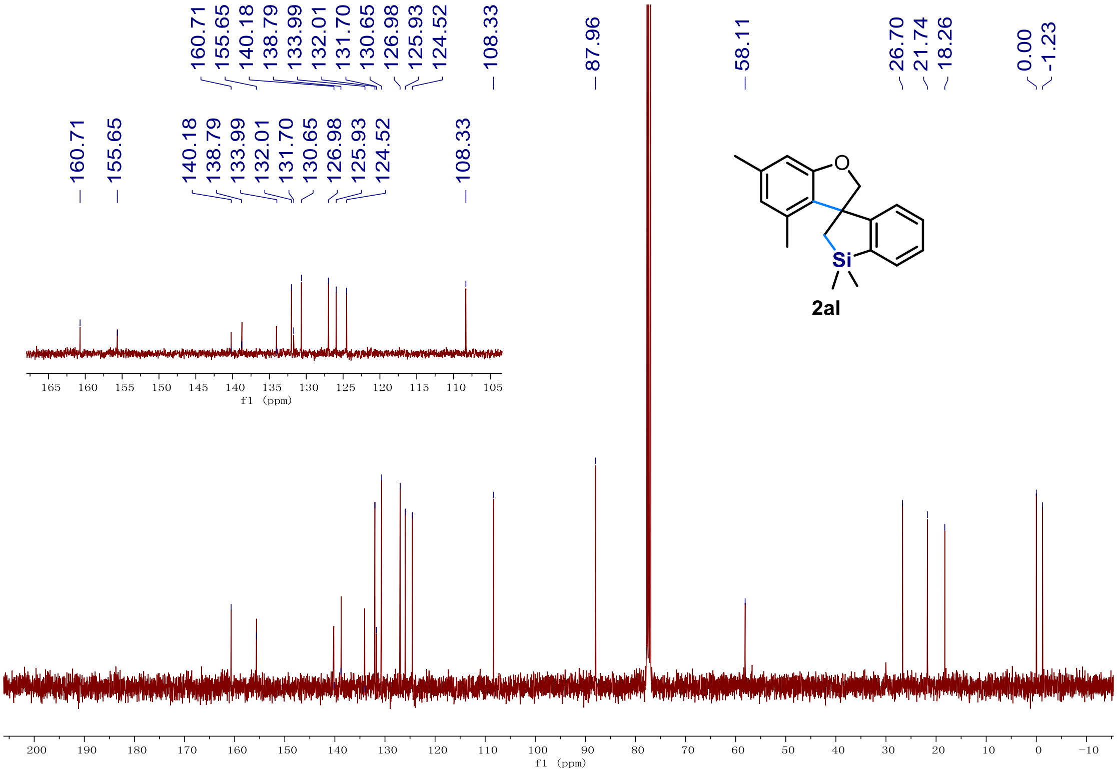

Supplementary Figure 121. ^{13}**C NMR (101 MHz, CDCl$_3$)** spectra of **2al**

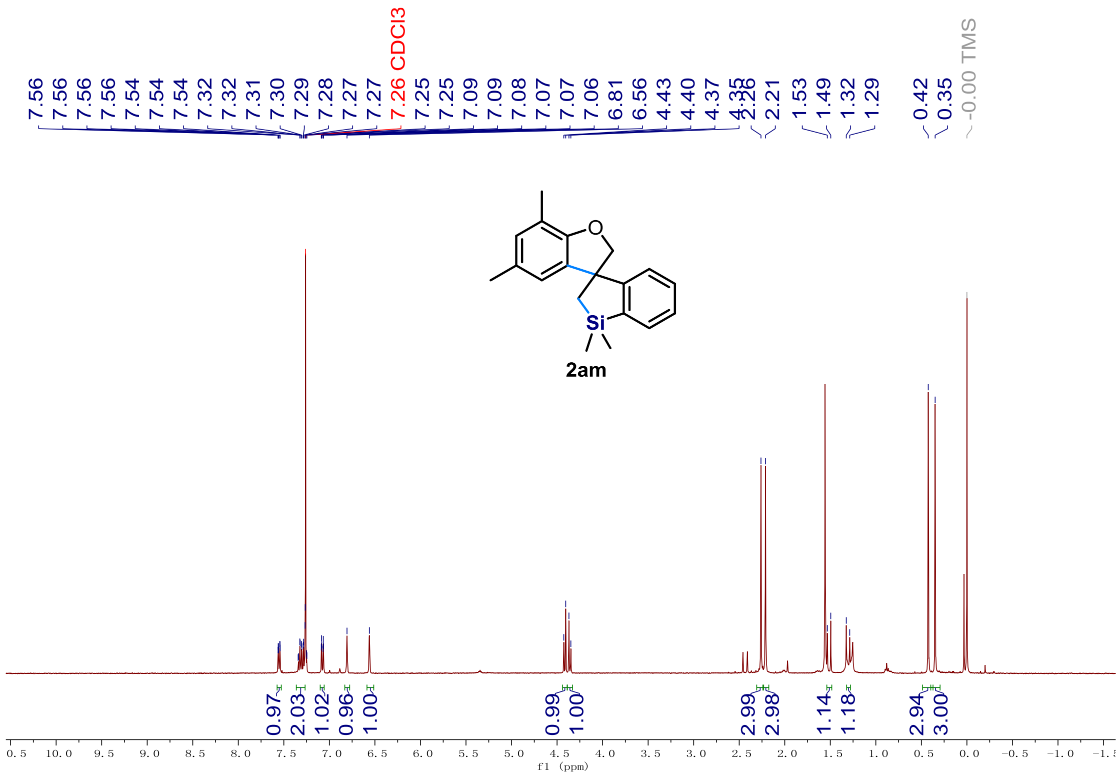

Supplementary Figure 122. **^{1}H NMR (400 MHz, CDCl$_3$)** spectra of **2am**

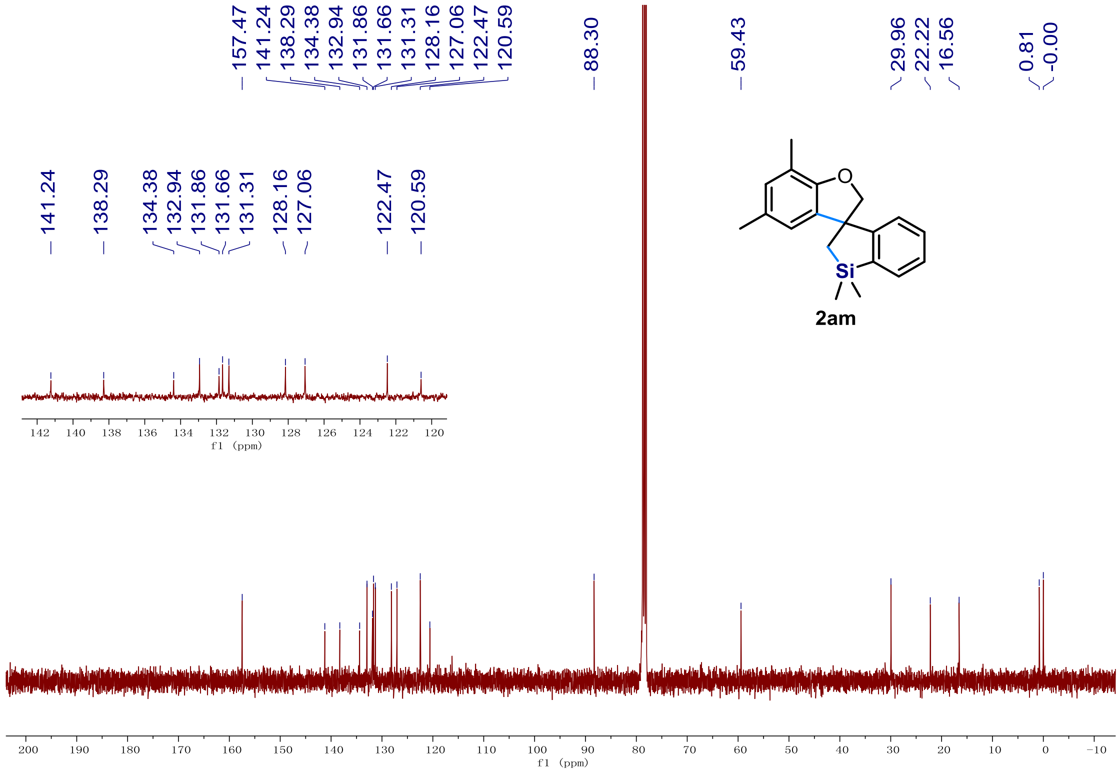

Supplementary Figure 123. **^{13}C NMR (101 MHz, CDCl$_3$)** spectra of **2am**

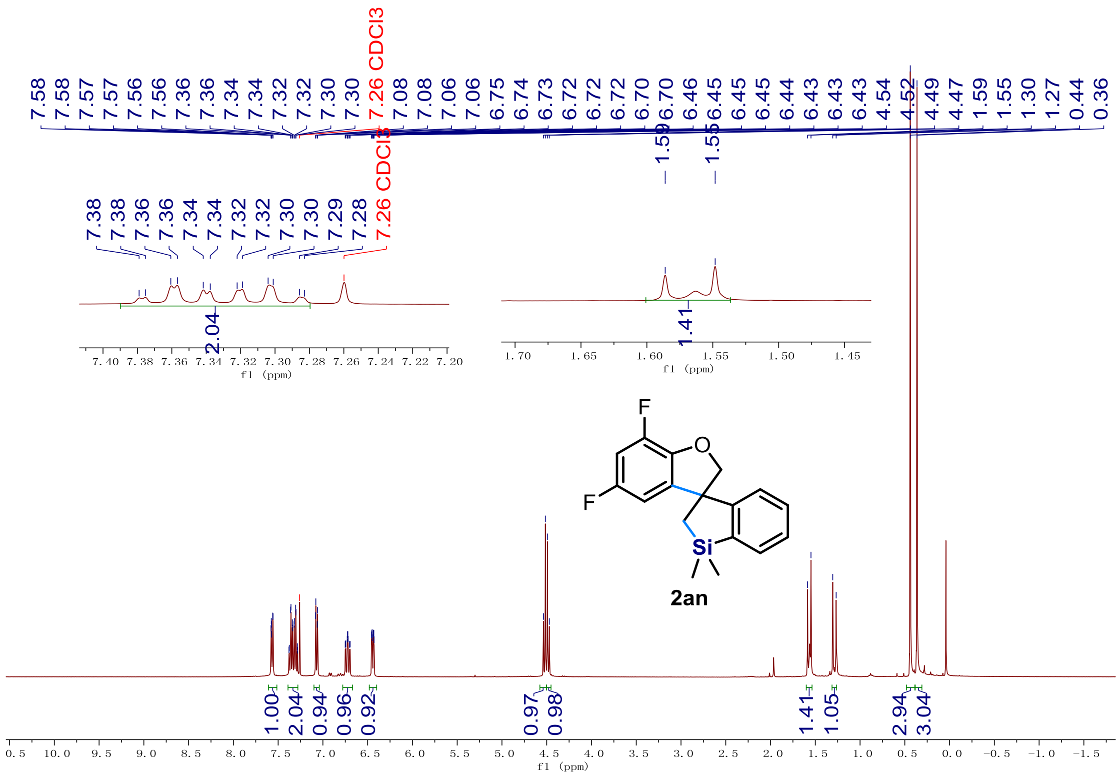

Supplementary Figure 124. ^{1}**H NMR (400 MHz, CDCl$_3$)** spectra of **2an**

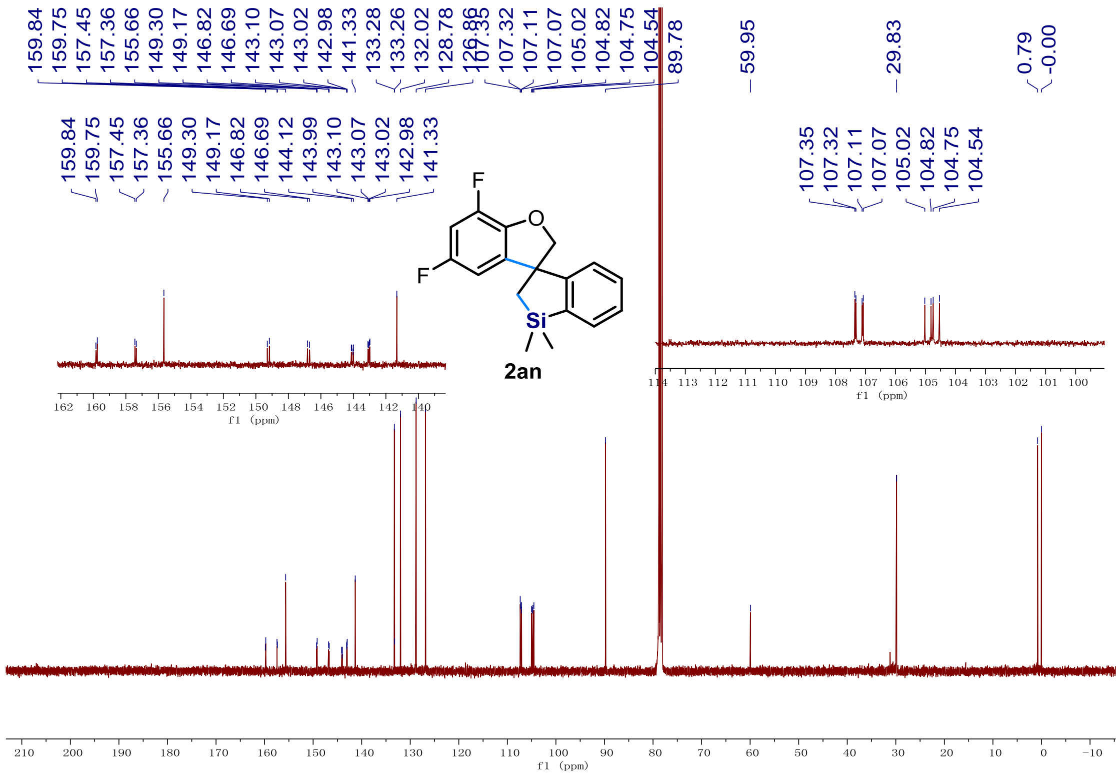

Supplementary Figure 125. ^{13}**C NMR (101 MHz, CDCl$_3$)** spectra of **2an**

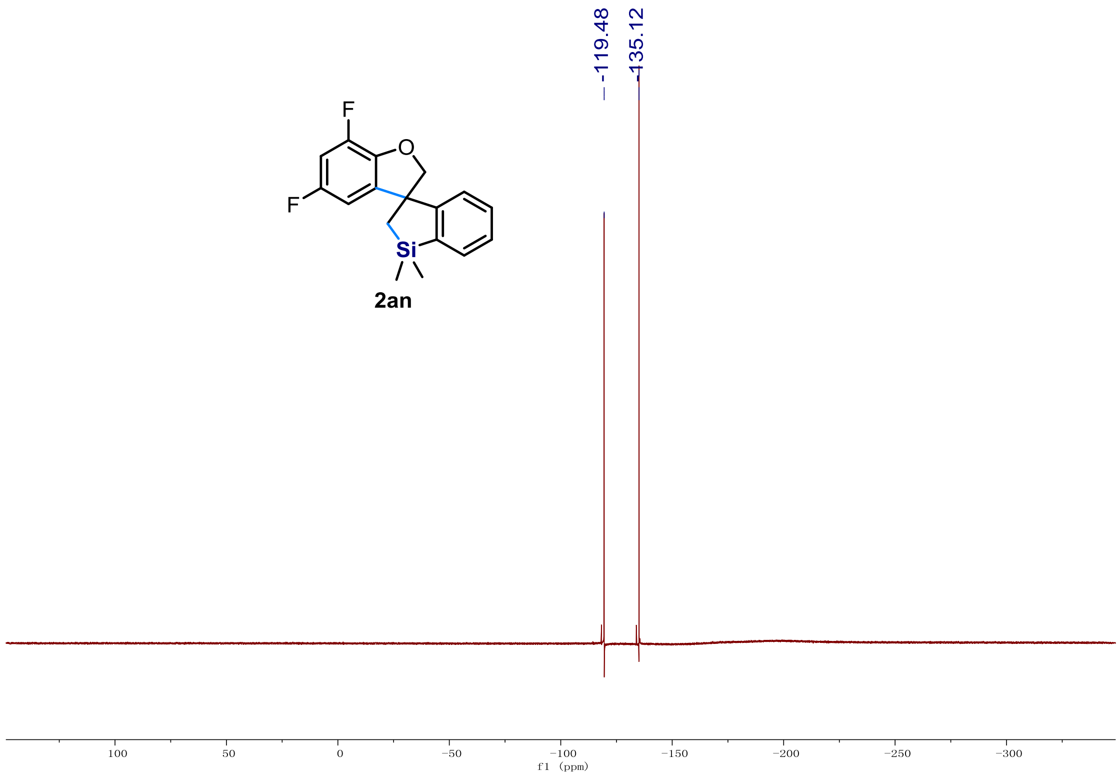

Supplementary Figure 126. ^{19}**F NMR (376 MHz,** $CDCl_3$**)** spectra of **2an**

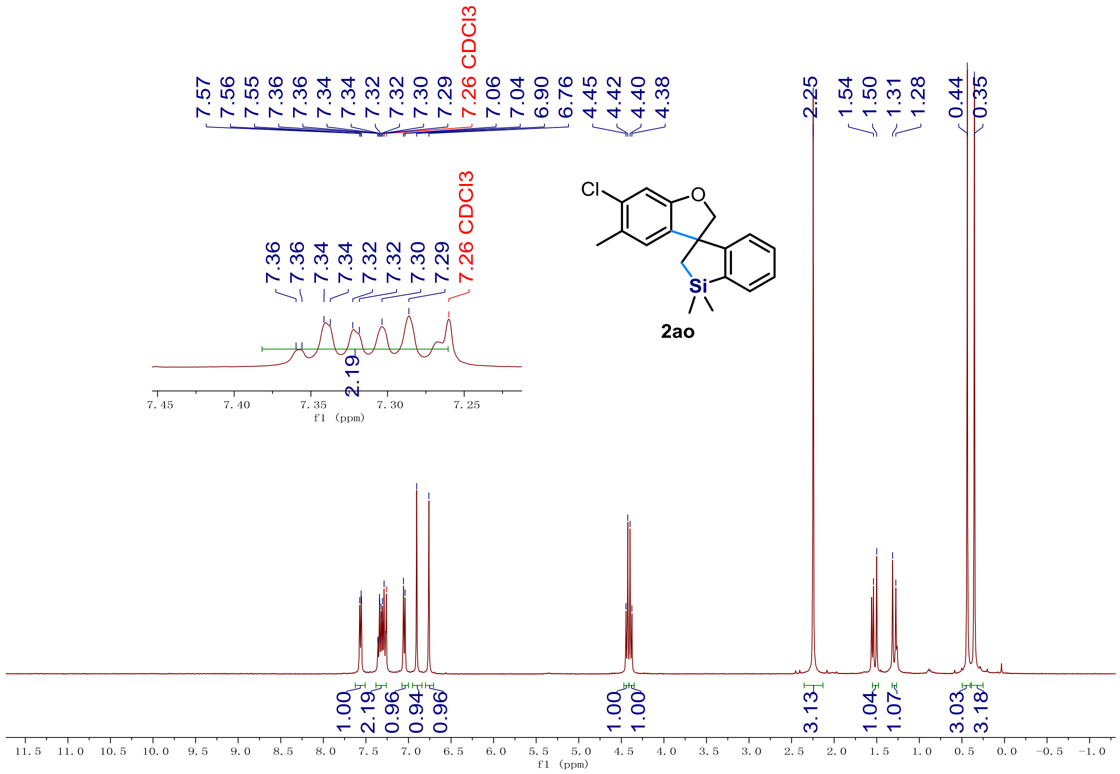

Supplementary Figure 127. ^{1}**H NMR (400 MHz, CDCl**$_3$**)** spectra of **2ao**

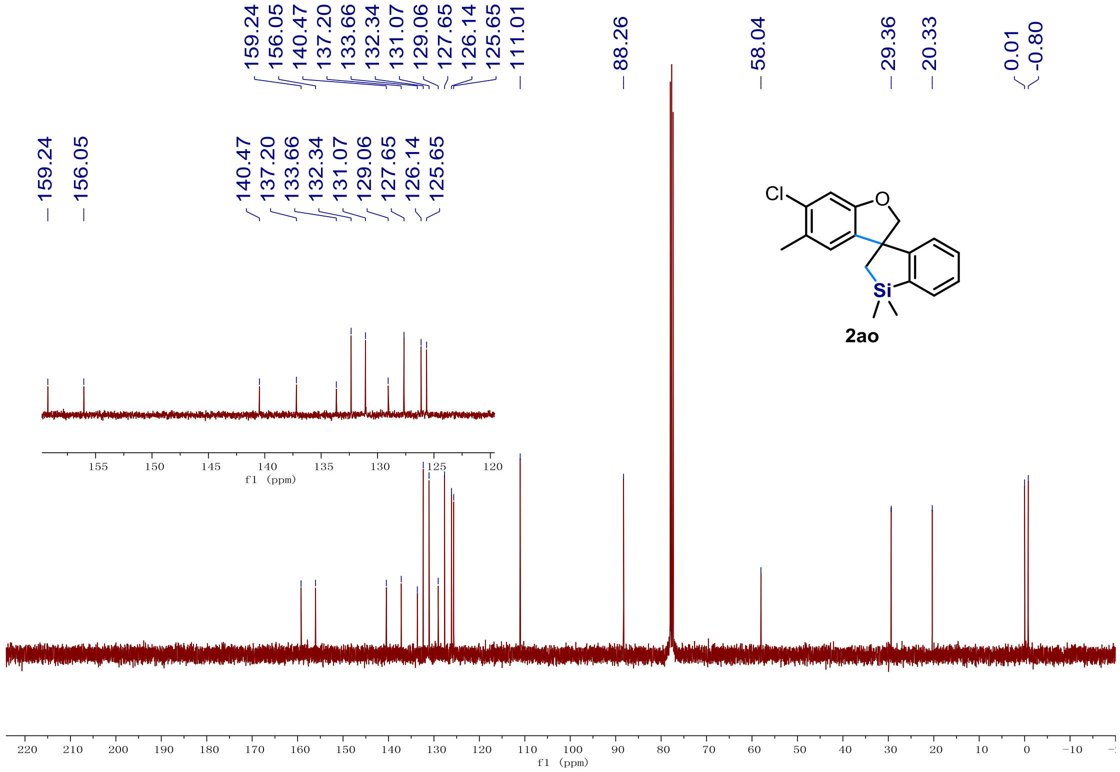

Supplementary Figure 128. ^{13}**C NMR (101 MHz, CDCl**$_3$**)** spectra of **2ao**

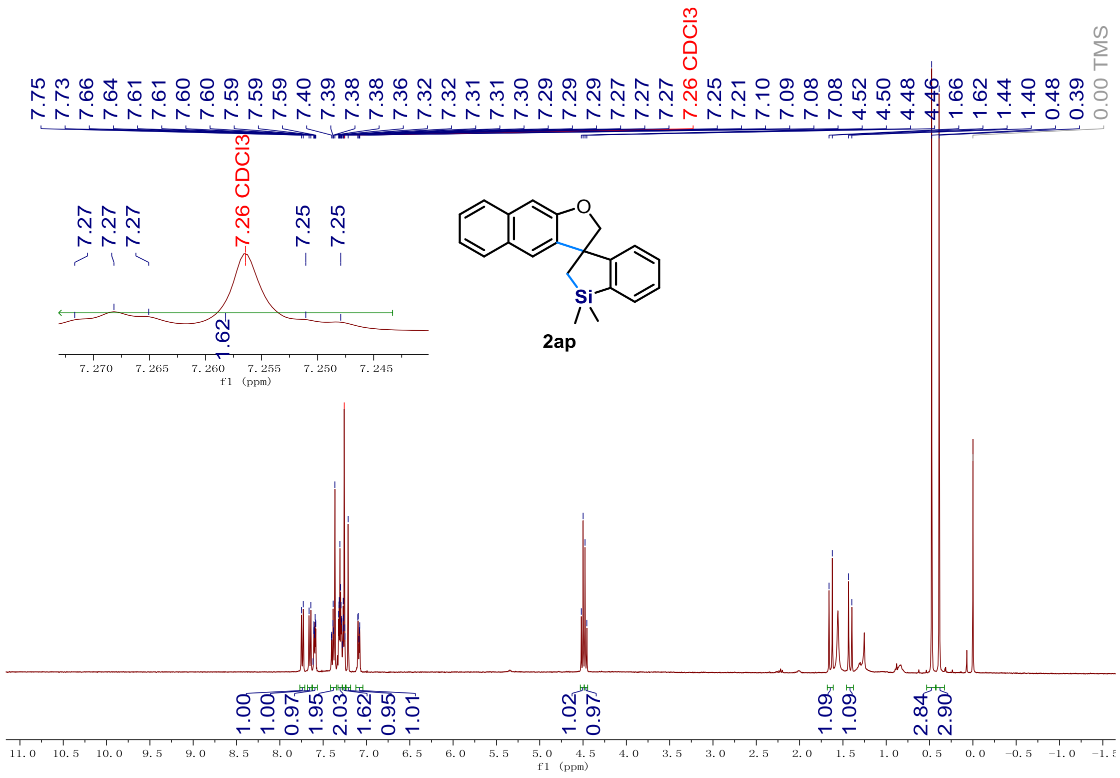

Supplementary Figure 129. ^{1}**H NMR (400 MHz, CDCl$_3$)** spectra of **2ap**

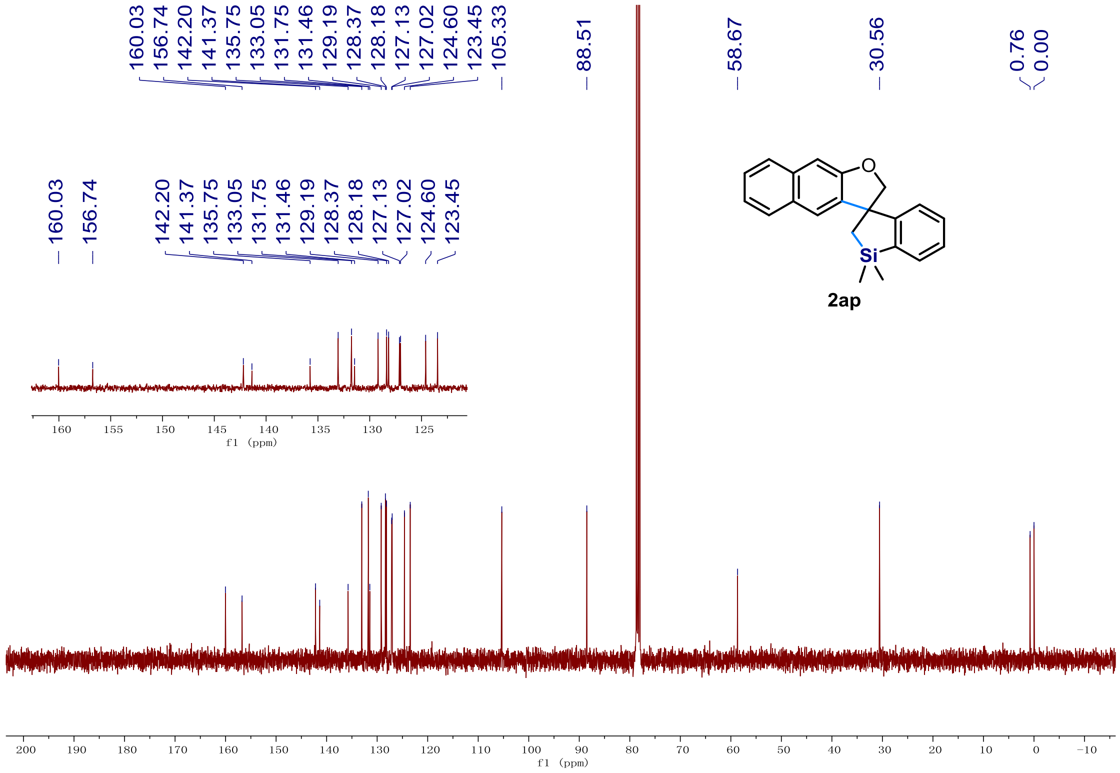

Supplementary Figure 130. ^{13}**C NMR (101 MHz, CDCl$_3$)** spectra of **2ap**

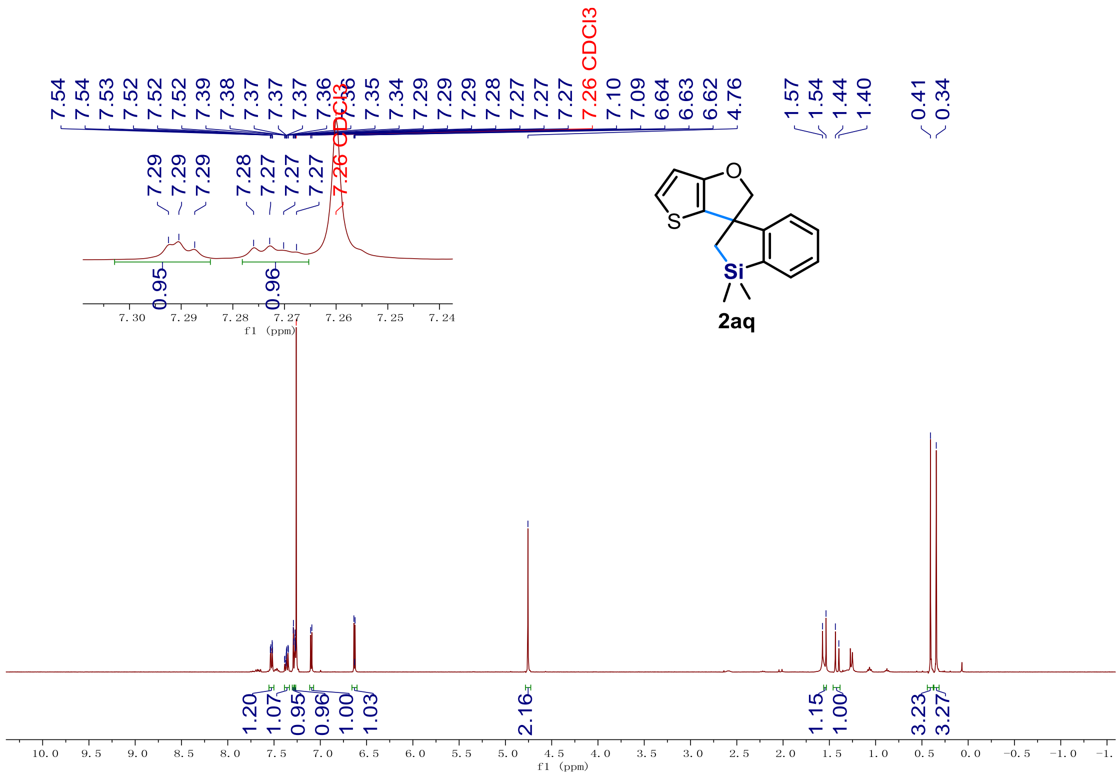

Supplementary Figure 131. **^{1}H NMR (400 MHz, CDCl$_3$)** spectra of **2aq**

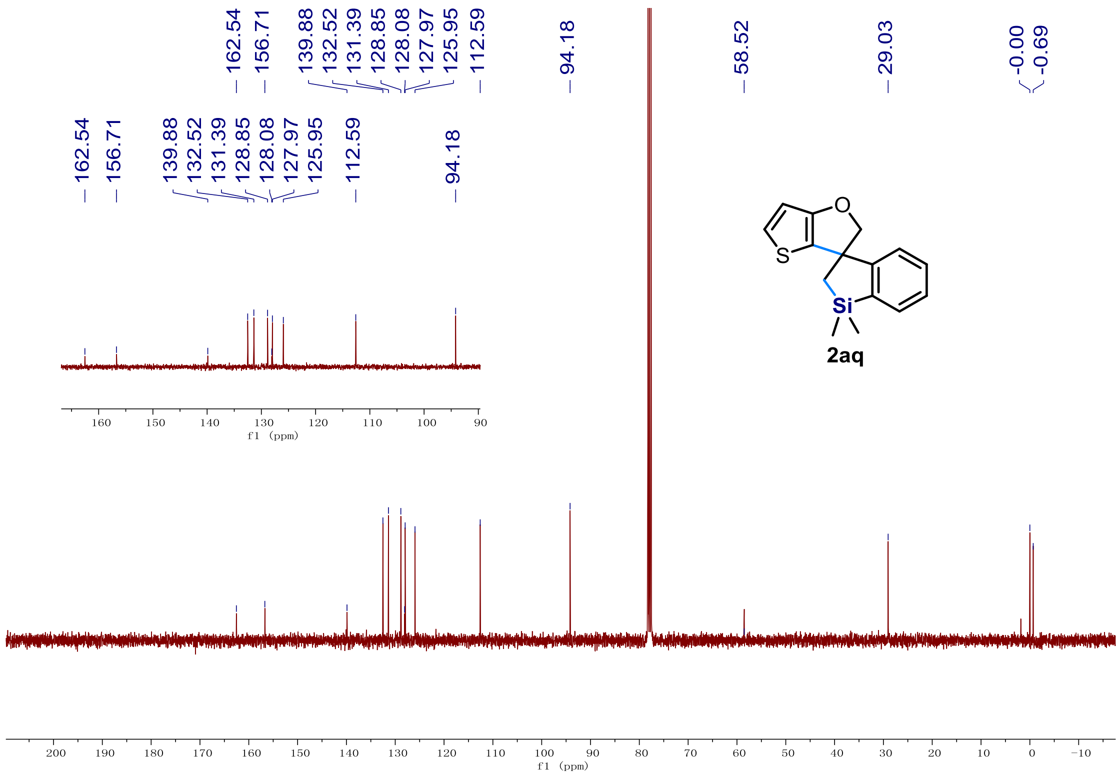

Supplementary Figure 132. **^{13}C NMR (101 MHz, CDCl$_3$)** spectra of **2aq**

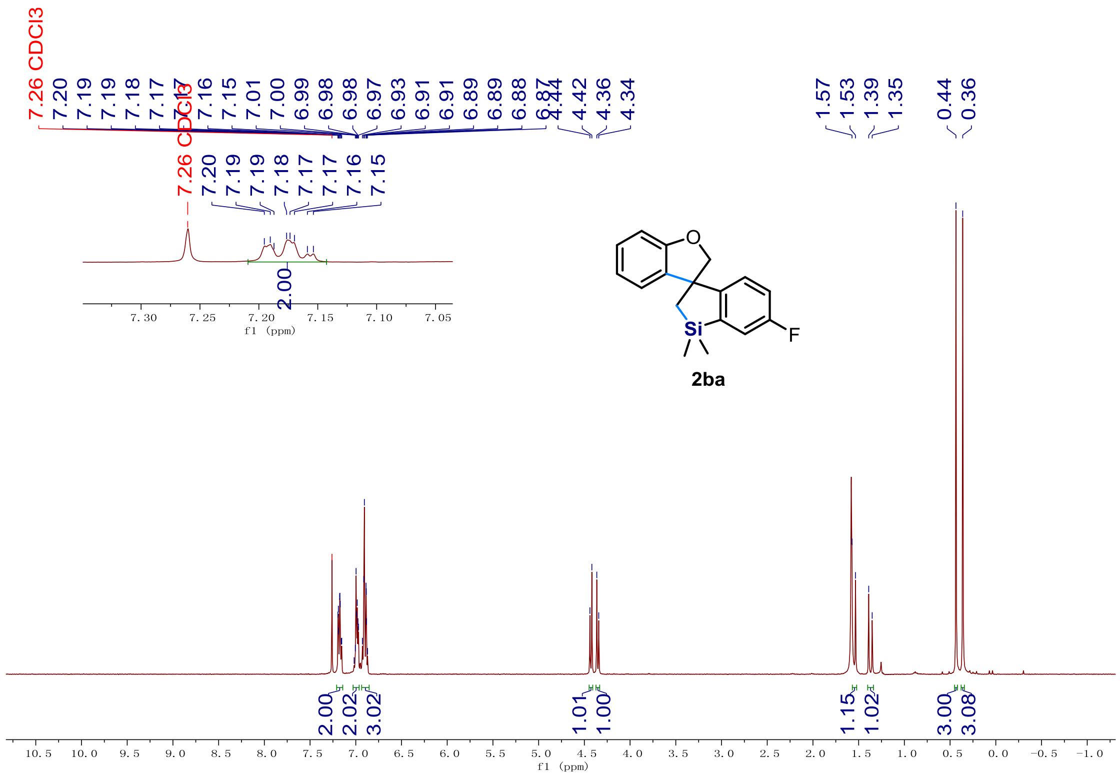

Supplementary Figure 133. **^{1}H NMR (400 MHz, $CDCl_3$)** spectra of **2ba**

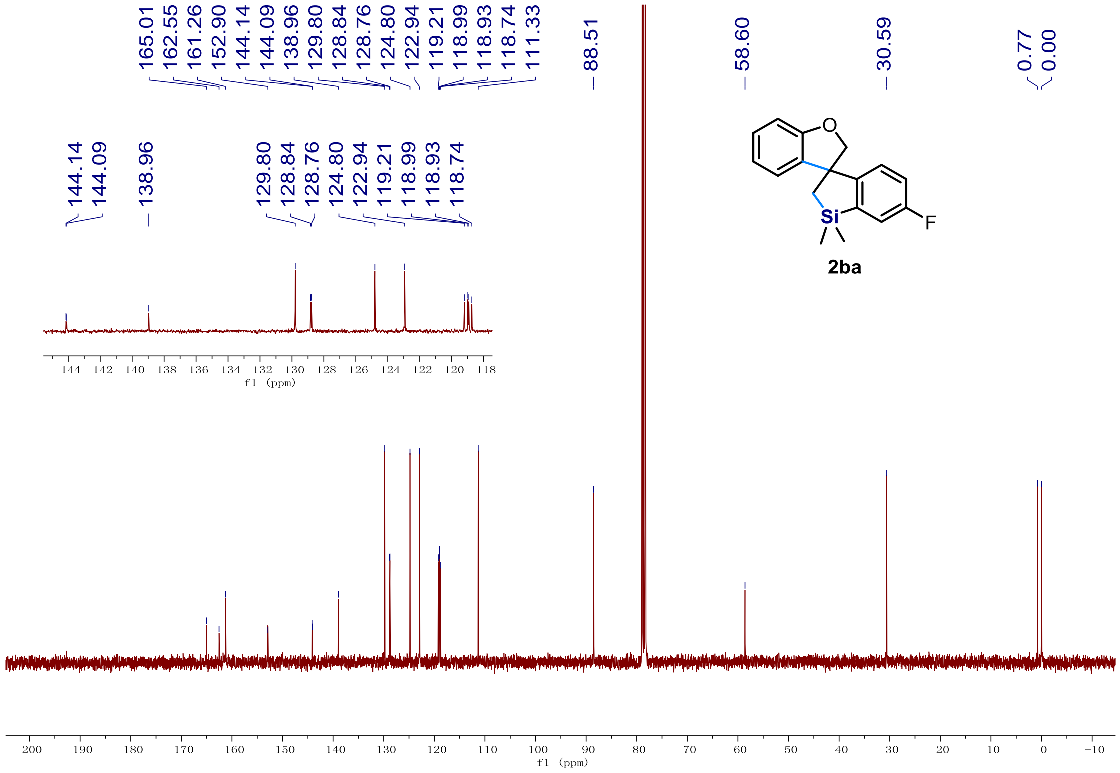

Supplementary Figure 134. **^{13}C NMR (101 MHz, $CDCl_3$)** spectra of **2ba**

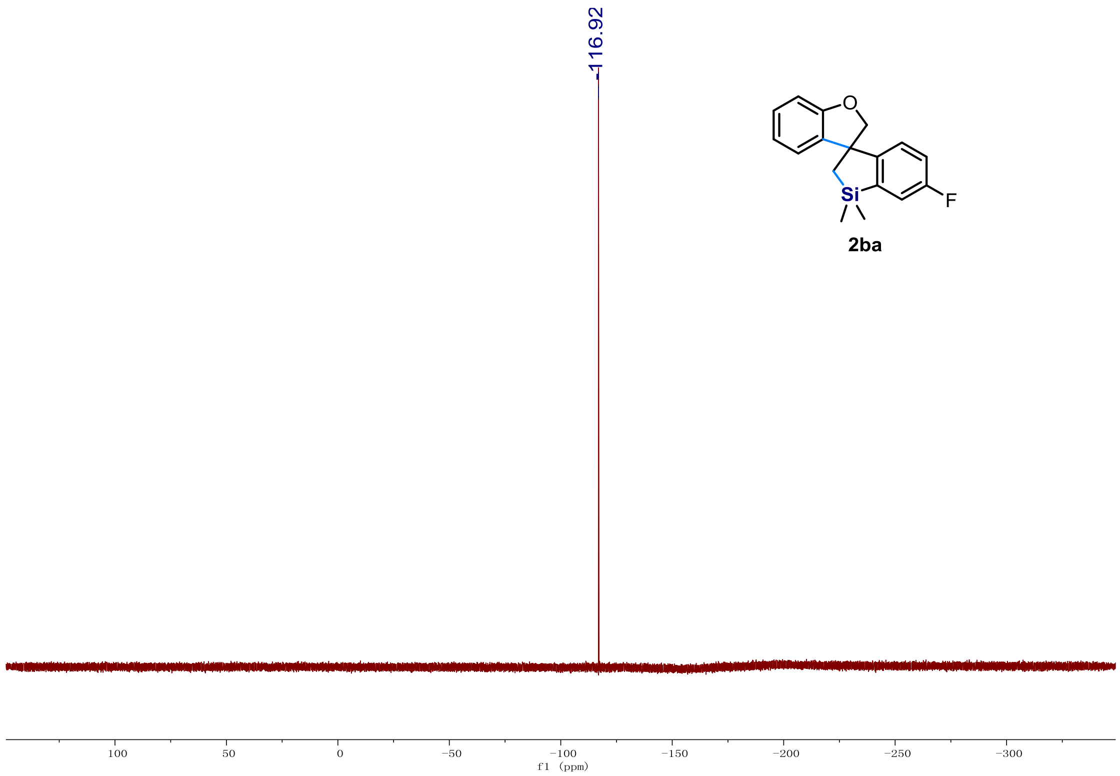

Supplementary Figure 135. ^{19}**F NMR (376 MHz, CDCl$_3$)** spectra of **2ba**

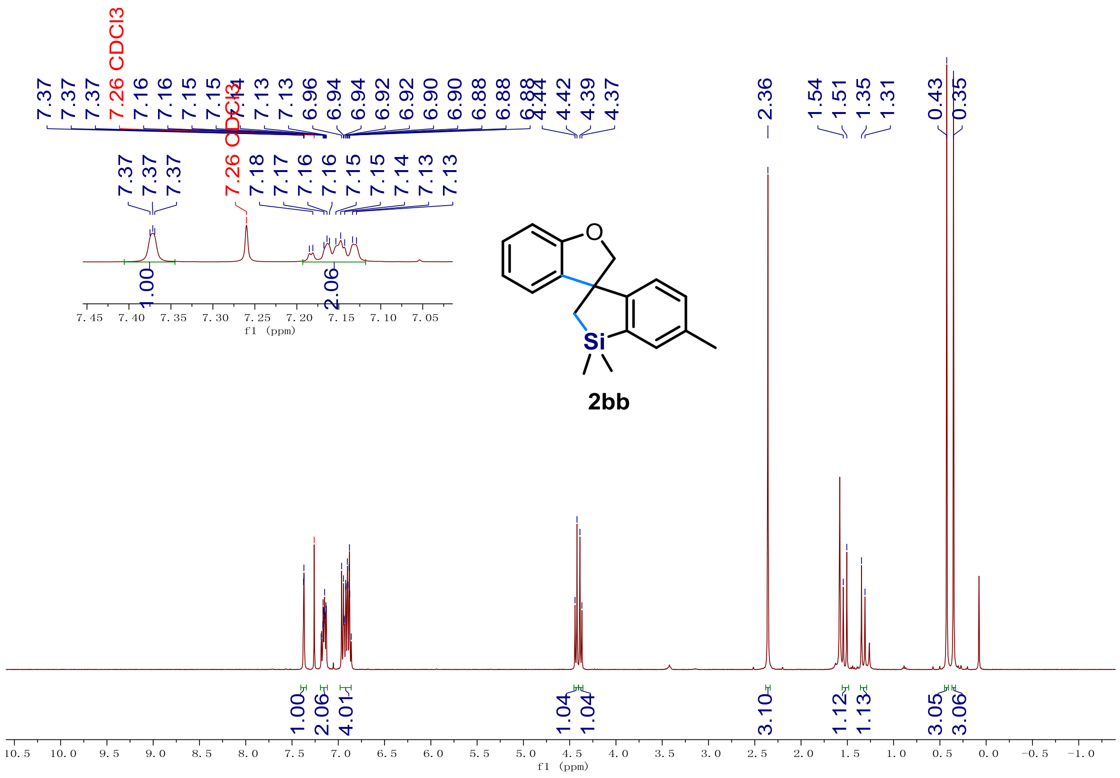

Supplementary Figure 136. ^{1}**H NMR (400 MHz, CDCl**$_3$**)** spectra of **2bb**

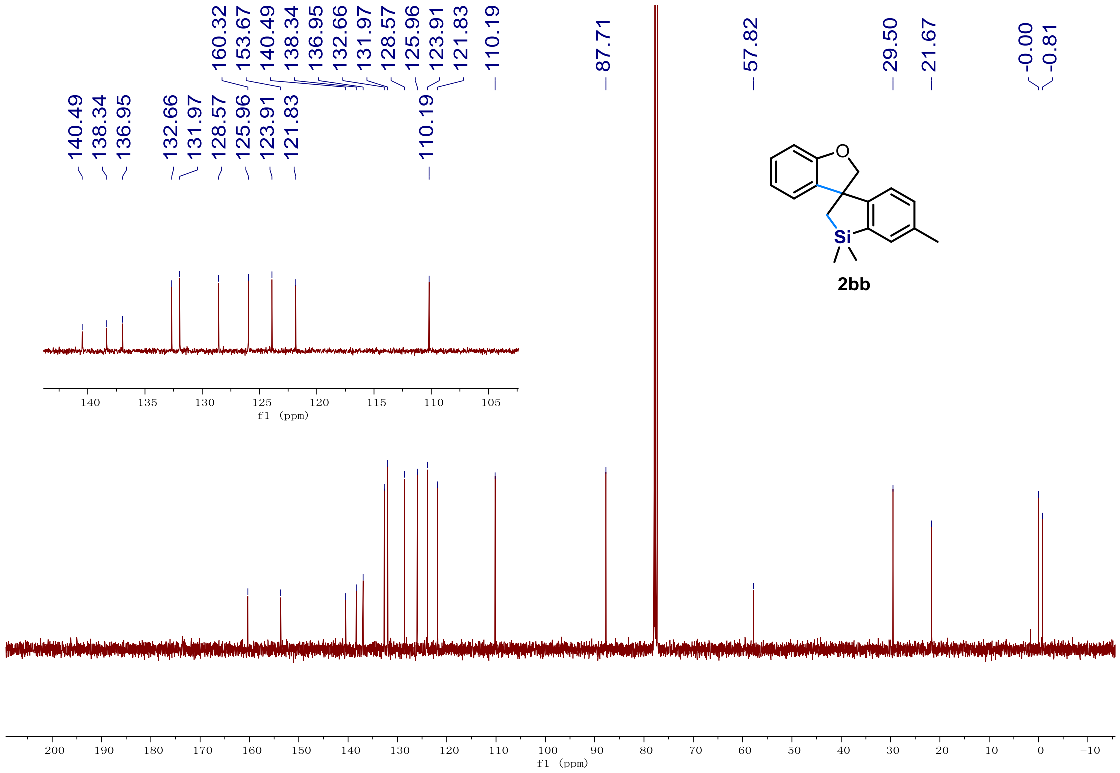

Supplementary Figure 137. ^{13}**C NMR (101 MHz, CDCl**$_3$**)** spectra of **2bb**

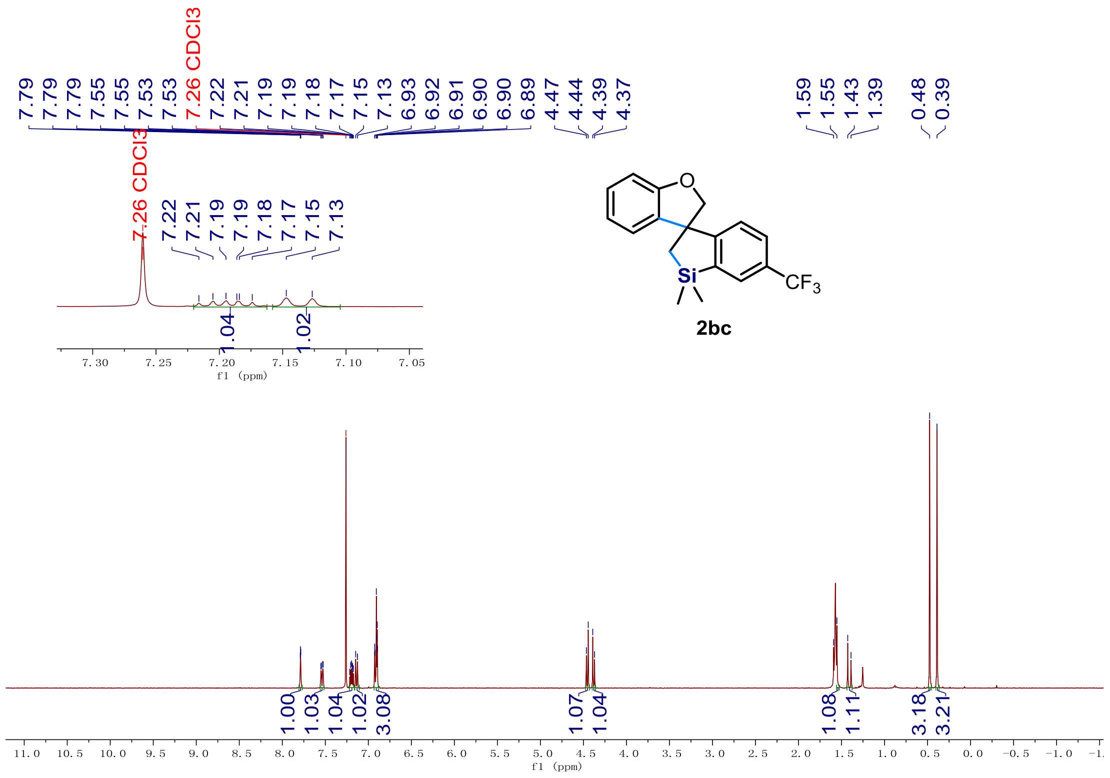

Supplementary Figure 138. ^{1}H **NMR (400 MHz,** $CDCl_3$**)** spectra of **2bc**

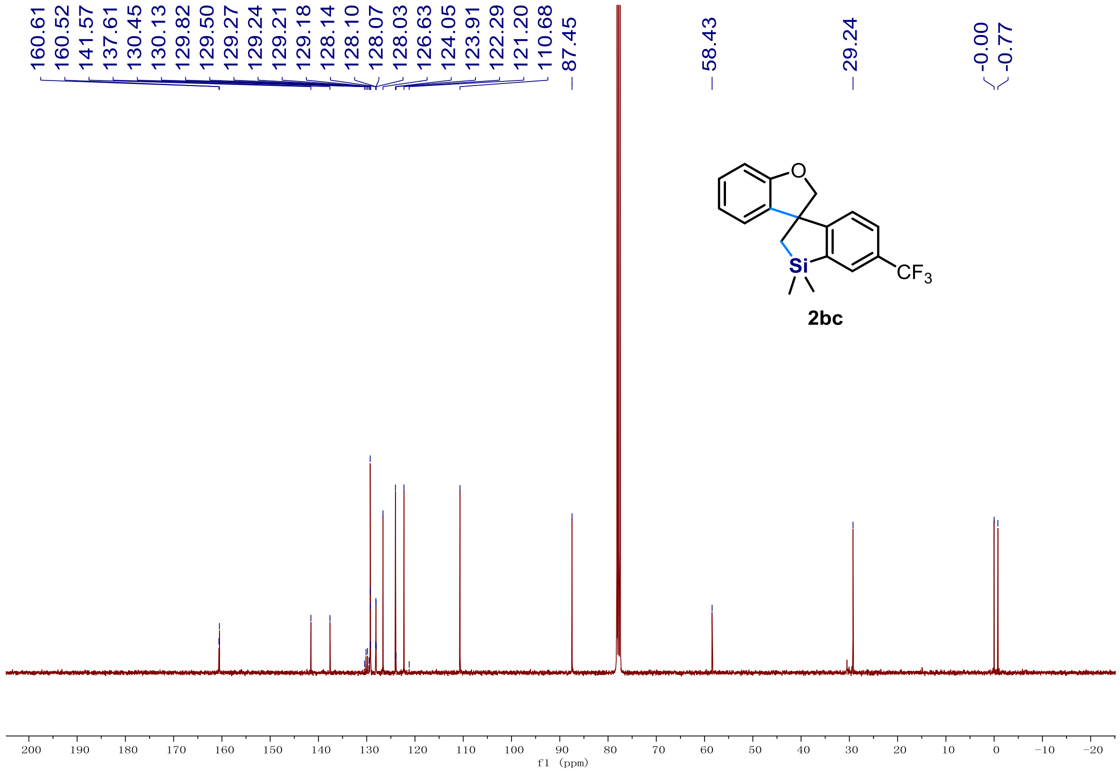

Supplementary Figure 139. ^{13}C **NMR (101 MHz,** $CDCl_3$**)** spectra of **2bc**

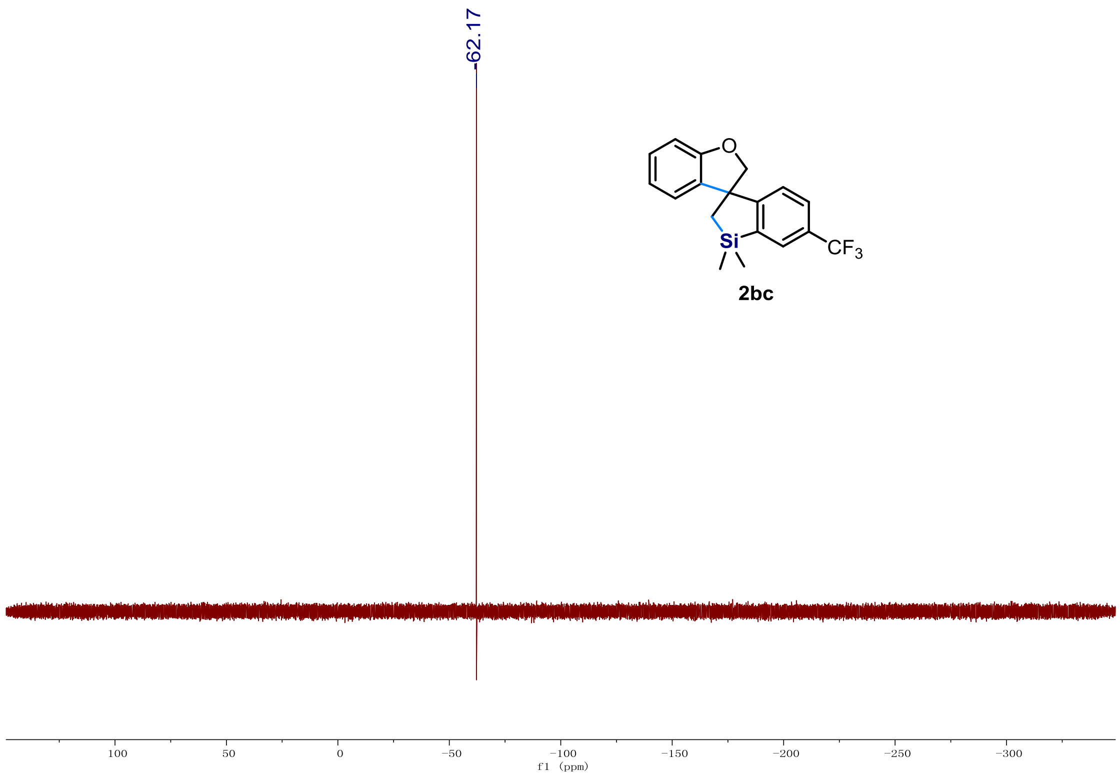

Supplementary Figure 140. ^{19}F NMR (376 MHz, CDCl$_3$) spectra of **2bc**

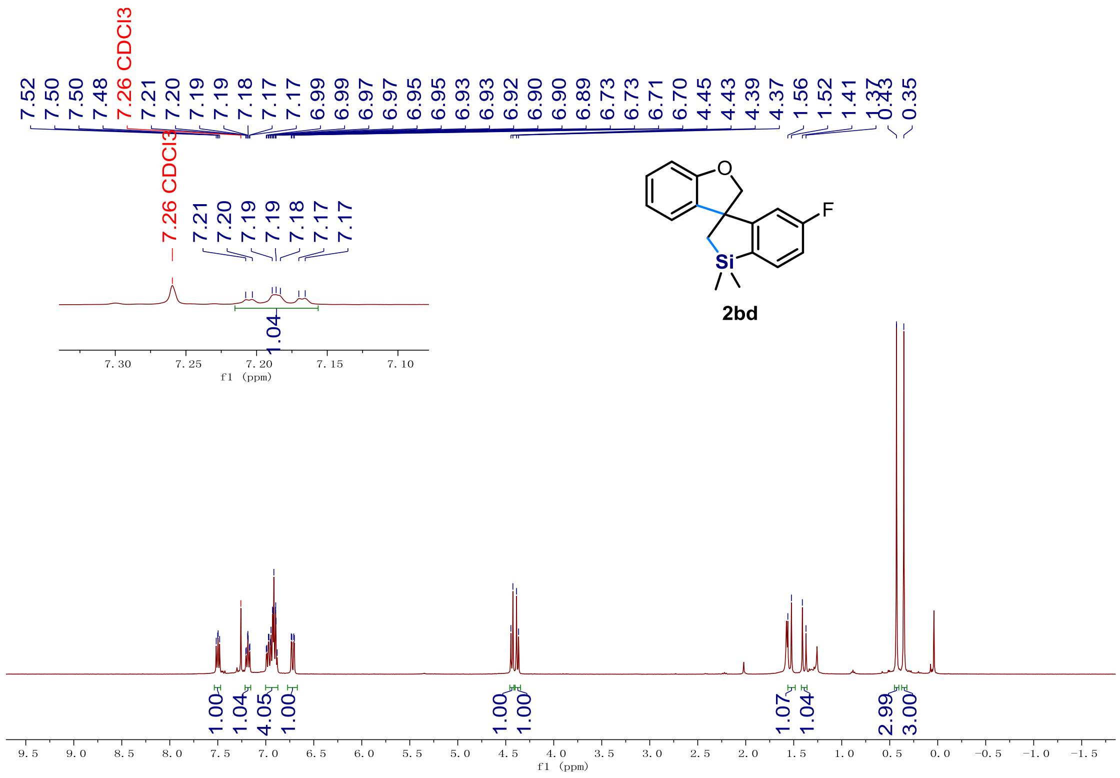

Supplementary Figure 141. ^{1}**H NMR (400 MHz, CDCl$_3$)** spectra of **2bd**

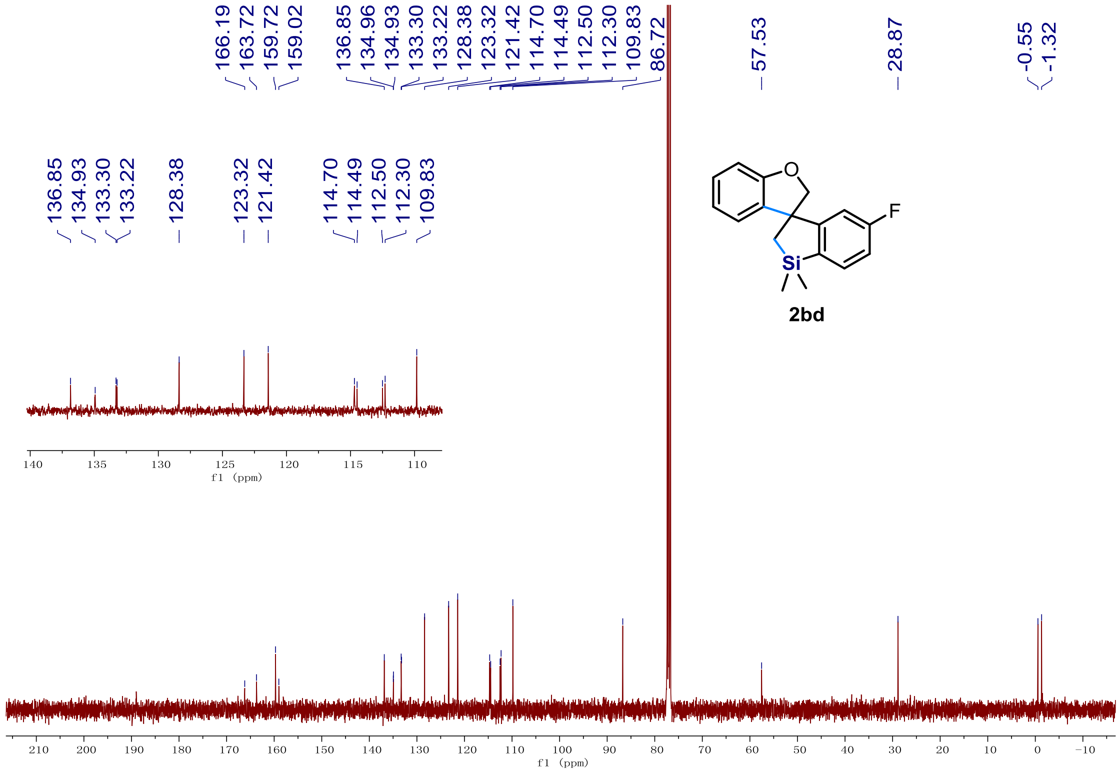

Supplementary Figure 142. ^{13}**C NMR (101 MHz, CDCl$_3$)** spectra of **2bd**

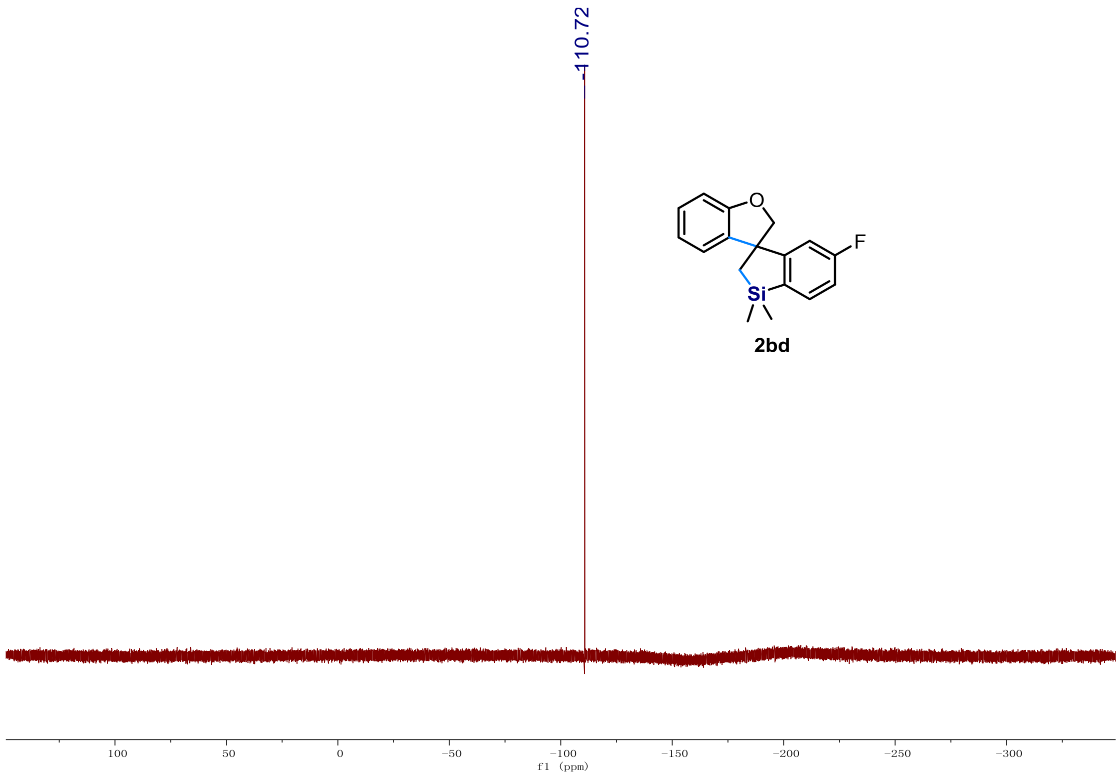

Supplementary Figure 143. ^{19}**F NMR (376 MHz, CDCl$_3$)** spectra of **2bd**

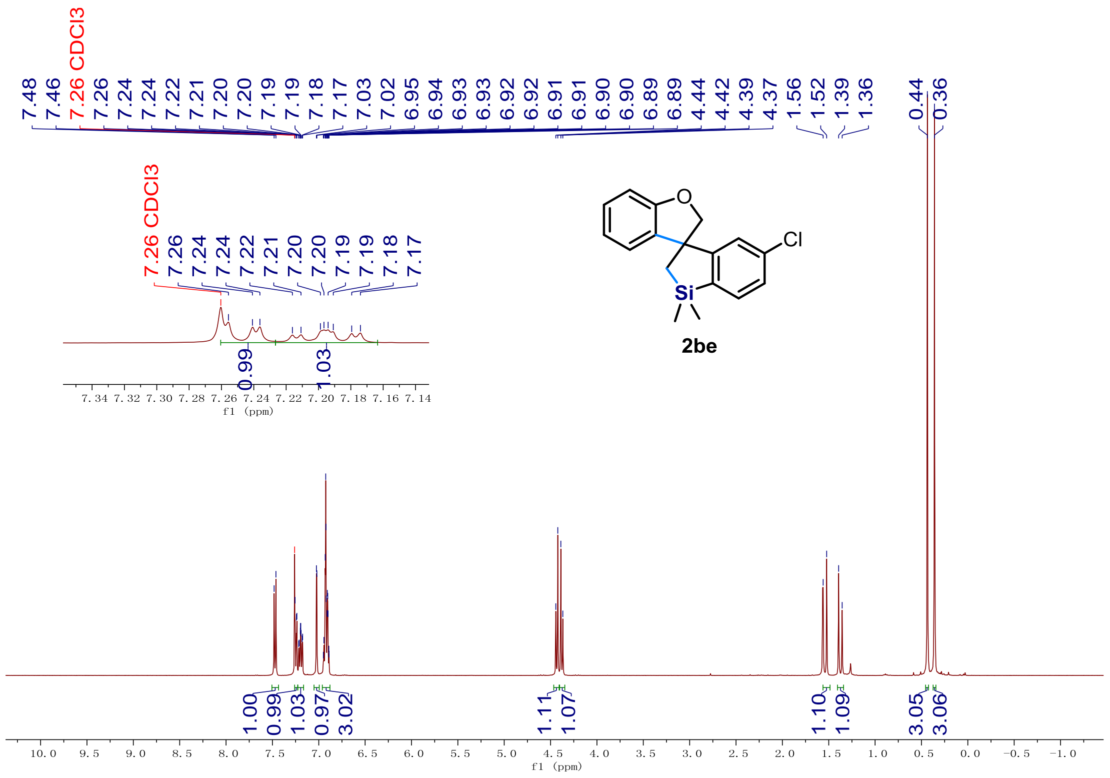

Supplementary Figure 144. **^{1}H NMR (400 MHz, CDCl$_3$)** spectra of **2be**

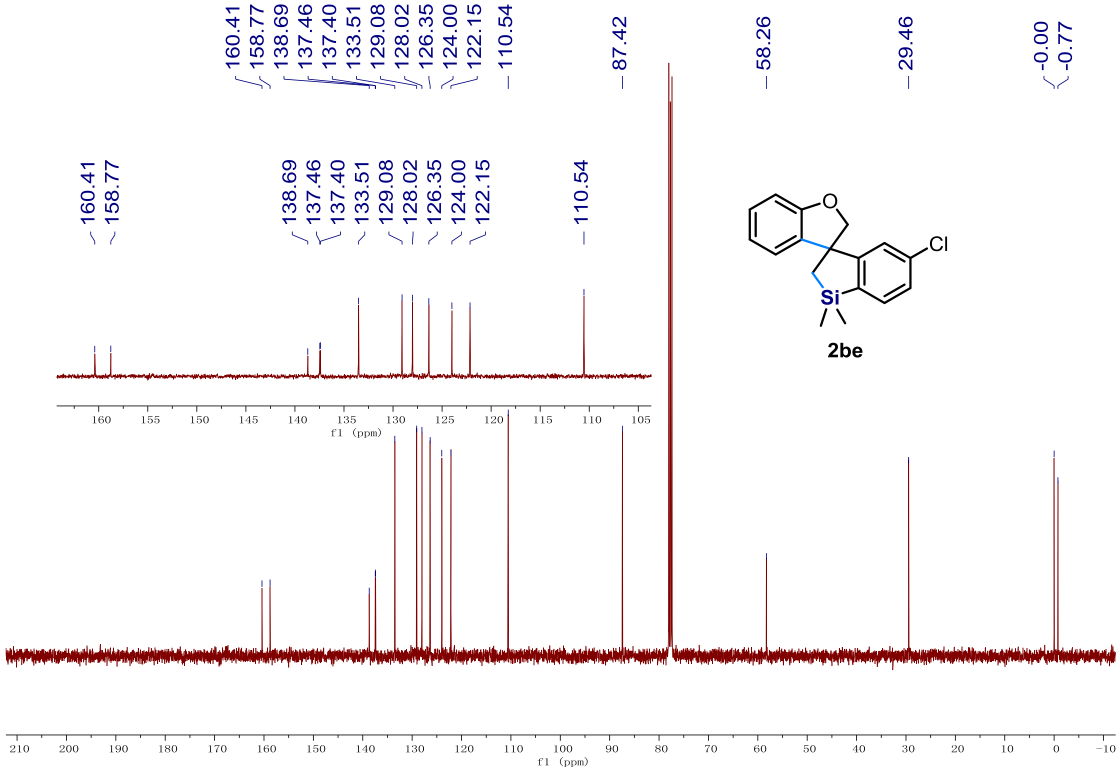

Supplementary Figure 145. **^{13}C NMR (101 MHz, CDCl$_3$)** spectra of **2be**

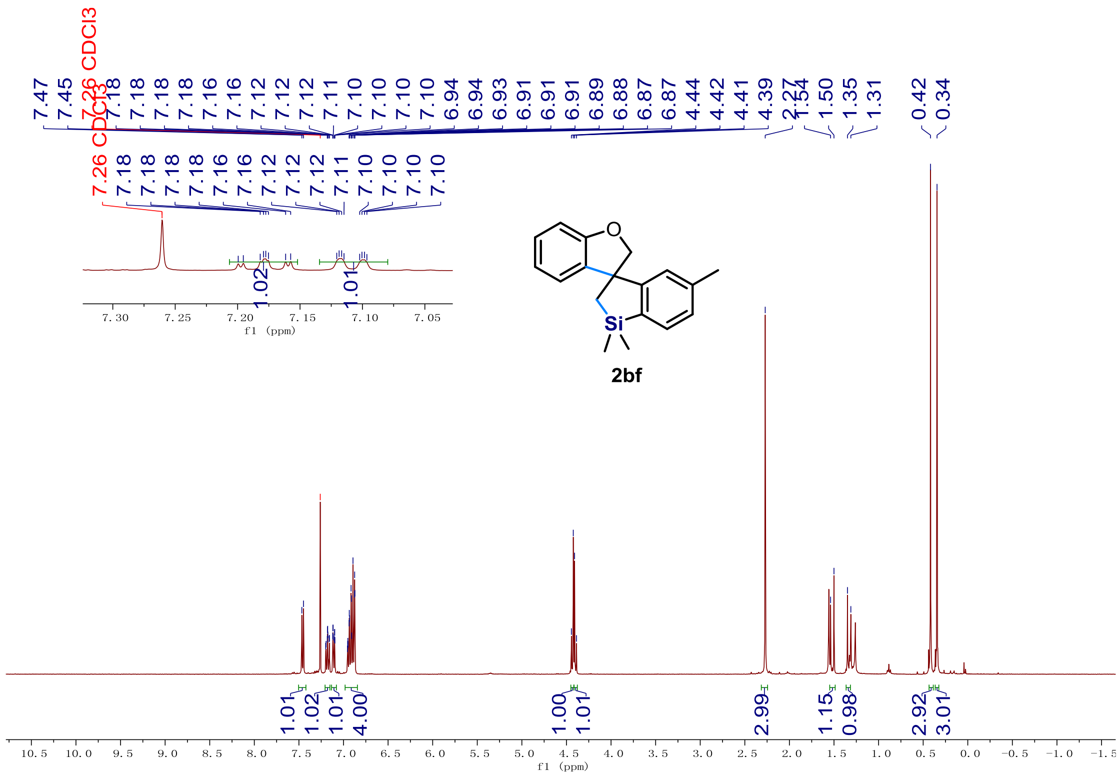

Supplementary Figure 146. **^{1}H NMR (400 MHz, CDCl$_3$)** spectra of **2bf**

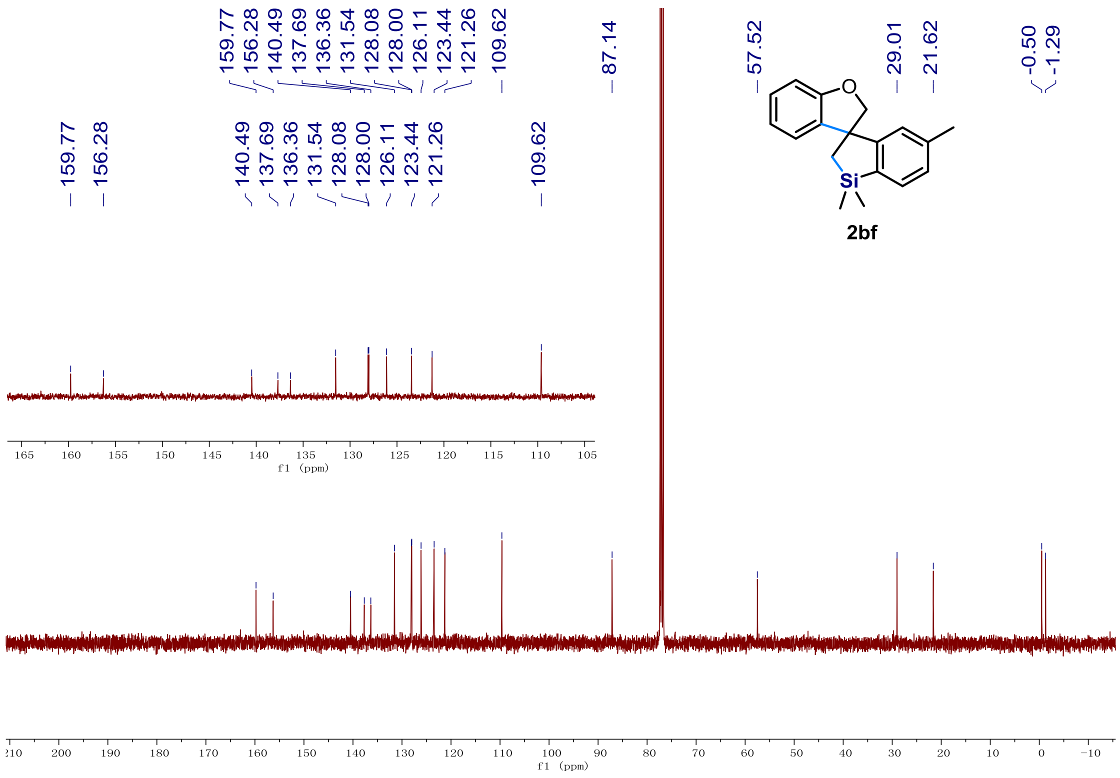

Supplementary Figure 147. **^{13}C NMR (101 MHz, CDCl$_3$)** spectra of **2bf**

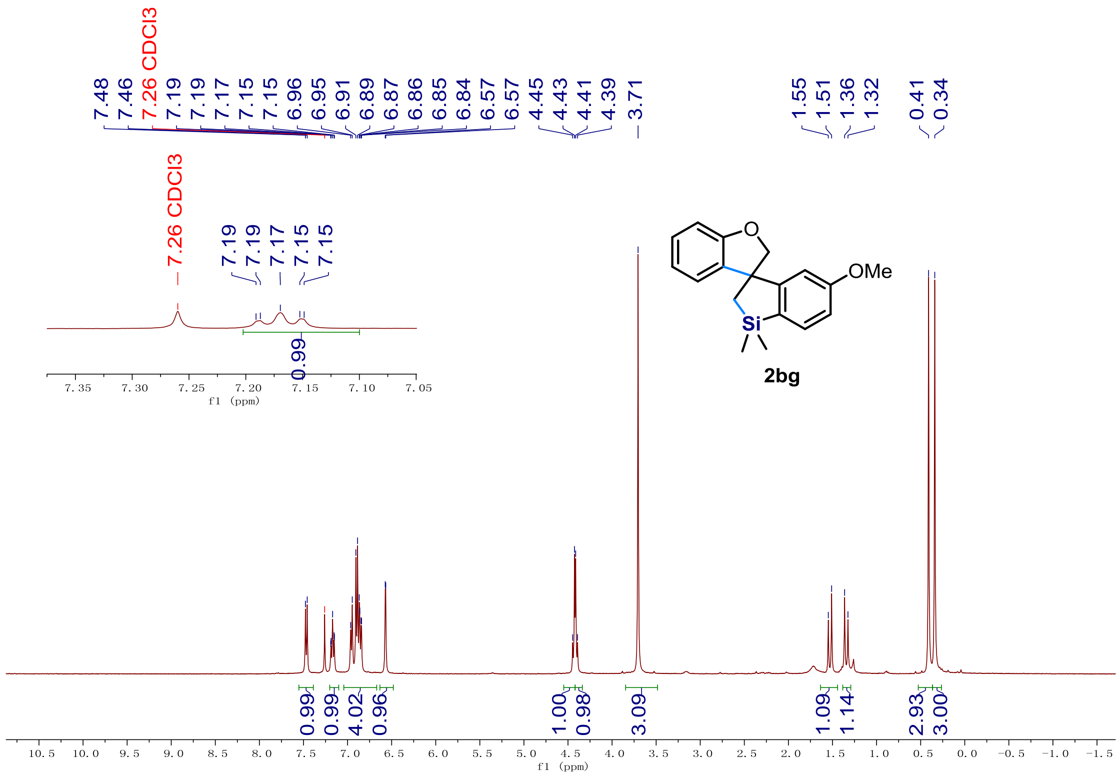

Supplementary Figure 148. ^{1}**H NMR (400 MHz, CDCl$_3$)** spectra of **2bg**

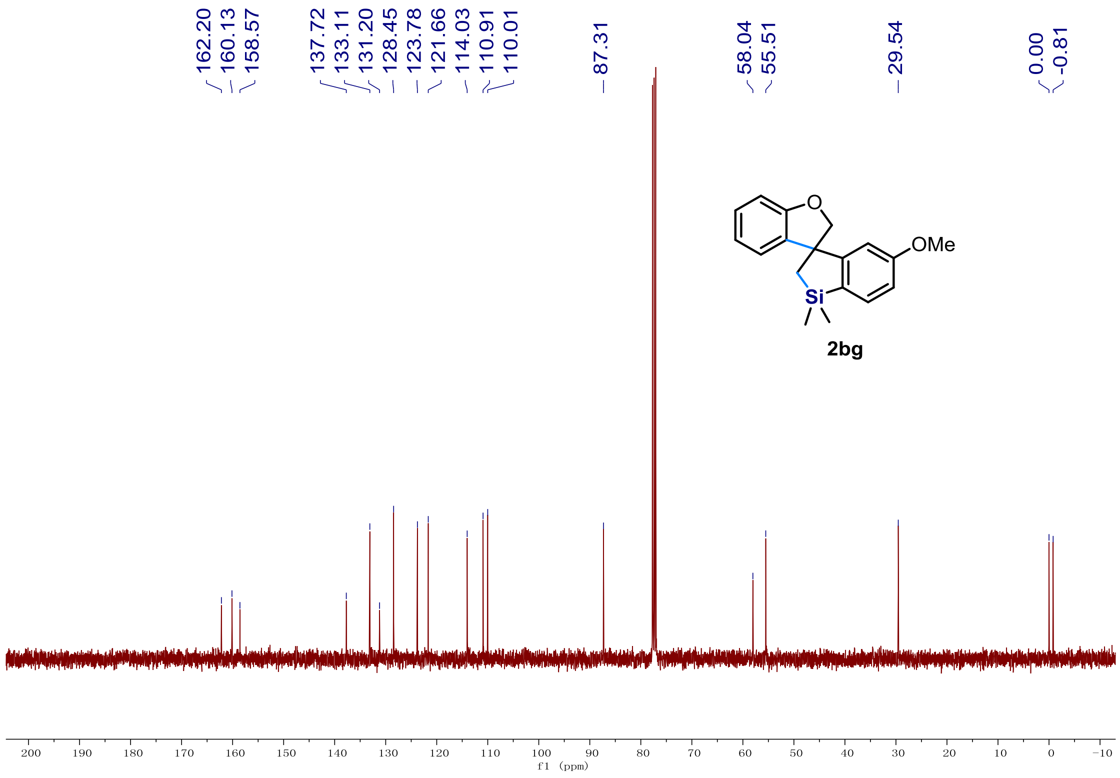

Supplementary Figure 149. ^{13}**C NMR (101 MHz, CDCl$_3$)** spectra of **2bg**

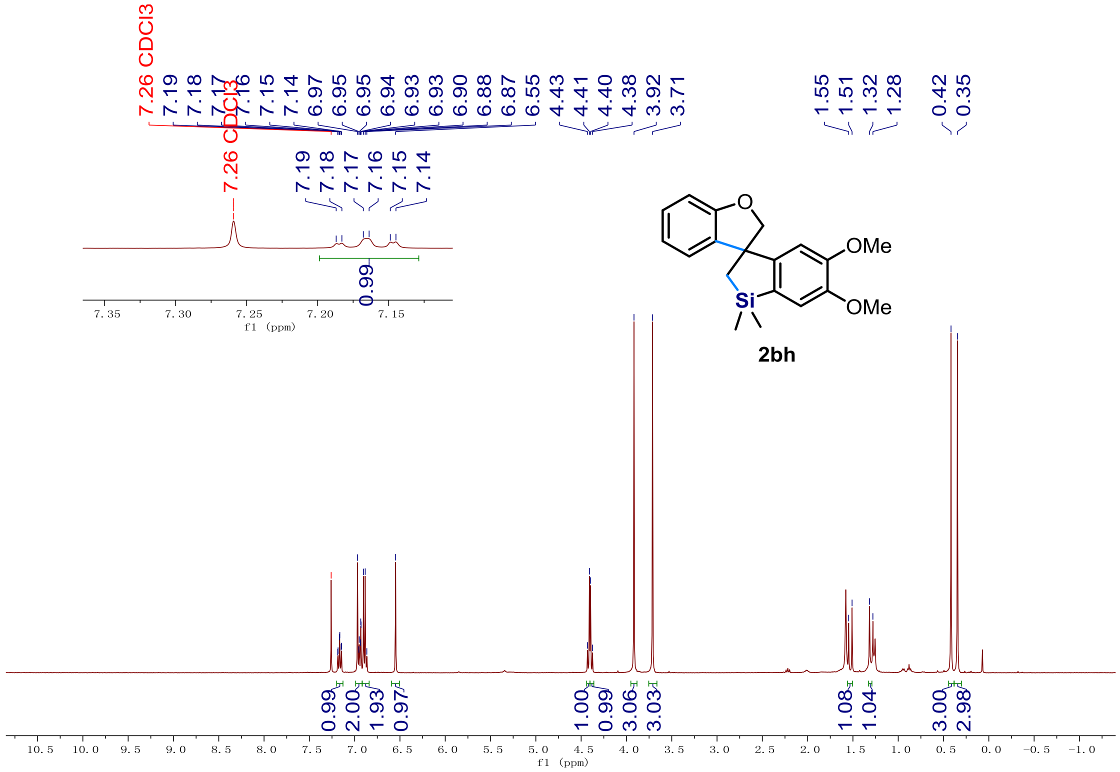

Supplementary Figure 150. ^{1}**H NMR (400 MHz, CDCl**$_3$**)** spectra of **2bh**

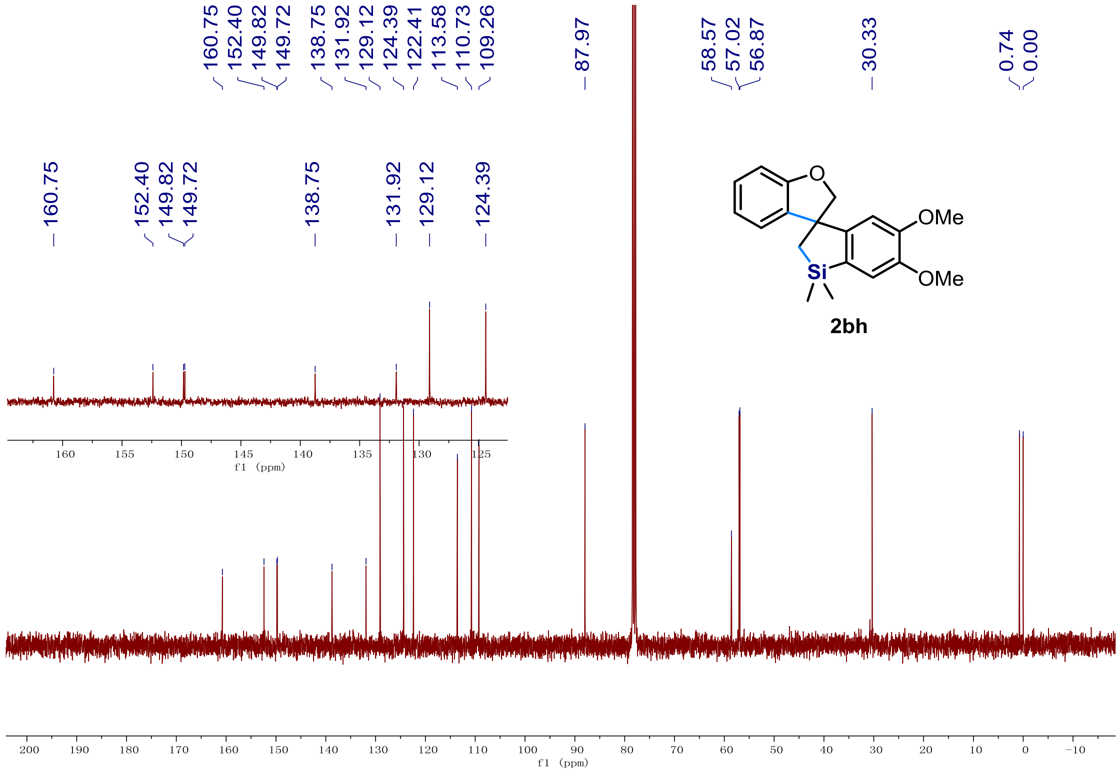

Supplementary Figure 151. ^{13}**C NMR (101 MHz, CDCl**$_3$**)** spectra of **2bh**

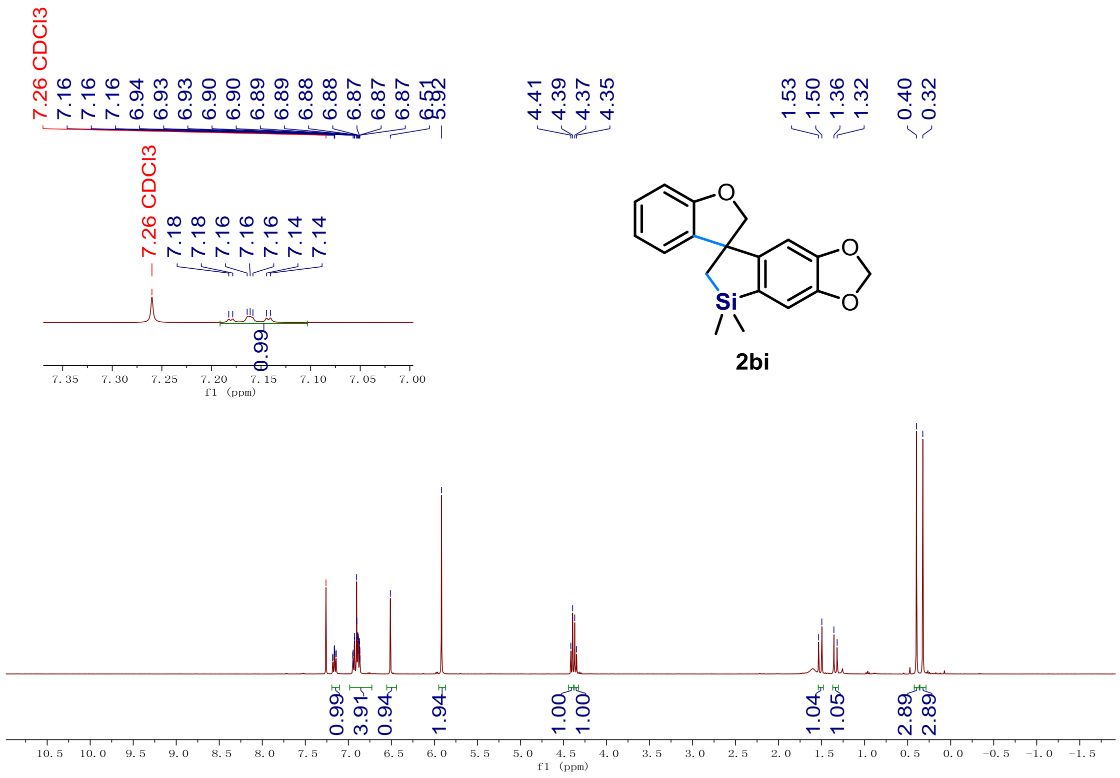

Supplementary Figure 152. ^{1}**H NMR (400 MHz, CDCl$_3$)** spectra of **2bi**

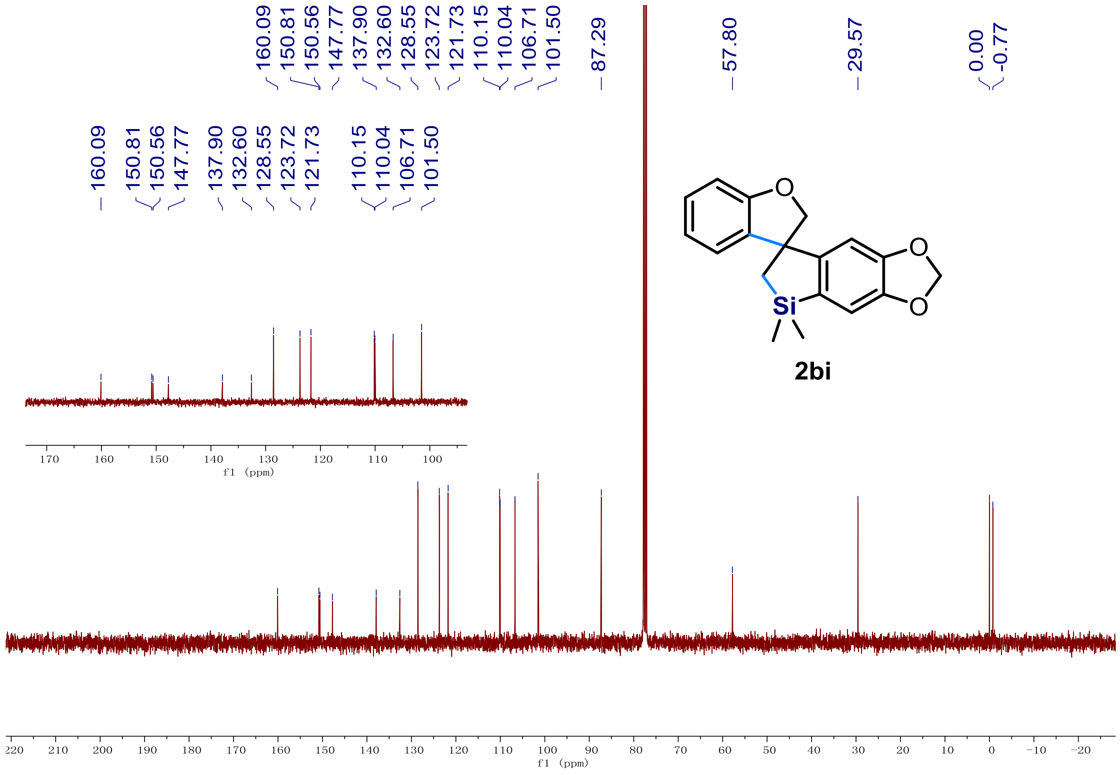

Supplementary Figure 153. ^{13}**C NMR (101 MHz, CDCl$_3$)** spectra of **2bi**

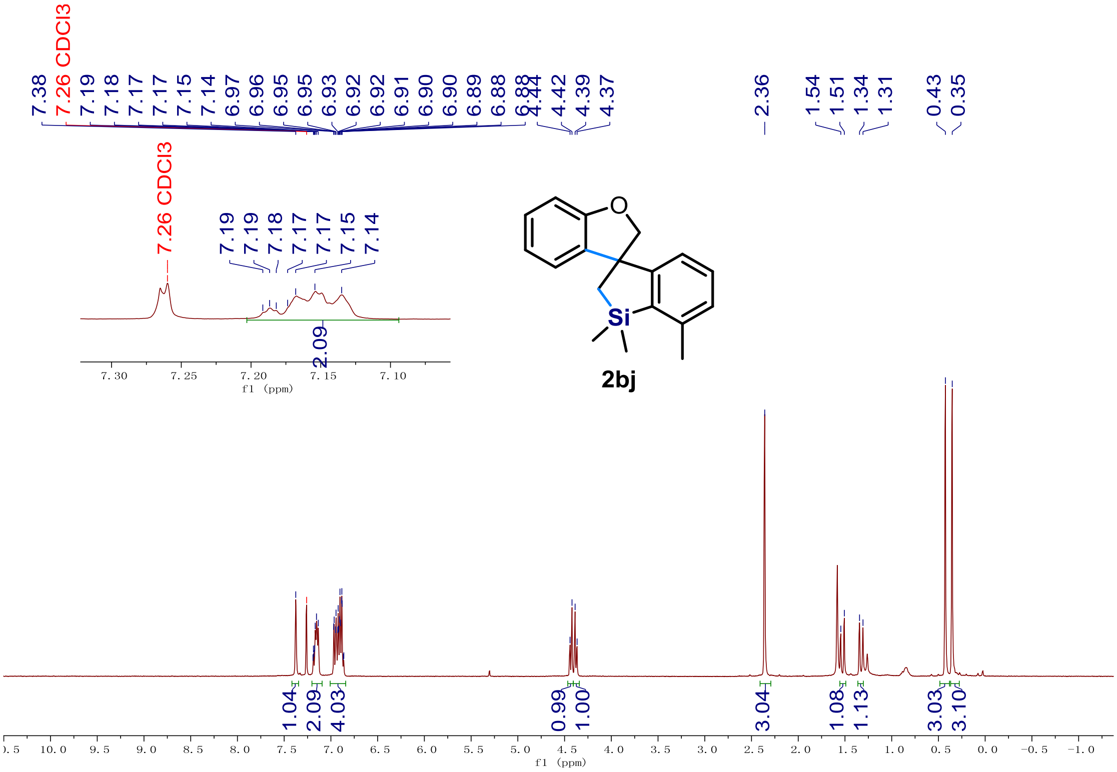

Supplementary Figure 154. ^{1}H NMR (400 MHz, $CDCl_3$) spectra of **2bj**

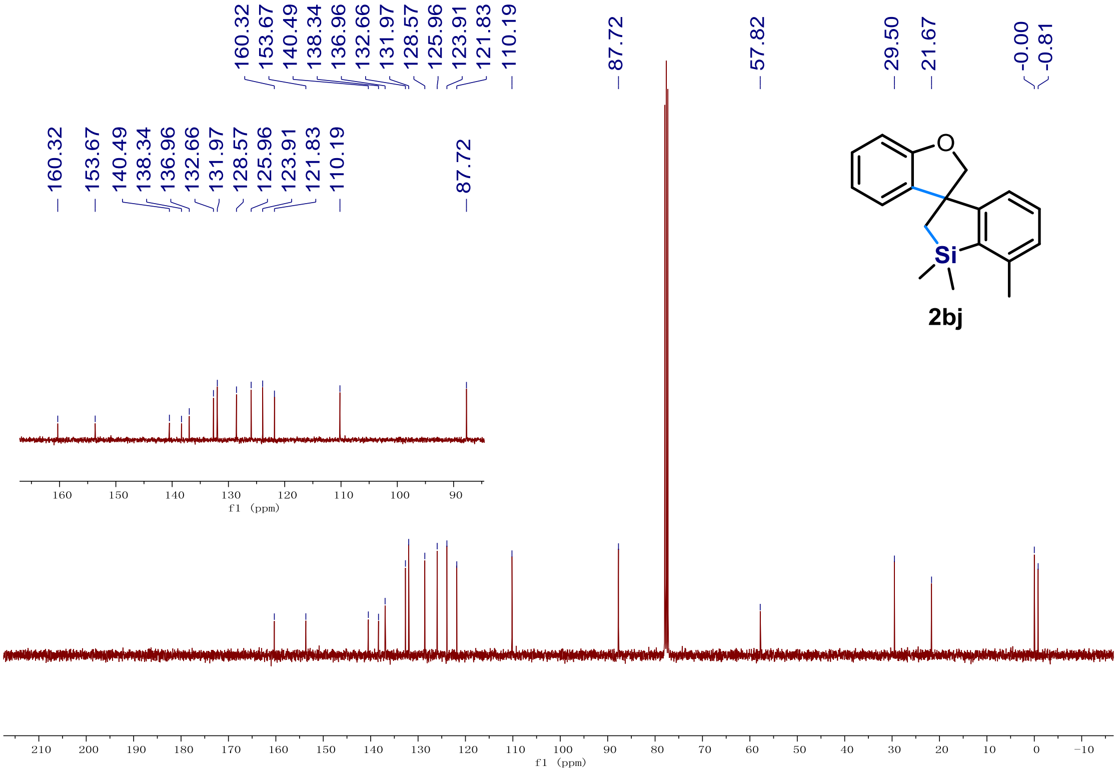

Supplementary Figure 155. ^{13}C NMR (101 MHz, $CDCl_3$) spectra of **2bj**

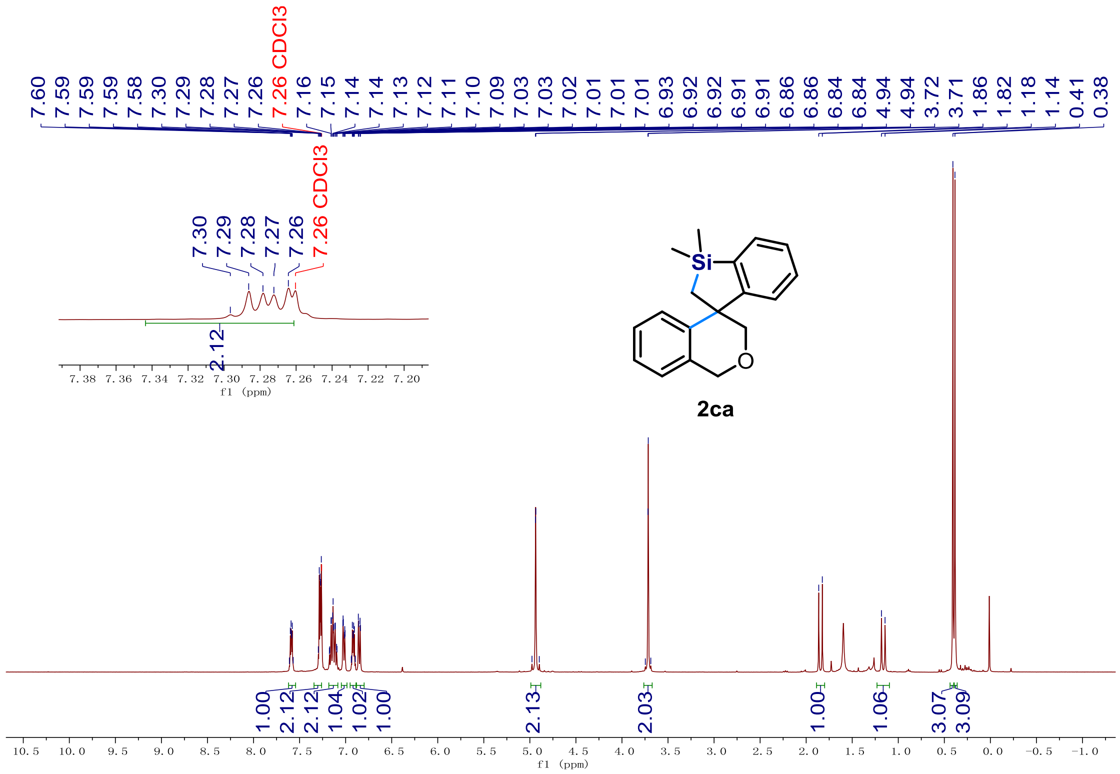

Supplementary Figure 156. ^{1}**H NMR (400 MHz, CDCl$_3$)** spectra of **2ca**

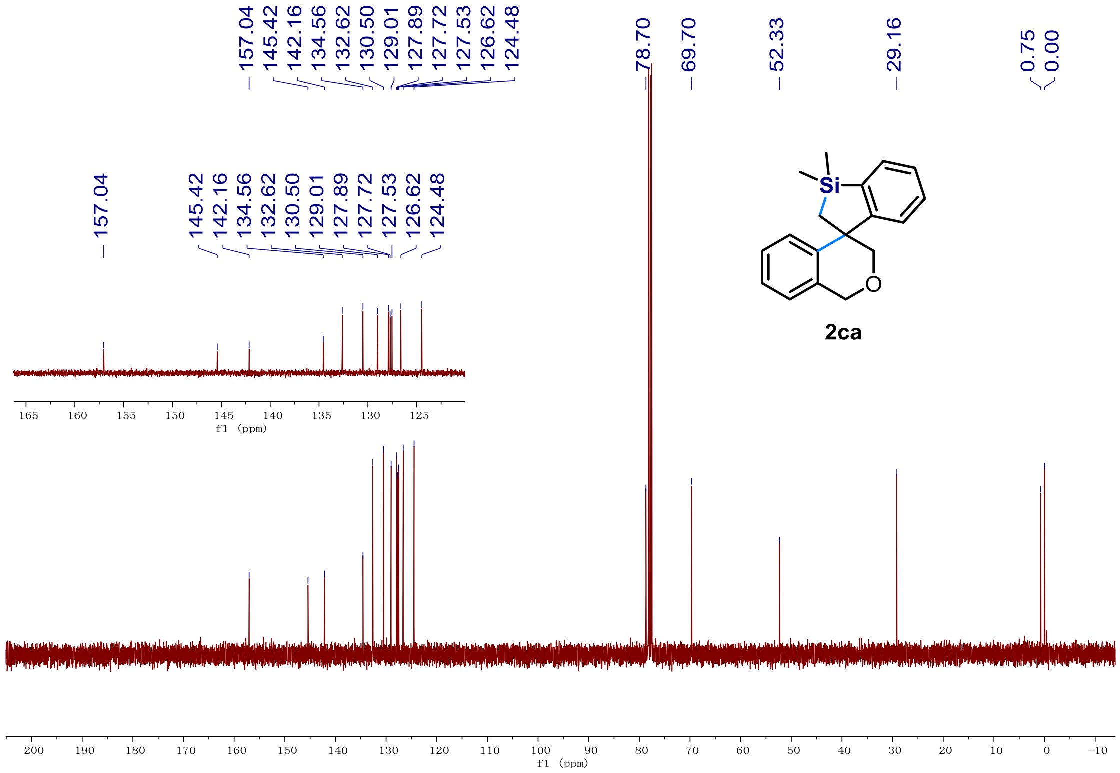

Supplementary Figure 157. ^{13}**C NMR (101 MHz, CDCl$_3$)** spectra of **2ca**

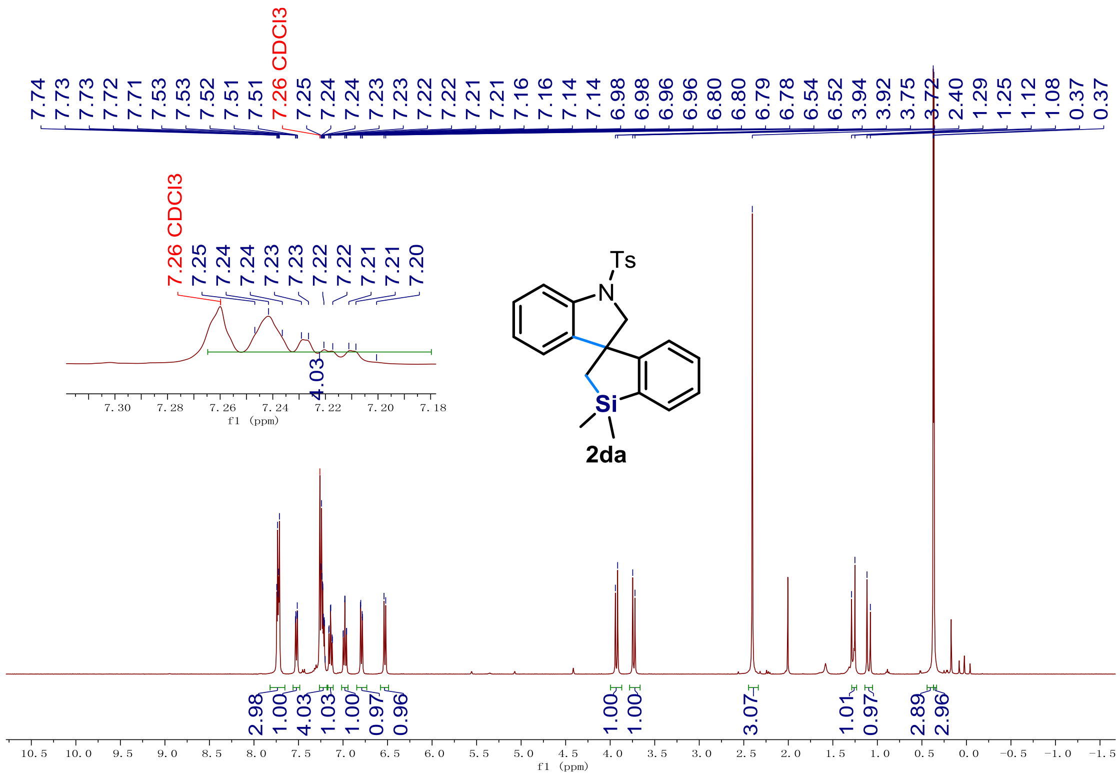

Supplementary Figure 158. **^{1}H NMR (400 MHz, $CDCl_3$)** spectra of **2da**

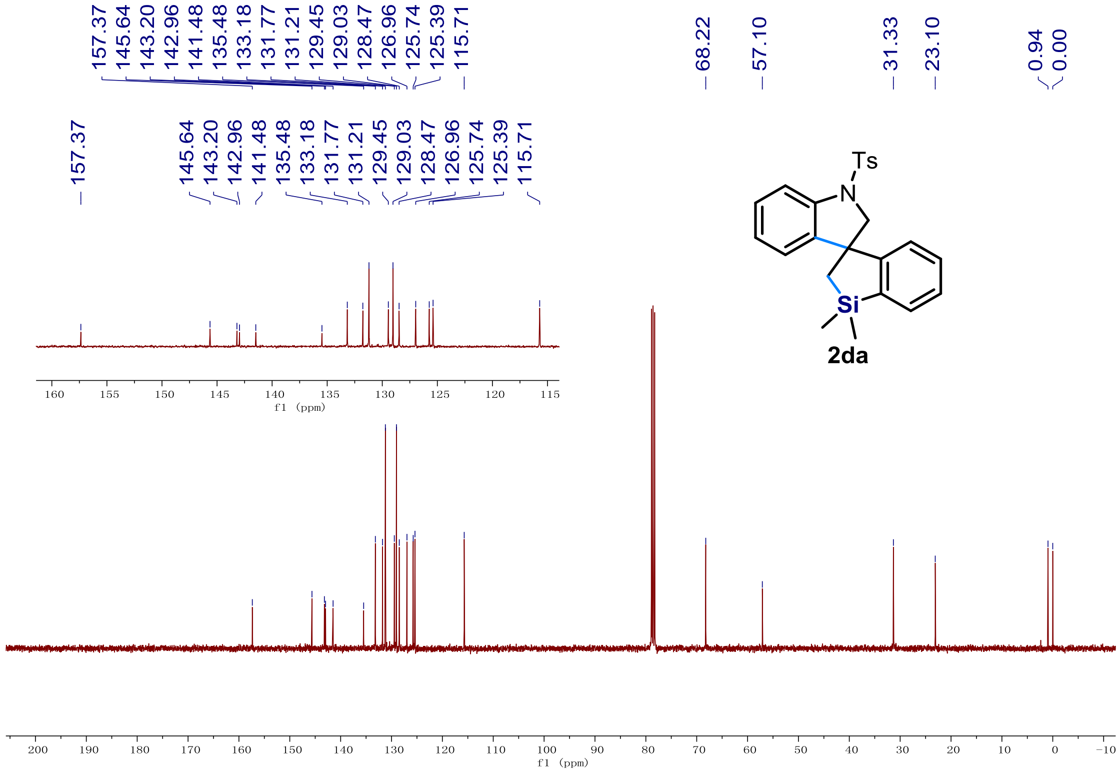

Supplementary Figure 159. **^{13}C NMR (101 MHz, $CDCl_3$)** spectra of **2da**

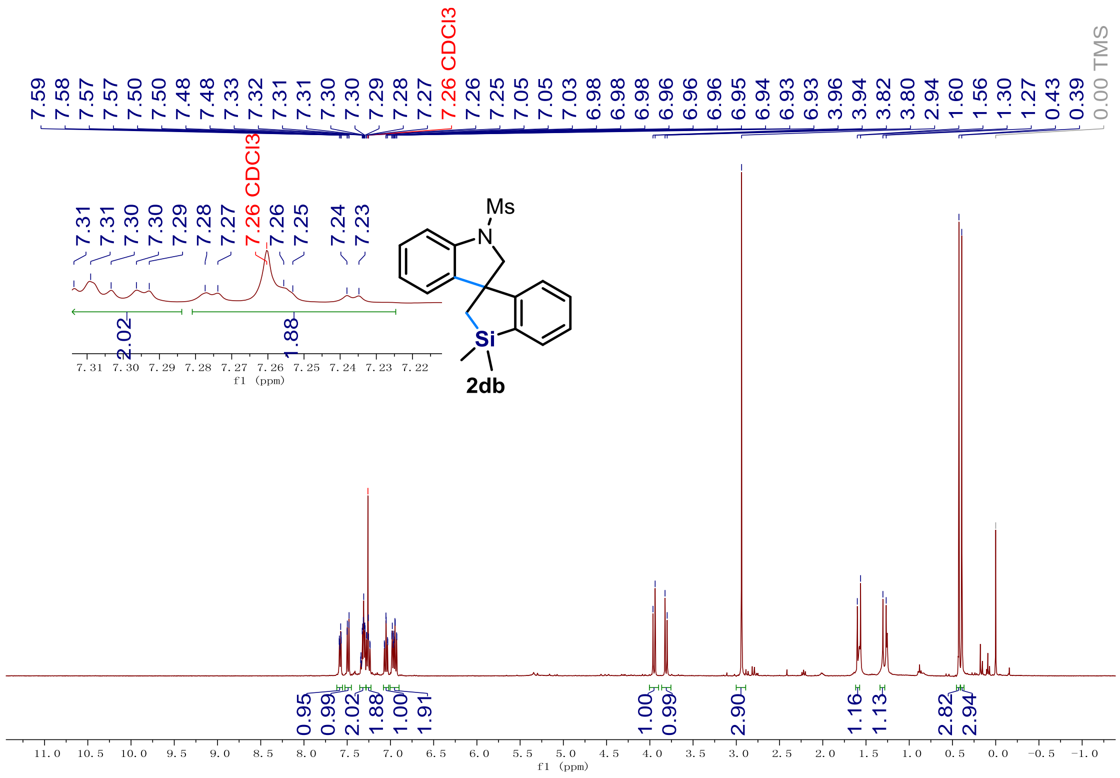

Supplementary Figure 160. **^{1}H NMR (400 MHz, CDCl$_3$)** spectra of **2db**

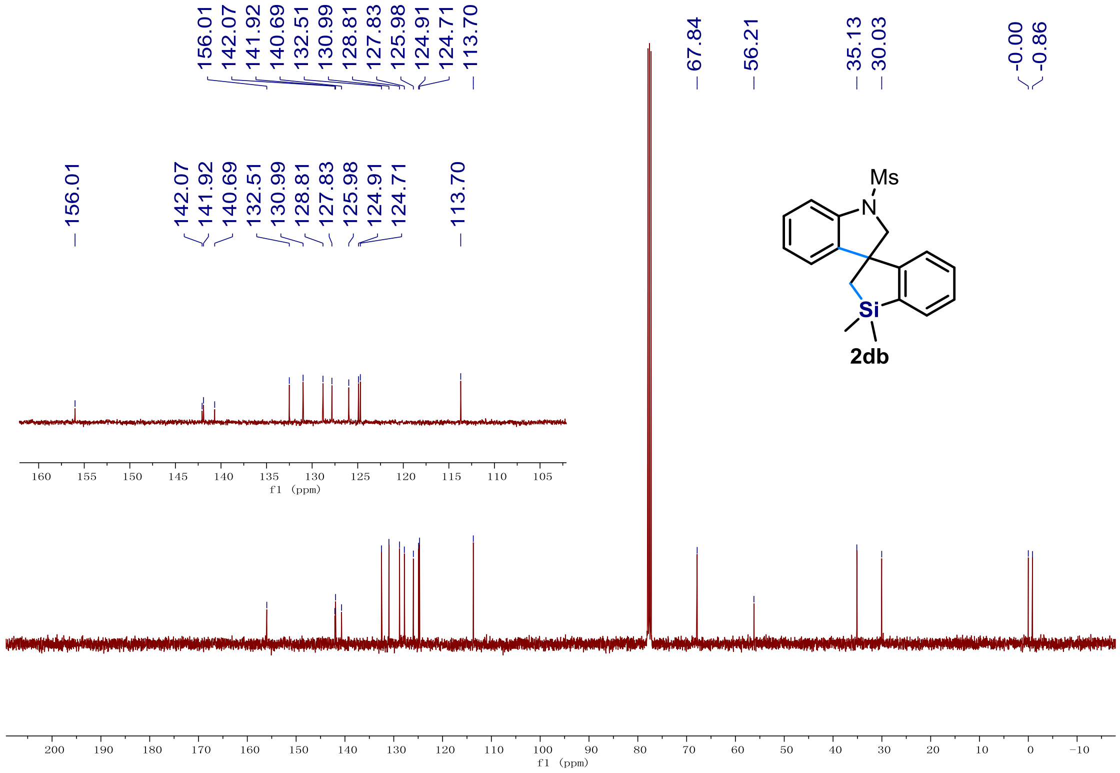

Supplementary Figure 161. **^{13}C NMR (101 MHz, CDCl$_3$)** spectra of **2db**

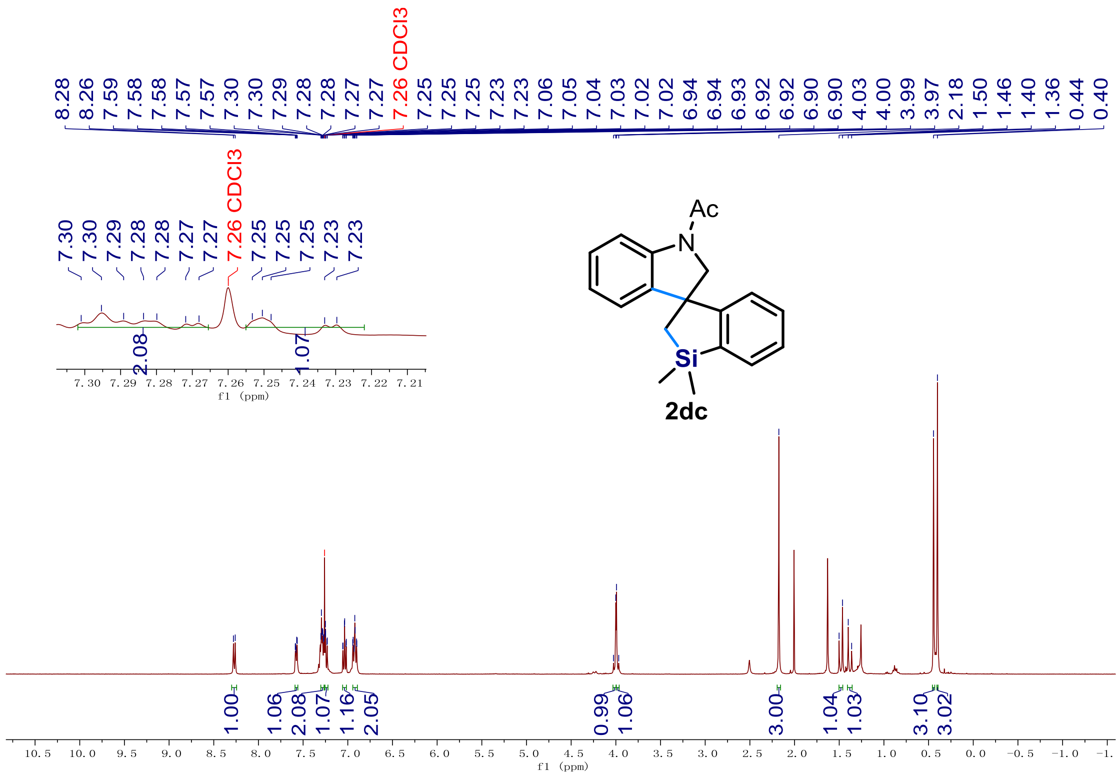

Supplementary Figure 162. **^{1}H NMR (400 MHz, $CDCl_3$)** spectra of **2dc**

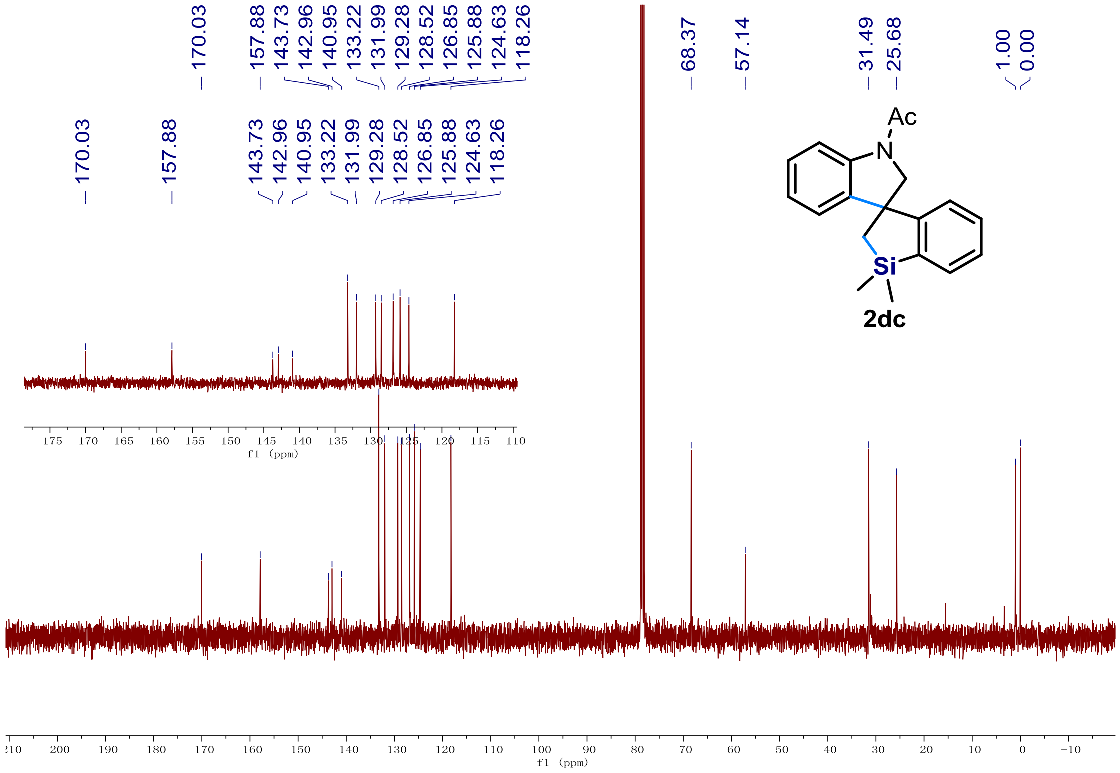

Supplementary Figure 163. **^{13}C NMR (101 MHz, $CDCl_3$)** spectra of **2dc**

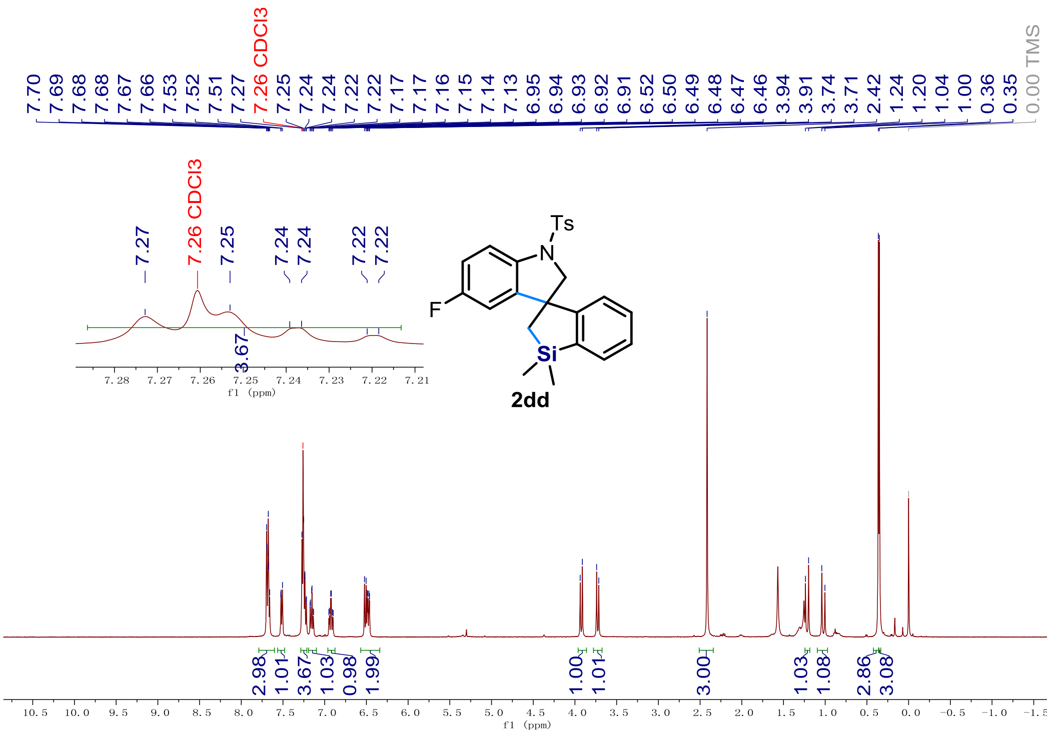

Supplementary Figure 164. ^{1}**H NMR (400 MHz, CDCl$_3$)** spectra of **2dd**

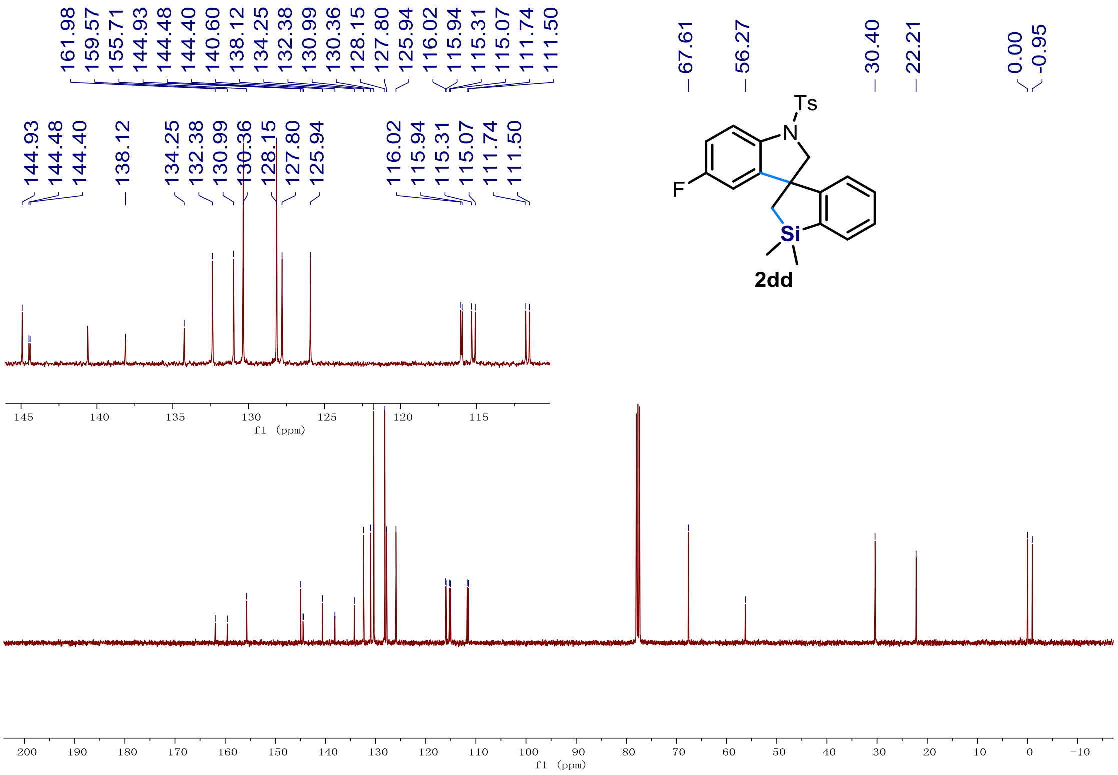

Supplementary Figure 165. ^{13}**C NMR (101 MHz, CDCl$_3$)** spectra of **2dd**

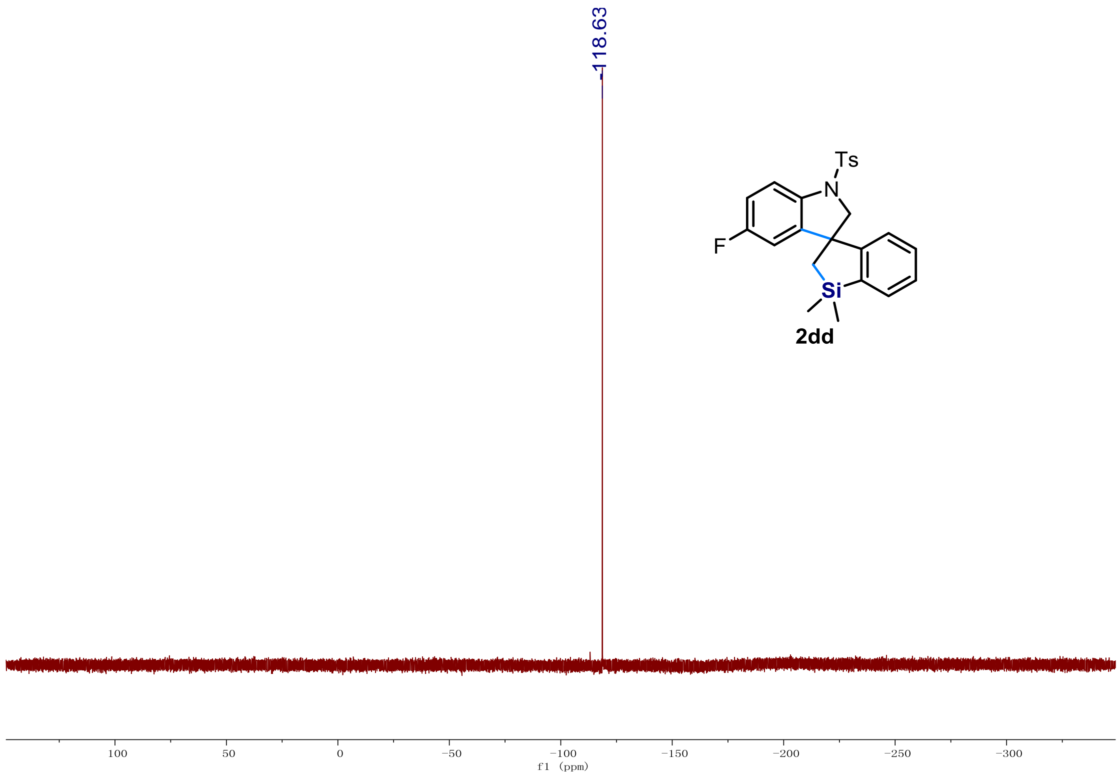

Supplementary Figure 166. **^{19}F NMR (376 MHz, $CDCl_3$)** spectra of **2dd**

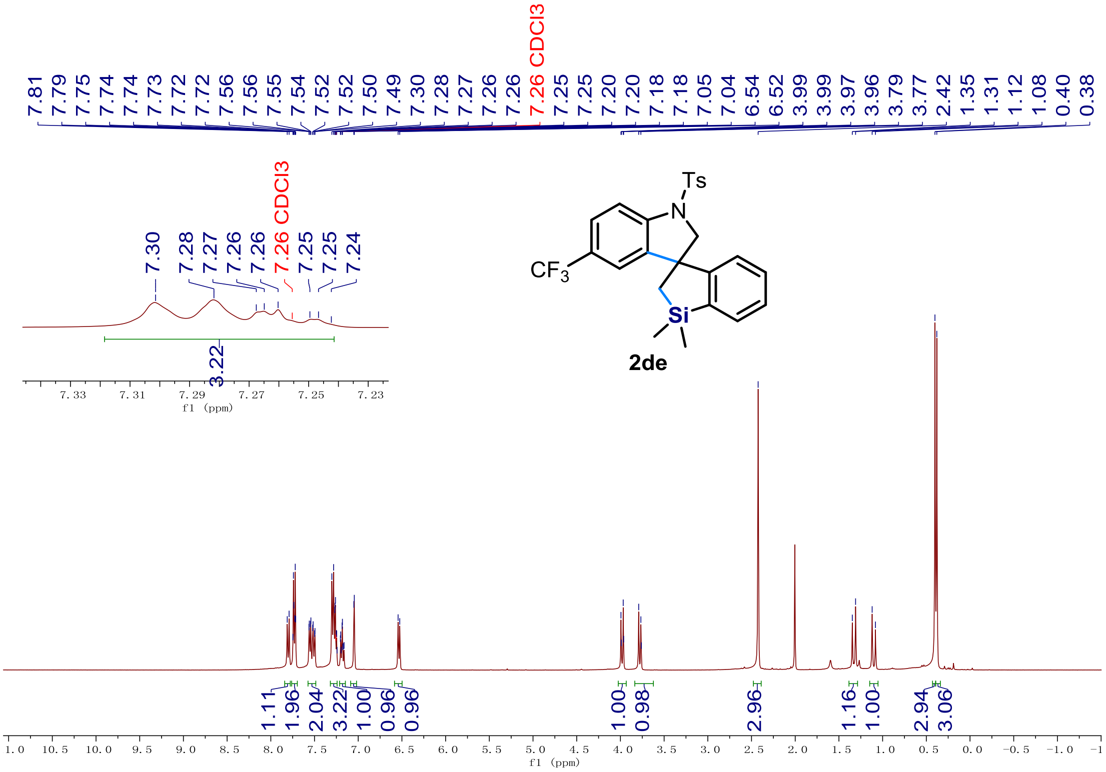

Supplementary Figure 167. **^{1}H NMR (400 MHz, $CDCl_3$)** spectra of **2de**

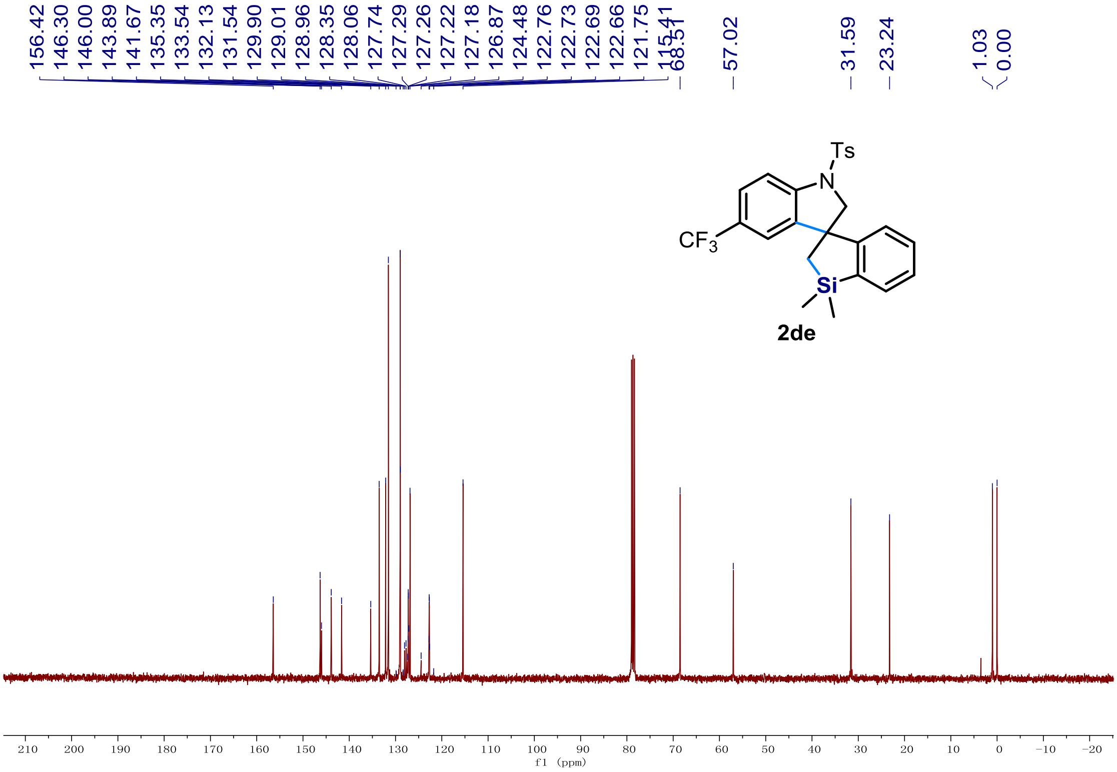

Supplementary Figure 168. **^{13}C NMR (101 MHz, $CDCl_3$)** spectra of **2de**

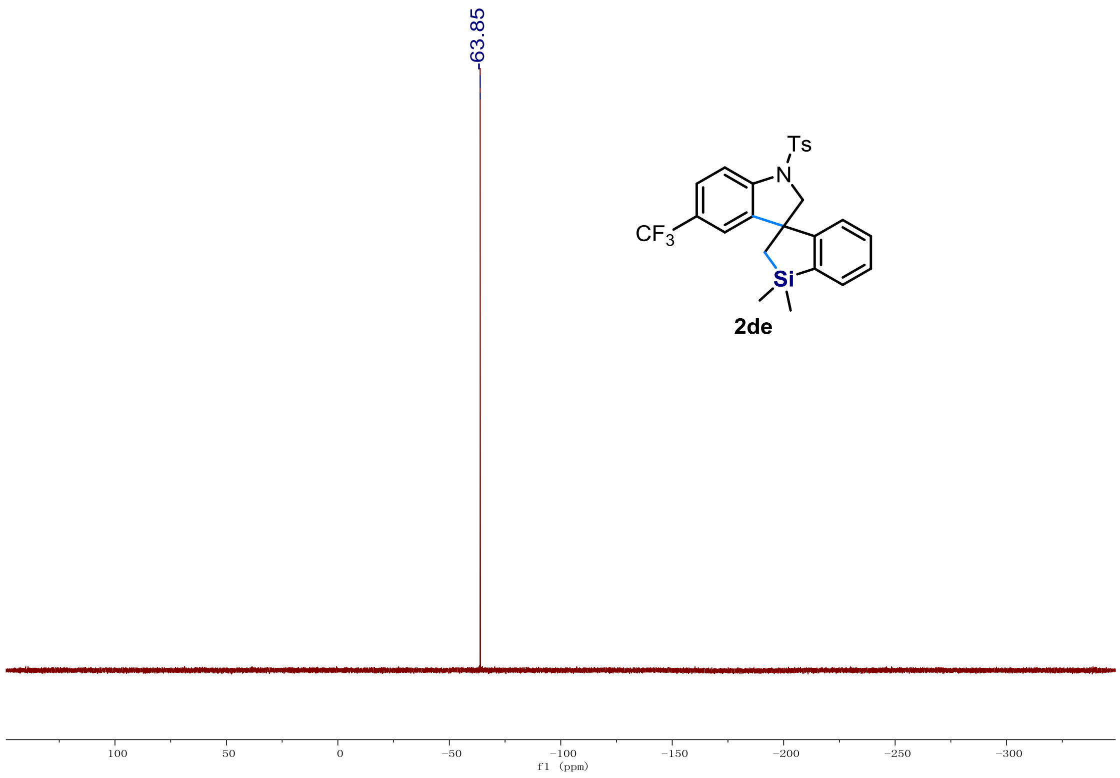

Supplementary Figure 169. ^{19}F NMR (376 MHz, CDCl$_3$) spectra of **2de**

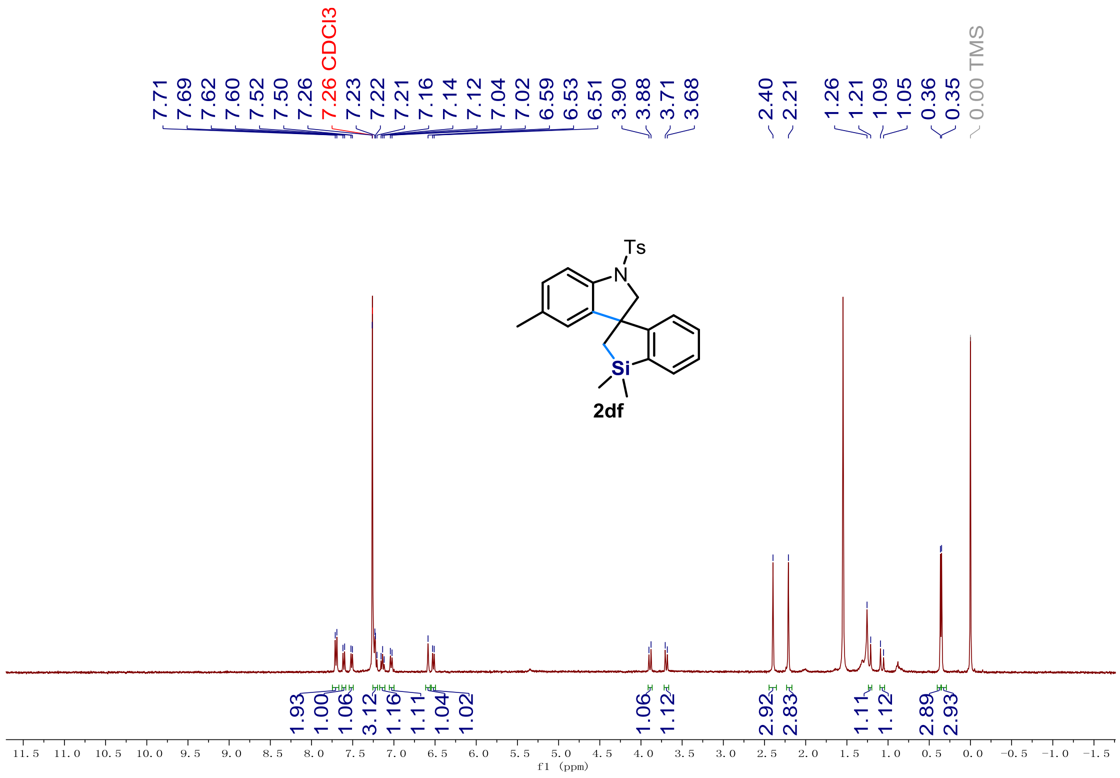

Supplementary Figure 170. **^{1}H NMR (400 MHz, $CDCl_3$)** spectra of **2df**

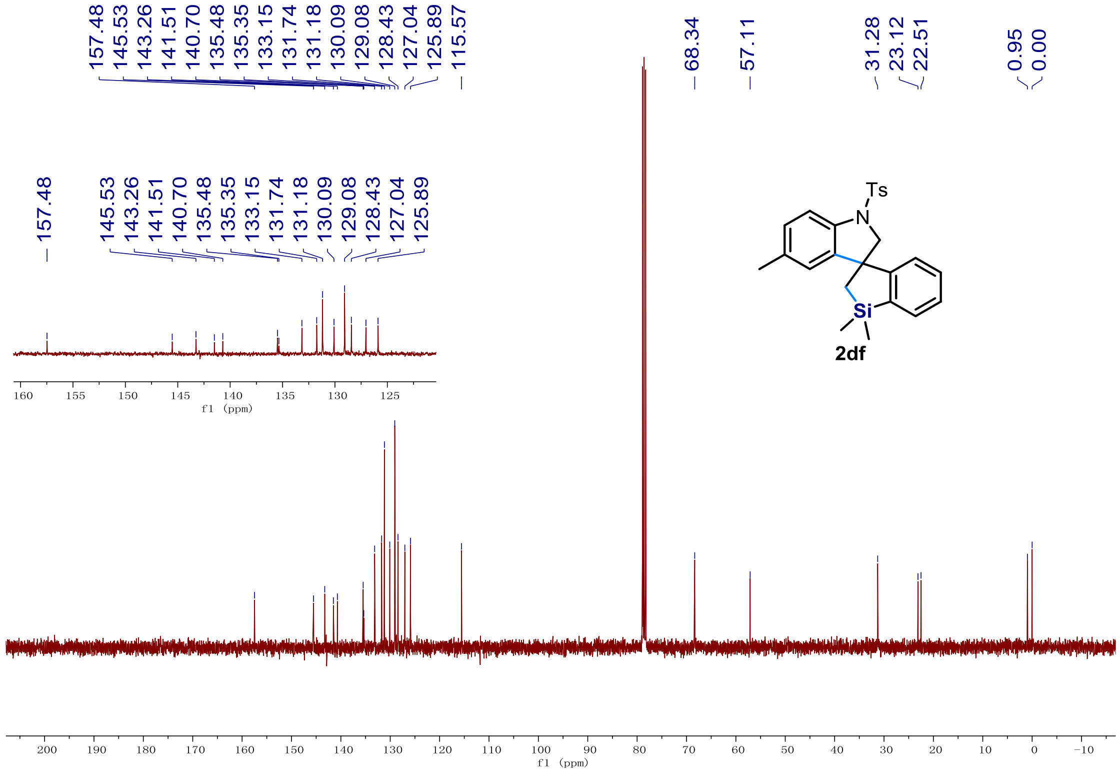

Supplementary Figure 171. **^{13}C NMR (101 MHz, $CDCl_3$)**spectra of **2df**

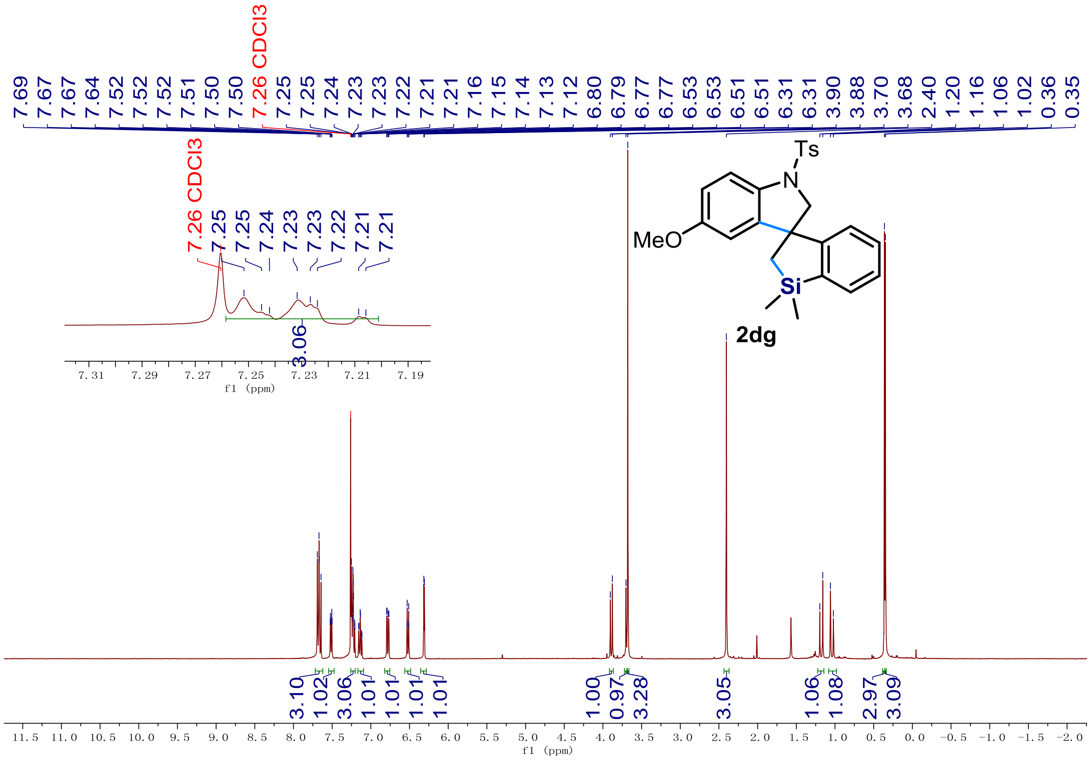

Supplementary Figure 172. **^{1}H NMR (400 MHz, CDCl$_3$)** spectra of **2dg**

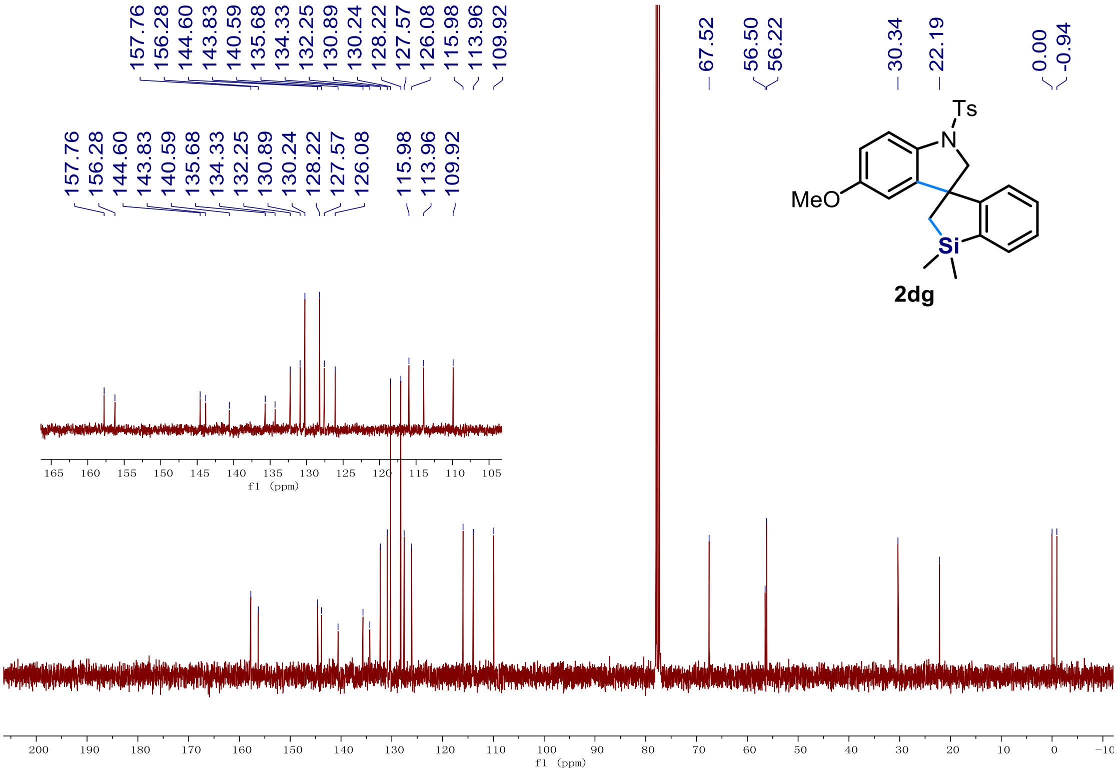

Supplementary Figure 173. **^{13}C NMR (101 MHz, CDCl$_3$)** spectra of **2dg**

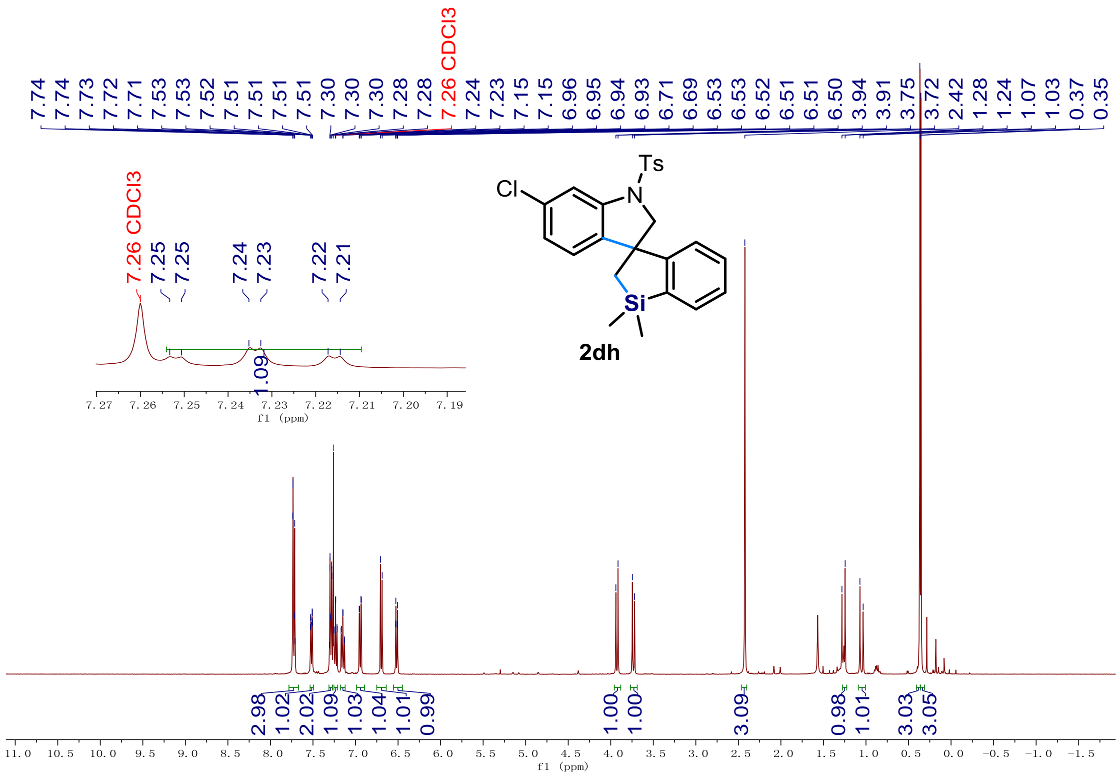

Supplementary Figure 174. ^{1}**H NMR (400 MHz, CDCl$_3$)** spectra of **2dh**

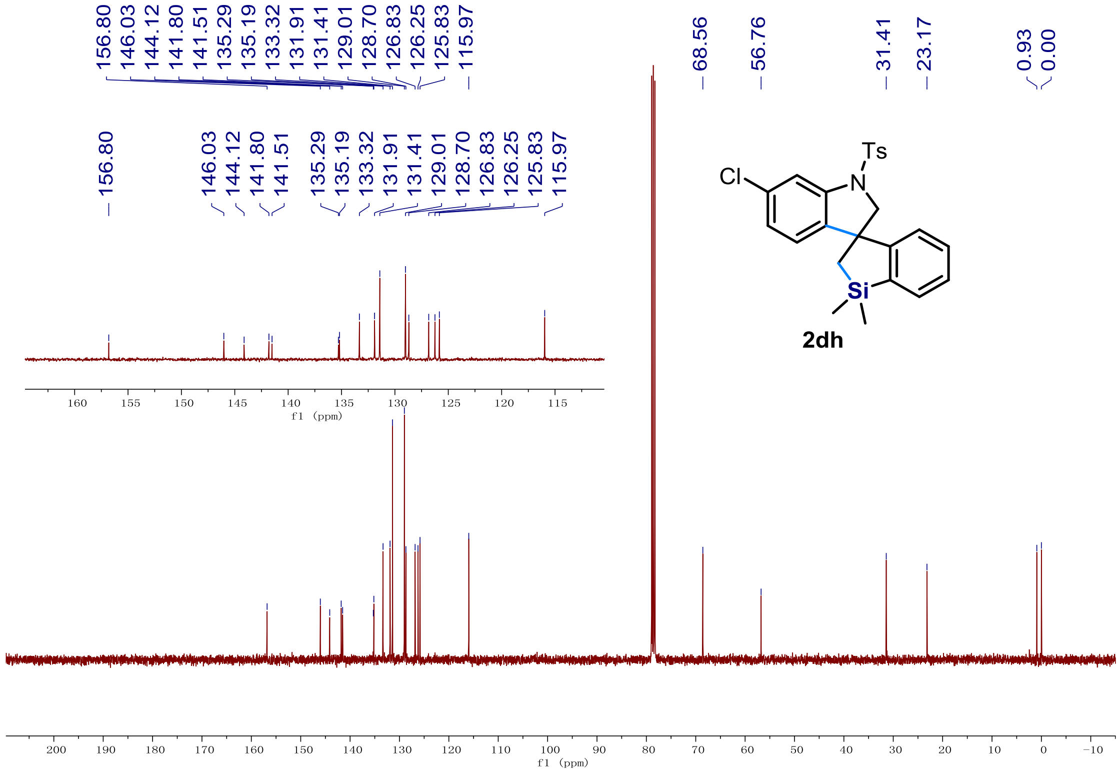

Supplementary Figure 175. ^{13}**C NMR (101 MHz, CDCl$_3$)** spectra of **2dh**

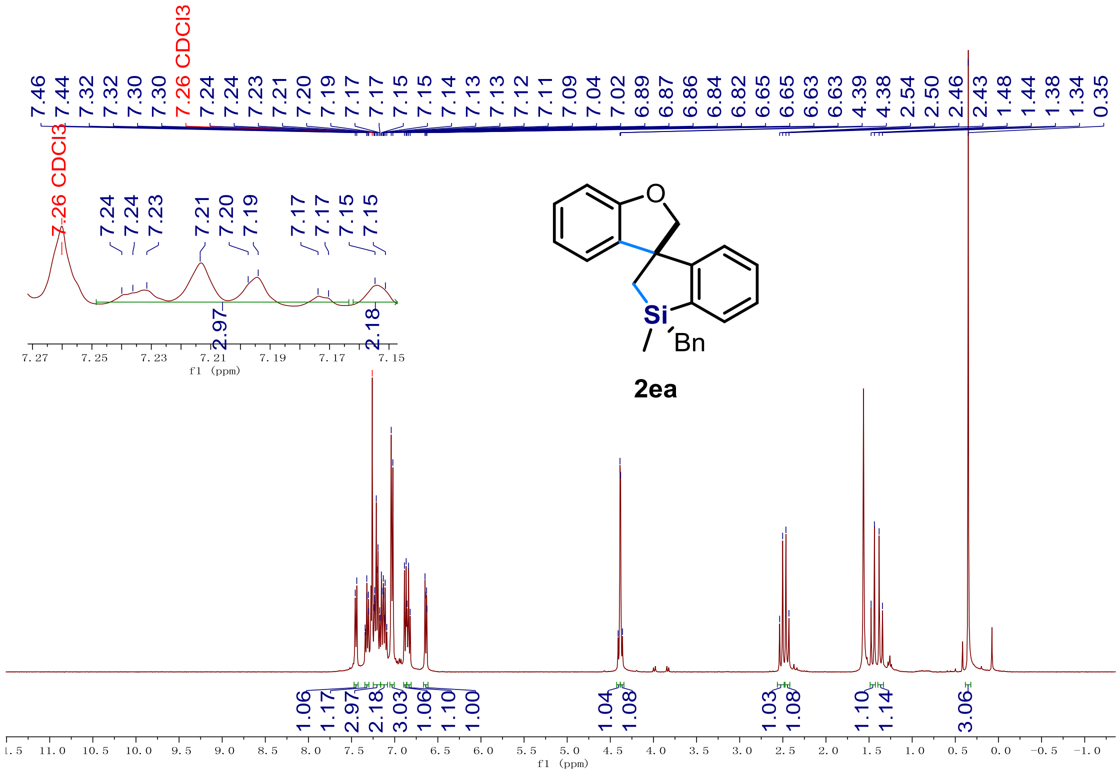

Supplementary Figure 178. ^{1}H NMR (400 MHz, $CDCl_3$) spectra of the first diastereoisomer **2ea**

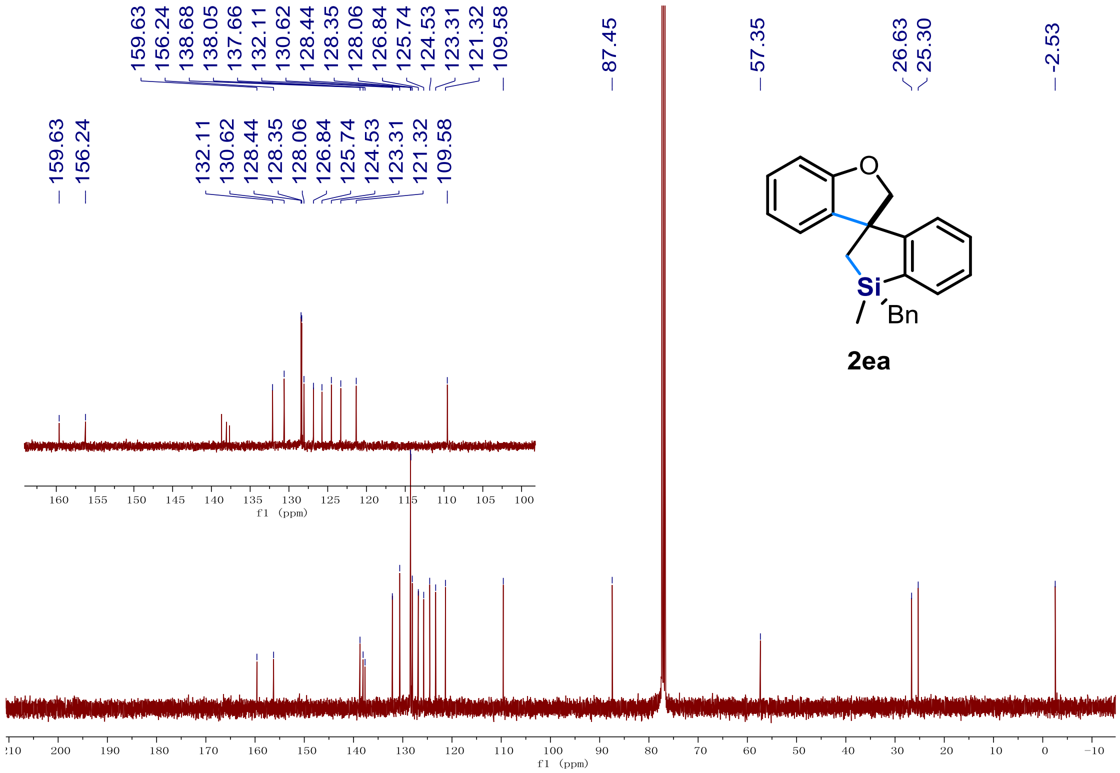

Supplementary Figure 177. ^{13}C NMR (101 MHz, $CDCl_3$) spectra of the first diastereoisomer **2ea**

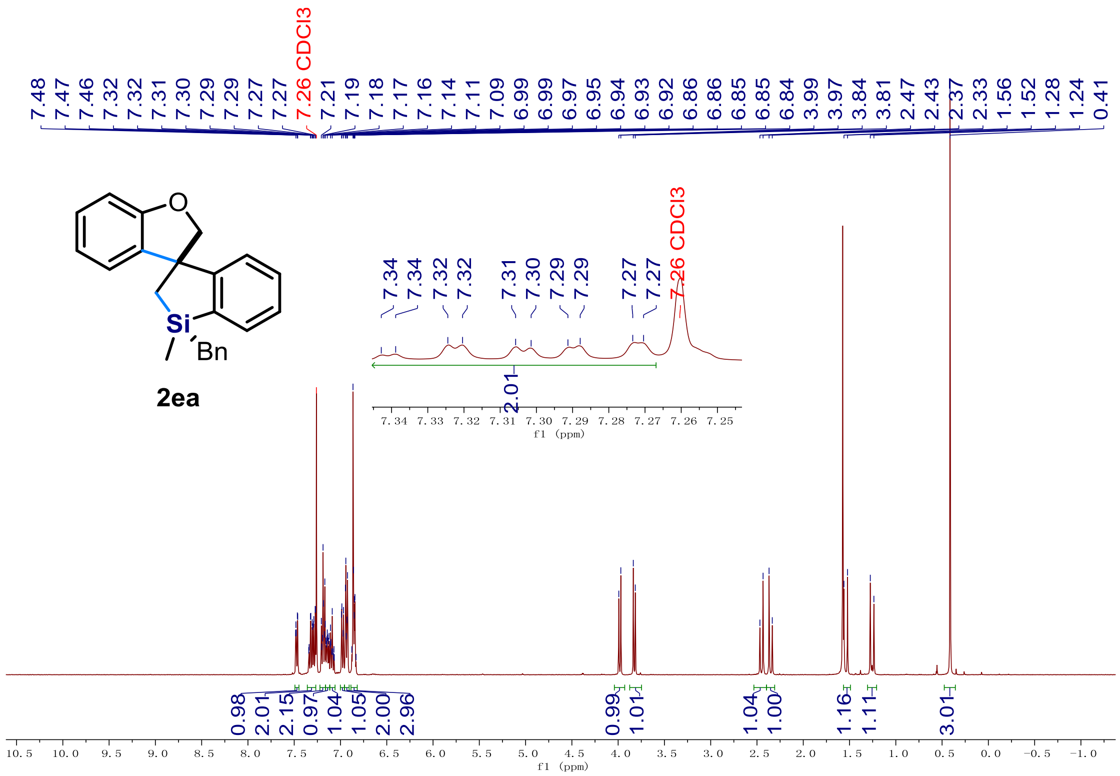

Supplementary Figure 178. ^{1}**H NMR (400 MHz, CDCl$_3$)** spectra of the second diastereoisomer **2ea**

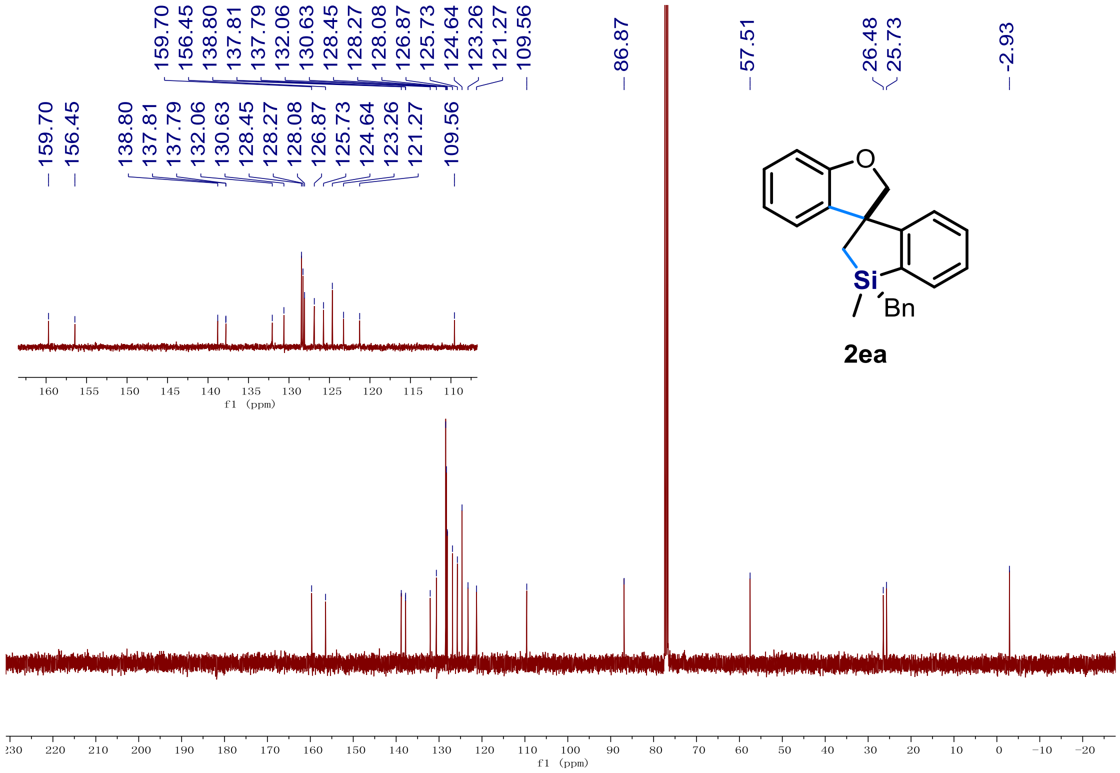

Supplementary Figure 179. ^{13}**C NMR (101 MHz, CDCl$_3$)** spectra of the second diastereoisomer **2ea**

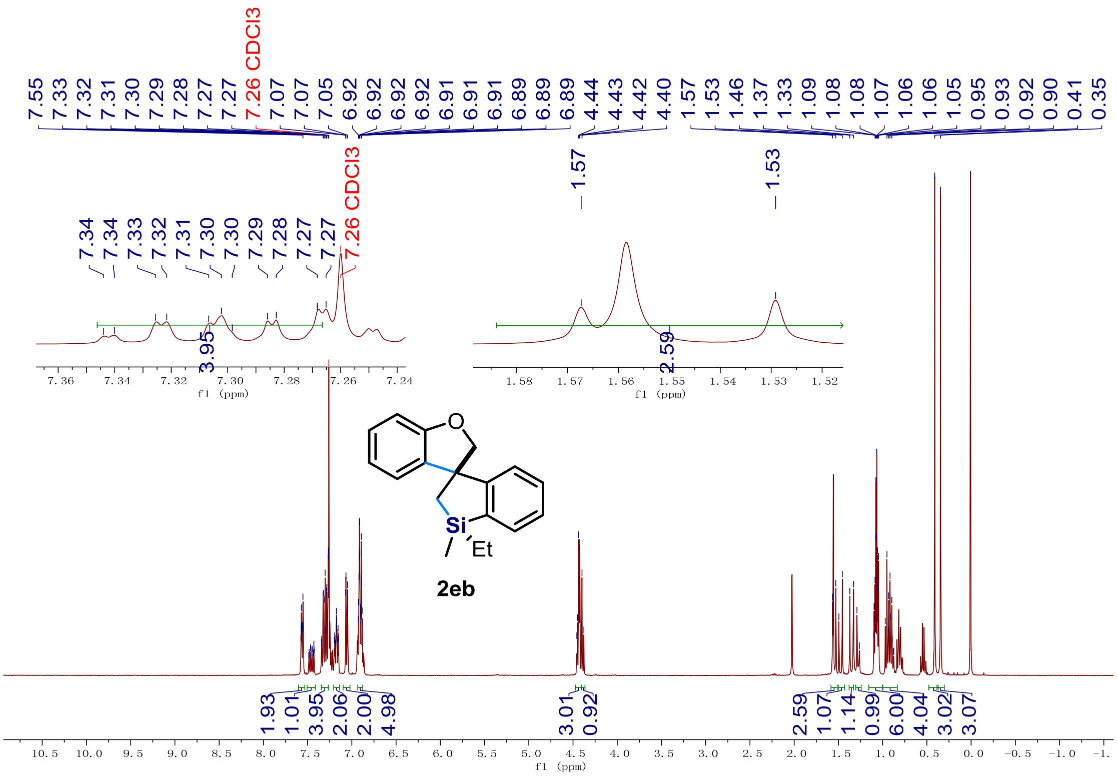

Supplementary Figure 180. ^{1}**H NMR (400 MHz, CDCl$_3$)** spectra of **2eb**

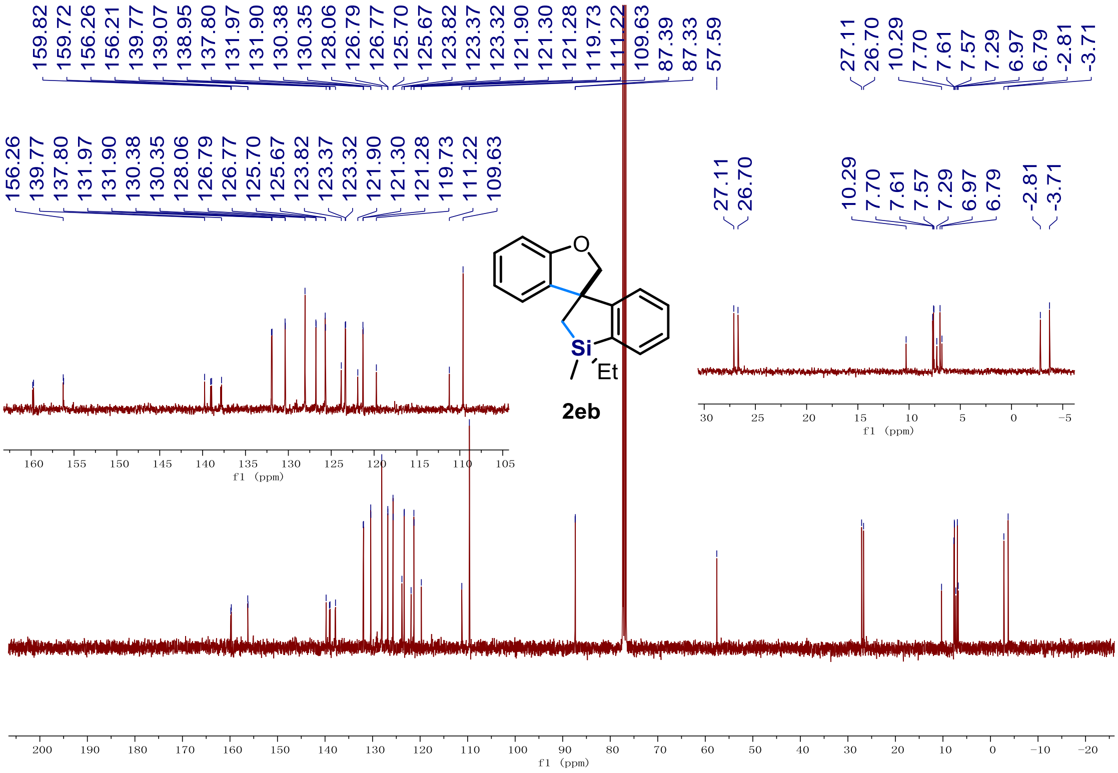

Supplementary Figure 181. ^{13}**C NMR (101 MHz, CDCl$_3$)** spectra of **2eb**

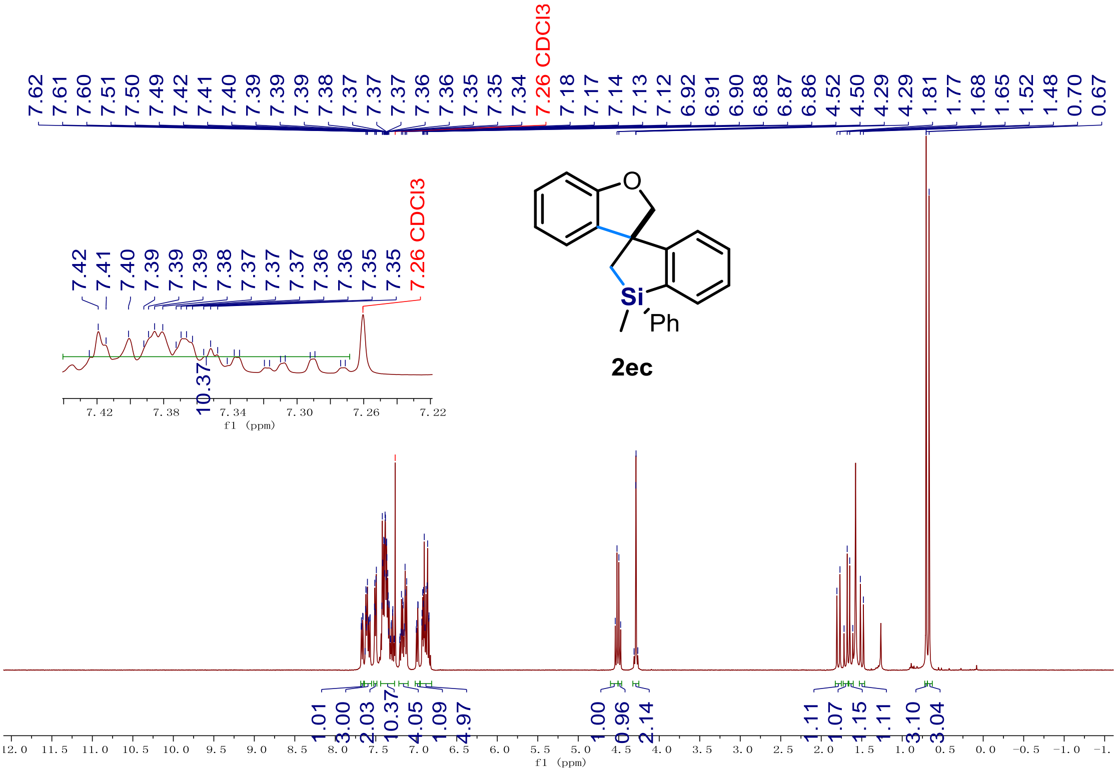

Supplementary Figure 182. **^{1}H NMR (400 MHz, CDCl$_3$)** spectra of **2ec**

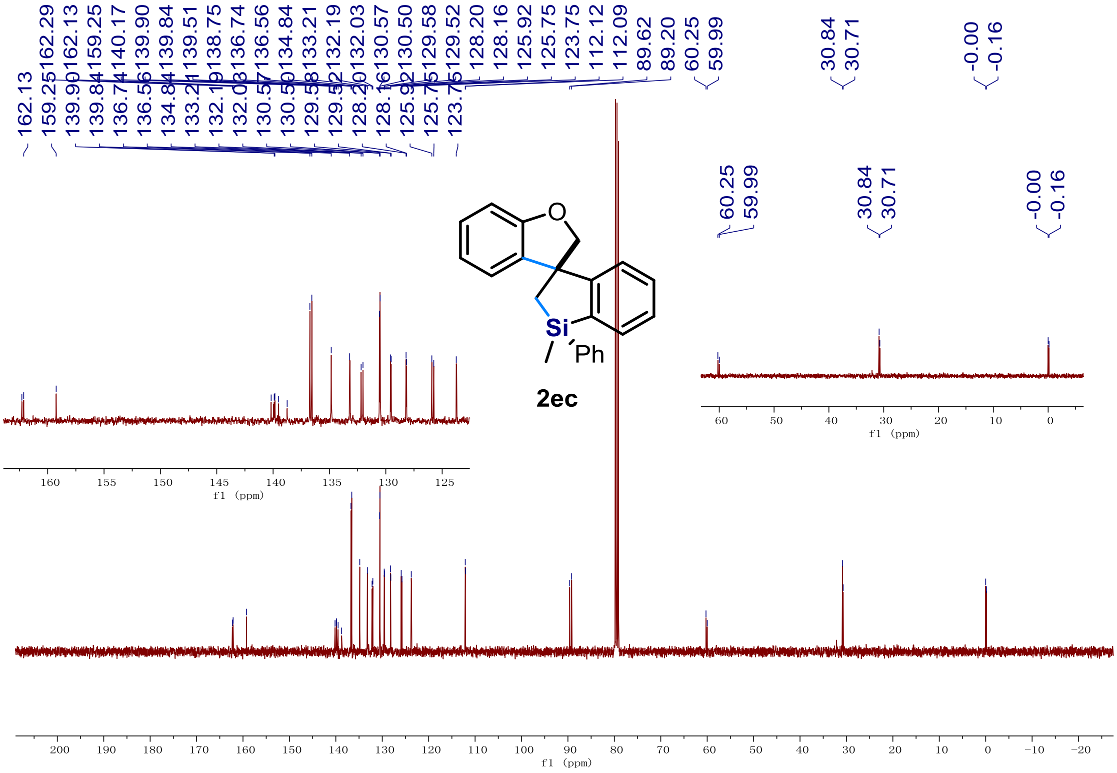

Supplementary Figure 183. **^{13}C NMR (101 MHz, CDCl$_3$)** spectra of **2ec**

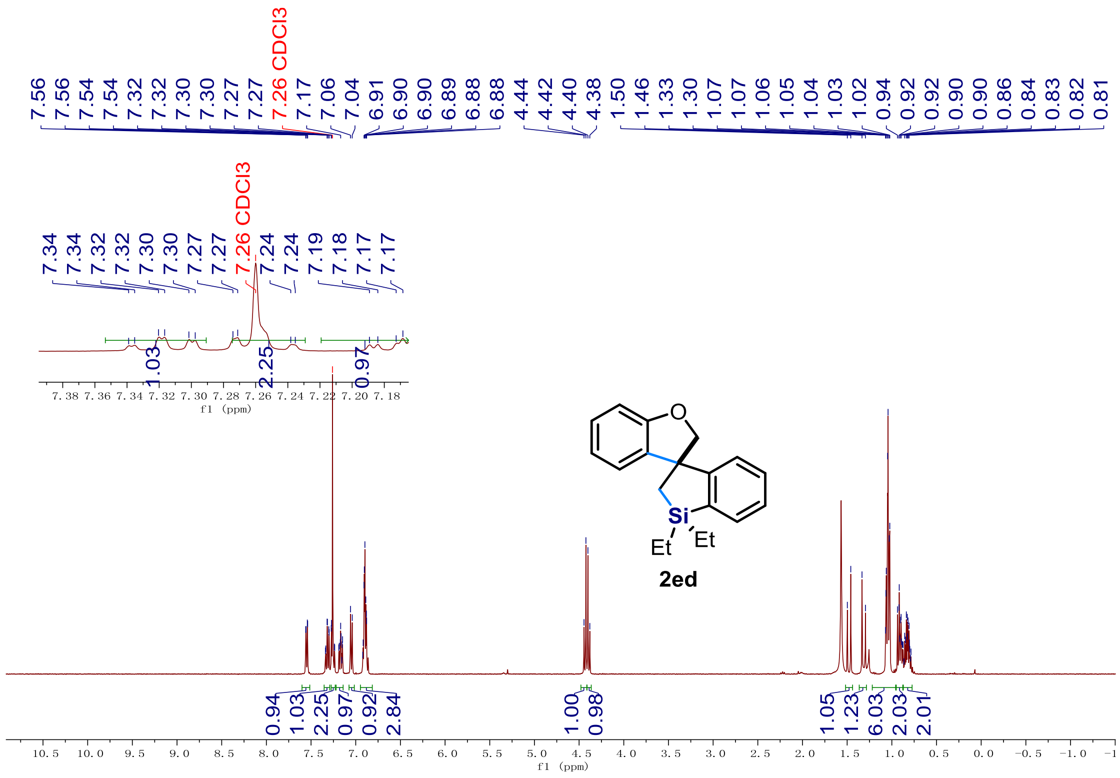

Supplementary Figure 184. ^{1}**H NMR (400 MHz, CDCl$_3$)** spectra of **2ed**

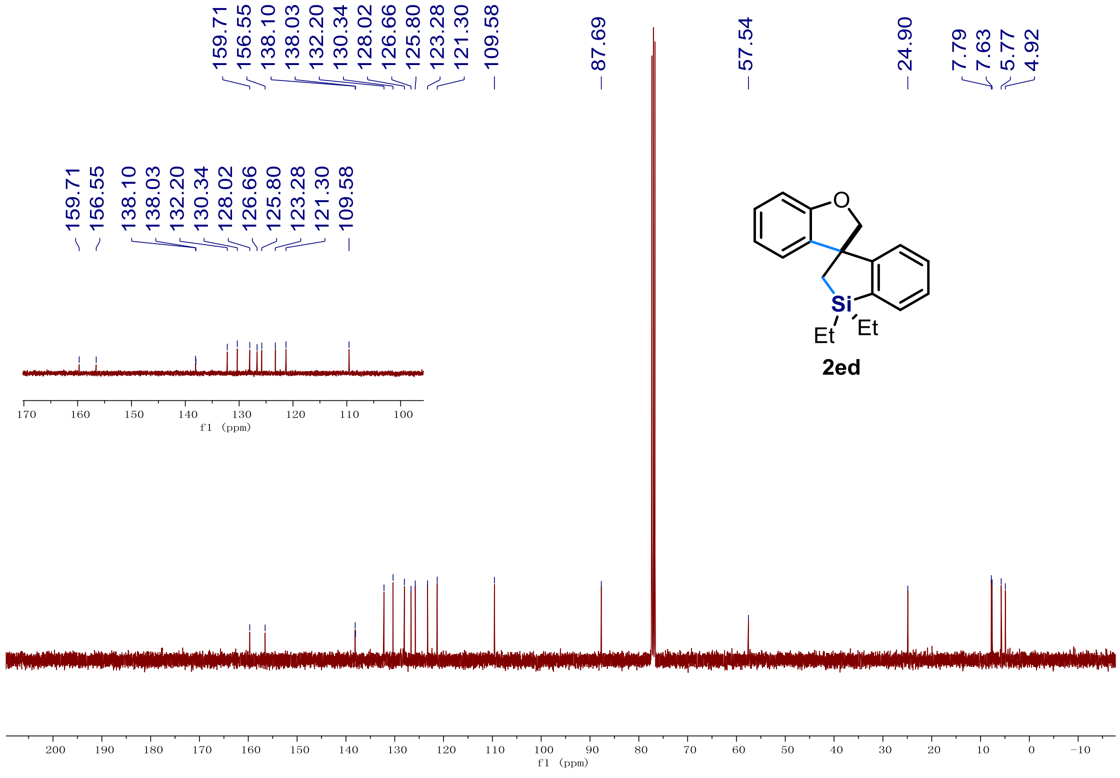

Supplementary Figure 185. ^{13}**C NMR (101 MHz, CDCl$_3$)** spectra of **2ed**

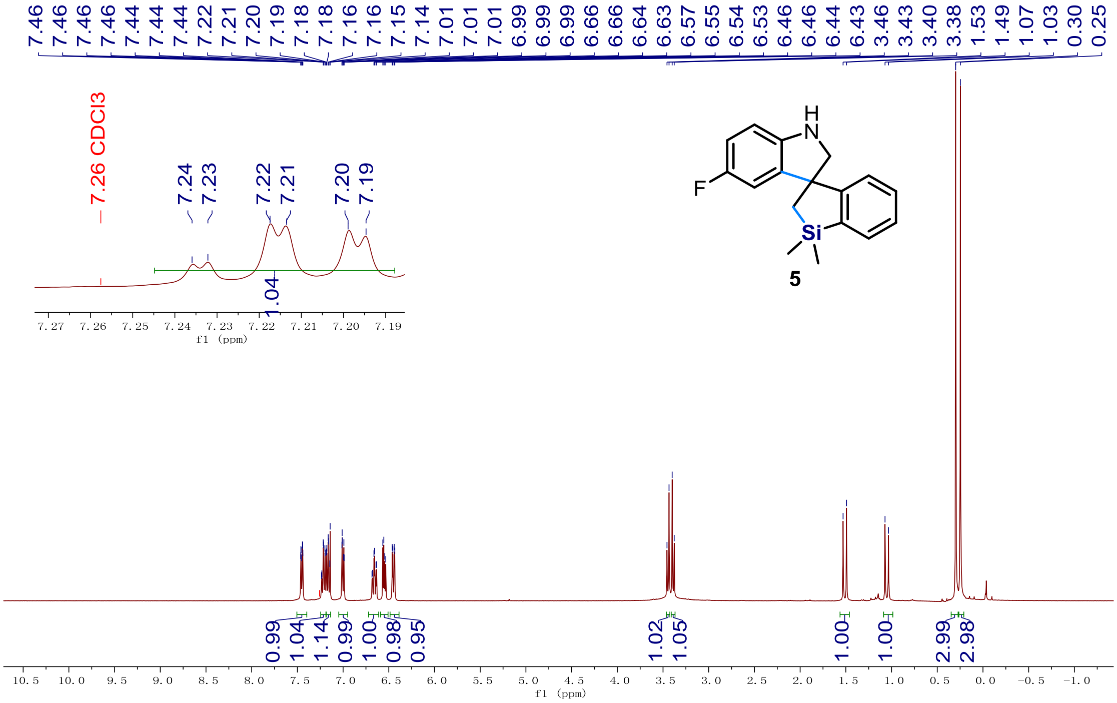

Supplementary Figure 186. ^{1}**H NMR (400 MHz, CDCl$_3$)** spectra of **5**

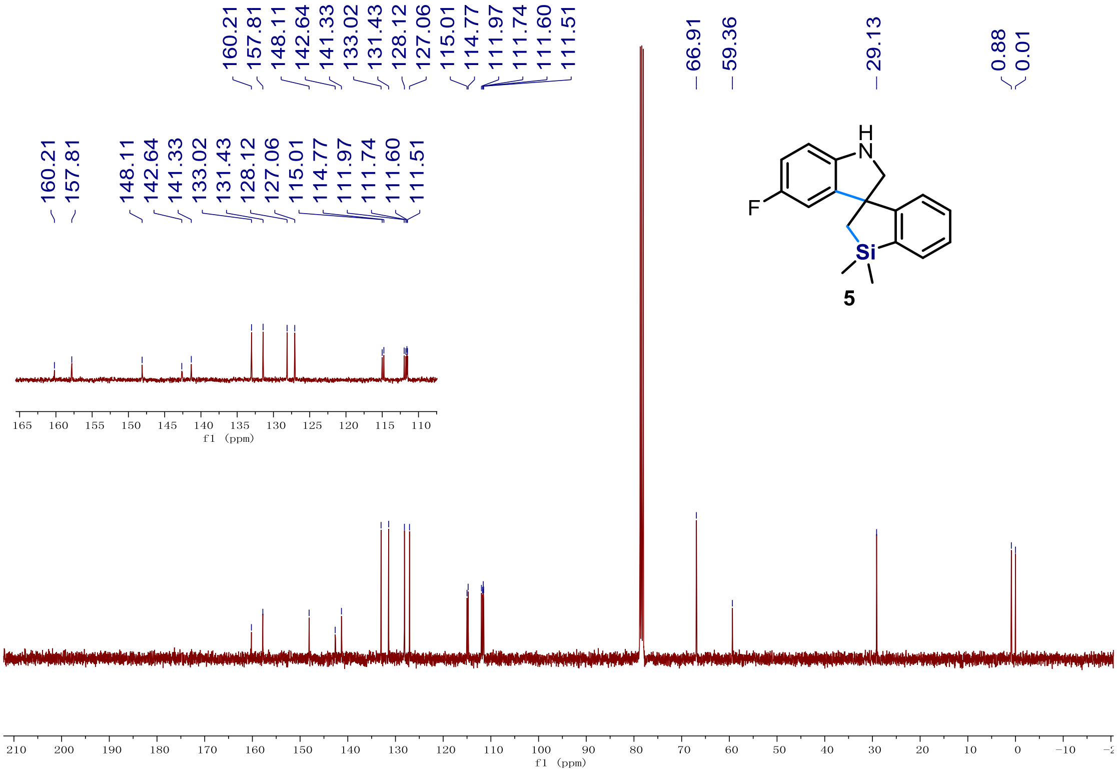

Supplementary Figure 187. ^{13}**C NMR (101 MHz, CDCl$_3$)** spectra of **5**

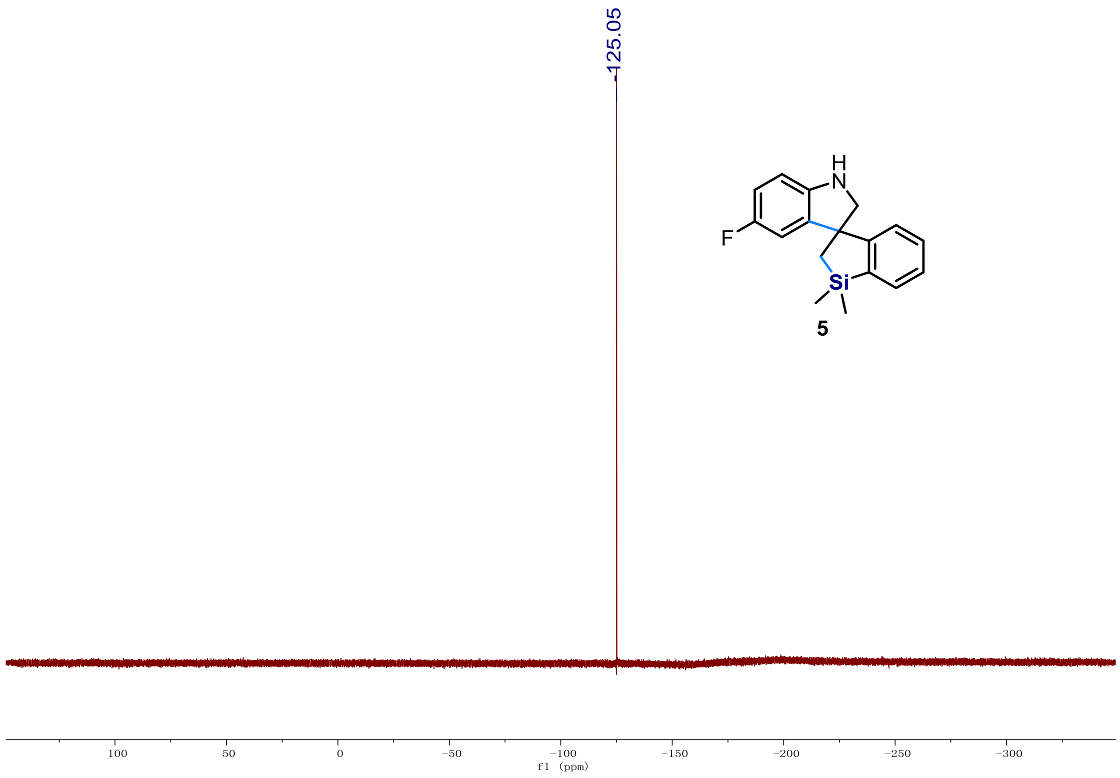

Supplementary Figure 188. ^{19}**F NMR (376 MHz, CDCl$_3$)** spectra of **5**

5. Supplementary References

1. X. Chang, P.-L. Ma, H.-C. Chen, C.-Y. Li, and P Wang, *Angew. Chem. Int. Ed.* **2020**, *59*, 8937–8940.
2. C. B. Tripathi and S. Mukherjee, *Angew. Chem. Int. Ed.* **2013**, *125*, 8608 –8611.
3. J.-L. Han, Y. Qin, C.-W. Ju, and D. Zhao, *Angew. Chem. Int. Ed.* **2020**, *59*, 6555 – 6560.
4. S. G. Newman and M. Lautens, *J. Am. Chem. Soc.* **2011**, *133*, 1778–1780.
5. Y.-Q. Zhang, X.-Q. Zhu, Y. Xu, H.-Z. Bu, J.-L. Wang, T.-Y. Zhai, J.-M. Zhou, and L.-W. Ye, *Green Chem.* **2019**, *21*, 3023-3028.
6. Y. Zhao, X. Xing, S. Zhang and D. Z. Wan, *Org. Biomol. Chem.* **2014**, *12*, 4314-4317.
7. J. Ye, Z. Shi, T. Sperger, Y. Yasukawa, C. Kingston, F Schoenebeck, M. Lautens, *Nat. Chem.* **2017**, *9*, 361-368.
8. T. Ankner, G. Hilmersson, *Org. Lett.* **2009**, *11,* 3, 503–506.
9. Y. Liang, S. Zhang, and Z. Xi, *J. Am. Chem. Soc.* **2011**, *133*, 9204–9207.
10. C. J. Jenks, S.-L. Chang, J. W. Anderegg, P. A. Thiel, and D. W. Lynch, *J. Phys. Rev. B* **1996**, *54*, 6301- 6306.
11. M. C. Militello and S. J. Simko, Palladium Oxide (PdO) by XPS. *Surface Science Spectra* **1994**, *3*, 395-401.
12. M. J. Frisch, G. W. Trucks, H. B. Schlegel, G. E. Scuseria, M. A. Robb, J. R. Cheeseman, G. Scalmani, V. Barone, G. A. Petersson, H. Nakatsuji, X. Li, M. Caricato, A. V. Marenich, J. Bloino, B. G. Janesko, R. Gomperts, B. Mennucci, H. P. Hratchian, J. V. Ortiz, A. F. Izmaylov, J. L. Sonnenberg, D. Williams - Young, F. Ding, F. Lipparini, F. Egidi, J. Goings, B. Peng, A. Petrone, T. Henderson, D. Ranasinghe, V. G. Zakrzewski, J. Gao, N. Rega, G. Zheng, W. Liang, M. Hada, M. Ehara, K. Toyota, R. Fukuda, J. Hasegawa, M. Ishida, T. Nakajima, Y. Honda, O. Kitao, H. Nakai, T. Vreven, K. Jr. Throssell, J. A. Montgomery, J. E. Peralta, F. Ogliaro, M. J. Bearpark, J. J. Heyd, E. N. Brothers, K. N. Kudin, V. N. Staroverov, T. A. Keith, R. Kobayashi, J. Normand, K. Raghavachari, A. P. Rendell, J. C. Burant, S. S. Iyengar, J. Tomasi, M. Cossi, J. M. Millam, M. Klene, C. Adamo, R. Cammi, J. W. Ochterski, R. L. Martin, K. Morokuma, O. Farkas, J. B. Foresman, D. J. Fox, Gaussian 16, Revision A.03; Gaussian, Inc.: Wallingford, CT, **2016**.
13. Y. Zhao & D. G. Truhlar, *J. Chem. Phys.* **2006**, *125*, 194101.
14. F. Weigend, R. Ahlrichs, *Phys. Chem. Chem. Phys.* **2005**, 7, 3297-3305.
15. a) C. Adamo, V. Barone, *J. Chem. Phys.* **1999**, *110*, 6158- 6170; b) S. Grimme, S.

Ehrlich, L. Georigk, *J. Comput. Chem.* **2011**, *32*, 1456-1465.

16. A. V. Marenich, C. J. Cramer, D. G. Truhlar, *J. Phys. Chem. B* **2009**, *113*, 6378–6396.
17. a) G. Luchini, J. V. Alegre-Requena, I. Funes-Ardoiz, R. S. Paton, *F1000Research* **2006**, 9, 291-305; b) S. Grimme, *Chem. Eur. J.* **2012**, *18*, 9955-9964; c) Y. Li, J. Gomes, S. M. Sharada, A. T. Bell, M. Head-Gordon, *J. Phys. Chem. C*. **2015**, *119*, 1840–1850.
