# Supplementary material for: Sila-spirocyclization involving unstrained C(sp3)−Si bond cleavage
Source: Nat Commun. 2022 Nov 5;13:6697. doi: 10.1038/s41467-022-34466-4 (PMC9637223; doi:10.1038/s41467-022-34466-4)
Supplement: Supplementary file 2 — Description of Additional Supplementary Files [file 41467_2022_34466_MOESM2_ESM.pdf]

Description of Additional Supplementary Files

File Name: Supplementary Data 1
Description: Cartesian coordinates of DFT optimized structures.
