# Supplementary material for: Sila-spirocyclization involving unstrained C(sp3)−Si bond cleavage
Source: Nat Commun. 2022 Nov 5;13:6697. doi: 10.1038/s41467-022-34466-4 (PMC9637223; doi:10.1038/s41467-022-34466-4)
Supplement: Supplementary file 3 — Supplementary Data 1 [file 41467_2022_34466_MOESM3_ESM.docx]

**Supplementary Data 1**

**Cartesian coordinates and energies of the optimized structures:**

The Cartesian coordinates of optimized structures are given below in the standard format, and units are in Å.

**1aa**

C 2.31634100 -0.07163600 -0.26998300

C 1.58917300 0.95023900 -0.90647800

C 2.08927000 2.25794300 -0.87071600

C 3.28533000 2.53994000 -0.21274200

C 4.00018100 1.52403400 0.41685200

C 3.51326000 0.21637400 0.38244800

H 1.53457400 3.06605900 -1.35744300

H 3.65757000 3.57054700 -0.19574000

H 4.93869600 1.74154600 0.93812700

H 4.06499100 -0.58886200 0.87905900

I 1.55429900 -2.03896100 -0.26199800

O 0.43594700 0.60748600 -1.52851700

C -0.56447100 1.59573700 -1.69822500

H -0.23891200 2.38201000 -2.40871100

H -1.38436500 1.05019700 -2.19670300

C -1.01212000 2.20353600 -0.39236900

C -1.29458400 3.51113200 -0.30879800

H -1.19497300 4.17783800 -1.17685800

H -1.65521900 3.95995100 0.62537900

C -1.03999300 1.30986400 0.79747900

C -0.17912400 1.64376400 1.85490700

C -1.84563800 0.14721700 0.88205000

C -0.08211900 0.84478400 2.99124700

H 0.44897000 2.53870200 1.75980600

C -1.73231900 -0.63043400 2.04775900

C -0.86085200 -0.30483900 3.08611800

H 0.60838200 1.12013600 3.79698800

H -2.35062300 -1.53109900 2.15600600

H -0.79444100 -0.94989600 3.97010200

Si -3.13505000 -0.41730000 -0.39515200

C -4.43699700 -1.43646900 0.50940000

H -4.04391400 -2.39810800 0.88533700

H -4.86977600 -0.89507900 1.37029700

H -5.26993200 -1.68060600 -0.17584000

C -2.36471700 -1.53747400 -1.69950600

H -3.09628400 -1.78486100 -2.49157900

H -1.46267800 -1.12565700 -2.18398500

H -2.05516100 -2.49090000 -1.23291300

C -4.04745200 1.04207000 -1.16625200

H -4.90132400 0.66763400 -1.76171100

H -4.46335600 1.69953600 -0.38075100

H -3.44593600 1.68346400 -1.83185300

**2aa**

C -1.50601500 -0.50350100 0.04490300

C -2.63913100 0.11014900 0.58322200

C -3.88738800 0.02438900 -0.02358500

C -3.97722000 -0.71130600 -1.20903100

C -2.85633100 -1.33655100 -1.76089100

C -1.60857800 -1.22653600 -1.13552600

H -4.75982200 0.51206800 0.42258600

H -4.94872700 -0.80195900 -1.70882700

H -2.95318200 -1.91079500 -2.68870200

H -0.72459300 -1.70395800 -1.57602800

O -2.37925900 0.78950300 1.72943800

C -1.06304800 0.40999800 2.14429300

H -1.16140500 -0.37908900 2.91612800

H -0.58223600 1.29149500 2.60011200

C -0.30999400 -0.13698400 0.90761000

C 0.62405000 -1.31962800 1.25652900

H 0.97995700 -1.20000500 2.29969400

H 0.07525800 -2.27799700 1.23532000

C 0.50360300 0.94548300 0.19251400

C 0.01334500 2.24257700 -0.00244700

C 1.76522000 0.58271600 -0.31536500

C 0.77890600 3.17843200 -0.69394400

H -0.97456600 2.52362300 0.38576700

C 2.51902200 1.53643200 -1.01382800

C 2.03287500 2.82802800 -1.20340000

H 0.39341100 4.19438600 -0.84090200

H 3.50789400 1.27064700 -1.41319700

H 2.63005100 3.56917600 -1.74764900

Si 2.15726600 -1.19277100 0.14255100

C 2.22234400 -2.36674100 -1.32482700

H 3.10416300 -2.14855100 -1.95513900

H 1.33593700 -2.29897600 -1.97830300

H 2.31414400 -3.41842100 -0.99664300

C 3.76360400 -1.36349600 1.10296100

H 4.63534300 -1.10209600 0.47504100

H 3.92359600 -2.39784400 1.45906200

H 3.78554100 -0.69969800 1.98532900

AgI

Ag 0.00000000 0.00000000 -1.38203600

I 0.00000000 0.00000000 1.22557900

AgOAc

C -1.50965700 0.01127800 -0.00040200

O -0.91592600 1.12416200 -0.00008700

O -0.91573600 -1.10434300 -0.00001100

C -3.01955700 -0.00483400 -0.00012700

H -3.38486500 -0.55507700 -0.88320300

H -3.38458100 -0.55349700 0.88406800

H -3.43828600 1.01194700 -0.00089600

Ag 1.10715500 -0.00214000 0.00008500

LiOAc

C -0.04443900 0.00797900 -0.00005400

O 0.59169500 1.10357000 -0.00002100

O 0.56210000 -1.10622900 0.00005100

C -1.55024500 0.01292100 -0.00003300

H -1.92514100 -0.53361700 -0.88206700

H -1.92504000 -0.53203500 0.88303300

H -1.95383600 1.03645700 -0.00085800

Li 2.04725400 -0.02498000 0.00005600

LiO*^t^*Bu

O 1.26791900 -0.00100400 -0.00029700

C -0.10215300 -0.00002400 -0.00004500

C -0.62828400 1.34631300 -0.52006900

C -0.63029800 -1.12310600 -0.90535200

C -0.62908200 -0.22207900 1.42574400

H -0.25763900 2.17035900 0.11757200

H -0.25865100 1.52716800 -1.54654700

H -1.73399700 1.39942900 -0.53993200

H -0.26131900 -2.10283500 -0.54900200

H -1.73608300 -1.16568500 -0.94026100

H -0.26060800 -0.98349000 -1.93822900

H -1.73482000 -0.22959500 1.48133900

H -0.26023700 -1.18701800 1.82051000

H -0.25831400 0.57569400 2.09589000

Li 2.85240600 -0.00087400 -0.00021200

MeO*^t^*Bu

O 0.78241500 0.01461300 -0.80534000

C -0.39897600 -0.00114200 0.00954100

C -0.37358800 -1.16356800 0.99700500

C -0.55389000 1.32793900 0.74384700

C -1.52539200 -0.18291100 -0.99462900

H -0.16374600 -2.11421400 0.47509700

H 0.39018400 -1.02738100 1.78313500

H -1.34845600 -1.26231600 1.50585400

H -0.53233400 2.16876000 0.02871900

H -1.51294000 1.36577600 1.29010500

H 0.24728400 1.49019900 1.48621900

H -2.51071800 -0.17842500 -0.49747700

H -1.50654600 0.62969300 -1.74086700

H -1.40955300 -1.13763600 -1.53576800

C 2.01136600 0.00716000 -0.15244000

H 2.22177200 -0.95093400 0.36913600

H 2.78494100 0.14381700 -0.92597000

H 2.12367700 0.83089300 0.58458900

NR_3_

N 0.00005000 -0.00000100 -0.35111700

C -0.66167300 1.21113700 0.05633900

H -0.12608500 2.09081800 -0.34288400

H -1.69119200 1.23566900 -0.34311800

H -0.72838200 1.33288700 1.16722500

C -0.71810200 -1.17855800 0.05633900

H -1.74760900 -1.15452000 -0.34318300

H -0.22458000 -2.08253700 -0.34282200

H -0.79054800 -1.29699000 1.16722500

C 1.37976700 -0.03257800 0.05634800

H 1.87370900 -0.93623400 -0.34302100

H 1.91579600 0.84678100 -0.34299000

H 1.51859700 -0.03587200 1.16723000

**IM1^II^**

C -1.20831300 1.13728500 -0.24036900

C -0.87342800 1.74501600 0.97670400

C -1.39596600 3.00496900 1.28942800

C -2.23719100 3.65885800 0.39015600

C -2.57730500 3.04884000 -0.81718600

C -2.07468200 1.78046400 -1.12217400

H -1.15145900 3.46466700 2.25517500

H -2.64379900 4.64392500 0.64638000

H -3.25076100 3.55171800 -1.52114500

H -2.36309100 1.28727700 -2.05836600

O -0.07292800 1.06747400 1.86867100

C 1.11068600 1.70198400 2.32204600

H 0.97879200 2.80412700 2.30926900

H 1.26359700 1.40223200 3.37339400

C 2.29654800 1.31388100 1.47517700

C 3.49388100 1.02815800 1.99914400

H 3.65929800 1.02845300 3.08517700

H 4.35821900 0.79857100 1.36321300

C 2.01829800 1.28522300 0.01610200

C 1.79979600 2.48919200 -0.64811100

C 1.86938000 0.04109400 -0.67111400

C 1.44993800 2.52336000 -2.00453400

H 1.90116800 3.43086800 -0.09362900

C 1.48354800 0.11180600 -2.04091000

C 1.29029600 1.33899400 -2.70161800

H 1.29141700 3.48673700 -2.50212300

H 1.45609400 -0.80467000 -2.64604000

H 1.01012400 1.34583000 -3.76081100

Si 2.25167200 -1.68363900 0.05670500

C 1.28664700 -2.92785900 -1.00406800

H 0.18063200 -2.80808600 -1.06197200

H 1.65986900 -2.98831300 -2.04268800

H 1.42585500 -3.93155900 -0.55870700

C 1.74722300 -1.89302200 1.85344900

H 1.50355900 -2.95340800 2.05304700

H 2.55190800 -1.59988000 2.55015500

H 0.85058000 -1.29786800 2.10068000

C 4.07692200 -2.08971900 -0.16273000

H 4.28478700 -3.13484900 0.13319700

H 4.39904400 -1.97396500 -1.21343200

H 4.72448000 -1.44265300 0.45493600

Pd -0.40734200 -0.60558600 -0.71873700

O -2.14182900 -1.59228600 -0.69084000

C -2.90932900 -1.84469300 0.45656900

C -2.02286500 -2.04774500 1.68392300

C -3.91019000 -0.71754300 0.71676500

C -3.66685600 -3.14047500 0.14498600

H -1.27393000 -2.84035900 1.49398100

H -1.48093600 -1.11199500 1.92206000

H -2.60664200 -2.33769000 2.57851200

H -4.50756500 -0.51891400 -0.19084700

H -4.60500800 -0.97094600 1.53974500

H -3.39296900 0.21947500 0.98900700

H -4.32772300 -3.43194500 0.98196400

H -4.28692000 -3.01298800 -0.75915300

H -2.95844500 -3.96581700 -0.04539300

**IM1^II^-S1**

C 0.97465000 -1.61141500 0.23536000

C 2.09611000 -1.36075500 -0.56783900

C 3.28257600 -2.07754500 -0.40424600

C 3.35625000 -3.07726600 0.56880300

C 2.24712900 -3.35450800 1.36708500

C 1.06440100 -2.62410500 1.19300400

H 4.15003300 -1.85556500 -1.03833100

H 4.28683900 -3.64192200 0.69897800

H 2.30114100 -4.13610000 2.13472300

H 0.20320200 -2.83453500 1.84599500

O 1.96794400 -0.37939100 -1.52823000

C 2.95158200 0.63166900 -1.56255800

H 3.89581800 0.26976500 -2.02124500

H 2.54110000 1.38564000 -2.26069900

C 3.24533900 1.23367500 -0.20548500

C 4.45554600 1.74933200 0.05768100

H 5.26176700 1.73114900 -0.68799400

H 4.67745300 2.23238500 1.01757000

C 2.18121600 1.13962600 0.82441200

C 2.53814500 0.57811400 2.05369500

C 0.81976700 1.49563200 0.57424000

C 1.59094900 0.28433600 3.03850500

H 3.58766300 0.30291100 2.21636600

C -0.12528500 1.18426300 1.59281100

C 0.25565500 0.55984000 2.79936200

H 1.90634000 -0.18351300 3.97789500

H -1.14695700 1.57075200 1.49638300

H -0.50423500 0.33432600 3.55699000

Si 0.26646400 2.70068900 -0.81315200

C -1.16642500 3.69535400 -0.12727600

H -2.04564600 3.03633300 -0.01833900

H -0.94723300 4.15686800 0.85293800

H -1.43833000 4.50908200 -0.82519200

C -0.33863300 1.83924700 -2.36775100

H -0.43155500 2.55414600 -3.20765300

H 0.29875900 0.99879500 -2.69336100

H -1.34679500 1.44016800 -2.14903100

C 1.66819400 3.91387600 -1.17487100

H 1.26171400 4.75367300 -1.76985000

H 2.07236600 4.34674600 -0.24127900

H 2.52482700 3.50646700 -1.73741000

Pd -0.70244400 -0.52292500 0.05694800

O -2.52960400 0.55070000 -0.12023700

C -3.59268400 0.42415300 0.77394800

C -4.81975000 -0.13286100 0.03348800

C -3.26124300 -0.50338700 1.95220100

C -3.97403500 1.79993800 1.34134400

H -5.04507100 0.49840000 -0.84502800

H -4.63913800 -1.15964500 -0.33482800

H -5.72320500 -0.16497500 0.67237300

H -2.44586500 -0.08583400 2.56939300

H -4.13292000 -0.65520700 2.61793300

H -2.93165800 -1.50176100 1.60538800

H -4.84614300 1.74756300 2.02150200

H -3.13609600 2.24462000 1.91023700

H -4.22595700 2.49580300 0.52111900

N -1.41459800 -2.01641500 -1.48568700

C -2.22330900 -2.99693900 -0.76133600

H -2.61594300 -3.77278300 -1.45433000

H -1.61197900 -3.49029800 0.01288700

H -3.07525200 -2.49172300 -0.28270600

C -0.34094300 -2.70701400 -2.20827500

H 0.23429100 -3.34785500 -1.52375700

H -0.77563100 -3.33956200 -3.01142300

H 0.34253900 -1.96974300 -2.65762900

C -2.24685600 -1.28500400 -2.44860000

H -2.92897600 -0.61395800 -1.90255200

H -1.59709300 -0.67117500 -3.09146900

H -2.80686000 -1.99962900 -3.08867700

**IM1^II^-S2**

C 0.68615400 -1.68887300 -0.21263400

C 1.54288600 -1.18614400 -1.19804300

C 2.75132400 -1.82960200 -1.47362300

C 3.09134400 -2.98903700 -0.77531000

C 2.22669100 -3.50580500 0.18933700

C 1.01937900 -2.85478700 0.46966400

H 3.42357000 -1.42057500 -2.23772700

H 4.03827100 -3.49568700 -0.99414000

H 2.48764700 -4.42301100 0.73049000

H 0.34596700 -3.25760900 1.23707400

O 1.14748800 -0.04598900 -1.85775500

C 2.03751400 1.04713800 -1.84646300

H 2.90175900 0.87800300 -2.52202300

H 1.45849400 1.87774000 -2.29245400

C 2.53912100 1.40276700 -0.46463300

C 3.72029500 2.02314200 -0.31700800

H 4.35420500 2.25979800 -1.18205200

H 4.08382300 2.34253600 0.66750400

C 1.72384000 0.98430800 0.69985300

C 2.38541900 0.31844700 1.72909300

C 0.29721700 1.18426300 0.77214700

C 1.70527900 -0.20472900 2.83820000

H 3.46610400 0.15673500 1.63662400

C -0.36074900 0.63677900 1.91991100

C 0.33543100 -0.06217800 2.93140800

H 2.26408600 -0.73859200 3.61522700

H -1.40460400 0.91157600 2.12197800

H -0.21770000 -0.46078300 3.78911400

Si -0.64857200 2.53310100 -0.22613600

C -2.08762500 3.08548400 0.84859100

H -2.84190400 2.28617900 0.96198000

H -1.77310000 3.41439400 1.85561900

H -2.59739700 3.94211200 0.36940600

C -1.37664900 1.95593800 -1.85976400

H -1.77685800 2.82689200 -2.41375600

H -0.67741600 1.41961200 -2.52099800

H -2.22570200 1.27538200 -1.66496800

C 0.49384600 4.01255100 -0.46968400

H -0.09724300 4.87308700 -0.83558700

H 0.96055000 4.31989500 0.48414200

H 1.31270700 3.85202300 -1.19137700

Pd -0.97636400 -0.69162000 0.17939000

O -3.17505600 -0.16684300 0.25971700

C -3.38829200 -1.25066200 -0.35086100

O -2.41709500 -2.02162700 -0.65477500

C -4.77127000 -1.64392800 -0.76445800

H -4.92421200 -1.37402200 -1.82400700

H -4.91073700 -2.73339800 -0.68414300

H -5.52720500 -1.11593100 -0.16377400

**IM1^II^-S3**

C -0.92452500 -1.50100500 0.61626500

C 0.04240600 -1.98578900 -0.28162600

C 0.58085500 -3.26576400 -0.11855600

C 0.18004400 -4.05698600 0.96109700

C -0.75273700 -3.57574500 1.87500000

C -1.30317700 -2.29969700 1.69396400

H 1.33257500 -3.64685500 -0.81891000

H 0.61434200 -5.05581700 1.08606900

H -1.05761200 -4.18740200 2.73240800

H -2.03836300 -1.92333800 2.41779200

O 0.38221100 -1.15418600 -1.32051100

C 1.60286200 -1.37316500 -1.99322600

H 1.56826200 -2.30766200 -2.59243000

H 1.65797200 -0.54475100 -2.72340000

C 2.82293500 -1.38445700 -1.09731700

C 3.91410900 -2.06903100 -1.47293600

H 3.93698300 -2.65106300 -2.40504100

H 4.83181800 -2.05552800 -0.87197300

C 2.74145700 -0.68912800 0.21203600

C 3.02850600 -1.46656800 1.34534500

C 2.33431000 0.66055500 0.36558400

C 2.89627000 -0.95421600 2.63180500

H 3.31778300 -2.51492600 1.20196200

C 2.21276100 1.14636400 1.67888500

C 2.46960700 0.35941700 2.79976000

H 3.10790700 -1.58971000 3.49976000

H 1.89358600 2.18143200 1.84337500

H 2.33411900 0.77754100 3.80394600

Si 2.14501500 1.93477400 -1.03075300

C 2.30149500 3.64657800 -0.25604500

H 1.43790800 3.89560700 0.38731000

H 3.21427600 3.75561100 0.35701100

H 2.34272800 4.41626400 -1.04922500

C 0.50135100 1.94651500 -1.95507100

H 0.37244500 1.11010300 -2.66163800

H -0.35973200 1.92058400 -1.26006900

H 0.42785900 2.88467800 -2.53851800

C 3.55809600 1.73914300 -2.26559700

H 3.53373400 2.55592200 -3.01125200

H 4.53772200 1.78841200 -1.75561000

H 3.53944300 0.78637500 -2.82331300

Pd -1.77401700 0.25572700 0.29463800

N -3.07346400 -0.55861000 -1.23268400

C -4.41744300 -0.30925600 -0.69720500

H -5.19239300 -0.52929400 -1.46252200

H -4.58935300 -0.95209600 0.18083500

H -4.50358400 0.74517700 -0.38835900

C -2.93446500 -1.96804100 -1.60730100

H -3.06490500 -2.61016000 -0.72271500

H -3.69400700 -2.23551400 -2.37151700

H -1.93050200 -2.14344200 -2.02016000

C -2.82388300 0.29836800 -2.39848400

H -2.89890500 1.35787000 -2.10345600

H -1.80765900 0.10734000 -2.77628300

H -3.56116800 0.09001000 -3.20303600

O -0.85493200 1.57516700 1.68735400

C -1.55189500 2.56260700 1.28723700

O -2.40180800 2.41324000 0.36333400

C -1.35430100 3.90486600 1.92260600

H -1.17272200 4.66679900 1.14636200

H -2.27518700 4.20499800 2.45146100

H -0.51816000 3.89567500 2.63839000

**IM1^II^-S4**

C 0.75902500 1.42586000 -0.03045700

C -0.08633600 1.74428000 -1.10105300

C -0.34851100 3.08724100 -1.38678200

C 0.25662300 4.09349000 -0.63321500

C 1.12256500 3.76676100 0.40825100

C 1.38715500 2.42379000 0.70444900

H -1.03367700 3.34201200 -2.20406200

H 0.05088900 5.14340600 -0.87037600

H 1.60899600 4.55486000 0.99445400

H 2.07071600 2.16425400 1.52029200

O -0.65358800 0.71106400 -1.79679600

C -2.03808500 0.78747800 -2.06377100

H -2.26517900 1.54290900 -2.84350600

H -2.28173600 -0.19317900 -2.51280200

C -2.86973300 1.05494900 -0.82895200

C -4.05458600 1.67612000 -0.93137400

H -4.43778700 2.02356400 -1.90022200

H -4.69622700 1.83791300 -0.05632200

C -2.28601500 0.68004300 0.48256400

C -2.24863400 1.67261900 1.46541500

C -1.69308400 -0.59872000 0.73715400

C -1.61924900 1.47572100 2.69892200

H -2.68604000 2.65142900 1.23413200

C -1.05042000 -0.76082500 1.99904800

C -1.00388500 0.26452400 2.96443500

H -1.59645500 2.28620000 3.43575800

H -0.70695900 -1.76046200 2.30459900

H -0.49646500 0.08647900 3.91904600

Si -2.02105600 -2.18128300 -0.29779000

C -1.84157200 -3.63456800 0.88805500

H -0.79811200 -3.79232700 1.21803200

H -2.46902100 -3.53411500 1.79204200

H -2.14914000 -4.56650800 0.37845300

C -0.80579800 -2.49658200 -1.69634900

H -1.09414800 -3.42704300 -2.22277500

H -0.72682700 -1.69260000 -2.44524700

H 0.21826500 -2.65047200 -1.30644800

C -3.79937500 -2.16176300 -0.91389400

H -4.05731900 -3.15154000 -1.33460700

H -4.50442800 -1.96411400 -0.08589000

H -4.00607800 -1.41250600 -1.69677600

Pd 0.76779500 -0.47183500 0.54594500

I 3.16190800 -0.71578900 -0.38608500

**IM1^II^-S5**

C 1.73564300 1.76412800 -0.15094800

C 0.65236000 2.43620100 -0.72261500

C 0.54911300 3.82115800 -0.74796300

C 1.58542300 4.56144800 -0.16943400

C 2.67648300 3.91364700 0.41039600

C 2.75943500 2.51504500 0.42328400

H -0.30924000 4.32516000 -1.20745500

H 1.53529300 5.65626900 -0.17604500

H 3.48335400 4.50534300 0.85932200

H 3.62456400 2.02072400 0.87966800

O -0.28451600 1.55621600 -1.29117100

C -1.66888500 1.82182600 -1.02461600

H -1.94737900 2.79285900 -1.47606300

H -2.20598700 1.02806600 -1.57512300

C -1.96969200 1.79018300 0.44595500

C -2.15423800 2.92376500 1.13510600

H -2.14849000 3.90426900 0.64052300

H -2.32785300 2.91395100 2.21918700

C -1.92460000 0.46181500 1.11532500

C -0.83267800 0.18823300 1.95464400

C -2.93973000 -0.50830100 0.92722000

C -0.72338800 -1.03195800 2.62005300

H -0.05612300 0.95292900 2.08387300

C -2.80263100 -1.72635300 1.61880300

C -1.71699300 -1.99318000 2.45351400

H 0.14534800 -1.22774000 3.25886700

H -3.57891900 -2.49688800 1.51496700

H -1.64820900 -2.95663000 2.97269800

Si -4.51429800 -0.30903700 -0.12248500

C -5.90925000 -1.20857100 0.76642700

H -5.75730000 -2.30172400 0.81220200

H -6.03675800 -0.85111800 1.80430500

H -6.86814700 -1.04198300 0.24181400

C -4.28613700 -1.13709400 -1.80291800

H -5.24118900 -1.14225200 -2.36092100

H -3.54132900 -0.63516900 -2.44720500

H -3.96814300 -2.19029600 -1.69275100

C -5.05839900 1.47393700 -0.37672900

H -6.06422000 1.47476600 -0.83817800

H -5.13435300 2.02598000 0.57679200

H -4.40142300 2.06087500 -1.04153300

Pd 1.27219600 -0.11856900 -0.53904200

I 3.38572000 -1.12332600 0.60302600

N 0.50586200 -2.10460500 -1.39214000

C 0.36410400 -3.16849900 -0.40283800

H -0.05244900 -4.09143700 -0.86332400

H 1.34875700 -3.39708800 0.03690000

H -0.30883400 -2.83674200 0.40464500

C 1.42776600 -2.50936100 -2.45080000

H 2.41314000 -2.73909800 -2.01310200

H 1.05243300 -3.40498200 -2.99329300

H 1.55533400 -1.68551200 -3.17284000

C -0.79320300 -1.74370000 -1.94692900

H -1.46481000 -1.41604000 -1.13296000

H -0.67649800 -0.91390100 -2.66679400

H -1.27250300 -2.59931200 -2.47314600

**IM2^II^**

C 0.04520100 -1.68952700 -0.05145100

C 0.22534900 -2.52467000 1.07423700

C 0.95181000 -3.70201700 0.99896900

C 1.52345500 -4.02765900 -0.24520000

C 1.39439900 -3.20598700 -1.36038900

C 0.64362800 -2.01488500 -1.28361600

H 1.04918500 -4.36109500 1.86714400

H 2.09826000 -4.95782200 -0.33066000

H 1.85776600 -3.49626500 -2.30962400

H 0.39070200 -1.45605800 -2.19240100

O -0.45520600 -2.07849400 2.14205100

C -0.97087600 -0.77175400 1.81498900

H -1.93744600 -0.67044400 2.33074000

H -0.25381200 -0.02222600 2.20799300

C -1.05796000 -0.68702700 0.27070300

C -0.47901400 0.58830600 -0.30522500

H -0.64430700 1.51044700 0.26465200

H -0.76243700 0.72388100 -1.36476600

C -2.44081000 -1.09121900 -0.23839700

C -2.63403400 -2.39741600 -0.70708400

C -3.53478400 -0.18497400 -0.21513300

C -3.87294800 -2.83772200 -1.16874800

H -1.79306100 -3.10095900 -0.71741700

C -4.77082900 -0.66293700 -0.69143500

C -4.95068600 -1.96102500 -1.16785500

H -3.98787500 -3.86674100 -1.52910600

H -5.64196300 0.00293200 -0.69359000

H -5.93392800 -2.28176300 -1.53144600

Si -3.57287300 1.63916400 0.36265900

C -2.98590000 2.81758100 -0.98629800

H -1.92320400 2.72148600 -1.26254800

H -3.57971200 2.66595600 -1.90671500

H -3.14837200 3.86363100 -0.66431100

C -2.72379300 1.99759000 2.01057200

H -2.91043200 3.05957700 2.26010900

H -3.17098800 1.39993400 2.82640400

H -1.63171100 1.85649600 2.05262600

C -5.37953200 2.09270800 0.67401500

H -5.42597700 3.09597800 1.13742000

H -5.98431900 2.14431100 -0.24900600

H -5.88426800 1.39308000 1.36503800

Pd 1.48470900 0.05185100 -0.15838600

N 3.75042100 -0.53508000 0.22457700

C 4.09025200 -1.94747400 0.18689700

H 5.16702100 -2.11082800 0.41894900

H 3.48791900 -2.50337800 0.92430800

H 3.88475300 -2.36258500 -0.81294500

C 4.02221400 0.00871300 1.55371400

H 3.42921600 -0.54320900 2.30387100

H 5.09963400 -0.08810500 1.81585600

H 3.72224800 1.06827200 1.57136300

C 4.51215700 0.18883200 -0.78741600

H 4.26725200 -0.20623900 -1.78901300

H 4.23039100 1.25143300 -0.74463800

H 5.60831400 0.08311800 -0.62435600

O 2.21986200 1.91548000 0.24170300

C 1.93542200 3.12992400 -0.39143700

C 0.83360400 3.90254000 0.34587300

C 3.22871800 3.95531400 -0.31782900

C 1.55502700 2.93653000 -1.85958500

H -0.13962600 3.38547500 0.29061900

H 1.09604400 4.00556600 1.41404300

H 0.68920000 4.91739800 -0.07126700

H 4.04250700 3.46444200 -0.88145300

H 3.09431300 4.97181500 -0.73300100

H 3.55733900 4.05374500 0.73216500

H 1.42940000 3.90041800 -2.38914600

H 2.33774200 2.35718100 -2.38455500

H 0.60722300 2.37693500 -1.95208200

**IM3^IV^**

C -0.66503900 1.80821400 0.05486200

C -0.32488800 2.97514100 -0.63087600

C 0.59845200 3.87961600 -0.11799600

C 1.17236600 3.57937800 1.12385000

C 0.83909900 2.41843000 1.82371900

C -0.08630100 1.51305900 1.28427200

H 0.85368900 4.79255400 -0.66501500

H 1.89723900 4.27903900 1.55597600

H 1.29903400 2.21165500 2.79529100

H -0.37355600 0.60836400 1.83976600

O -0.97950800 3.09530900 -1.81173500

C -2.02399000 2.11157600 -1.81925500

H -2.96249300 2.61406700 -1.52130900

H -2.13875400 1.75029400 -2.85359700

C -1.59654000 0.99903300 -0.83288100

C -0.75552000 -0.04893200 -1.57121500

H -0.09554200 0.45266300 -2.30662800

H -1.40219700 -0.72824600 -2.16224000

C -2.71140600 0.34787700 -0.03450800

C -3.84341000 1.05936400 0.38379500

C -2.54827000 -0.99085600 0.37544300

C -4.82697600 0.45133700 1.16022800

H -3.96597000 2.11487800 0.11550200

C -3.54533100 -1.58233900 1.16966500

C -4.68534400 -0.87964700 1.55081100

H -5.70832000 1.02603800 1.46792600

H -3.43142000 -2.62427500 1.50111700

H -5.45627300 -1.36410500 2.16107400

Si -1.00965400 -2.00133500 -0.03792200

C -0.40447800 -2.84625900 1.53503800

H 0.51677100 -3.42270400 1.33937800

H -0.16313800 -2.12164000 2.33193600

H -1.16945400 -3.54464700 1.92788500

C 1.51972400 -2.05615500 -1.76503200

H 1.82889700 -2.96751300 -1.22641700

H 0.74808100 -2.26752800 -2.52280400

H 2.39634500 -1.57447900 -2.23173700

C -1.42044800 -3.34797000 -1.28218100

H -0.58675600 -4.06177900 -1.40243200

H -2.28565000 -3.91988900 -0.89565300

H -1.69907500 -2.97553600 -2.28236300

Pd 0.88325100 -0.68622600 -0.39850700

O 2.48674700 -0.91529500 0.82282600

C 3.70067100 -0.28148600 0.53682000

C 4.22259500 0.30189200 1.85303100

C 4.70938500 -1.29931100 -0.00483100

C 3.51390500 0.85330200 -0.47742200

H 3.51859000 1.05739400 2.24458600

H 4.31531600 -0.49658700 2.60962100

H 5.21246200 0.78005700 1.72905100

H 4.34828100 -1.74830000 -0.94741100

H 5.69943400 -0.84636000 -0.20361000

H 4.84529100 -2.12002200 0.72140500

H 4.44593400 1.42251300 -0.65905900

H 3.18257600 0.45683600 -1.45805000

H 2.74884700 1.57064700 -0.12072900

**IM4^II^**

C -0.38675600 2.02880100 0.13076800

H 0.62904800 2.45088200 0.07539500

H -1.02911200 2.49112500 -0.64176500

H -0.81679000 2.17174300 1.14110500

Pd -0.26316200 0.06754600 -0.30508500

N -2.40694000 -0.29079100 0.13105000

C -2.58660800 -0.40549900 1.57834100

H -3.63364800 -0.67786800 1.83548500

H -1.90936000 -1.17677400 1.97928900

H -2.34064900 0.55340100 2.06087100

C -2.67898800 -1.57454000 -0.51603300

H -1.98225500 -2.34075400 -0.13520000

H -3.71842000 -1.91887400 -0.32196100

H -2.53610500 -1.48113600 -1.60487600

C -3.29172500 0.74047200 -0.40797400

H -3.09247200 1.70100100 0.09119600

H -3.10638400 0.86412800 -1.48706300

H -4.35951300 0.47477300 -0.25239400

O 1.63602900 0.11353300 -0.85563700

C 2.61917600 -0.19608200 0.09353100

C 3.93127200 -0.30183200 -0.68623000

C 2.73718600 0.90221900 1.15235600

C 2.32198000 -1.53359400 0.78212400

H 3.85297400 -1.08296400 -1.46249500

H 4.14963500 0.65339900 -1.19415600

H 4.78247600 -0.55016000 -0.02535000

H 1.78963900 0.99311700 1.71707700

H 3.54848000 0.69614500 1.87629100

H 2.94258500 1.87792000 0.67636900

H 3.11702000 -1.83552000 1.49132000

H 1.37235200 -1.46634000 1.35152000

H 2.21306900 -2.33673300 0.02968000

**IM5^IV^**

C 3.37384300 0.02247700 -1.59529100

H 4.19587100 -0.01318100 -0.86459200

H 3.34816300 -0.88675300 -2.21736400

H 3.40695800 0.94379000 -2.19637200

Pd 1.58534400 0.08373700 -0.66461000

C 2.21779900 0.20295100 1.23499300

C 1.05333800 0.26534400 2.01666000

C 1.12412500 0.24128300 3.40727800

C 2.38409700 0.18693400 4.01602500

C 3.54441100 0.15746700 3.24971400

C 3.45777700 0.15895700 1.84847700

H 0.22309500 0.25519200 4.02862900

H 2.44756200 0.16737800 5.10994500

H 4.52661800 0.12258300 3.73467700

H 4.38117400 0.12094600 1.25704800

O -0.08427400 0.37193700 1.26144100

C -1.35521200 0.60140200 1.85468600

H -1.23961100 1.08388500 2.84152400

H -1.84633000 1.33003200 1.18446500

C -2.14056900 -0.68010500 1.93138100

C -2.27942200 -1.34778800 3.08457300

H -1.87165700 -0.96169600 4.02855600

H -2.82001100 -2.30236700 3.13248900

C -2.65471000 -1.20697300 0.63841000

C -2.10750100 -2.40332600 0.15310500

C -3.63930800 -0.52128500 -0.11641700

C -2.50180500 -2.93353200 -1.07298300

H -1.33268300 -2.90563900 0.74254200

C -4.02257000 -1.08895800 -1.34495800

C -3.46336100 -2.27008300 -1.83080500

H -2.04748200 -3.86242500 -1.43829700

H -4.79122000 -0.59166200 -1.95238600

H -3.78335000 -2.67267600 -2.79938400

Si -4.54168400 1.08227600 0.36489900

C -6.32003800 0.93912700 -0.24186400

H -6.40060900 0.90919100 -1.34306900

H -6.81879800 0.03378200 0.14926200

H -6.91029500 1.81075500 0.09658600

C -3.75548100 2.54580700 -0.52813500

H -4.33006300 3.47293100 -0.34520500

H -2.71027500 2.74694800 -0.23032600

H -3.74997500 2.37622200 -1.62104400

C -4.62055600 1.39201900 2.22023100

H -5.32459400 2.22339100 2.41344000

H -5.00183000 0.50895000 2.76376300

H -3.66164500 1.67324500 2.68742700

N 0.44607200 -0.08767400 -2.73744100

C 1.01024100 0.80066900 -3.74456400

H 0.33968500 0.88469600 -4.62735000

H 1.16724400 1.79521400 -3.29712300

H 1.98008400 0.40268100 -4.08832700

C -0.86488100 0.38515000 -2.30034100

H -0.76295000 1.41059600 -1.90836600

H -1.60147300 0.37603800 -3.13472400

H -1.24195900 -0.27578700 -1.49941700

C 0.36005900 -1.45420600 -3.23378100

H 1.36864000 -1.82828500 -3.47974200

H -0.05955300 -2.10560300 -2.45360400

H -0.27228100 -1.50283000 -4.14771100

O 1.35272100 2.06808800 -1.06331000

C 1.88726000 3.21794100 -0.46845700

C 1.45593100 4.36219300 -1.39545300

C 1.30944100 3.46863600 0.92803200

C 3.41317900 3.17608200 -0.39839400

H 1.85389400 4.20549500 -2.41397800

H 0.35399900 4.40497600 -1.46495500

H 1.81710900 5.34103900 -1.02915000

H 1.69083300 2.73993400 1.66338100

H 1.56975300 4.47796300 1.29905200

H 0.20709700 3.38804200 0.90156200

H 3.82359700 4.09585700 0.05868700

H 3.75209400 2.32024700 0.21286800

H 3.85326500 3.07736900 -1.40736000

O 1.22887600 -1.90839000 -0.53257400

C 1.88767800 -2.97479000 0.09506600

C 1.62530600 -3.02689000 1.60591300

C 1.29402000 -4.23161900 -0.55714800

C 3.39372700 -2.95365000 -0.15713600

H 2.19112500 -2.25339300 2.15161200

H 0.55266100 -2.86789500 1.82067200

H 1.91701800 -4.00680900 2.02947700

H 1.45567900 -4.21191500 -1.64979600

H 1.75132100 -5.15429900 -0.15422500

H 0.20541700 -4.28415900 -0.37706400

H 3.89305100 -3.82385300 0.30853800

H 3.61516400 -2.97622800 -1.23974600

H 3.84622100 -2.04244100 0.27267500

**IM2^II^-I**

C 0.13691600 1.80748400 -0.03118900

C -0.00900900 2.56868200 1.14436100

C -0.64930000 3.80279700 1.14101600

C -1.15997600 4.25819900 -0.08207500

C -1.05442600 3.50833700 -1.25543000

C -0.39326200 2.27111700 -1.24513100

H -0.72180400 4.40055900 2.05480500

H -1.66263500 5.23233100 -0.11316800

H -1.47045500 3.89733500 -2.19103300

H -0.20025600 1.72875400 -2.17890000

O 0.60955400 2.00845500 2.19492600

C 1.04554700 0.69505500 1.79215400

H 1.99449700 0.49772700 2.31212900

H 0.27545900 -0.03067600 2.12778300

C 1.15157500 0.70020100 0.24599000

C 0.54969500 -0.53101900 -0.39562500

H 0.74122400 -1.48339400 0.11242900

H 0.79665700 -0.61907600 -1.46969200

C 2.57074300 1.04305500 -0.21821200

C 2.85208600 2.35346500 -0.62695700

C 3.60841900 0.07118000 -0.21119000

C 4.12368600 2.73776100 -1.04751900

H 2.05777000 3.10866000 -0.62145400

C 4.87860100 0.49497300 -0.64841500

C 5.14567200 1.79762100 -1.06749400

H 4.30642100 3.77250900 -1.36012400

H 5.70882200 -0.22044500 -0.66562400

H 6.15308300 2.07156300 -1.40190600

Si 3.55029400 -1.77797600 0.29691400

C 2.92285000 -2.88080100 -1.09539400

H 1.83869600 -2.82427000 -1.28857100

H 3.44174000 -2.64232000 -2.04211200

H 3.15552900 -3.93562500 -0.85664200

C 2.66446100 -2.15813000 1.92058400

H 2.83275700 -3.22852000 2.14491100

H 3.10034900 -1.58935600 2.76254500

H 1.57227300 -2.00911700 1.94090500

C 5.33123700 -2.32277500 0.61184100

H 5.32257200 -3.34040300 1.04515300

H 5.94250500 -2.37826100 -0.30668900

H 5.86273800 -1.67006800 1.32814800

Pd -1.41392000 0.00413400 -0.22263600

N -3.64801800 0.65129800 0.13454300

C -3.76278700 2.10232600 0.16362000

H -4.79996300 2.42413800 0.40868700

H -3.07938900 2.52190200 0.92091200

H -3.49271900 2.52212900 -0.81844200

C -4.03125900 0.10402200 1.43484300

H -3.35350100 0.49053800 2.21529500

H -5.07437600 0.38739500 1.69929200

H -3.94908400 -0.99483200 1.41771000

C -4.50643900 0.11127100 -0.91733100

H -4.17943500 0.49596900 -1.89796000

H -4.43088100 -0.98782800 -0.93545100

H -5.56875300 0.40096500 -0.75779700

I -1.99271900 -2.58740400 -0.25804900

**IM3^IV^-I**

C 1.02006800 1.77176700 -0.14955500

C 0.88010600 3.01017200 0.47986100

C -0.00415000 3.97070800 0.00538300

C -0.75510100 3.65008900 -1.13354100

C -0.62112000 2.42094000 -1.77807900

C 0.27659900 1.46089200 -1.28437500

H -0.10618200 4.93728200 0.50852000

H -1.46386400 4.38774900 -1.52597300

H -1.22618100 2.19510600 -2.66128300

H 0.41479300 0.50226700 -1.80614400

O 1.66842000 3.12743000 1.57787200

C 2.59067400 2.02977400 1.55413100

H 3.53251200 2.38908900 1.09991700

H 2.79522500 1.73772100 2.59636700

C 1.91846400 0.91165800 0.72655400

C 0.98891900 0.09717800 1.62826900

H 0.49578100 0.75564100 2.36932300

H 1.53652100 -0.67360700 2.20438400

C 2.83437600 0.01606500 -0.08460500

C 4.04368100 0.47397000 -0.62280100

C 2.40065400 -1.29338100 -0.37952300

C 4.83833800 -0.35453400 -1.41179900

H 4.38025300 1.50025400 -0.43910600

C 3.20991400 -2.10823200 -1.18943000

C 4.42622500 -1.65583400 -1.69413900

H 5.78499200 0.02442700 -1.81395100

H 2.88491300 -3.12949400 -1.43328500

H 5.04678700 -2.31279900 -2.31423800

Si 0.75057800 -1.99172200 0.21237000

C -0.09716400 -2.85050700 -1.23382800

H -1.08499600 -3.24280700 -0.93472000

H -0.26336300 -2.17389900 -2.08983900

H 0.51532500 -3.70373300 -1.58720000

C -1.56500000 -1.41444500 2.14668500

H -2.01437600 -2.34335000 1.76050400

H -0.75690600 -1.61879700 2.86768100

H -2.34440300 -0.77146600 2.58817200

C 1.01343300 -3.28020100 1.55342500

H 0.07790600 -3.81251500 1.79879800

H 1.72424800 -4.03449200 1.16489200

H 1.43930200 -2.88772800 2.49196400

Pd -0.83150300 -0.28975000 0.60496100

I -3.23336000 -0.23902200 -0.58613100

**IM4^II^-I**

C -0.41185200 1.99569900 0.00028300

H 0.54851300 2.44660300 0.29345000

H -0.67512400 2.28713500 -1.03316700

H -1.20496000 2.27353200 0.71981300

Pd -0.32396400 -0.01292100 0.00054300

N -2.56055800 -0.22660000 -0.00062000

C -3.08929300 0.04263700 1.33539800

H -4.18279200 -0.15193300 1.38394900

H -2.58087800 -0.59557400 2.07567200

H -2.90813000 1.09512700 1.60526400

C -2.73982000 -1.63652400 -0.34604600

H -2.20390200 -2.27176900 0.37987200

H -3.81288200 -1.92694000 -0.33697600

H -2.33270000 -1.83096300 -1.35187200

C -3.21023500 0.62871600 -0.99066700

H -3.05265300 1.68742700 -0.73183700

H -2.77567900 0.44565200 -1.98654000

H -4.30375800 0.43572200 -1.03391600

I 2.24563000 -0.14899300 0.00003500

**IM5^IV^-I**

C 3.06156500 -1.73446200 -1.19191900

H 3.94701100 -1.31851300 -0.69120700

H 2.81326300 -2.74920500 -0.84996400

H 3.14977300 -1.68388600 -2.29068800

Pd 1.53960500 -0.45442700 -0.79684700

C 1.58083100 -0.65345800 1.19589700

C 0.68060800 0.26219700 1.75395800

C 0.57017600 0.35614800 3.14213100

C 1.35871900 -0.47511100 3.94416000

C 2.24220000 -1.38803300 3.37686200

C 2.35221700 -1.48791500 1.98114500

H -0.13369900 1.06213000 3.59625200

H 1.26969100 -0.40500000 5.03391600

H 2.85140200 -2.04054000 4.01207100

H 3.03995200 -2.21528100 1.53674600

I -0.23742400 -2.41541800 -0.91695200

O 0.01294300 1.01491800 0.84088400

C -0.76793000 2.11530500 1.27018300

H -0.19198600 2.73209300 1.98874600

H -0.88710600 2.72568600 0.35791800

C -2.10041900 1.71889300 1.84852500

C -2.61581600 2.41550100 2.87345400

H -2.07317000 3.25577700 3.32873400

H -3.60818300 2.18467300 3.28039000

C -2.78352000 0.52312000 1.29197000

C -3.13354100 -0.47710200 2.21322500

C -3.07854000 0.35079100 -0.08483600

C -3.77400600 -1.64573900 1.81148000

H -2.87350200 -0.33330000 3.26941900

C -3.73501100 -0.83640900 -0.45335100

C -4.07747700 -1.82674100 0.46616100

H -4.02696700 -2.41468700 2.55056500

H -3.99027700 -1.00533900 -1.50682000

H -4.57626000 -2.74185600 0.12607100

Si -2.77148200 1.60503100 -1.48292100

C -3.88732300 1.14309100 -2.92875000

H -3.58647600 0.19503600 -3.41041700

H -4.94819600 1.04747600 -2.63459500

H -3.83284700 1.92644100 -3.70752100

C -1.01347100 1.54872400 -2.16684100

H -0.94479800 2.18568400 -3.06941400

H -0.20235300 1.86538200 -1.48826300

H -0.78476500 0.51419500 -2.49047200

C -3.27777000 3.34421600 -0.95547400

H -3.14422900 4.04970200 -1.79685000

H -4.35084100 3.35798600 -0.68909100

H -2.73425700 3.76077100 -0.09066300

O 2.53668200 1.21632700 -1.22645900

C 3.47275300 2.00477600 -0.54300400

C 4.62710700 1.17097700 0.00733500

C 3.99919900 2.99518800 -1.58642200

C 2.79433600 2.76774900 0.59636500

H 4.25713600 0.43588000 0.74682900

H 5.14404600 0.62764800 -0.80397300

H 5.37465800 1.80642200 0.51652000

H 3.16972700 3.59386000 -2.00054700

H 4.74023900 3.68501300 -1.14302000

H 4.47761600 2.45695800 -2.42251600

H 3.47523900 3.51044700 1.05212300

H 1.90365800 3.30188500 0.21899500

H 2.47261700 2.07668900 1.39611200

**TS1_MI_**

C 0.61021600 -1.49979300 0.08512900

C 0.97638800 -2.58624500 -0.71852900

C 1.57469200 -3.72694300 -0.17744200

C 1.92128600 -3.72789900 1.17120200

C 1.70254600 -2.59141300 1.96024600

C 1.06774100 -1.47813000 1.41249300

H 1.81438500 -4.56950200 -0.83519900

H 2.41336700 -4.60787200 1.60114900

H 2.02768500 -2.57539000 3.00666600

H 0.90427300 -0.58787300 2.03276100

O 0.86775500 -2.46296900 -2.06196400

C 0.60382500 -1.12534100 -2.45413300

H 1.22106300 -0.92978300 -3.35098900

H -0.46040800 -1.04521500 -2.74648000

C 0.87656600 -0.10166400 -1.35204000

C -0.07318800 0.97996800 -1.40124100

H -0.68084000 1.06791900 -2.31330100

H 0.21560900 1.95596700 -0.99732800

C 2.30435800 0.19483600 -0.96900200

C 3.28701100 -0.60507200 -1.57911500

C 2.69982700 1.21554300 -0.05683700

C 4.64635100 -0.41383100 -1.35808800

H 2.99003000 -1.42281800 -2.24219500

C 4.08739400 1.37192300 0.13775100

C 5.05436600 0.59390300 -0.49533900

H 5.37649600 -1.05842300 -1.86065300

H 4.44737200 2.14787100 0.82165100

H 6.11792500 0.77334400 -0.30041300

Si 1.72917400 2.53200600 0.96900900

C 2.83046400 3.01203300 2.43080500

H 3.19593700 2.13302500 2.99307100

H 3.71075100 3.61933700 2.15567200

H 2.23577100 3.62163100 3.13612000

C 0.07958000 2.13379500 1.78422000

H -0.17599300 3.03591900 2.37523700

H -0.78794400 1.92682300 1.13274500

H 0.14973400 1.30730000 2.51544400

C 1.53965600 4.08622400 -0.08591000

H 1.15082600 4.91523800 0.53461100

H 2.51756400 4.40678800 -0.48960600

H 0.85352000 3.97884700 -0.94460400

Pd -1.10882500 -0.27538500 -0.14549000

N -2.38201700 -1.80224200 1.07597600

C -1.84580700 -3.15718800 1.14751800

H -2.53201300 -3.82335200 1.71484300

H -1.72368800 -3.56642100 0.13067200

H -0.86490300 -3.16479300 1.64641400

C -3.70810800 -1.84225000 0.46239900

H -3.63038900 -2.25920400 -0.55638600

H -4.39827900 -2.48177400 1.05548300

H -4.09751000 -0.81603900 0.40007800

C -2.45851200 -1.19630900 2.40281300

H -1.45216400 -1.17381800 2.85677400

H -2.83122400 -0.16379600 2.29819900

H -3.13365300 -1.77145800 3.07497300

O -2.74668800 0.93602100 0.18946600

C -3.45654100 1.73144200 -0.70901000

C -4.79810300 2.04725900 -0.03325600

C -2.73529100 3.05701000 -0.98746300

C -3.72637100 0.99621200 -2.02586300

H -5.35988500 1.11823600 0.17309600

H -4.62542800 2.55509900 0.93204600

H -5.43560300 2.70002700 -0.65869100

H -1.78837500 2.89969200 -1.53146000

H -3.35121300 3.74749300 -1.59495700

H -2.49473500 3.56417700 -0.03514900

H -4.30028900 0.06904700 -1.84516800

H -4.29917900 1.61276200 -2.74494100

H -2.77757500 0.70348700 -2.51348000

**TS1'_MI_**

C 2.49320500 -0.64309600 -0.32093500

C 3.16994900 -0.64674800 0.91005300

C 4.56554300 -0.59554500 0.93503600

C 5.28243800 -0.60526800 -0.26038400

C 4.61570800 -0.67746000 -1.48607200

C 3.22128800 -0.71137700 -1.51547600

H 5.06940400 -0.57480800 1.90735200

H 6.37724500 -0.57236900 -0.23133500

H 5.18023000 -0.70315500 -2.42464400

H 2.69127700 -0.76146700 -2.47499100

O 2.53931500 -0.81069400 2.10260200

C 1.14375400 -0.67641100 2.30548600

H 0.94290800 -1.38565800 3.13130700

H 0.93422000 0.33977900 2.69682500

C 0.23584800 -0.97660500 1.13280100

C 0.87493400 -1.76772400 0.09675400

H 1.63224200 -2.49240600 0.42457100

H 0.23631800 -2.21261400 -0.67809200

C -1.23009400 -0.97833000 1.37121900

C -1.65992200 -0.86145200 2.70986100

C -2.20774800 -1.09302300 0.33949400

C -3.00190700 -0.87794600 3.06879900

H -0.92778900 -0.75412800 3.51479400

C -3.55285900 -1.13112400 0.75133700

C -3.96434000 -1.02685900 2.07853400

H -3.28669600 -0.77985100 4.12237800

H -4.34096000 -1.22346400 -0.00760000

H -5.03116000 -1.05095900 2.32822600

Si -2.22727400 -1.07015000 -1.59731800

C -3.25522400 0.43189400 -2.05984300

H -4.22740200 0.50074600 -1.54236500

H -2.66428500 1.33242300 -1.80803400

H -3.45029100 0.45057600 -3.14810300

C -3.08285100 -2.68594600 -2.07480400

H -3.27325100 -2.71233800 -3.16382000

H -2.44720300 -3.55968800 -1.83740000

H -4.05037900 -2.84875100 -1.56934100

C -0.74246900 -0.96967200 -2.76548400

H -1.18587900 -1.08386800 -3.77535200

H -0.24907500 0.01986200 -2.75927900

H 0.02880900 -1.75412900 -2.68080200

Pd 0.70457600 0.33168600 -0.39854500

O -0.39577800 1.93797600 -0.82457800

C -0.63635000 3.03136000 0.01966200

C -1.31348100 4.11568300 -0.81935400

C -1.56056900 2.59628100 1.15745100

C 0.67830400 3.56217700 0.59335900

H -0.66902500 4.39891400 -1.66999600

H -2.26980400 3.74748100 -1.23092300

H -1.52197100 5.02483900 -0.22555300

H -1.06350000 1.83142100 1.78389900

H -1.84188200 3.44105600 1.81412700

H -2.48691100 2.14425300 0.75704300

H 1.36040200 3.86489900 -0.22158300

H 0.52975400 4.43342700 1.25941400

H 1.18876200 2.76943100 1.17563300

**TS2_OA_**

C -2.49583300 -0.20764800 -0.49542000

C -3.51435100 -1.15687000 -0.42479200

C -4.85789500 -0.80206400 -0.36207100

C -5.15881500 0.56274200 -0.37897800

C -4.14937600 1.52715000 -0.45817200

C -2.80296400 1.14797800 -0.51158600

H -5.63728400 -1.56853700 -0.30612200

H -6.20837700 0.87776700 -0.33734500

H -4.41566400 2.59003300 -0.47651000

H -1.98761600 1.88322900 -0.55669000

O -3.04908500 -2.43258100 -0.39365400

C -1.66940600 -2.38158200 -0.73918700

H -1.14822700 -3.15241400 -0.14980600

H -1.57411400 -2.63142100 -1.81677700

C -1.15835100 -0.92821300 -0.46279500

C -0.24614500 -0.44571900 -1.60225700

H -0.75272600 0.29196800 -2.24671000

H 0.13970600 -1.26795700 -2.23337900

C -0.51956000 -0.88436200 0.93809000

C -1.33548200 -0.61035900 2.04018500

C 0.85403200 -1.17846400 1.15613900

C -0.83158600 -0.57297500 3.34076000

H -2.39791300 -0.39516000 1.88412500

C 1.33809300 -1.12395000 2.47690800

C 0.51707500 -0.81845500 3.56217700

H -1.50135700 -0.33736100 4.17591200

H 2.39678600 -1.32377000 2.67774800

H 0.93627800 -0.77892700 4.57437700

Si 2.11632900 -1.73480600 -0.16796100

C 3.72844600 -2.11786600 0.72917800

H 4.19278900 -1.22141100 1.17950300

H 3.58818000 -2.86131500 1.53405500

H 4.46070300 -2.54093200 0.01773400

C 2.96258200 -0.83323800 -1.76499000

H 3.07518100 -1.77203900 -2.33646900

H 2.56548900 -0.13520200 -2.54136000

H 3.96531800 -0.47434800 -1.47786100

C 1.52848600 -3.36353800 -0.92046500

H 2.38615800 -3.88709400 -1.38303200

H 1.12765000 -4.02891500 -0.13444700

H 0.75301700 -3.27193500 -1.69984200

Pd 1.37778000 0.62496600 -0.98046700

O 0.15528100 2.18895500 -0.59968400

C 0.79700700 3.01975200 0.33228000

C 0.45272000 2.59105300 1.75899500

C 2.32012700 2.95112800 0.12051400

C 0.30840800 4.44264400 0.07237900

H -0.64252300 2.60242600 1.90269100

H 0.79815600 1.55749400 1.94815300

H 0.90868100 3.25053100 2.52209300

H 2.59055900 3.24842900 -0.90938600

H 2.88201500 3.59767700 0.82101800

H 2.71720700 1.92162800 0.31991300

H 0.75953800 5.16579000 0.77733000

H 0.55181200 4.75557200 -0.95782000

H -0.78873500 4.49120600 0.18720900

**TS3_RE_**

C -0.80864400 1.70705800 0.06996400

C -0.36819900 2.79860500 -0.68020600

C 0.63363200 3.64554900 -0.21832900

C 1.19421500 3.35695300 1.03139400

C 0.76734900 2.26649500 1.79239300

C -0.24537100 1.42572500 1.30995400

H 0.96834200 4.49795000 -0.81765300

H 1.98885100 4.00527700 1.41903000

H 1.22550400 2.06300000 2.76554900

H -0.60900100 0.58109000 1.91041500

O -0.99410400 2.89514400 -1.87877000

C -2.09503100 1.97700600 -1.85580600

H -3.00955400 2.54655100 -1.60339900

H -2.21227500 1.56514300 -2.87084500

C -1.76792000 0.90823900 -0.79316800

C -0.96679900 -0.26079700 -1.39807400

H -0.06684000 0.15119200 -1.92252900

H -1.51690900 -0.75472600 -2.22426400

C -2.94521500 0.33675500 -0.02566100

C -4.06211700 1.08321300 0.36449700

C -2.82398900 -1.00904700 0.36654200

C -5.07478700 0.48530100 1.11273500

H -4.15010800 2.14362300 0.09748500

C -3.84378000 -1.58874100 1.13339700

C -4.97176200 -0.85411000 1.49446500

H -5.95219400 1.07324200 1.40658300

H -3.76125900 -2.63714700 1.45362000

H -5.77113600 -1.32082100 2.08191700

Si -1.19305800 -1.82551300 -0.13094700

C -0.40565900 -2.44679600 1.47861300

H -0.11943600 -1.65345400 2.18946000

H -1.15014600 -3.09370800 1.98741600

H 0.50022100 -3.04921800 1.29103700

C 1.74760500 -2.02037000 -1.67231600

H 2.03836600 -2.91054600 -1.08863200

H 0.95336700 -2.25796100 -2.40255900

H 2.62546800 -1.59669300 -2.18922000

C -1.45302300 -3.35717800 -1.18949200

H -0.50943900 -3.90675100 -1.35528500

H -2.13474000 -4.04940100 -0.66050800

H -1.90230600 -3.14709400 -2.17566800

Pd 1.06574100 -0.61862800 -0.37009700

O 2.63337900 -0.76557200 0.84744600

C 3.89653600 -0.23382800 0.54438000

C 4.41939200 0.35674100 1.85824200

C 4.83974500 -1.34273300 0.07148000

C 3.81512100 0.87291300 -0.50781700

H 3.76617700 1.17976800 2.19834900

H 4.42857600 -0.41606800 2.64625200

H 5.44674800 0.75037200 1.74565200

H 4.47830400 -1.79615200 -0.86786400

H 5.86539100 -0.96852600 -0.10810400

H 4.89367400 -2.14464700 0.82875500

H 4.79387600 1.36327700 -0.67187000

H 3.47833800 0.46853800 -1.48112300

H 3.09088400 1.64965300 -0.19682000

**TS4_OA_**

C 2.67036300 -2.49145400 -1.09213700

H 3.74363900 -2.72169000 -1.03456100

H 2.06144400 -3.26367900 -0.59251800

H 2.36201200 -2.34003600 -2.14043400

Pd 2.32010300 -0.68550600 -0.21248300

C 0.70791800 0.63041800 0.63616900

C 0.07525100 1.33635300 -0.41839400

C 0.34633400 2.69636400 -0.56499600

C 1.19949700 3.35363500 0.32718600

C 1.78976600 2.66704300 1.38212700

C 1.53367300 1.30329300 1.55137100

H -0.10970000 3.25338700 -1.38910800

H 1.39563300 4.42232300 0.18752800

H 2.45073900 3.18226600 2.08714000

H 1.95432500 0.76152900 2.40468600

I -0.03005800 -1.35769500 1.14719400

O -0.73355300 0.62672900 -1.23444700

C -1.77169100 1.31011100 -1.92034500

H -1.36250700 2.01809000 -2.66753000

H -2.27267300 0.50915900 -2.48996700

C -2.71735200 2.00086100 -0.97219000

C -3.18252000 3.22924400 -1.23562900

H -2.88612400 3.77388500 -2.14294200

H -3.89273000 3.72857800 -0.56439300

C -3.02877700 1.28969100 0.29949500

C -2.56674200 1.88439100 1.48442900

C -3.71966300 0.05423600 0.35061600

C -2.76184900 1.27830000 2.72333800

H -2.02044200 2.83431300 1.41999800

C -3.91486800 -0.52247700 1.61846900

C -3.44005000 0.06426100 2.79105400

H -2.38222900 1.75749900 3.63335700

H -4.46348000 -1.47020100 1.69986700

H -3.60539700 -0.42657600 3.75744000

Si -4.44713200 -0.88825700 -1.13175100

C -5.90268400 -1.91068700 -0.51117100

H -5.59707400 -2.72936600 0.16461800

H -6.64681500 -1.29593900 0.02722200

H -6.42491300 -2.38279200 -1.36385700

C -3.16821400 -2.08972400 -1.81838200

H -3.54551200 -2.60607800 -2.72068700

H -2.19922300 -1.62717800 -2.07512000

H -2.94914300 -2.86983700 -1.06579700

C -5.12802600 0.26217800 -2.45982900

H -5.69991200 -0.33213900 -3.19705400

H -5.82917200 0.99557100 -2.02113500

H -4.37620000 0.83773300 -3.02497100

O 3.88710700 -0.01908500 -1.28029600

C 5.11163400 0.18981800 -0.63228200

C 5.76400900 -1.13069000 -0.22032200

C 5.98801000 0.89369700 -1.67334100

C 4.95998800 1.08705600 0.59781100

H 5.13011600 -1.65701400 0.51872200

H 5.89008800 -1.79351000 -1.09503800

H 6.75919500 -0.97419800 0.23703800

H 5.52418500 1.84607600 -1.98317600

H 6.99635900 1.10793300 -1.27337800

H 6.09389800 0.26248900 -2.57222200

H 5.93634800 1.33478200 1.05646500

H 4.45404300 2.03145200 0.32580400

H 4.34689700 0.58317100 1.37109000

**TS5_RE_**

C 0.14461800 0.81536800 -1.23711400

H 0.18040900 1.12682800 -2.28638800

H 0.65711800 1.48693600 -0.54219800

H 0.44423000 -0.23599900 -1.08286200

Pd -1.71737000 -0.11939700 -0.25880900

C -1.25236400 0.47862700 1.59547900

C 0.04332400 0.30858300 2.11682400

C 0.39470900 0.87994100 3.34637100

C -0.56526800 1.56445600 4.09690600

C -1.87107400 1.67457900 3.63058600

C -2.19977700 1.13701500 2.37960600

H 1.41688000 0.79669400 3.73059500

H -0.28057500 2.00220600 5.06078300

H -2.63539800 2.18625600 4.22710000

H -3.22968700 1.23931900 2.01351300

O 0.89336600 -0.46429700 1.37854200

C 2.11471100 -0.88206900 1.94109000

H 1.98946200 -1.16309400 3.00564700

H 2.35694000 -1.81710600 1.40419200

C 3.21503200 0.13355400 1.77506900

C 4.07995200 0.38929000 2.76723100

H 4.00214500 -0.11776900 3.73902000

H 4.90667700 1.10011200 2.64094100

C 3.25566200 0.87486100 0.48759300

C 3.17561200 2.27424800 0.55846600

C 3.33869600 0.23532900 -0.77723000

C 3.16754800 3.06251500 -0.59071900

H 3.09175700 2.74625000 1.54565700

C 3.33130600 1.06066500 -1.91593700

C 3.23778600 2.45114300 -1.83889800

H 3.09577800 4.15342700 -0.50652000

H 3.41023600 0.60439100 -2.91154000

H 3.22886000 3.05358400 -2.75517600

Si 3.56407100 -1.63334000 -1.07843500

C 4.36581100 -1.84285400 -2.77215000

H 3.70006100 -1.55086600 -3.60432600

H 5.30007900 -1.26086500 -2.87222000

H 4.62499800 -2.90572100 -2.93430600

C 1.91579600 -2.54863200 -1.14663200

H 2.07917700 -3.64279200 -1.10884900

H 1.17605200 -2.29663200 -0.36432800

H 1.42664200 -2.34127000 -2.11669100

C 4.77025800 -2.38400900 0.16007400

H 4.98549500 -3.43125300 -0.12392000

H 5.73128100 -1.83787800 0.13509200

H 4.43774100 -2.39385200 1.21121400

N -2.45776500 -0.83219000 -2.40538200

C -3.69989300 -1.53719200 -2.10851600

H -4.15396000 -1.96479200 -3.03050600

H -3.48552400 -2.34638100 -1.39438700

H -4.42448900 -0.84128500 -1.65087500

C -1.46451800 -1.76575800 -2.92270300

H -1.23210600 -2.50903400 -2.14143900

H -1.82860500 -2.28356100 -3.83803200

H -0.54307200 -1.21722900 -3.18445100

C -2.70627300 0.24266900 -3.35579100

H -3.43858400 0.95191600 -2.93917300

H -1.77936100 0.80270900 -3.55320800

H -3.09649000 -0.15938800 -4.31735500

O -1.60759400 -2.11035800 0.08328300

C -2.08842200 -2.84555800 1.17403700

C -2.23169100 -4.27808800 0.64239500

C -1.10465900 -2.85551300 2.34812900

C -3.45076100 -2.33637000 1.64710000

H -2.94367600 -4.31393300 -0.20232000

H -1.25668800 -4.64644900 0.27518100

H -2.59172500 -4.97212600 1.42456000

H -1.04268500 -1.86449200 2.82926200

H -1.40810900 -3.58334800 3.12468000

H -0.09493500 -3.13184300 1.99364900

H -3.85791300 -2.94728900 2.47519400

H -3.36706000 -1.29528500 2.00970700

H -4.18830700 -2.35231700 0.82343000

O -1.63777500 1.75501500 -1.16810700

C -1.60476300 3.13467900 -0.82108200

C -1.27758300 3.87764700 -2.11840700

C -3.01861700 3.48218500 -0.35825000

C -0.59859400 3.53586700 0.25642300

H -0.27286700 3.59954800 -2.48623800

H -2.01019900 3.62719400 -2.90631400

H -1.28987700 4.97342700 -1.97245200

H -3.25329700 2.96751300 0.58871900

H -3.13224600 4.57019900 -0.19565900

H -3.76037400 3.16725800 -1.11352000

H -0.74876900 4.59924900 0.51730500

H -0.71824600 2.94659300 1.17926000

H 0.44583500 3.44068000 -0.08789800

**TS5'_RE_**

C 0.41913200 -1.33370000 -0.90216600

H 0.73022000 -2.38456300 -0.97009700

H 0.92657000 -0.68061400 -1.62504700

H 0.56082900 -0.95101600 0.12973600

Pd -1.49274200 -0.24337600 0.16851500

C -0.92227800 1.39151500 -0.85782800

C 0.25391800 2.03269600 -0.42879500

C 0.68562300 3.20904500 -1.05369100

C -0.04344700 3.73903800 -2.12102100

C -1.19268800 3.09936800 -2.57125900

C -1.62338400 1.92788600 -1.93533500

H 1.59005400 3.72427900 -0.71614200

H 0.30300400 4.66184200 -2.60077600

H -1.76386200 3.50531700 -3.41423600

H -2.53547900 1.43387700 -2.29144600

I -1.64949100 -1.69253000 -2.13475400

O 0.90547600 1.45836000 0.62964700

C 2.06462700 2.07322100 1.13951500

H 1.86534700 3.13053300 1.41062700

H 2.25211400 1.53977500 2.08946200

C 3.26633100 1.98136000 0.22837200

C 4.15976800 2.97861100 0.17185700

H 4.03075300 3.89847900 0.75956600

H 5.06032500 2.90802400 -0.45135800

C 3.40062900 0.77450500 -0.63236300

C 3.33448800 0.97702400 -2.01948000

C 3.59125000 -0.53217800 -0.11803100

C 3.45238200 -0.08455600 -2.91448500

H 3.17172400 1.99453800 -2.39549800

C 3.71851400 -1.58007300 -1.04822000

C 3.64653600 -1.37352400 -2.42576800

H 3.39194300 0.09984700 -3.99331500

H 3.88958700 -2.60302700 -0.68732000

H 3.74563300 -2.22042200 -3.11502300

Si 3.75447300 -0.98665000 1.71918700

C 4.68389800 -2.62140500 1.83710200

H 4.11491700 -3.47203000 1.42061900

H 5.65855900 -2.58786900 1.31706900

H 4.88840500 -2.86496000 2.89618800

C 2.04672800 -1.27052800 2.46913400

H 2.10805700 -1.45334800 3.55846000

H 1.33452200 -0.44147500 2.30681300

H 1.60038700 -2.17603000 2.01572600

C 4.75969100 0.27500200 2.68974900

H 4.96125800 -0.11801200 3.70392600

H 5.73920700 0.44444000 2.20624600

H 4.28934500 1.26446300 2.81173700

N -2.09933300 -2.08147000 1.55481700

C -3.55628100 -2.09197000 1.47034100

H -3.99826700 -2.86382600 2.13937600

H -3.93070500 -1.09804700 1.76334300

H -3.87162500 -2.29913700 0.43321900

C -1.68257200 -1.76597700 2.91982000

H -2.03811900 -0.75594500 3.17596400

H -2.08570100 -2.51219400 3.63934300

H -0.58194300 -1.77688000 2.98310700

C -1.54800500 -3.36909300 1.15608100

H -1.87021900 -3.62905400 0.13431100

H -0.44484000 -3.32820200 1.17747600

H -1.87465600 -4.18075900 1.84312300

O -2.25154100 0.80648600 1.73117300

C -3.05276100 1.95484300 1.75467400

C -3.68322000 1.95360300 3.15480900

C -2.23870900 3.24025700 1.57956700

C -4.15750000 1.88417400 0.70140100

H -4.26460900 1.02753600 3.31713800

H -2.89704900 1.99902400 3.92948200

H -4.36061400 2.81585900 3.29849900

H -1.88564700 3.36068400 0.54195500

H -2.83827500 4.13541200 1.83207600

H -1.35374200 3.22010600 2.24148400

H -4.82526000 2.76577300 0.73907800

H -3.71888400 1.83605100 -0.31124700

H -4.77891800 0.98068000 0.84388800

**TS5''_RE_**

C 0.14712900 0.59811800 -1.31175100

H 0.08712400 0.67293500 -2.40304600

H 0.77435300 1.36292300 -0.84359200

H 0.40348500 -0.40939900 -0.94042700

Pd -1.74220600 -0.00865400 -0.17034500

C -1.15433300 0.67210300 1.62117800

C 0.14113600 0.46483900 2.12401500

C 0.54494500 1.12064500 3.29415600

C -0.36150500 1.91829600 3.99707000

C -1.66933100 2.06367000 3.54517100

C -2.05634500 1.43743000 2.35446800

H 1.56890900 1.01293400 3.66752600

H -0.03388600 2.41756000 4.91630700

H -2.39081000 2.66785800 4.10721600

H -3.08310900 1.56692400 1.98797500

O 0.93761800 -0.39478100 1.42582800

C 2.15988100 -0.82025500 1.98316500

H 2.05502700 -1.02291800 3.06684700

H 2.35104500 -1.79758900 1.50460200

C 3.29094200 0.13958500 1.71865700

C 4.19319200 0.42780600 2.66726900

H 4.12702100 -0.01158600 3.67232200

H 5.03996400 1.09814100 2.47136200

C 3.32119800 0.79503300 0.38400700

C 3.23915000 2.19607600 0.36635900

C 3.40043800 0.07735900 -0.83762800

C 3.22416100 2.90991100 -0.83020800

H 3.15887100 2.72978300 1.32194000

C 3.39169100 0.82897700 -2.02685100

C 3.29752700 2.22107400 -2.03733700

H 3.14248500 4.00362200 -0.81537600

H 3.47039200 0.31062800 -2.99156800

H 3.28479000 2.76469800 -2.98962400

Si 3.60658700 -1.80677800 -1.02552800

C 4.42730600 -2.12862400 -2.69156600

H 3.77856400 -1.87671400 -3.54971800

H 5.37148400 -1.56683900 -2.81180800

H 4.67302200 -3.20266500 -2.78660600

C 1.93468700 -2.67781300 -1.06740600

H 2.06582800 -3.77635300 -1.07436700

H 1.25012100 -2.43587200 -0.23408700

H 1.40599300 -2.41714800 -2.00365700

C 4.76595200 -2.51895000 0.27700300

H 4.98521000 -3.57550500 0.03372600

H 5.73012500 -1.97807800 0.27058400

H 4.39445900 -2.49637400 1.31467200

N -2.70000000 -0.80249800 -2.22453100

C -4.04342000 -1.26117000 -1.87310900

H -4.58425700 -1.64138200 -2.76807700

H -3.98391100 -2.06435600 -1.12125500

H -4.62056600 -0.42493800 -1.44372000

C -1.90066700 -1.91650400 -2.72576400

H -1.80392800 -2.69160900 -1.94676600

H -2.35878700 -2.37002700 -3.63266700

H -0.89108800 -1.55976800 -2.99170000

C -2.80141900 0.23384800 -3.24657200

H -3.39412600 1.08050200 -2.86874300

H -1.80411300 0.61845200 -3.50801000

H -3.28363400 -0.16810900 -4.16516900

O -1.51774200 1.78398700 -1.24715800

C -1.17755500 3.15376300 -1.07739300

C -0.37548500 3.58683600 -2.30769600

C -2.52952300 3.87078100 -1.06228200

C -0.41181300 3.50704300 0.19523600

H 0.62646500 3.12214800 -2.32389000

H -0.90421500 3.29604300 -3.23361900

H -0.23208100 4.68268100 -2.32635700

H -3.13782100 3.52574500 -0.20799500

H -2.39696800 4.96512800 -0.97337100

H -3.09226100 3.66584100 -1.98996100

H -0.10576700 4.56904300 0.15658700

H -1.02387200 3.36654300 1.09927000

H 0.50679700 2.90930400 0.32129700

I -1.99856100 -2.34197500 1.07123400

**TS5_RE_^ArMe^**

C -0.08481100 1.15277700 -0.35703900

H -0.42821100 1.13012700 -1.41306900

H -0.18240000 2.17117000 0.03560100

H 0.94708400 0.78840200 -0.32897300

Pd -1.75190800 -0.18421100 -0.11173500

C -0.48530500 0.29427500 1.44447700

C 0.73382000 -0.33491000 1.80375600

C 1.16249100 -0.30955900 3.13710300

C 0.38756600 0.30428800 4.12077100

C -0.81578400 0.91714300 3.78239700

C -1.23345000 0.92021400 2.45170700

H 2.11323800 -0.77373000 3.41619000

H 0.73844400 0.30071500 5.15927700

H -1.43678800 1.39253500 4.54998600

H -2.18738600 1.37680800 2.17698700

O 1.44362600 -0.92160600 0.81533600

C 2.72129500 -1.45849600 1.06681700

H 2.70033200 -2.17350300 1.91340800

H 2.92921400 -2.06092000 0.16432400

C 3.77563700 -0.40077800 1.27799000

C 4.78237900 -0.59984200 2.14087700

H 4.85598900 -1.52206300 2.73395400

H 5.57968600 0.14242600 2.27296000

C 3.61072500 0.88084500 0.54220000

C 3.51453500 2.04468500 1.32123800

C 3.50895500 0.96548000 -0.87052000

C 3.31255100 3.29430300 0.74016200

H 3.57569000 1.95294200 2.41277500

C 3.31790100 2.24365900 -1.42487400

C 3.21028900 3.39454700 -0.64431000

H 3.23051100 4.18605100 1.37244300

H 3.25120600 2.35280000 -2.51509000

H 3.04986500 4.36870800 -1.12116000

Si 3.65305800 -0.47946000 -2.10280700

C 4.16890300 0.23883500 -3.76842000

H 3.37898400 0.85335300 -4.23657000

H 5.07829100 0.86287300 -3.69606700

H 4.39185500 -0.58113500 -4.47633900

C 1.99007800 -1.32365800 -2.37266900

H 2.10543800 -2.19261500 -3.04820000

H 1.45716500 -1.66388700 -1.46683600

H 1.30834000 -0.61522400 -2.88102600

C 5.02854000 -1.67382100 -1.61552600

H 5.15034700 -2.44005800 -2.40402400

H 5.98918500 -1.13128500 -1.54156400

H 4.89314700 -2.20831300 -0.66091700

O -3.07682700 1.41297600 -0.01050900

C -3.12318000 2.80253400 -0.17817500

C -2.48019400 3.58406800 0.97788900

C -2.51774700 3.29626900 -1.50023100

C -4.62390800 3.13533900 -0.18578900

H -2.97096800 3.34467900 1.93802100

H -1.40403200 3.36407200 1.08960300

H -2.57802100 4.67473600 0.82170300

H -3.01435800 2.83899100 -2.37167900

H -2.63398800 4.39142700 -1.59798700

H -1.43899400 3.08064300 -1.57197800

H -4.79752100 4.22485700 -0.26779400

H -5.13248700 2.64273000 -1.03384200

H -5.09767500 2.77645300 0.74431100

N -3.17984600 -0.90606500 -1.85390900

C -2.48423400 -1.89241900 -2.67344100

H -1.63394400 -1.40659100 -3.18092800

H -2.07468500 -2.68658600 -2.03481300

H -3.16394900 -2.31744600 -3.44387300

C -4.33928600 -1.48463900 -1.18648500

H -4.02781500 -2.35597800 -0.58967400

H -4.78713600 -0.73066900 -0.51657900

H -5.10763100 -1.81807900 -1.91911300

C -3.60101700 0.21374900 -2.68421600

H -4.12408300 0.95220000 -2.06153800

H -2.71690200 0.69346400 -3.13682700

H -4.27137600 -0.13204200 -3.50199800

O -1.01817500 -2.10299600 -0.18302200

C -1.28162600 -3.06362700 0.80779800

C -1.90164400 -4.27468400 0.08790700

C 0.01253300 -3.54222000 1.48032500

C -2.25402300 -2.57600600 1.88557100

H -2.88156900 -4.02476600 -0.35583700

H -1.23803600 -4.61050700 -0.72909000

H -2.05489800 -5.12678300 0.77622000

H 0.41708700 -2.78599500 2.17336000

H -0.15567600 -4.46347800 2.06955200

H 0.77709600 -3.75967000 0.71254900

H -2.56027900 -3.40591700 2.55042200

H -1.79954400 -1.79318100 2.51768000

H -3.17489700 -2.15122300 1.44035400

**TS6_RE_**

C -0.89090100 -1.89666800 0.06073100

H -1.83893900 -2.17001100 0.54533900

H -0.75376900 -2.44523000 -0.87881800

H -0.06058100 -2.08130200 0.78040100

Pd 0.38620200 -0.04496400 -0.25340100

N 2.54570400 0.15512900 0.12526600

C 2.82443800 -0.04296800 1.54546400

H 3.91269400 0.05848700 1.76503200

H 2.27070700 0.69877400 2.14265500

H 2.49469800 -1.04870400 1.85312600

C 2.95046700 1.50089400 -0.28101200

H 2.39188100 2.25054200 0.30205900

H 4.04153800 1.66280400 -0.12087400

H 2.72088800 1.65279500 -1.34762000

C 3.26241000 -0.84232300 -0.66651800

H 2.93181200 -1.85345900 -0.37832500

H 3.04033800 -0.69959600 -1.73571300

H 4.36415200 -0.76836500 -0.51293800
